# Probing ion channel functional architecture and domain recombination compatibility by massively parallel domain insertion profiling

**DOI:** 10.1038/s41467-021-27342-0

**Published:** 2021-12-08

**Authors:** Willow Coyote-Maestas, David Nedrud, Antonio Suma, Yungui He, Kenneth A. Matreyek, Douglas M. Fowler, Vincenzo Carnevale, Chad L. Myers, Daniel Schmidt

**Affiliations:** 1 Department of Biochemistry, Molecular Biology & Biophysics, University of Minnesota, Minneapolis, MN 55455 USA; 2 Department of Chemistry, Temple University, Philadelphia, PA 19122 USA; 3 Department of Genetics, Cell Biology & Development, University of Minnesota, Minneapolis, MN 55455 USA; 4 Department of Pathology, Case Western Reserve University School of Medicine, Cleveland, OH 44106 USA; 5 Department of Genome Sciences, University of Washington, Seattle, WA 98115 USA; 6 Department of Bioengineering, University of Washington, Seattle, WA 98115 USA; 7 Department of Computer Science and Engineering, University of Minnesota, Minneapolis, MN 55455 USA

**Keywords:** Potassium channels, Protein design, Molecular biophysics

## Abstract

Protein domains are the basic units of protein structure and function. Comparative analysis of genomes and proteomes showed that domain recombination is a main driver of multidomain protein functional diversification and some of the constraining genomic mechanisms are known. Much less is known about biophysical mechanisms that determine whether protein domains can be combined into viable protein folds. Here, we use massively parallel insertional mutagenesis to determine compatibility of over 300,000 domain recombination variants of the Inward Rectifier K^+^ channel Kir2.1 with channel surface expression. Our data suggest that genomic and biophysical mechanisms acted in concert to favor gain of large, structured domain at protein termini during ion channel evolution. We use machine learning to build a quantitative biophysical model of domain compatibility in Kir2.1 that allows us to derive rudimentary rules for designing domain insertion variants that fold and traffic to the cell surface. Positional Kir2.1 responses to motif insertion clusters into distinct groups that correspond to contiguous structural regions of the channel with distinct biophysical properties tuned towards providing either folding stability or gating transitions. This suggests that insertional profiling is a high-throughput method to annotate function of ion channel structural regions.

## Abstract (web summary)

Here, the authors perform a large-scale, high-throughput biochemical assay to determine the compatibility of over 300,000 domain recombination variants of the inward rectifier K^+^ channel Kir2.1. They derive rules for designing domain insertion variants that fold and traffic to the cell surface and conclude that the insertion of domains at protein termini is evolutionary favoured.

## Introduction

Most metazoan proteins are comprised of multiple domains, with distinct tertiary structure elements and functions^1,2^. Domains are the words of the protein universe^3^, and several dictionaries have been compiled (e.g., SMART, Pfam, SCOP2, CATH). The omics era provided key insight into how multidomain proteins evolve. Rearrangement of existing protein domains^4–6^, mostly at termini^7–9^, is a sufficiently effective strategy to rapidly diversify protein function^2,10,11^.

The potassium (K^+^) channel superfamily exemplifies this rapid functional diversification through domain rearrangement. This family first appeared in prokaryotes and expanded into distinct structural and functional classes during metazoan evolution^12^. Different sub-families arose from appending domains at the termini of the K^+^-selective pore, such as adding RCK domains in K_Ca_, cyclic nucleotide-binding (CNG), voltage-sensing domains in voltage-dependent K^+^ channel (Kv), and an immunoglobulin (Ig)-like C-terminal Domain (CTD) in inward rectifier K^+^ channel (Kir)^12,13^. The same ancestral architecture of Kir, with minor adaptation in eukaryotes^14^, was leveraged throughout the evolution of the Kir family for regulation by allosteric ligands at different sites, all of which couple to pore domain gating^15^.

Considering wide-spread domain recombination, do different domains have different intrinsic combinatorial propensity? Frequently recombined domains often encode broadly useful functions (e.g., SH2 domains dock to phosphorylated residues), but the evidence for a causal link between function and likelihood of combinatorial expansion is ambiguous^16–18^. Although some studies hint at a biophysical basis of domain compatibility^17,18^, comprehensive studies are needed to test whether these correlations reflect genuine biological mechanisms that underlie a domain recombination grammar^19^. We must go beyond domain combinations founds in extant proteins, which are biased towards combinations that work, and broadly explore domain recombination space for a global picture of protein domain compatibility. Are extant proteins examples of what multidomain architectures have to be, or are they sampled from a much larger set of possible multidomain protein architectures?

Knowing the answer to this question has implications beyond natural proteins; domain recombination-based approaches are also used to generate synthetic proteins in biomedical engineering^20^. Many biosensors and protein switches are made by combining domains that sense the desired property (e.g., ligand, voltage, aberrant protein activity) and domains that respond to these events (e.g., fluorescence, alter gene expression, induce apoptosis)^21–23^. Domain recombination is a key tool in synthetic biology for designing programmable circuits from multidomain proteins in living cells^24,25^. Light-gated K^+^ channels, useful as optogenetic tools, can be constructed from domain recombination^26,27^. Despite these successes, synthetically recombined proteins that fold and function well are typically the result of trial-and-error and iterative optimization^28–34^. Only a few synthetic multidomain proteins emerged from explicit rule-based proteins design^35–41^. Deriving practical rules to accelerate domain recombination-based protein design is challenging because structure/function relationships and folding/unfolding equilibria of isolated and recombined domains can differ^42,43^. Computational methods have become increasingly useful in protein engineering; however, they are most successful for designing stable folds^44–47^ or simple, non-allosteric multi-component protein switches^48^. Systematic domain recombination studies, therefore, would not only provide insight into the fundamental biological mechanism of multidomain protein evolution but also help improve the rule-based and computational design of synthetic proteins.

**Fig. 1:**
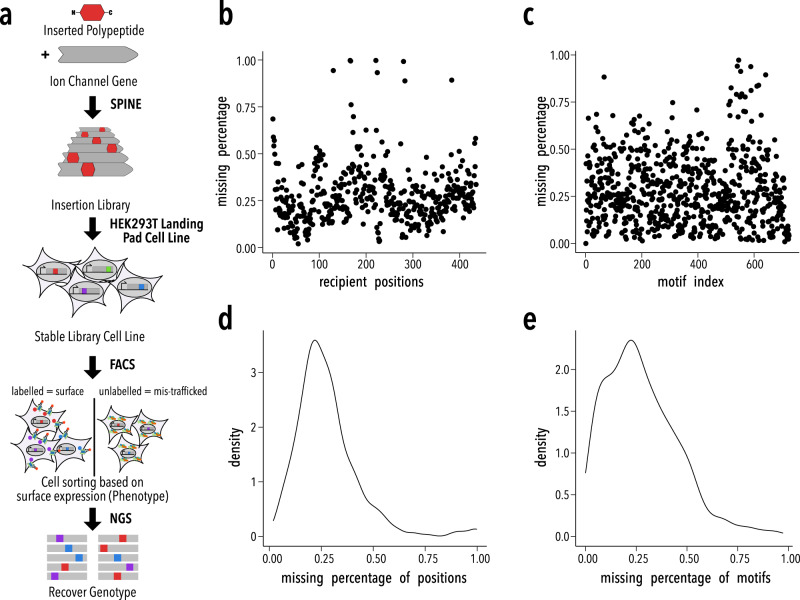
Large-scale insertional fitness profiling. **a** Motifs are inserted into all positions of a recipient protein using SPINE^53^. A stable single-copy insertion library is generated by BxBI-mediated recombination in HEK293T^54^. Cells are sorted based on channel surface expression (the phenotype) as determined by antibody labeling of an extracellular FLAG tag. Genotypes of each sorted cell population are recovered by NextGen Sequencing (NGS). **b**–**c** Scatterplots with the percent missing of Kir2.1 insertion fitness data after alignment by (**b**) position and (**c**) motif. **d**–**e** Density plots of Kir2.1 insertion fitness data percent missing by (**c**) position and (**d**) motif.

To produce a rich dataset from which these potential insights and improvements can be sourced, we generated 759 polypeptide motif (donor) insertions at all 435 amino acids of the Inward Rectifier K^+^ channel Kir2.1 (recipient) and measured cell surface expression of the resulting channel/insertion variants. Previously, we found surprising variability between three motif’s insertional profiles, which implies complex constraints on donor-recipient compatibility^27^. We chose 759 donor motifs as a representative sample to exhaustively study compatibility (Supplementary Table 1, Supplementary Data 1, see Supplementary Table 2 for summary statistic of biophysical properties). They comprise everything from small motifs to larger domains, hydrophobic filaments, flexible linkers, and disordered fragments. Larger domains include motifs often used in protein engineering (e.g., the phototropin LOV2 domain), and domains commonly found in extant proteomes (e.g., SH2 domains). We included all PDB entries <50 AA used in a global analysis of protein folding^49^, conserved structural motifs in proteins (smotifs^50^), peptide motifs enriched in all known protein folds^51^, disordered fragments, and domains curated from DisProt^52^, cysteine-rich peptide toxins, and short polypeptide linkers. The massive scale of these experiments (over 300,000 variants) is made possible by incorporating recent technology advances in the form of unbiased insertional libraries^53^ and rapid construction of single copy, stable variant library cell lines^54^ (Fig. 1).

## Results

### Systematic motif insertions reveal strong fitness pattern consistent with known ion channel biochemistry

For Kir2.1 to conduct K^+^ ions and maintain cellular excitability^15^, it must first fold, tetramerize, and traffic to the plasma membrane (collectively referred to as surface expression from hereon)^55–59^. While the impact of domain insertion on surface expression is distinct from its impact on function (K^+^ permeation)^27^, measuring how domain recombination affects surface expression provides a valuable perspective that is relevant to the evolution of ion channels^12^, engineering of synthetic ion channels^26,60^, and understanding the mechanism of ion channel mutations that cause defects in folding and trafficking^61^.

**Fig. 2:**
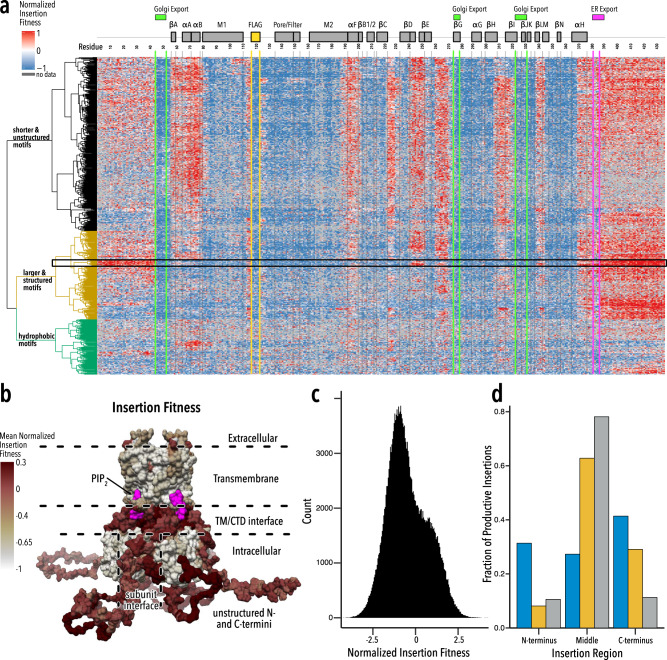
Heatmap of insertion fitness. **a** Insert fitness, normalized to a control flexible linker motif (see Methods) for 759 motifs inserted into all positions of Kir2.1. Secondary structural elements (gray boxes) are Kir2.1 are shown above, along with known Golgi and ER export signals (green and magenta boxes, respectively). Motifs are hierarchically clustered using a cosine distance metric. Dendrograms are colored by major motifs groups. The black box indicates a subset of well-structured motifs (Supplementary Table 1, Supplementary Data 1). **b** Mean normalized insertion fitness mapped onto the structure of Kir2.2 (PDB: 3SPI^65^; 70% identity with Kir2.1; residues 1–40 and 379–410 are modeled). **c** Distribution of normalized insertion fitness. **d** Productive insertions at N- or C-termini (first or last 45 residues) or the middle of Kir2.1 (342 residues) for commonly recombined motifs (e.g., SH2 domains; blue bars; see Supplementary Table 1), short peptide linker (AGSAGSA; yellow bars). Gray bars indicate a random distribution.

As we did in smaller scale Kir2.1 domain insertion studies^27^, we measure the impact of insertions on surface expression through fluorescent antibody labeling and fluorescently activated cell sorting coupled to sequencing (Fig. 1a). We calculate surface expression fitness of insertion variants as enrichment or depletion of surface-expressed vs. non-surface-expressed variants (Fig. 2a–c, Supplementary Figure 1). Note that the specific implementation of this assay prevents us from including wildtype Kir2.1 as one of the variants for which we determine surface expression. Fitness of domain insertion variants is therefore *z* score normalized to the effect (averaged across all Kir2.1 positions) of inserting a flexible linker motif (amino-acid sequence AGSAGSA). Thus, positive insertion fitness means this insertion variant is trafficking better than insertions of a small flexible linker on average, zero is neutral, and negative fitness is worse trafficking.

Overall, surface expression fitness is consistent with expected biochemistry. Insertions into the extracellular FLAG tag, used to label surface-expressed Kir2.1, mimic decreased fitness because they disrupt antibody binding. Motif insertions into transmembrane regions (M1, M2, Pore, Filter) strongly decrease fitness (Wilcoxon rank-sum test *p* value <2.2e-16) presumably by impairing membrane insertion of the nascent protein^57,62^. Insertions in folding-critical core beta-sheets of the CTD^13^ also decrease fitness. This aligns with many disease-associated mutations impairing forward trafficking by affecting the core stability of the CTD^13,61,63,64^. Conversely, most insertions in the unstructured N- or C-termini are tolerated. As expected, insertions into Golgi export signals decrease surface expression (Fig. 2a, green lines). This is particularly strong for an N-terminal signal with a tertiary structure (Fig. 2a, positions 46–50^56^). On the other hand, insertion phenotypes in an ER export signal (the linear diacidic FCYENE signal^55^, Fig. 2a, positions 382–387) are more varied with some not affecting surface trafficking. Perhaps the specific residue orientation that is required for function in structured export signals renders them more sensitive to motif insertion, while linear unstructured signals that rely on localized charge or hydrophobicity are more robust.

Although many insertional fitness patterns are consistent with known biochemistry and therefore expected, the relatively high insertion fitness of the interfacial helix (aka slide helix, αA/B) and tether helix (αF) is surprising, given their prominent roles in allosteric ligand binding and gating^65^ and several known mutations that impair cell surface localization in Andersen–Tawil syndrome^61,66^. Homologous mutations in Kir1.1 and Kir6.2 also have defective trafficking phenotypes and are associated with Bartter Syndrome and hyperinsulinemic hypoglycemia type 2, respectively^61^. In this context, it is important to remember that our assay measures surface-expression fitness and does not assess channel function, which is a distinct phenotype. It is possible that channel function is impaired after insertions into interfacial and tether helices, but that these regions have an intrinsic capacity to accommodate the insertions such that the channel subunit can fold, assemble into a tetramer, and traffic to the cell surface.

Taken together, our data confirm many expected motif insertion effects, which shows that our approach is working. Nevertheless, the variability of insertion fitness across donor motifs and recipient insertion sites (trafficking motifs, tertiary structure elements) implies more complex mechanisms for domain compatibility.

### Recipient and donor properties interact to determine insertion fitness

To learn if donor properties affect fitness, we hierarchically clustered insertion fitness by motif revealing three groups enriched for short unstructured motifs, larger folded motifs, and hydrophobic motifs (Fig. 2a, Supplementary Fig. 3).

Hydrophobic motifs are most distinct from other motifs groups by having less insertional fitness across the gene. In qualitative terms, hydrophobics have decreased in fitness in regions that are more often compatible with the other two motif groups, such as the N terminus. On the other hand, some hydrophobic motifs can be inserted where no other motifs can (e.g., beginning of M1 and end of M2 transmembrane helices).

Short unstructured motifs are allowed in more parts of Kir2.1 than the other groups and seem to have little variability between motifs. Short unstructured motifs are unsurprisingly enriched for in flexible loops connecting secondary structure elements (e.g., βCD, βDE, or βHI) in the CTD. Again, it is a surprise that insertions into the slide helix (αA/B) and the tether helix (αF, also known as C-linker) are largely permitted. Both helices undergo conformational changes during Kir2.1 gating, so compatibility with the insertion of unstructured motifs may reflect a higher degree of conformational plasticity. Insertions into N- or C-termini are generally allowed, but not consistently in every position.

The group of larger structured motifs contains nearly all motifs longer than 90 amino acids. This group is most allowed at the termini—more than any other group—and occasionally in structured Kir2.1 regions. For a cluster of motifs enriched for frequently recombined domains (e.g., SH2; Supplementary Table 1) this effect is particularly prominent and, apart from the βDE loop, they are only allowed in the termini with the N terminus having a particularly strong signal in comparison to the rest of the protein (Fig. 2a, black box). If the biophysical properties of an insertion determine whether it is beneficial at the termini, we would expect different enrichment of folded domains vs. unstructured peptides at termini compared to within the protein. To test this premise, we compared the fraction of insertions with high surface expression fitness at termini (45 terminal residues) versus the middle of Kir2.1 for either structured frequently recombined domains and unstructured short peptides (Fig. 2d). Most (~65%) productive short peptide insertions are found in the middle of Kir2.1, whereas 72% of productive promiscuous domain insertions occur at either N- or C-termini. Interestingly, this number is very close to the reported number of evolutionary domains gains at protein termini in the human genome (~71%)^67^. A likely mechanism for this observation is that—compared to unstructured peptides—well-folded domains, when inserted at termini, can provide a stabilizing influence on a recipient protein in the form of chaperone-like activities (preventing aggregation) or passive folding assistance whereby folding of one domain can promote folding of others^43,68,69^. As previously established in several other fusion proteins, adding structured domains on N- or C-termini is also a common strategy to improve the biochemistry and structural biology of hard-to-fold proteins^70–72^.

Biophysical mechanisms, such as the impact of motif gain or loss on protein folding, likely played a major role in shaping the evolution of multidomain proteins. Perhaps multidomain proteins became enriched in metazoans because both genomic mechanisms (intron formation, loss of stop codon, exon extension, etc.^9^) act in concert with biophysical mechanisms to favor the gain of larger structured domains at protein termini. Domain gain at termini is less likely to disrupt the folding and function of the rest of the protein. It may increase folding robustness and stability of the fusions protein by reducing topological frustration^73^ or increasing folding cooperativity^68,69^. Because larger structured domains are more likely to encode useful functions (compared to shorter unstructured motifs), they are more likely to provide a selectable advantage to the resulting recombined protein.

**Fig. 3:**
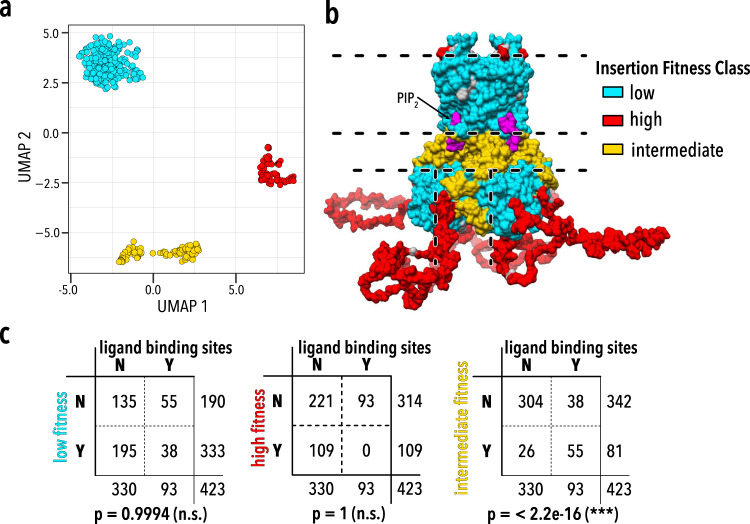
Unbiased clustering of insertion fitness. **a** Uniform Manifold Approximation Projection (UMAP) was used to cluster the insertion fitness of each channel. Cluster membership of each residue is indicated by color. Optimal cluster number was determined using the R package Nbclust using the majority rule. **b** UMAP classification clusters are mapped onto the structure of Kir2.2 (PDB: 3SPI^65^; 70% identity with Kir2.1; residues 1–40 and 379–410 are modeled). Fitness classes describe conformationally rigid and structured pore domain and CTD beta-sheet core (low fitness; cyan), highly flexible and unstructured N/C termini (high fitness; red), and structured yet dynamic interface between TM and CTD, or between subunit in the CTD (intermediate fitness; yellow). PIP_2_ (Kir2.1’s activator) is shown in magenta. **c** The intermediate fitness cluster is enriched for Inward Rectifier K^+^ channels allosteric modulator and ligand binding sites Independence of binding sites (PIP_2_ – Kir2.1, Kir3.1, Kir6.2, Gβγ–Kir3.1 only, ATP–Kir6.2 only; see Supplementary Table 7 for annotation) with respect to different residue classes identified by unbiased clustering of insertion fitness was tested using two-sided Fisher’s Exact tests, *p* values are shown. Only the intermediate fitness class (colored yellow in **a**–**b**) is enriched for ligand binding sites.

To learn if there are commonalities in surface expression phenotypes among different insertion positions, we used Uniform Manifold Approximation and Projection (UMAP^74^). Three distinct insertion position clusters emerge (Fig. 3a) corresponding to contiguous regions of Kir2.1 (Fig. 3b). These regions represent the (1) pore domain and CTD core beta-sheets, (2) unstructured N- and C-termini, and (3) PIP_2_ (Kir2.1’s activator) binding sites, interfaces between the pore domain/CTD, and monomer interfaces within CTD. The emergence of discrete yet contiguous Kir2.1 regions from unbiased clustering of surface expression fitness suggests commonalities in local Kir2.1 properties in these regions that influence fitness.

**Fig. 4:**
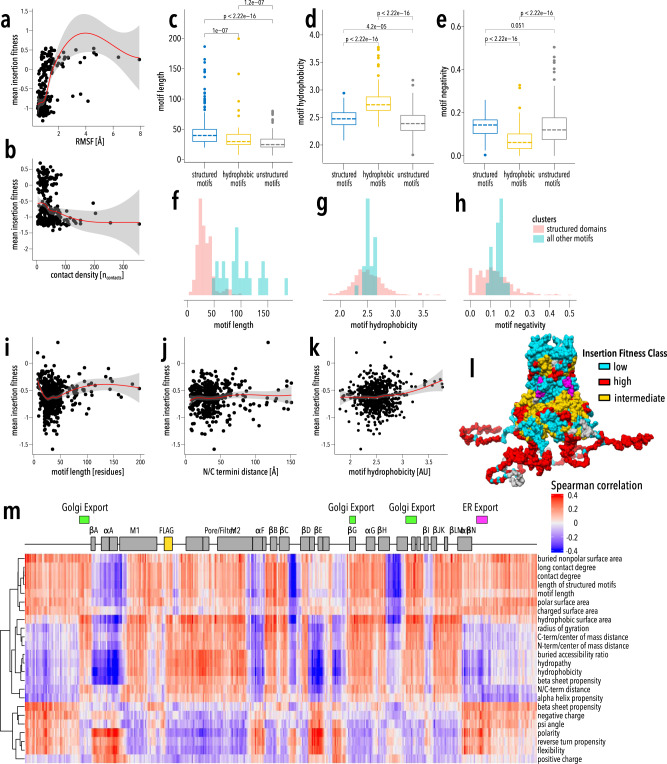
Relationships between fitness data and computed properties. Pairwise scatterplots between recipient properties (**a** RMSF, **b** contact density) and insertion fitness. A LOESS regression curve is fitted to each scatterplot, with the red line representing the fit and the gray area representing the 95% confidence interval. **c**–**e** Boxplots of motif (**c**) length, (**d**) hydrophobicity, and (**e**) negativity across the three motif clusters from Fig. 2a. Cluster samples sizes: Structured motifs (*n* = 168), unstructured motifs (*n* = 317), hydrophobic motifs (*n* = 151). Median is marked with a dashed line, the vertical length of the box represents the interquartile range (IQR), upper fence: 75th percentile +1.5 × IQR, lower fence: 25th percentile −1.5 × IQR, outlier points and *p* values from pairwise Wilcoxon tests are shown. **f**–**h** Density plots of motif (**f**) length, (**g**) hydrophobicity, and (**h**) negativity of the cluster of well-structured domains (black box in Fig. 2a) and all other motifs. Density is weighted by group size to allow direct comparison. **i**–**k** Pairwise scatterplots between motif properties (**i** motif length and **j** NC termini distance, **k** motif hydrophobicity) and insertion fitness. A LOESS regression curve is fitted to each scatterplot, with the red line representing the fit and the gray area representing the 95% confidence interval. **l** Hierarchical clusters of motif properties correlations with Kir2.1 position (Supplementary Fig. 3) is mapped onto the structure of Kir2.2 (PDB: 3SPI^65^; 70% identity with Kir2.1; residues 1–40 and 379–410 are modeled). The regulator PIP_2_ is shown in magenta. **m** Spearman correlation plot between motif properties and the fitness of that motif at each Kir2.1 position. Properties are hierarchically clustered.

To test if specific biophysical properties influence insertion fitness, we calculated sequence-, structure-, and dynamics-based properties of inserted motifs (Supplementary Table 2, Supplementary Data 4) and recipient Kir2.1 (Supplementary Table 3, Supplementary Data 5). We find that insertion fitness has a weak to moderate correlation with Kir2.1 backbone flexibility (molecular dynamics-derived root mean square fluctuation and anisotropic network model-derived stiffness; Pearson correlation coefficient 0.48 and −0.32, respectively, Fig. 4a) implying that Kir2.1 rearranges structurally after motif insertion. Available space at insertion sites (e.g., contact density) was only weakly correlated with insertion fitness (Spearman correlation coefficient −0.21, Fig. 4b). Previously discussed inserted motif clusters have distinct property distributions (Fig. 4c–h), which implies that the pattern of insertion fitness correlates with the biophysical properties of the motif. This is illustrated by a subcluster comprised of longer motifs that are commonly recombined in evolution containing hydrophobic and negatively charged residues (black box in Fig. 2a, Fig. 4f–h).

While motif properties are clearly important, they behave non-linearly. For example, the correlation of insertion fitness with motif length is negative for motifs under 25 amino acids but becomes positive for longer motifs (−0.31 and 0.18 Pearson correlation coefficients, respectively, Fig. 4i). All motif properties correlate positively and negatively with fitness dependent on insertion position. Motif lengths, for example, are positively correlated in flexible termini and loops but negatively correlated in the G-loop (Fig. 4m). This suggests that our data provide highly resolved information about both donor motifs and the recipient channel that may hint at the rules that govern domain compatibility. Hierarchical clustering of correlations between fitness and motif properties at each residue separates Kir2.1 into three distinct classes (Fig. 4l, Supplementary Fig. 4). Within each class, the correlation sign (positive or negative) between fitness with inserted donor properties is identical. For example, all residues in the pore domain and beta-sheet core of the CTD class correlate positively with motif hydrophobicity and negatively with motif polarity (Supplementary Fig. 4). Furthermore, the classes that emerge from hierarchical clustering of fitness/calculated property correlation are similar to those that emerge from UMAP clustering of fitness alone (compare Fig. 3b and Fig. 4l, Pearson’s χ2 test *p* value <2.2e-16, Cramer’s V 0.42). This strongly points to insertion fitness being influenced by the inserted motif’s properties.

Taken together, we can draw two important qualitative conclusions from this systematic quantification of domain recombination compatibility. First, insertional compatibility is based on biophysical properties of both inserted motif (donor) and Kir2.1 (recipient). Second, recipient and donor properties interact, often non-linearly, to determine insertion fitness.

### Machine learning reveals the basis for donor/recipient compatibility

To further identify which donor and recipient properties are important and how they interact in compatible insertion variants, we used Machine Learning (ML). While ML methods are sometimes treated as black boxes, they are useful for exploring and interpreting rich genotype/phenotype datasets with non-linear interactions^75^. We trained and tested regression random forests to predict insertional fitness at every amino-acid position based on recipient and motif properties. To identify the most important properties and aid interpretation, we reduced properties from over 900 to 10 based on redundancy and feature importance with little impact on performance (Supplementary Fig. 5, Supplementary Table 4). The final model successfully predicts ~40% insertional fitness variance in data withheld from model training. It performs better when predicting fitness in recipient position (for all inserted motifs) when compared with the mean fitness effect (for all insertion positions) of a motif (Supplementary Fig. 6e–h).

**Fig. 5:**
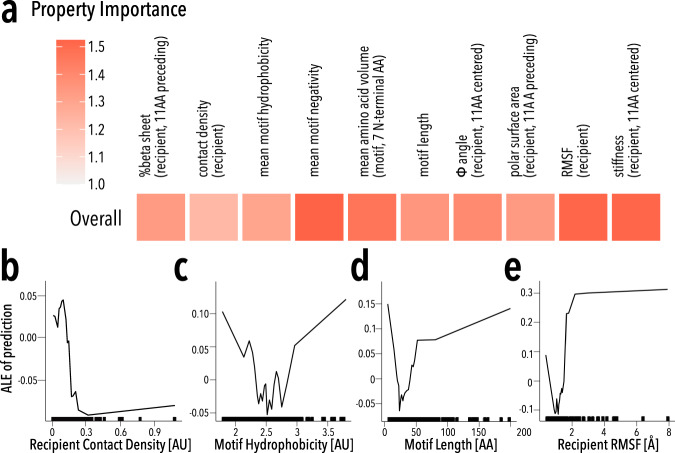
Machine learning model. **a** Heatmap of the recipient or donor property importance in predicting insertion fitness. Importance is based on the mean absolute error of removing features from the predictive model. **b**–**e** Plots of the Accumulated Local Effects (ALE) of properties on prediction insertion fitness for **b** recipient contact density, **c** motif hydrophobicity, **d** motif length, and **e** recipient RMSF. Marginal ticks indicate values that are present in the property data.

On the recipient side, local Kir2.1 flexibility (root mean squared fluctuations (RMSF) and stiffness) is important for model performance and is positively associated with insertion fitness (Fig. 5a, e, Supplementary Fig. 7c, g). Insertion position space (contact density) plays a major non-linear role (Fig. 5a, b). All recipient properties have monotonic relationships with insertional fitness, which suggests that insertion compatibility is predominantly determined by recipient properties (Supplementary Fig. 7).

**Fig. 6:**
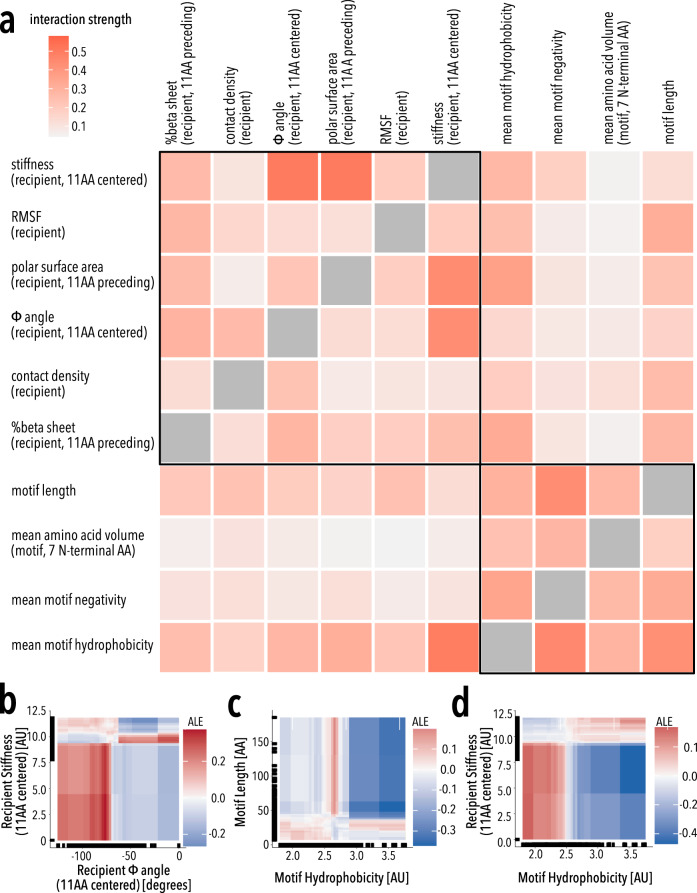
Property interactions. **a** Heatmap of pairwise property’s interaction strength. Boxes indicate interactions among properties of the recipient (top left) and motifs (lower right), respectively. **b**–**d** Pairwise ALE plots investigate how pairwise interactions contribute to the prediction of **b** recipient stiffness-recipient φ angle, **c** motif hydrophobicity-motif length, and **d** motif hydrophobicity-recipient stiffness. Marginal ticks indicate values that are present in the property data.

The most important motif properties are length, hydrophobicity, and negativity, all of which have non-linear relationships with insertion fitness (Fig. 5a, c–d, Supplementary Fig. 7). To understand why they are non-linear and how properties interact, we explored all property interactions within the model (Fig. 6). Recipient properties have relatively few interactions with other recipient properties (strength > 0.3 in ~17% of interactions), whereas motif properties strongly interact amongst themselves (strength > 0.3 in ~67% of interactions) and recipient properties (Fig. 6a). By exploring property interactions, we can begin to develop mechanistic explanations for insertion fitness patterns (Supplementary Discussion, Supplementary Figs. 8–11). For example, there is an enrichment for insertions in surface-exposed loops between beta-strands. Recipient-recipient interactions reveal a strong positive fitness in regions with low stiffness (high flexibility) and a φ angle between −150 to −75 degrees (corresponding to beta-sheets). A similar relationship also exists between insertion positions with low stiffness and high polar surface area. In both cases, these are consistent with insertions being allowed in flexible surface-exposed beta hairpins.

We can also explore why motif clusters behave distinctly (Supplementary Discussion, Supplementary Figs. 8–10). For example, longer motifs benefit from strong positive interactions between motif length and moderate hydrophobicity (Fig. 6c, Supplementary Fig. 8b). We interpret moderate mean hydrophobicity as the presence of a hydrophobic core in these motifs that can promote folding^76^. The formation of a stable hydrophobic core as a desirable property of engineered domains corroborates conclusions from high-throughput protein design experiments^49^. Too much or too little hydrophobicity in large motifs negatively impact insertion fitness probably because this either promotes aggregation or disorder, respectively. Interestingly, high contact density is somewhat beneficial for the largest structured motifs (Supplementary Fig. 8l). Highly hydrophobic donor motifs are deleterious within flexible regions (small flexible loops) of Kir2.1 likely because their solvent-exposed hydrophobic residues will be destabilizing and promote aggregation (Supplementary Fig. 10a, f)^77^. In contrast, being inserted into buried regions with high stiffness and contact density is beneficial for hydrophobic motifs because these insertion positions minimize solvent exposure (Supplementary Fig. 10a, c, f). Shorter unstructured motifs, which are less hydrophobic (Fig. 4d, Supplementary Fig. 9b) are deleterious in stiff, non-dynamic regions of Kir2.1 but beneficial in flexible, dynamic regions (Supplementary Fig. 9a, f, k). Stiff, non-dynamic regions are characterized by high contact density; the disruptive effect of inserting unstructured motifs we observe in the model likely is the result of important structural elements being disrupted (Supplementary Fig. 9l). The high compatibility of beta-sheet hairpin to accept short unstructured motifs (Fig. 2a) is captured by the model (Supplementary Fig. 9d). By investigating the interactions between properties in the model we can develop mechanistic explanations for the patterns seen in this rich dataset.

The ML model also allows us to propose a rudimentary framework of rules for successfully inserting donor motifs into recipient proteins:

- Insertion positions are ideally located in flexible protein regions with sufficient space.
- To form a well-folded domain, motifs need sufficient length and hydrophobic amino-acid content to form a well-ordered hydrophobic core.
- If the desired insertion position is located within a buried and rigid region, the inserted motif should be hydrophobic.
- More flexible regions prefer small non-hydrophobic insertions, and larger more structured domains will only be allowed if there is sufficient flexibility.
- Most significantly, the interactions between motifs and recipient properties determine the outcome of protein recombination.

### Distinct insertion fitness phenotypes classes are driven by a hierarchical organization of Kir2.1 that balances folding stability and dynamics required for gating

Motif and recipient property interactions produce distinct classes of motifs and regions (Fig. 2b, Fig. 4l). From correlation with biophysical properties, the ML model, and prior studies, we can develop an intuitive explanation for why we observe the segregation of distinct Kir2.1 regions into these classes. The first class represents protein regions that comprise TM and CTD core beta-sheets. Based on prior protein structure/function studies, we hypothesize that these regions require specific conformations to achieve a stable fold. This renders them very sensitive to insertions, in particular those that introduce disorder (e.g., unstructured motifs). The second class is comprised mostly of the N/C termini. The termini are not resolved in crystal structures of Kir, presumably because they are flexible. Flexibility suggests that they can adopt many folding-compatible conformations and they, therefore, allow most insertion types. The third class represents interfaces between TM and CTD, or between tetramer subunits. These contain many Kir2.1 regions that conformationally change upon PIP_2_ binding and during closed to open state transitions (PIP_2_ binding site, TM/CTD, and subunit interfaces)^65,78^. Since gating mechanisms are conserved across the inward rectifier family^79^, we hypothesize that the interface class may also be enriched for other inward rectifier regulators binding sites, such as Gβγ (GIRK), and ATP (Kir6.2). This is indeed the case (*p* value < 2e-16, two-sided Fisher’s Exact test, Fig. 3c).

Taken together, a steady-state biochemical assay that measures surface expression alone appears to map out the sensitivity of different regions of the channel to domain insertion. Sensitivity falls into distinct categories (class patterns) that are correlated with different structural and functional roles in Kir2.1. The contiguous nature of these class patterns suggests a hierarchical organization of inward rectifiers. Some regions are conformationally rigid to support folding and stability, while others are conformationally dynamic to enable gating and allosteric regulation. Considering what we know about how proteins work, this is an expected and obvious result. However, it points to the possibility that we can use domain insertion profiling—measuring how a protein’s phenotype changes upon systematic motif insertion—for annotating protein sequences with distinct roles in folding stability and conformational dynamics.

What is a possible mechanistic basis for detecting protein regions with distinct roles in folding stability and conformational dynamics through an assay that measures surface expression alone? It is plausible that the recipient protein will need to fold into an alternative conformation to adapt to a large topological insertion. However, this confirmation must still occupy allowable conformational states for successful folding, membrane insertion, and tetramerization. This implies that insertions are sampling an allowed conformational landscape, and with surface expression-based assays we are learning how tolerant a given insertion position is to large perturbation. From this perspective, it is intuitive how we could identify regions essential for folding—they will not allow any type of insertions. In contrast, for the regions not directly associated with folding and that can adopt a wide range of conformations, it is expected that any insertions type is allowed. The functional and regulatory regions we have identified with our assays are enriched for regions that undergo disorder/order transitions during channel gating or ligand binding. This means that they sample a limited ensemble of conformational states, which makes them more amenable to insertion compared to folding-critical sites, but less tolerant compared to non-critical sites. The metastable nature of these sites renders them sensitive to the specific degree of perturbation that different inserted motifs represent, which is why the intermediate and more insertion sensitive class overlaps well with regions involved in regulation and function.

### Insertional profiling is a generalizable method for coarse-grain annotation of ion channels

**Fig. 7:**
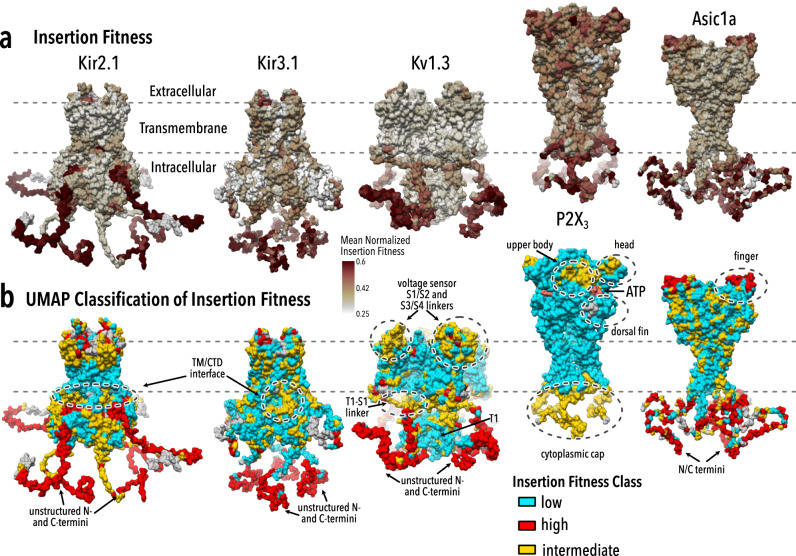
Generalization to other ion channels. Mean normalized insertion fitness (**a**) and UMAP insertion fitness clustering (**b**) mapped onto the crystal structures of Kir2.2 (PDB 3SPI^65^; 70% identity with Kir2.1), Kir3.2 (PDB 4KFM^79^; 45% identity with Kir3.1), Kv1.2/Kv2.1 paddle chimera (PDB 2R9R^80^, 62% identical with Kv1.3), P2X_3_ (PDB 5SVK^81^), and Asic1a (PDB 6AVE^82^). N- and C-terminal residues not resolved in crystal structures are modeled. For all channels apart from P2X_3_, low fitness (cyan) coincides with conformationally rigid and structured regions, while high fitness (red) coincides with highly flexible and unstructured regions. Intermediate fitness (yellow) is enriched in regions known to be dynamic and/or important for gating transitions (dashed circles). In P2X_3_, there are two regions that separate rigid transmembrane helices and ectodomain (class 1; cyan) and structured and dynamic regions (class 3; yellow). The ligand ATP is shown in soft red.

While it is clearly interesting that insertional profiling can probe Kir2.1’s backbone based on distinct structure-function roles, a question remains whether this applies to other ion channels. To test if our compatibility framework and the hierarchical organization generalizes, we profiled surface expression fitness in the inward rectifier Kir3.1 (GIRK), the voltage-dependent K^+^ channels Kv1.3, the purinoreceptor P2X_3_, and the acid-sensing channel Asic1a by inserting a smaller set of 15 motifs (Fig. 7a, Supplementary Table 5, Supplementary Fig. 12). Kir3.1 is a G-protein regulated paralog of Kir2.1 with a very similar structure^79^ but requires co-expression of Kir3.2 for effective trafficking^56^. Kv1.3, P2X_3_, and Asic1a have different folds, gating, and regulation^80–82^.

For related channels—Kir2.1 and Kir3.1—insertion profiles are correlated (Pearson correlation coefficient 0.56). Insertions in membrane-embedded regions are deleterious, insertions into termini are allowed, and different inserted motifs give rise to distinct fitness profiles (Supplementary Fig. 12). This suggests that biophysical properties and mechanisms that dictate fitness in Kir2.1 are generalizable to other ion channels that share the inward rectifier architecture. The general patterns of surface expression in inward rectifiers also apply to Kv1.3, P2X_3_, and Asic1a, however, the data sets are noisier likely due to reduced numbers of motifs and less efficient epitope labeling. There is a weak to moderate correlation between the relative impact of each domain (Supplementary Figs. 13 and14) in different channels. Although inserted motifs have similar effects across channels, in an assay that determines the fitness of recipient protein upon insertion, we would expect the recipient channel’s properties (i.e., the fold-specific difference in insertion sensitivity) to dominate.

Since properties manifested as distinct classes in Kir2.1, we wondered if this concept would also apply to Kir3.1, Kv1.3, and P2X3. Applying the same UMAP-based clustering approach used for Kir2.1, reveals discrete insertion fitness classes in all channels (Fig. 7b, Supplementary Fig. 2). As expected from shared fold architecture, Kir3.1’s classes resemble Kir2.1’s (Pearson’s *χ*^2^ test *p* value <2.2e-16, Cramer’s V 0.36) with three classes encompassing the TM and CTD core, regulator binding sites and interfaces, and termini. Using established structure/function data, we can infer those classes have distinct roles in folding stability and conformational dynamics. In each channel, there is a class that allows few insertions and corresponds to structural elements required for tetramerization (Kv1.3 T1 tetramerization domain), folding (inward rectifier CTD Ig-like fold^13^, P2X_3_ disulfide-stabilized ectodomain^81^, and Asic1a beta-sheets), or membrane insertion (transmembrane helices). All channels except for P2X3 have a class that allows nearly all insertions, and which coincides with flexible protein termini. The final class is intermediate, allowing only certain insertions. The intermediate class is enriched for residues that conformationally change during gating or regulation, for example, the Kir TM/CTD interface^65^, the Kv1.3 S1-T1 linker (based on homology of this region to Kv1.2^83^), and P2X_3_ cytoplasmic cap^81^. That we can identify distinct classes in all ion channel data sets with clear correspondence to different roles in folding and function suggest that insertional scanning is a generalizable method for annotation of ion channel structure.

## Discussion

We applied a high-throughput surface-expression assay that leverages flow cytometry and NextGen Sequencing to determine phenotypes of hundreds of thousands Kir2.1 insertional variants in a single experiment. This provides the necessary scale to comprehensively map genotype/phenotype relationships of systematic domain recombination. From this large-scale dataset, we explore the biophysical basis for domain compatibility, develop a basic framework of rules for protein engineering by domain insertion, and discover that insertional scanning can annotate structure/function relationships of a protein’s backbone.

The basic premise for our study is that systematic perturbation can provide insight into the general, fundamental properties of the Kir family. This is well supported by the literature (reviewed in ref. ^84^), which has shown that systematic single amino-acid mutations can reveal intrinsic properties of proteins^85^, determine protein structures^86^, and explain protein behavior in healthy and diseased cellular contexts^87^. Systematic domain insertion^27,33,88,89^ can help map different biophysical properties of proteins. Although motif insertions represent the more extreme end of a perturbation spectrum, they are highly relevant to fundamental protein evolution processes. Proteins evolve through (1) substitution, insertion, and deletions of amino acids or (2) large-scale recombination of proteins domains with discrete structures and functions^11^. The former predominantly fine-tune existing protein functions, the latter enables rapid acquisition of new protein functions. The exact combination of mechanisms that drive protein evolution through domain recombination is an open question, but it is generally accepted that they involve genomic mechanisms (e.g., exon joining) that bias domain gain to protein termini and adaptive dynamics that result from potential selective advantages conferred by domain gain^9,43,90^. Genomic events (recombination, retroposition) that result in a domain insertion into the middle of a protein are rare^7,67,91^. This means that the viability of domain gain at termini vs. domain insertion into the middle of a protein has not been sampled at the same rate during protein evolution. Studying the evolution of genome architecture may therefore be insufficient to detect additional mechanisms that constrain viable multidomain architectures. In this study, we are not restricted by genomic mechanisms to generate domain insertions; we can systematically probe the viability of inserting any domain into any position of a recipient gene with respect to folding and trafficking. Proper folding and trafficking are only two among likely many other properties (e.g., ion conductance) that are under selection pressure in the domain recombination scenario of ion channel evolution. However, they are absolutely required and therefore represent a hard constraint on allowable domain recombination space that evolution can explore. While for the moment limited to one ion channel, our data indicate that terminal gain is a generalizable feature across many different types of larger, structured domains. This provides strong support for a biophysical basis of domain compatibility in ion channels. Acquisition of this domain type at termini is tolerated because it is more likely to retain unperturbed folding of each domain that comprises a multidomain protein while still linking the functions of each domain to the same polypeptide chain. Compared to unstructured or hydrophobic motifs, gaining larger structured domains at the termini also avoids the potential aggregation of nascent polypeptide chains. It may even assist folding by nucleating a fast-folding stable subdomain that reduces topological frustration^73^ or increases folding cooperativity^68,69^. In this scenario, domain gain at termini would result in more stable ion channels, which could represent a selective advantage even in the absence of specific functional gains. How functionally gratuitous adaptation can become entrenched in protein evolution was recently demonstrated for the evolution of multimerization in protein complexes^92^. In this model, domain gains that initially only increase protein stability without adding new functions can serve as a foundation of latent functional capacity. Further adaptive changes can reveal this latent capacity and then give rise to functional divergence, for example, new protein/protein interactions or signaling behaviors^93^.

Beyond the evolution of protein recombination, our study provides an opportunity for improving engineering efforts. Using our experimental dataset and computed biophysical properties of the recipient protein and inserted motifs, we build a quantitative biophysical model of domain recombination in ion channels. Our discovery of specific interactions between donor and recipient properties is a crucial step towards universal domain recombination grammar^19^ for rational engineering of fusion proteins. The framework of rules we derived is very coarse and insufficient for practical use in designing synthetic proteins. Nevertheless, it is representative of fundamental mechanistic insight data-driven approaches to protein science can provide. More data, from across protein types and measured phenotypes (abundance, function, trafficking, etc.), and more sophisticated analytical models are needed to compile a comprehensive framework of design rules for algorithmic multidomain protein design. The need for such protein engineering frameworks will only grow in the future when combining protein domains and motifs into synthetic proteins with new functions (e.g., light- or drug switchable channels^26,60,94^) emerges as a strategy for observing and controlling molecular and cellular circuits. Successes notwithstanding, engineering synthetic multidomain proteins that fold and function well remains challenging. By moving away from trial-and-error towards computational models that predict viable combinations for given donor motifs and recipient proteins, we can accelerate this process. Beyond identifying compatible domain insertion sites, these models could be used to improve the recombinability of commonly used protein domain switches^23,34^ and sensors^95^ itself. A model of domain recombinability could also be incorporated in de novo protein design to expand its reach beyond well-folded isolated proteins. A toolbox of recombination-optimized protein domains would allow us to take the next step in synthetic protein engineering: standardized assembly of designed domains into useful multidomain protein tools and therapeutics.

Systematic mutation experiments such as Deep Mutational Scanning^84^ can reveal intrinsic properties of proteins^85^, determine protein structures^86^, and identify pathogenic mutations^87^. Circular permutation profiling has been used to study how protein topology and stability influences protein activity and mutational tolerance^96^. It is an open question whether insertional mutagenesis can reveal similar insight into protein features. Amino acid substitutions, which sample how a specific residue’s biochemistry contributes to phenotype, are often neutral because they do not affect a protein’s activity (catalysis, binding, etc.), folding, or conformational ensembles. Circular permutation alters protein topology and local chain entropy without affecting the overall structure. Insertions, on the other hand, represent more severe perturbations and will affect all these protein properties. Perhaps this is why insertions appear to sample more global properties of a protein backbone such as conformational plasticity. A comparison of single amino acid substitutions (i.e., Deep Mutational Scanning^84^) and motif insertion in the same assay is needed to establish a universal protein perturbation scale. A unified perturbation framework that ranges from amino-acid substitutions to large-scale topological changes would set the foundation for using perturbation scanning as a general approach for protein biochemistry and biophysics.

Insertional profiling provided insight into the general, fundamental properties of Kir2.1 and other ion channels. Unbiased clustering of insertion fitness reveals that insertion fitness phenotypes are driven by ion channel regions with different material properties (conformationally rigid, semi-flexible, flexible). We propose that class organization is a universal feature of ion channels that results from constraints on channel structure to satisfy folding, assembly, and interaction with trafficking partners while providing flexibility for allosteric regulation and conformational changes during channel opening and closing. Other studies proposed similar ideas of spatially contiguous protein regions linked to specific functions: protein sectors based on co-evolution across homologs^85,97–99^, mapping regions involved in folding stabilization vs. conformational flexibility by circular permutation profiling^96^, or revealing the functional architecture of enzymes from high-throughput enzyme variant kinetics^100^. Shared among these studies is the concept of a perturbation (naturally occurring or engineered mutations, insertions) probing the underlying biophysical properties that directly impact sequence-function relationships. Further experiments are required to establish whether the hierarchical organization of insertion fitness exists in all proteins. Should this be the case, insertional profiling could take a place within the larger spectrum of perturbation profiling as a universal high-throughput coarse-grain structural biology method for protein function, folding, and dynamics from steady-state biochemical experiments.

Between Deep Learning-based method (e.g., AlphaFold^101^) and atomic resolution cryo-electron microscopy protein structure determination is becoming less of a bottleneck in protein science. With an abundance of available structural information, the focus will shift towards the functional annotation of these structures. Insertional profiling could play a crucial role in solving this problem as a generalizable approach for annotating the functional capacity of different regions of a protein. Mapping the functional contribution of protein backbone components is crucial for understanding basic protein biology and developing small molecule drugs to treat disease.

## Methods

### Choice of domains

We curated 759 motifs a representative sample of biophysical properties that drive donor/recipient compatibility (Supplementary Table 1, see Supplementary Data 1 for oligonucleotide sequences). Common domains in extant proteins are selected from SMART domain groups, focusing on those with available structural information, and varying ranges of frequencies within the human genome^102^. The disordered protein fragments and proteins are from a curated disordered protein database, DISPROT^52^. While disordered fragments are derived from disordered regions within mostly structured proteins, disordered proteins are proteins that are entirely disordered. The manually curated motifs include natural, synthetic proteins, several switchable proteins, and a flexible glycine-serine-alanine-glycine (AGSAGSA) linker (Supplementary Table 5). The polypeptide linkers are manually selected hydrophobic and hydrophilic subsections from Kir2.1. Ancestral motifs have been proposed by Alva et al.^51^. The small non-domain proteins are manually selected monomeric small proteins which are not commonly recombined. The smotifs are super-secondary structural motifs that are common across proteins^50^. The natural proteins <50 AA acid motifs are a set of proteins under 50 amino acids that do not contain cysteines that were used in a massive protein stability assay^49^. Peptide toxins are a set of genetically encodable disulfide-rich neurotoxin peptides.

### Molecular biology

Genes encoding *mouse* Kir2.1 (Uniprot P35561), *mouse* Kir3.1 (Uniprot P63250), *mouse* Kir3.2 (Uniprot P48542), *human* Asic1a (Uniprot P78348), *human* P2X_3_ (Uniprot P56373), and *human* Kv1.3 (Uniprot P22001) were produced by DNA synthesis (Twist Bioscience). A Kozak sequence (GCCACC) and P2A-EGFP were added before and after each open reading frame, respectively. FLAG tag epitopes were added into previously described extracellular loops of Kir2.1 (between S116 and K117^59^), Kir3.1 (between K114 and A115^56^), Asic1a (between F147 and K148^103^), and P2X_3_ (between N72 and R73 based on insertion into the paralog P2X_2_^104^). Golden Gate compatible 5′ and 3′ sites were added to each gene by inverse PCR. Sequences of final constructs are in Supplementary Data 2.

### Library generation

We generated motif insertion libraries using Saturated Programmed Insertional Engineering (SPINE)^53^. In brief, we use multi-step Golden Gate cloning to insert a series of motifs in between all consecutive residue pairs of a gene. In silico, we break up a gene into fragments (~159 bp or 53 amino acids) with a genetic handle cassette inserted at every amino-acid position. See Supplementary Data 7 for oligonucleotide sequences: Cassette primers that encode insertional diversity, barcode primer to amplify subpool of cassette primers, and gene primers for inverse PCR of the backbone. The genetic handle has outward-facing BsaI type IIS restriction sites, which are subsequently replaced by an antibiotic cassette, chloramphenicol, to remove background wild-type DNA and to select for inserted library members. As the final step, the chloramphenicol cassette is replaced by the candidate motif flanked by short N-terminal Ser-Gly and C-terminal Gly-Ser linkers. As a quality control step, we sequence all our libraries for baseline coverage prior to screens (Supplementary Fig. 15).

### Cloning domains

The common domains, hand-curated motifs, and non-domain proteins were ordered as gene fragments (Twist Bioscience). The disordered, gene fragments, ancestral, structural, and motifs PDBs <50 amino acids were ordered in the form of an OLS pool (Agilent). See Supplementary Data 1 for nucleotide sequences. All motifs were mammalian codon-optimized and designed with amplifiable barcodes and BsaI type IIS restriction sites complementary to those in the inserted genetic handle. Golden gate cloning is conducted with BsaI-v2 HF (NEB), T4 Ligase (NEB) following the manufacturer’s instructions. Completed Golden Gate reactions were cleaned with Zymo Clean Concentrate kits and transformed into E. cloni™ electrocompetent cells (Lucigen). Diversity was maintained at every step such that there are at least 30× successfully transformed colony-forming units as determined by serial dilutions and plating an aliquot of liquid cultures.

### Library cell line construction

To generate cell lines, we used a rapid single-copy mammalian cell line generation pipeline^51^. In brief, insertion libraries are cloned into a staging plasmid with BxBI-compatible *attB* recombination sites using BsaI Golden Gate cloning. We amplify the staging plasmid backbone using inverse PCR and the library of interest with primers that add complementary BsaI cut sites. Golden Gate cloning is conducted with BsaI-v2 HF (NEB), T4 Ligase (NEB) following the manufacturer’s instructions. Completed Golden Gate reactions were cleaned with Zymo Clean Concentrate kits and transformed into E. cloni™ electrocompetent cells (Lucigen). Diversity was maintained at every step such that there are at least 30× successfully transformed colony-forming units as determined by serial dilutions and plating an aliquot of liquid cultures. Completed library landing pad constructs are co-transfected with a BxBI expression construct (pCAG-NLS-Bxb1) into (TetBxB1BFP-iCasp-Blast Clone 12 HEK293T cells). This cell line has a genetically integrated tetracycline induction cassette, followed by a BxBI recombination site, and split rapalog inducible dimerizable Casp9. Cell are maintained in D10 (DMEM, 10% fetal bovine serum (FBS), 1% sodium pyruvate, and 1% penicillin/streptomycin). Two days after transfection, doxycycline (2 μg/ml, Sigma-Aldrich) is added to induce expression of our genes of interest (successful recombination) or the iCasp-9 selection system (no recombination). Successful recombination shifts the iCasp-9 out of frame, thus only cells that have undergone recombination survive, while those that have not will die from iCasp-9-induced apoptosis. One day after doxycycline induction, AP1903 (10 nM, MedChemExpress) is added to cause dimerization of Casp9 and selectively kill cells without successful recombination. One day after AP1903-Casp9 selection, media is changed back to D10 + doxycycline (2 μg/ml, Sigma-Aldrich) for recovery. Two days after cells have recovered, cells are reseeded to enable normal cell growth. Once cells reach confluency, library cells are frozen in 50% FBS and 10% DMSO stocks in aliquots for assays.

### Sequencing-based surface expression assay

Thawed stocks of library cell lines were seeded a six-well dish and media was swapped the following day to D10. Cells were grown to confluency, split once to ensure maximum cell health, and then media was swapped to D10 + doxycycline (2 μg/ml, Sigma-Aldrich). Kir3.1 cannot homo-tetramerize and therefore requires a co-expressed Kir3.2 or Kir3.4 inward rectifier to surface express^56^. For this reason, 48 hours prior to sorting Kir3.1 libraries, we transiently transfected the stable Kir3.1 insertion library cell line with 2 μg Kir3.2-P2A-miRFP670 and 6 μl Turbofect per well of a six-well plate. For all libraries except for Kv1.3, we detached cells with 1 ml Accutase (Sigma-Aldrich), spun down and washed three times with FACS buffer (2% FBS, 0.1% NaN_3_, 1× PBS), incubated for 1-hour rocking at 4degC with a BV421 anti-flag antibody (Biolegend catalog# 637321) at 1:200 dilution, washed twice with FACS buffers, filtered with cell strainer 5 ml tubes (Falcon), covered with aluminum foil, and kept on ice for transfer to the flow cytometry core. For Kv1.3, cells were detached and washed the same except after initial washing cells were brought up in FACS buffer with Agitoxin-2-Cys-TAMRA (5 nM, Alomone), filtered with cell strainer 5 ml tubes, and brought to cell sorting facility on ice. Before sorting, 5% of cells were saved as a control sample for sequencing prior to sorting.

All cells were sorted on a BD FACSAria II P69500132 cell sorter. Flow data was collected using FACSDiva version 8.0.1 and analyzed using FlowJo 10.

EGFP fluorescence was excited with a 488 nm laser and recorded with a 525/50 nm bandpass filter and 505 nm long-pass filter. BV421 fluorescence was excited using a 405 nm laser and recorded with a 450/50 nm bandpass filter, TAMRA fluorescence was excited using a 561 nm laser and recorded with a 586/15 nm bandpass filter, and miRFP670 was excited with a 640 nm laser and recorded with 670/30 nm bandpass filter. All cells (except those expressing Kir3.1) were gated on forwarding scattering area and side scattering area to find whole cells, forward scattering width, and height to separate single cells, EGFP for cells that expressed variants without errors (our library generation results in single base pair deletions that will not have EGFP expression because deletions will shift EGFP out of frame^53^), and label (either Agitoxin-Cys-TAMRA for Kv1.3, BV421 for all others) for surface-expressed cells. Kir3.1 library cells were gated on forwarding scattering area and side scattering area to find whole cells, forward scattering width, and height to separate single cells, miRFP670 for Kir3.2 co-expression, GFP for cells that expressed variants without errors, and label (BV421) for surface-expressed cells. The surface expression label gate boundaries were determined based on unlabeled cells from the same population because controls tend to have non-representative distributions. Examples of the gating strategy for each channel are depicted in Supplementary figures 16–20.

EGFP^high^/label^low^ (cells expressing non-trafficking variants) and EGFP^high^/label^high^ cells (expressing surface-trafficked variants) were collected into catch buffer (20% FBS, 0.1% NaN_3_, 1× PBS). For larger pooled sublibrary samples, we collected between at least 100,000 to 500,00 cells per gate which is ~8–35× coverage. 15,000 cells in both gates of a Kir2.1 library with a small flexible AGSAGSA linker was collected each day to normalize all the pooled libraries. For smaller 15 motifs samples, we collected between 4000–50,000 of each sample/library pair which is ~10–120× coverage for all libraries. Note that theoretical coverage given above assumes that all positions are present in each cell collection, which will underestimate coverage. In practice, the more disruptive an insertion the fewer surface-labeled cells can be collected. However, those cells that are collected will be enriched for the few positions in which this disruptive variant is allowed. This means that fewer collected cells sample that smaller set of compatible insertion positions, which in turn result in sufficient coverage.

The entire process was repeated to collect two independent replicates.

### Sequencing

For both biological replicates, DNA from pre-sort control and sorted cells was extracted with Microprep DNA kits (Zymo Research) and triple-eluted with water. The elute was diluted such that no more than 1.5 μg of DNA was used per PCR reaction and amplified for 20 cycles of PCR using Primestar GXL (Takara Clonetech), run on a 1% agarose gel, and gel purified. Primers that bind outside the recombination site ensure leftover plasmid DNA from the original cell line construction step is not amplified. Purified DNA was quantified using Picogreen DNA quantification. Equal amounts (by mass) of each domain insertion sample were pooled by cell sorting category (“pre-sort control”, “surface expression”, “no surface expression”). For Kir2.1, pools were further divided to segregate highly similar motifs sequences. Pooled amplicons were prepared for sequencing using the Nextera XT sample preparation workflow and sequenced using Illumina Novaseq in 2x150bp mode. Read count statistics and coverage of mapped reads are in Supplementary Table 6. Source sequencing data is available in the Sequence Raw Archive (SRA- https://www.ncbi.nlm.nih.gov/sra) and the accession codes for the data are: PRJNA766040 (Project_047) and PRJNA766074 (Project_045).

### Enrichment calculations

Forward and reverse reads were aligned individually using a DIPseq pipeline^32^, slightly modified for SPINE compatibility and for updated python packages. If both forward and reverse reads report an insertion, duplicated domain insertion calls are removed to avoid artificially boosting counts. This pipeline results in.csv spreadsheets indicating insertion position, direction, and whether it is in frame.

Surface-expression enrichment was calculated by comparing the change in EGFP^high^/label^low^ to EGFP^high^/label^high^. Enrichment calculation was based on Enrich2^105^ and implemented in R version 4.1.0. Only positions with reads in both label^low^ and label^high^ groups were used in enrichment calculations. For each cell group, the percentage of reads at each position was calculated after adding 0.5 to assist positions with very small counts. Enrichment was calculated by taking the natural logarithm of EGFP^high^/label^high^ percentage divided by the EGFP^high^/label^low^ percentage for each position (*i*), inserted motif (*m*), and replicate (*r*).

1 $${{{{{\rm{Enrichment}}}}}}_{i,m,r}^{{{{{\rm{raw}}}}}}={{{{{\rm{ln}}}}}}\frac{0.5+{{{{{{\rm{Count}}}}}}{{{\mbox{\_}}}}{{{{{\rm{High}}}}}}}_{i,m,r}}{{\sum }_{i}^{n}0.5+{{{{{{\rm{Count}}}}}}{{{\mbox{\_}}}}{{{{{\rm{High}}}}}}}_{i,m,r}}/\frac{0.5+{{{{{{\rm{Count}}}}}}{{{\mbox{\_}}}}{{{{{\rm{Low}}}}}}}_{i,m,r}}{{\sum }_{i}^{n}0.5+{{{{{{\rm{Count}}}}}}{{{\mbox{\_}}}}{{{{{\rm{Low}}}}}}}_{i,m,r}}$$

All datasets were *z* scored to an internal control flexible linker motif (AGSAGSA) enrichment (separate for each sequencing subpool) by subtracting the average control motif enrichment (*μ*_flex linker_) and dividing by the standard deviation of the control motif enrichment (*σ*_flex linker_).

2 $${{{{{\rm{Enrichment}}}}}}_{i,m,r}^{{{{{{\rm{z}}}}}}\;{{{{{\rm{scored}}}}}}}=\frac{{{{{{\rm{Enrichment}}}}}}_{i,m,r}^{{{{{\rm{raw}}}}}}-{\mu }_{{flex}\;{{{{{\rm{linker}}}}}}}}{{\sigma }_{{flex}\;{{{{{\rm{linker}}}}}}}}$$

Replicates (*r*) were combined by a weighted average, which was calculated by a restricted maximum likelihood estimate (*M*) and standard error (SE) using 50 Fisher scoring iterations.

3 $${{{{{\rm{Enrichment}}}}}}_{i,m}^{{{{{{\rm{z}}}}}}\;{{{{{\rm{scored}}}}}}}={\sum }_{r}^{n}{{{{{\rm{Enrichment}}}}}}_{i,m,r}^{{{{{{\rm{z}}}}}}\;{{{{{\rm{scored}}}}}}}* \frac{\sqrt{{M}_{m,r}+{{{{{{\rm{SE}}}}}}_{m,r}}^{2}}}{{\sum }_{r}^{n}\sqrt{{M}_{m,r}+{{{{{{\rm{SE}}}}}}_{m,r}}^{2}}}$$

Standard error was calculated assuming a Poisson distribution.

4 $${{{{{\rm{SE}}}}}}_{m,r}=\sqrt{\frac{1}{{{{{{{\rm{Count}}}}}}{{{\mbox{\_}}}}{{{{{\rm{High}}}}}}}_{i,m,r}+0.5}+\frac{1}{{{{{{{\rm{Count}}}}}}{{{\mbox{\_}}}}{{{{{\rm{Low}}}}}}}_{i,m,r}+0.5}+\frac{1}{{\sum }_{i}^{n}{{{{{{\rm{Count}}}}}}{{{\mbox{\_}}}}{{{{{\rm{High}}}}}}}_{i,m,r}+0.5}+\frac{1}{{\sum }_{i}^{n}{{{{{{\rm{Count}}}}}}{{{\mbox{\_}}}}{{{{{\rm{Low}}}}}}}_{i,m,r}+0.5}}$$

All other positions are treated as NA and are not considered in further analysis (exclusion criteria) except for correlations between datasets as removing data adds more noise than treating NAs as 0 s due to sampling.

### Data quality

Inserting 759 motifs into 435 Kir2.1 positions yields a total theoretical library diversity of 327,888 variants. Each sub-pooled library we generated and screened encompassed 12,500 variants. Owing to random variance, some data sets were incomplete (Fig. 1b–e). To make downstream analysis more robust, we only included motifs with data (after exclusion criteria outlined in *Enrichment Calculations)* in >80% of positions. This left us with 637 out of 759 motifs (further details in Supplementary Table 1).

### Clustering

All motif insertional profiling data was clustered by calculating a cosine distance matrix and clustering it with Ward’s hierarchical clustering method using the hclust function in R (version 4.1.0) with the “ward.D2” method. UMAP-based clustering was done using the uwot R package (version 0.1.10) using cosine or Euclidean distance metrics, and a local neighborhood size of 10 sample points. Neighborhood size influences how UMAP balances local versus global structure in the data. Within a range of neighborhood sizes tested (2–50), our choice best conveys the broader structure of the data.

### Ensemble network model

To calculate the dynamics of the recipient and motifs with available PDBs, we used the Prody Python package^106^ and code from Golinski et al.^107^ as a starting point kindly provided by Alexander Golinski and Benjamin Hackel (University of Minnesota). We calculated the mean stiffness of each backbone based on weighted sums of normal modes from an Anisotropic Network Model of vibration. We calculated summed recipient stiffness for varying lengths (1, 3, 5, 7, 9, 11 amino acids) before, centered on, and after an insertion position. Motif stiffness was summed for the entire motif and for varying lengths of the N- and C-termini (1, 2, 3, 4, 5, and 6 amino acids).

### Molecular dynamics simulations

All-atom force-field-based molecular dynamics simulations were carried out to sample multi-μs trajectories. Our structural models (PIP_2_-bound PDB 3SPI and apo state PDB 3JYC^65^) are constituted by the channel embedded in a bilayer of ~1300 POPC lipids hydrated by two slabs containing ~170,000 waters and ~600 KCl ion pairs, for a total of ~700,000 atoms. We first generated the coordinates of the missing amino acids in the experimental structures (mostly located in unstructured regions) using ROSETTA (for this purpose we generated 10,000 models and kept the representative structure of the most populated cluster). We then used charmm-gui^108^ to model the bilayer and the aqueous compartment. Simulations are being performed with the charmm36 force-field^109^ at a temperature of *T* = 303.15 K, using the highly parallel computational code NAMD2.12^110^ on 280 processors cores from Temple University’s Owlsnest. Per-RMSF were calculated by considering the position of the C_α_ atoms of each residue using the R bio3D package^111^.

### Structure mapping

Calculated properties (e.g., fitness) were mapped onto atomic ion channel structures using Chimera version 1.16 (build 42330)^112^. Missing loops were manually built using Pymol as poly-alanine chains.

### Amino-acid scoring

We calculated bioinformatic scores for amino acids using Quantiprot (version 0.2.4) written for Python 2.7.16^113^. For scores we used: molecular weight, surface area, alpha-helical propensity, beta-sheet propensity, buried accessibility ratio propensity, flexibility, hydropathy, hydrophobicity, negative charge, pKa, polarity, positive charge, reverse turn propensity, and volume. These scores were calculated for both recipients and donors. We calculated summed recipient scores for varying lengths before, centered on, and after an insertion position (1, 3, 5, 7, 9, 11 amino acids). Motif sequence scores were summed for the entire motif and for varying lengths of the N and C termini (1, 2, 3, 4, 5, and 6 amino acids). Motif length was also included.

### Protein structural properties

A series of properties were calculated with heavily modified code kindly provided by Alexander Golinski and Benjamin Hackel^107^ that uses Pymol called from python scripts (Python version 2.7.16). Recipient protein PDBs were trimmed of any ions, water, and other non-protein atoms. Recipient protein phi, psi, contact degree, contact order, long contact degree, secondary structure percentage, alpha-helical percentage, beta-sheet percentage, nonpolar solvent accessible surface area (SASA), charged SASA, and hydrophobic SASA. For each of these properties, we summed recipient structural scores for varying lengths (1, 3, 5, 7, 9, 11 amino acids) before, centered on, and after an insertion position. For motifs with structures, the mean phi angle, mean psi angle, the radius of gyration, the distance between N- and C-termini, the distance of N- and C-termini to the center of mass, motif size in Daltons, mean contact degree, mean contact order, mean long contact degree, mean secondary structure percentage, mean alpha-helical percentage, mean beta-sheet percentage, mean nonpolar SASA, mean charged SASA, mean hydrophobic SASA, and RMSD (in case of multiple conformers) were calculated. In addition to mean motif structural properties, N- and C-terminal varying lengths (1, 2, 3, 4, 5, and 6 amino acids) sums were calculated for the phi angle, psi angle, contact degree, contact order, long contact degree, secondary structure percentage, alpha-helical percentage, beta-sheet percentage, nonpolar SASA, charged SASA, hydrophobic SASA, and RMSD. Note that we switched from contact degree to contact density in the final construction of Random Forests. Contact density was calculated using the Arpeggio webserver^114^, which counts the number of interatomic contacts in Kir2.1 (PDB 3SPI) based on SMARTS atom-typing and distance/angle-based contact definitions (CREDO^115,116^).

### Choosing features to train Random Forest

To allow for greater interpretability of our Random Forest-based models, we filtered the input features for redundancy. Our approach to reducing property redundancy was as follows: for motifs, we took the shortest and longest N- and C-terminal features as well as the mean motif features. We identified redundant motif properties by setting a ±0.8 correlation cutoff calculated between the motif property and permissibility across all motifs for a given site. We chose the most explanatory of highly correlated motif properties based on summed absolute correlative value across all positions. For recipient properties, we took the longest and shortest of each mean property before, centered, and after the insertion position. We identified redundant recipient properties by setting a ±0.8 correlation cutoff calculated between the recipient property and permissibility across all positions for a given motif. We chose the most explanatory of highly correlated recipient properties based on summed absolute correlative value across all motifs. These steps reduced our properties from 908 (520 recipient and 388 motif) down to 64 (32 recipients and 32 motifs) properties.

### Random Forests

Once we had a non-redundant set of 64 properties, we trained a preliminary random forest model with 500 trees (Supplementary Fig. 5). Based on this preliminary model, we further trimmed the properties down to the most explanatory 20 (12 recipient and 8 motif properties). We retrained the model without a significant drop in model performance (39.98% variance explained for 69 properties and 39.44% for 18 properties, Supplementary Table 4). However, at this point, we were including motif structural properties. This meant that we were not able to include any motifs without structural data. As only 1 of the top 10 most predictive properties (“Motif Phi Mean” as the 9th most predictive) were from the structured domain set, we decided to exclude structure-based motif features altogether. This allowed us to include more motifs and reduce our non-redundant properties set down further (38.69% for 10 properties, Supplementary Table 4). We ended up choosing the top 10 most predictive features which included 6 recipient features (stiffness, phi angle of 11 AA centered around insertion site, MD simulation RMSF, contact density at insertion site, polar surface area of 11 AA preceding insertion site, beta-sheet content in 11 AA preceding insertion site) and 4 motif features (mean hydrophobicity, motif length, mean negative charge, mean amino-acid volume of seven N-terminal residues). This final model was trained using 85% of the data, with the other 15% withheld for testing, and performed well on the test dataset (Supplementary Fig. 6). All random forests were trained using the Randomforest package in R (version 4.6-14) with 500 trees and localimp = “TRUE” with all model parameters set to default values.

### Reporting summary

Further information on experimental design is available in the Nature Research Reporting Summary linked to this paper.

## Supplementary information

Supplementary Information

Description of Additional Supplementary Files

Supplementary Data 1

Supplementary Data 2

Supplementary Data 3

Supplementary Data 4

Supplementary Data 5

Supplementary Data 6

Supplementary Data 7

Reporting summary

## Data Availability

Sequencing data generated in this study have been deposited in the Sequence Raw Archive (https://www.ncbi.nlm.nih.gov/sra) under accession codes PRJNA766040 (Project_047) and PRJNA766074 (Project_045); refer to Supplementary Table 6 for corresponding metadata. Raw data are provided with this paper: Processed data (*z* scored surface trafficking scores) are available as Supplementary Data 3. Calculated inserted motif and recipient protein properties are available as Supplementary Data 4 and 5. Additional raw data (machine learning model, raw data of manuscript figures) are deposited on Zenodo (10.5281/zenodo.5683566). All structural models are available at the Protein Data Bank (https://www.rcsb.org) under accession codes 3SPI (PIP_2_-bound Kir2.2), 3JYC (apo state Kir2.2), 4KFM (Kir3.2), 2R9R (Kv1.2/Kv2.1 paddle chimera), 5SVK (P2X3), and 6AVE (Asic1a). Primary sequences of all channels used in this study are available at https://www.uniprot.org under accession codes P35561 (*mouse* Kir2.1), P63250 (*mouse* Kir3.1), P48542 (*mouse* Kir3.2), P78348 (*human* Asic1a), P56373 (*human* P2X3), P22001 (*human* Kv1.3). Supplementary Data 1 and 2 contain inserted domain and target channel sequences, respectively. Together these are the minimal set of data required to replicate the analysis. All data are available without restriction.

## Code availability

The code for handling data from domain insertion library sequencing is available at: https://github.com/SavageLab/dipseq. The SPINE code is available at: https://github.com/schmidt-lab/spine. The version used for this study is archived at https://zenodo.org/badge/latestdoi/223953195. The Enrich2 that was adapted for data analysis is available at: https://github.com/FowlerLab/Enrich2. Quantiprot is available at: https://pypi.org/project/quantiprot/. Prody is available at: http://prody.csb.pitt.edu. A R Markdown document to reproduce manuscript figures is provided as Supplementary Data 6.
