# Supplementary material for: Probing ion channel functional architecture and domain recombination compatibility by massively parallel domain insertion profiling
Source: Nat Commun. 2021 Dec 8;12:7114. doi: 10.1038/s41467-021-27342-0 (PMC8654947; doi:10.1038/s41467-021-27342-0)
Supplement: Supplementary file 1 — Supplementary Information [file 41467_2021_27342_MOESM1_ESM.pdf]

SUPPLEMENTARY INFORMATION FOR:

Probing Ion Channel Functional Architecture and Domain Recombination Compatibility by Massively Parallel Domain Insertion Profiling

Willow Coyote-Maestas[1], David Nedrud[1], Antonio Suma[2], Yungui He[3], Kenneth A. Matreyek[4], Douglas M. Fowler[5,6], Vincenzo Carnevale[2], Chad L. Myers[7], Daniel Schmidt[3]

[1]Department of Biochemistry, Molecular Biology & Biophysics, University of Minnesota, Minneapolis, MN, 55455, USA

[2] Department of Chemistry, Temple University, Philadelphia, PA, 19122, USA

[3] Department of Genetics, Cell Biology & Development, University of Minnesota, Minneapolis, MN, 55455, USA

[4] Department of Pathology, Case Western Reserve University School of Medicine, Cleveland, OH, 44106, USA

[5] Department of Genome Sciences, University of Washington, Seattle, WA, 98115, USA

[6] Department of Bioengineering, University of Washington, Seattle, WA, 98115, USA

[7] Department of Computer Science and Engineering, University of Minnesota, Minneapolis, MN, 55455, USA

*To whom correspondence should be addressed: schmida@umn.edu

This PDF file includes:

Supplementary Discussion

Supplementary References

Supplementary Figures 1 – 20

Supplementary Tables 1 – 7

Supplementary Discussion: Detailed rules for protein recombination from machine learning.

Properties that guide recombination: Random Forest models allow us to study how a set of properties interact non-linearly to give rise to a phenotype. We trained a random forest model on a set of recipient and motif properties to learn what determines productive protein motif insertions into our recipient protein Kir2.1. We calculate feature importance for every property by looking at how model performance is impacted when a given property is not included in the model. We find the most important property overall is motif hydrophobicity, with recipient flexibility (stiffness and RMSF), motif length, and recipient phi angle around an insertion site close behind. The most important motif properties are the motifs length and hydrophobicity, and the most important recipient properties are contact density and stiffness. However, based on feature importance alone, we do not know how properties relate to insertions.

We can further investigate how properties give rise to productive insertions through accumulated local effects (ALE) plots (Figure 5b-e, Supplementary Fig. 7). These plots summarize the local effects of a property on the model's prediction. For example, flexibility appears to have switch-like interactions whereby, below a threshold rigidity, it is quite deleterious (Fig. 5e, Supplementary Fig. 7c Positive relationship in RMSF and Supplementary Fig. 7g negative relationship in stiffness). Other recipient properties also have straightforward positive or negative monotonic relationships such as polar solvent accessible surface area (SASA) (negative, Supplementary Fig. 6j), beta sheet % (positive, Supplementary Fig. 7f), Phi angle (positive, Supplementary Fig. 7d), and Contact density (Fig. 5b, Supplementary Fig. 7e). Overall, recipient features appear to determine insertional fitness in relatively simple monotonic ways. For example, positive correlation with flexibility and beta sheets 11 amino acids prior to an insertion position are positive, which likely means flexible beta hairpin loops are desirable insertion positions. This result is in line with previous insertion strategies (see Dagliyan, O. et al. Engineering extrinsic disorder to control protein activity in living cells. *Science* 354, 1441-1444 (2016)).

In contrast, all motif properties have more complex relationships to insertional fitness. For example, lower motifs hydrophobicity appears to be deleterious (1.8-2.5) then becomes beneficial at higher values. Similarly, motif length is negative until it becomes beneficial in the model at about 25 amino acids. This is true for the other motif features as well: motif negativity

(Supplementary Fig. 7b) is initially negative (albeit noisy) then becomes positive. N-terminal 7 amino acid volume (Supplementary Fig. 7i) that is initially positive, becomes negative, and returns to be positive. Overall, this suggests motif properties have more complex relationships to insertional fitness. Motif properties are beneficial in some contexts and deleterious in others.

Taken together, recipient properties behave as expected in which flexible loops appear to be beneficial. In contrast to existing approaches to engineer synthetic fusion protein (e.g., [26]) that consider inserts to be interchangeable and solely focus on the properties of insertion positions, we propose that inclusion of motifs properties and their interactions is crucial to understand whether an insertion is viable at a given insertion position.

Interactions between properties: Random forests are comprised of many decision trees built from random subsets of features that in aggregate predict a desired outcome from properties. Decision trees make predictions by splitting a dataset at property thresholds set on each input feature. Thresholds on multiple input features enable decision trees, and by extension forests, to capture non-linear interactions between properties if they are predictive of the class being modeled. These non-linear interactions are why a property such as motif length can be positive and negative in different contexts.

To identify which properties are interacting with which, we calculated pairwise interaction strengths between all properties (Fig. 6a). The strongest interactions overall in-order of strength are pairwise interactions between recipient stiffness with motif hydrophobicity, phi angle, and polar solvent accessible surface area. Close behind are motif hydrophobicity with length and negativity. Overall, there are many pairwise interactions between all the motif features and fewer interactions between motif and recipient properties. Recipient contact density weakly interacts with motif hydrophobicity and length. There are also moderate pairwise interactions between recipient contact density with motif hydrophobicity and length. Overall, this means that motif properties interact with each other to determine how a motif behaves when inserted into a position and secondarily with recipient properties to determine whether a motif feature set is beneficial.

To learn which interactions are driving insertional fitness, we calculated and plotted pairwise ALE. It is important to note that pairwise ALE only represents the interaction that contributes to insertional fitness and does not consider how either property contributes alone.

For recipient properties there are strong interactions between stiffness, phi angle, and polar surface area (Supplementary Fig. 11).

When looking at the strongest interaction overall, motif hydrophobicity and recipient stiffness it is apparent that very high hydrophobicity is extremely deleterious within very flexible regions, low hydrophobicity is very beneficial in flexible regions, and high hydrophobicity is moderately beneficial in stiff (likely buried) regions (Fig. 6d). Observing non-linear interactions help us build hypotheses of underlying biophysical mechanisms, such as hydrophobic residues when exposed and inserted into flexible surface exposed regions are extremely deleterious, whereas when these same motifs are inserted into buried likely more hydrophobic regions these become beneficial. In addition, interactions between motif length (> ~25 AA) and stiffness demonstrate a different trend, where long insertions into very flexible regions are deleterious (these are regions at the termini of the structure likely needed for folding and small flexible loops) and very rigid regions are also deleterious for long motifs (Supplementary. Fig. 8i). Longer motifs are, however, beneficial in intermediate flexibility regions which are regions within the structured C-terminal domains that move (e.g., flexible loops and the PIP_2 binding sites). By comparing these two pairwise ALEs (Fig. 6d Stiffness-Hydrophobicity and Supplementary. Fig. 9i/ Supplementary. Fig. 10i Stiffness-Length), we can see that short non-hydrophobics are most preferred within very flexible regions, short hydrophobics are most preferred within very flexible regions, and longer partially hydrophobic motifs are preferred in semi-flexible regions. Furthermore, hydrophobicity is deleterious for short motifs, beneficial for longer motifs, and extremely deleterious for short motifs (Fig. 6c). Perhaps in longer motifs, hydrophobic residues provide stabilization by virtue of well-formed hydrophobic cores, whereas shorter motifs lack well-formed hydrophobic cores and instead expose hydrophobic residues thus becoming very disruptive by promoting aggregation. Overall, this analysis points to motif hydrophobicity and length interacting to determine how a motif behaves within the context of a recipient property. These interactions give rise to the classes of motifs and regions, we observe in clustering (Fig. 2a, Fig. 3a-b).

We can also investigate the few strong interactions between recipient properties to learn what features contribute to whether an insertion can occur at all at a given site. Recipient stiffness and phi angle strongly interact with a phi angle between -125 and -75 and a low stiffness being strongly beneficial (Supplementary Fig. 11a). This interaction strongly points towards flexible loops at beta

turns being ideal insertion positions. The other strong recipient-recipient interaction is between stiffness and polar solvent accessible surface area (Supplementary Fig. 11b). Highly polar surface exposed residues that are flexible allow many insertions which is intuitive because this also lines up with flexible loops. However, quite interesting is the strong beneficial interaction between very stiff and non-surface exposed or polar residues. This likely represents buried and stiff residues which with hydrophobic motifs could be beneficial (Supplementary Fig. 10a). Overall, recipient-recipient interactions point strongly to flexible loops between beta sheets as being ideal insertion positions. A question remains whether this is generally true across different backbones, which will need to be tested in future experiments.

To further investigate what drove specific motif cluster behavior, we calculated and annotated ALE plots based on where motif class properties are located (Fig. 4c-e).

Larger structured motifs: Larger more structured motifs contain nearly all folded proteins and are most interesting from an engineering perspective. This class is overwhelmingly determined by length, with hydrophobicity being intermediate and negativity only slightly higher than other groups. While the overall class does appear to be driven by length, length interacts strongly with hydrophobicity and weakly with negativity (Supplementary Fig. 8b, j). Hydrophobicity is positive for long motifs likely representing the ability to form a hydrophobic core and fold. This interaction becomes even more clear when focusing on a subset of motifs within this class that are commonly recombined domains and other well folded larger proteins. There is a clear demarcation above which hydrophobicity is highly beneficial (Supplementary Fig. 8b), which is likely why folded proteins has such a tight band of hydrophobicity (Fig. 4g). There is a similarly tight distribution of negativity and may be an impact, but it is not nearly as strong (Supplementary Fig. 8h, j). Large motifs in very flexible (and generally small loops) are deleterious but intermediate stiff regions are more amenable to larger insertions (Supplementary Fig. 8i). Paradoxically, high contact density appears beneficial for insertion of large motifs (Supplementary Fig. 8l). Insertions of long motifs appear very deleterious in beta sheet rich regions, which likely disrupt formation of the immunoglobulin-like C-terminal domain of Kir2.1 (Supplementary Fig. 8d). Overall, motif length and hydrophobicity strongly interact positively to give rise to increased insertional fitness likely through improving folding. Whether this is beneficial is dependent on where an insertion occurs. Regions with some flexibility and sufficient space are deleterious. However, if there is sufficient space (N -and C-termini and βD-βE loop) insertions are quite beneficial. To better design domains

for recombination, it would be ideal to have stable domains that have sufficient size and hydrophobicity to be able to maintain their fold after recombination, otherwise their folding thermodynamics will likely be overruled by the recipient protein.

Unstructured short cluster behavior: For the short unstructured motifs, non-hydrophobicity and length are important within unstructured regions because these regions prefer polar hydrophilic motifs as these will be solvent exposed (Supplementary Fig. 9a,c,i). These motifs however are not allowed well in buried regions based on high contacts being deleterious for small motifs (Supplementary Fig. 9l). In general negativity appears to play a weak negative role (Supplementary Fig. 9h, j). Finally, there is a strong beneficial interaction in regions with beta sheets in the 11 amino acids preceding – perhaps implying flexible beta hairpin loops (Supplementary Fig. 9d). Flexible motifs are overwhelmingly inserted within flexible loops or at the termini of beta sheets (Fig. 2a). This class is primarily best allowed within flexible and non-buried regions. Motifs fall into this class if they are non-hydrophobic and small, which means they will be non-disruptive from the perspective of space (contact density), flexibility (stiffness), and surface exposure (beta sheet %).

Hydrophobic motifs: For the hydrophobic motifs, it is quite clear that hydrophobicity drives the behavior of this class. The motif length is not as important because hydrophobic motifs range in size. Hydrophobic motifs mostly benefit from little negativity (Supplementary Fig. 10h, j), which makes sense as many hydrophobic motifs are best allowed with small segments of the transmembrane M1 and negativity would be disruptive when interacting with lipids. Hydrophobic motifs are very deleterious when inserted within very flexible regions and beneficial within rigid regions (Supplementary Fig. 10a). This combined with highly hydrophobic motifs being beneficial within high contact regions (Supplementary Fig. 10c) means hydrophobics are beneficial when inserted within buried regions. Hydrophobics are highly deleterious in and around beta sheets (Supplementary Fig. 10d). Overall, this means hydrophobics behave inversely to the unstructured short cluster. Hydrophobics are mostly deleterious but can be inserted in some buried and transmembrane regions where they will not be disruptive. That said, several recipient flexible loops can accept either motif class (βC-βD, βE-βG, βH-βI, βL-βM). Interestingly, the βD-βE loop and unstructured termini that strongly allows and prefers longer more structured motifs does not allow for most hydrophobic inserts, perhaps because hydrophobics would interact with the solvent to cause misfolding and aggregation.

SUPPLEMENTARY REFERENCES

1. Letunic, I. & Bork, P. 20 years of the SMART protein domain annotation resource. *Nucleic Acids Res* **46**, D493-D496 (2018).
2. Sickmeier, M. et al. DisProt: the Database of Disordered Proteins. *Nucleic Acids Res* **35**, D786-93 (2007).
3. Alva, V., Söding, J. & Lupas, A. N. A vocabulary of ancient peptides at the origin of folded proteins. *Elife* **4**, e09410 (2015).
4. Pugalenthi, G., Suganthan, P. N., Sowdhamini, R. & Chakrabarti, S. SMotif: a server for structural motifs in proteins. *Bioinformatics* **23**, 637-638 (2007).
5. Rocklin, G. J. et al. Global analysis of protein folding using massively parallel design, synthesis, and testing. *Science* **357**, 168-175 (2017).
6. Golinski, A. W., Holec, P. V., Mischler, K. M. & Hackel, B. J. Biophysical Characterization Platform Informs Protein Scaffold Evolvability. *ACS Comb Sci* **21**, 323-335 (2019).
7. Bakan, A., Meireles, L. M. & Bahar, I. ProDy: protein dynamics inferred from theory and experiments. *Bioinformatics* **27**, 1575-1577 (2011).
8. Konopka, B. M., Marciniak, M. & Dyrka, W. Quantiprot - a Python package for quantitative analysis of protein sequences. *BMC Bioinformatics* **18**, 339 (2017).
9. Doyle, D. A. et al. Crystal structures of a complexed and peptide-free membrane protein-binding domain: molecular basis of peptide recognition by PDZ. *Cell* **85**, 1067-1076 (1996).
10. Taslimi, A. et al. Optimized second-generation CRY2-CIB dimerizers and photoactivatable Cre recombinase. *Nat Chem Biol* **12**, 425-430 (2016).
11. Iwakura, M. & Nakamura, T. Effects of the length of a glycine linker connecting the N-and C-termini of a circularly permuted dihydrofolate reductase. *Protein Eng* **11**, 707-713 (1998).
12. Iwamoto, M., Björklund, T., Lundberg, C., Kirik, D. & Wandless, T. J. A general chemical method to regulate protein stability in the mammalian central nervous system. *Chem Biol* **17**, 981-988 (2010).
13. He, Y., Chen, Y., Alexander, P. A., Bryan, P. N. & Orban, J. Mutational tipping points for switching protein folds and functions. *Structure* **20**, 283-291 (2012).
14. Bhardwaj, G. et al. Accurate de novo design of hyperstable constrained peptides. *Nature* **538**, 329-335 (2016).

15. Dagliyan, O. et al. Rational design of a ligand-controlled protein conformational switch. *Proc Natl Acad Sci U S A* **110**, 6800-6804 (2013).
16. Halavaty, A. S. & Moffat, K. N- and C-terminal flanking regions modulate light-induced signal transduction in the LOV2 domain of the blue light sensor phototropin 1 from Avena sativa. *Biochemistry* **46**, 14001-14009 (2007).
17. Pazgier, M. et al. Structural basis for high-affinity peptide inhibition of p53 interactions with MDM2 and MDMX. *Proc Natl Acad Sci U S A* **106**, 4665-4670 (2009).
18. Kuhlman, B. et al. Design of a novel globular protein fold with atomic-level accuracy. *Science* **302**, 1364-1368 (2003).
19. Marcos, E. et al. Principles for designing proteins with cavities formed by curved β sheets. *Science* **355**, 201-206 (2017).
20. Marcos, E. et al. De novo design of a non-local β-sheet protein with high stability and accuracy. *Nat Struct Mol Biol* **25**, 1028-1034 (2018).

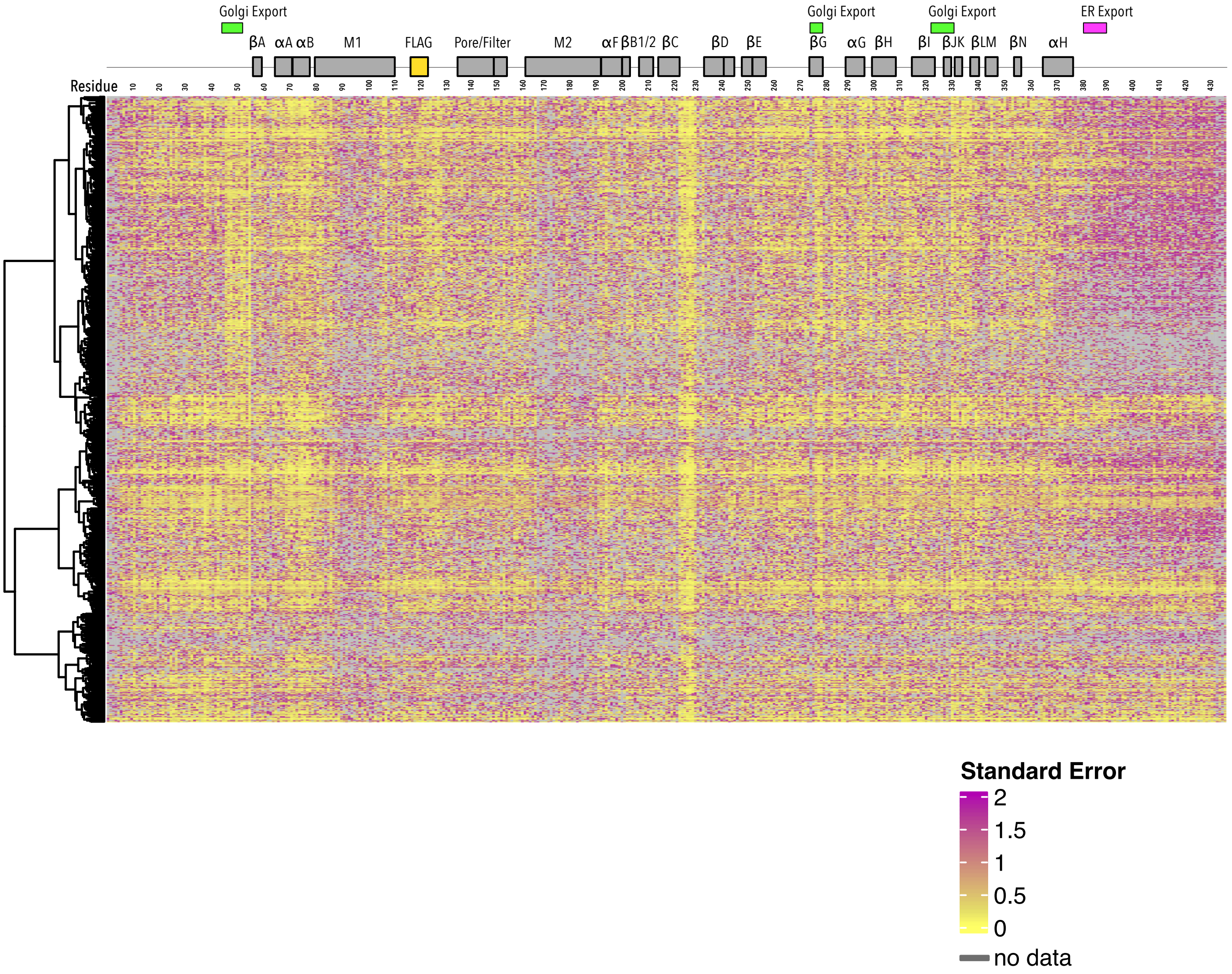

Supplementary Figure 1: Fitness Standard Error. Heatmap of Poisson Standard Error (see *Methods*) for 759 motifs inserted into all positions of Kir2.1. Grey indicates no data. Secondary structural elements (grey boxes) are Kir2.1 are shown above, along known Golgi and ER export signals (green and magenta boxes, respectively). Motifs are hierarchically clustered using a cosine distance metric (same as in Figure 2a).

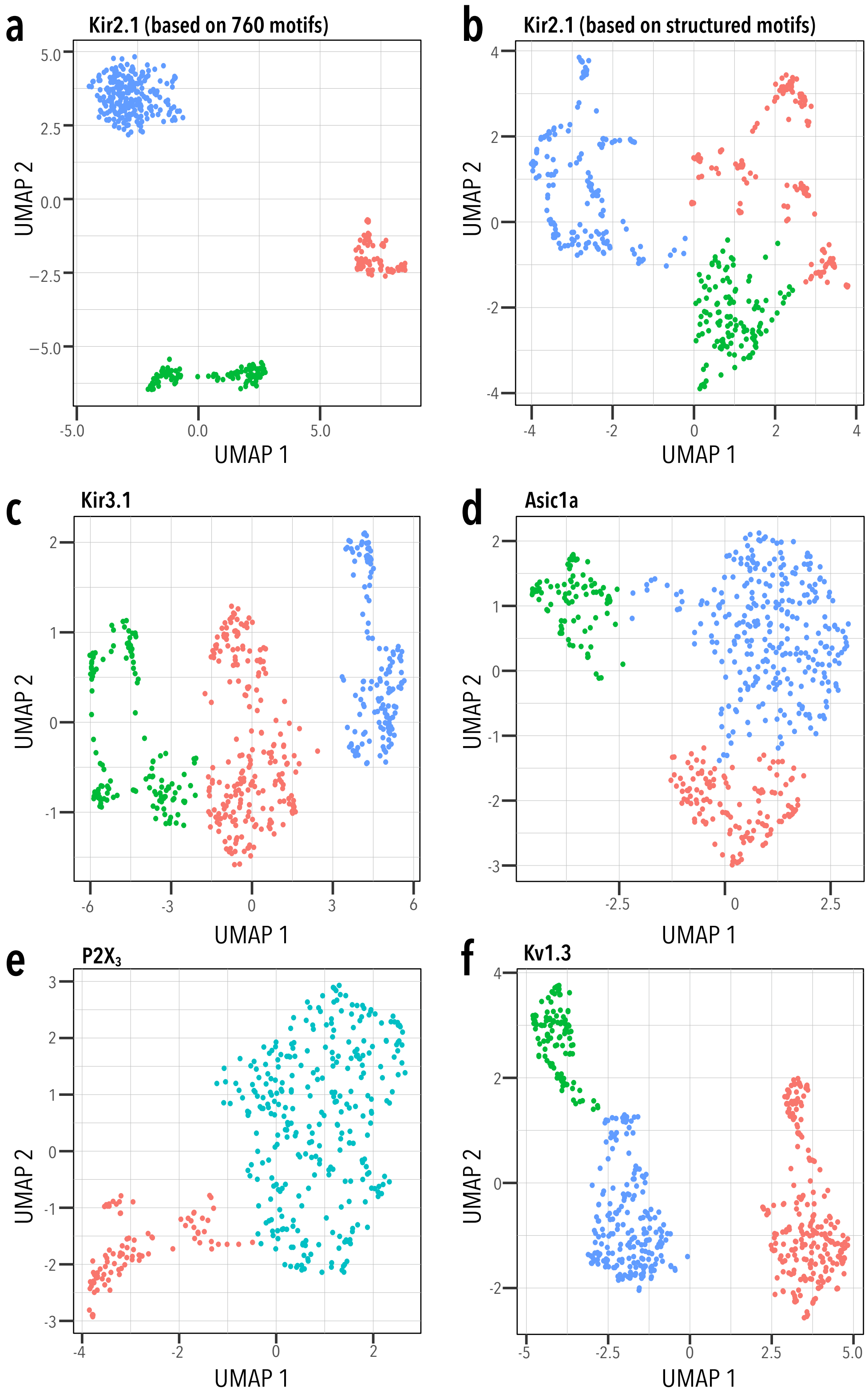

Supplementary Figure 2: Unbiased clustering of insertion fitness. Uniform Manifold Approximation Projection (UMAP) was used to cluster insertion fitness of each channel. Cluster membership of each residue is indicated by color. Optimal cluster number was determined using the R package Nbclust using the majority rule.

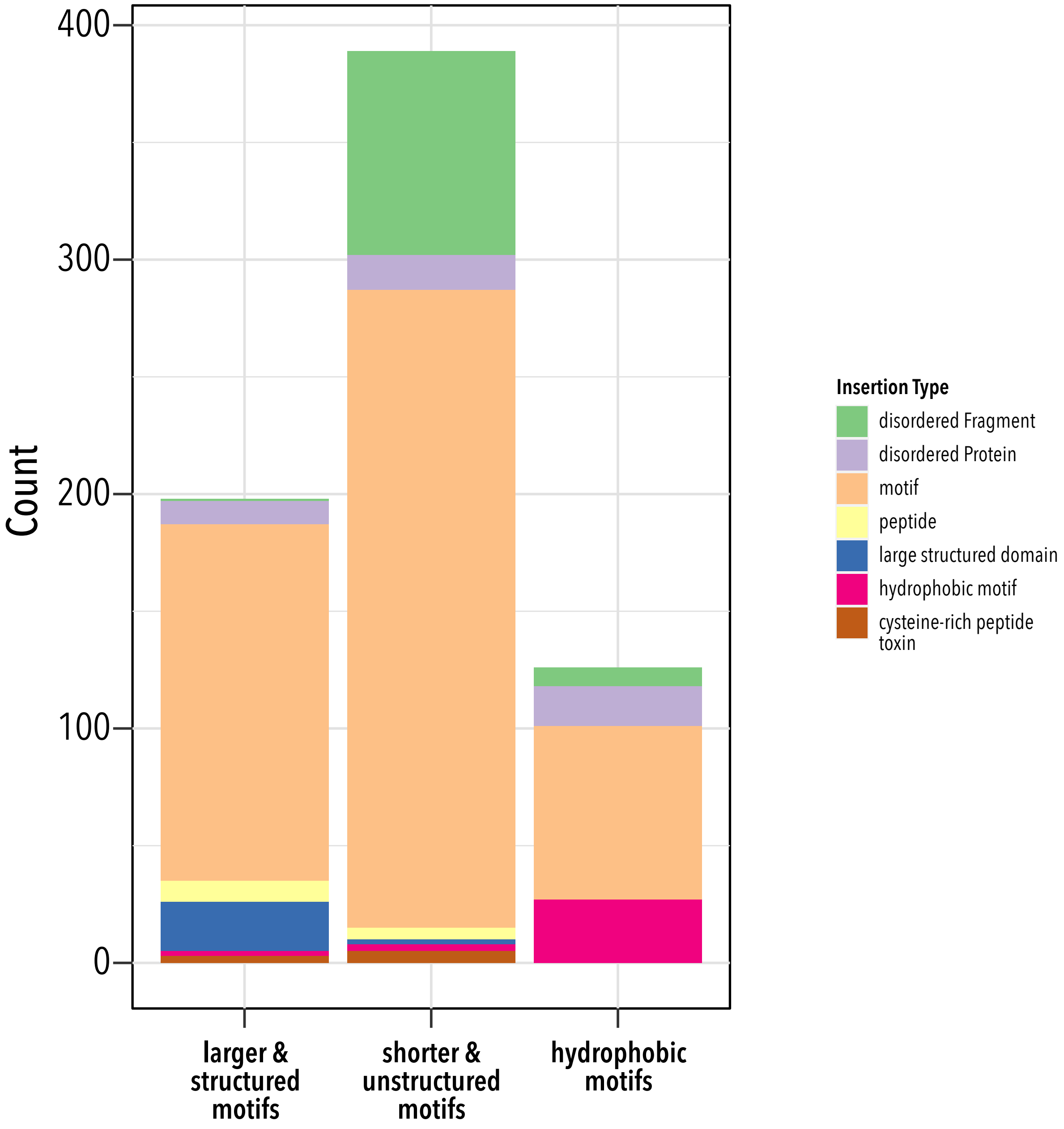

Supplementary Figure 3: Hierarchical clustering of insertion fitness by motif resulted in three major groups (dendrograms of Fig. 2a & Supplementary Fig. 1). Stacked barplot illustrates enrichment of these three groups for different insertion motif types.

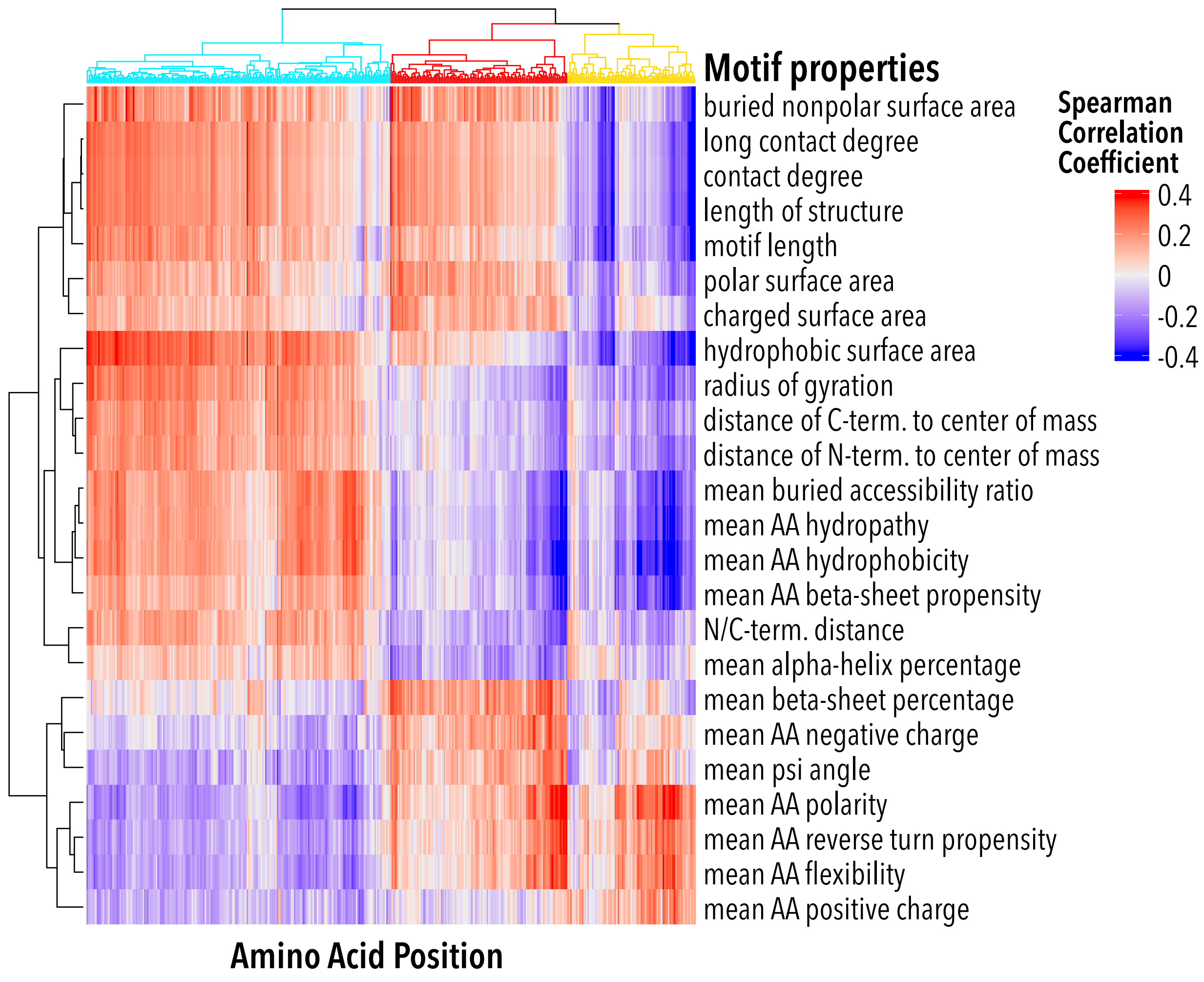

Supplementary Figure 4: Clustered positions and properties correlation plot. Correlation plot between motif properties and the fitness of that motif at each position. The motif properties and positions are hierarchically clustered. Position clusters dendrogram branches are colored (cyan, red, yellow) as in **Fig. 4I**.

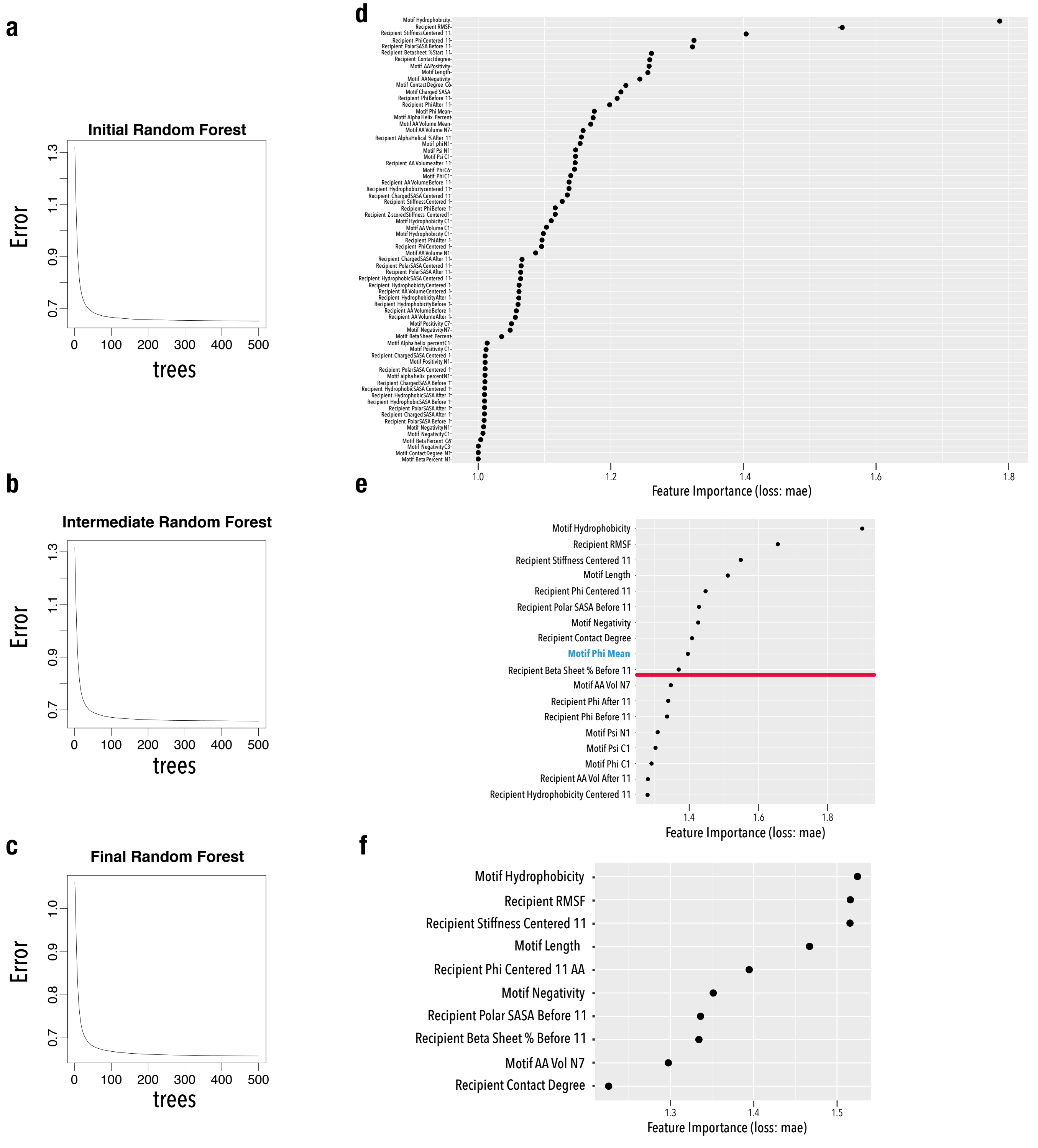

Supplementary Figure 5: Random Forest model iteration training and property importance. a-c Error curves with mean squared error plotted against number of trees in the **(a)** initial, (**b**) intermediate, and (**c**) final Random Forest models. As more trees are added, there is less error. **d-f** Bar plots of the importance of features in predicting insertional fitness in the (**d**) initial, (**e**) intermediate, and (**f**) final Random Forest models. The red line in (**e**) indicated the threshold that was used to trim features. In addition, mean motif phi angle (blue) was removed because it required motifs to have solved structures, which substantially limited the number of motifs we could include. Property importance is based on the mean absolute error (mae) of removing properties from the predictive model. Further details can be found in the *Methods*.

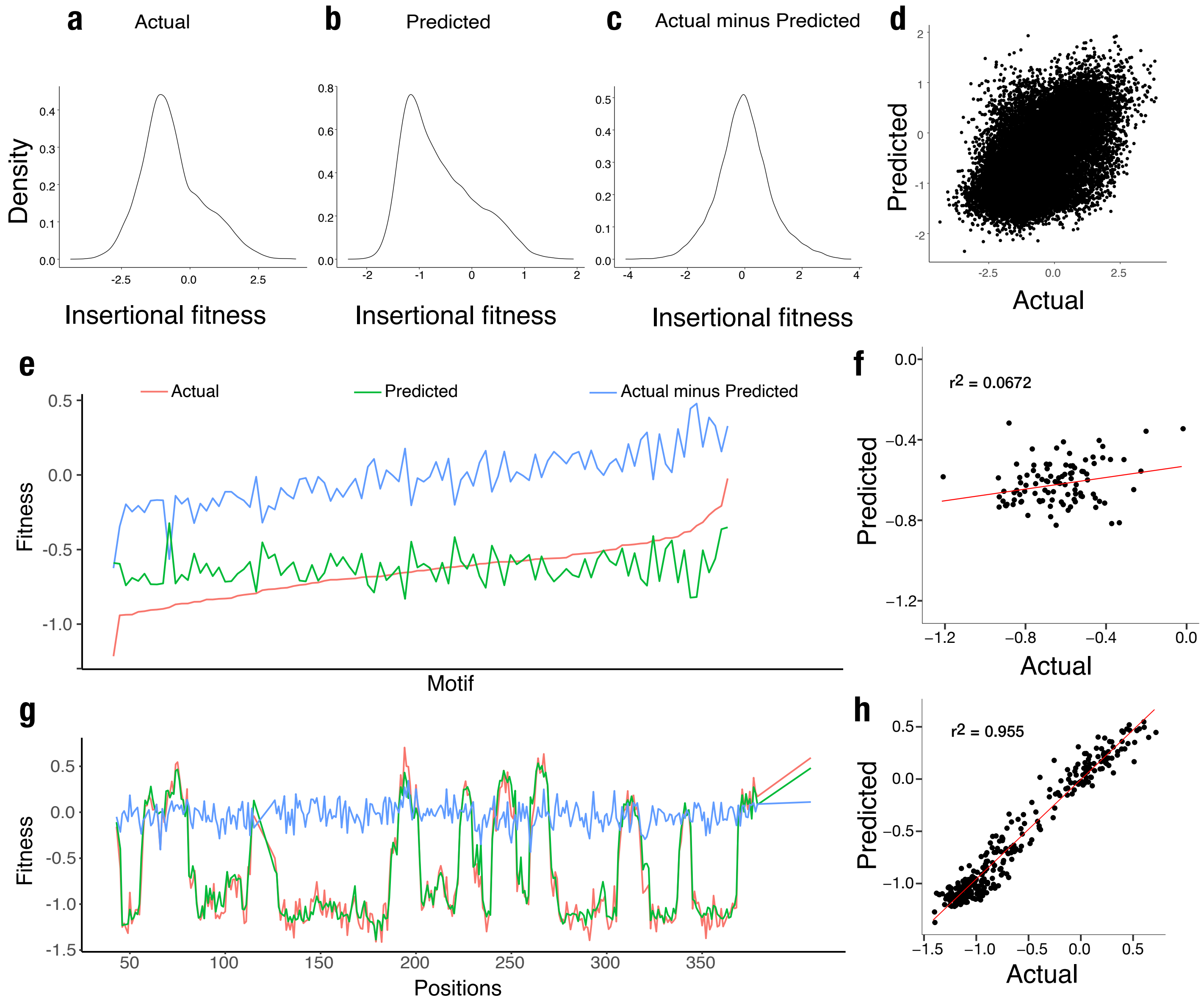

Supplementary Figure 6: Model performance plots. a-c Density plots for (**a**) actual, (**b**) predicted, and (**c**) difference between actual and predicted insertional fitness. (**d**) Scatterplot of actual vs. predicted fitness. **e** Actual Insertional fitness (ordered by lowest to highest actual fitness), predicted fitness, and the difference averaged over all positions per motif. **f** Scatterplot of actual vs. predicted fitness averaged over all position per motif. **g** Actual Insertional fitness, predicted fitness, and difference averaged over all motifs per recipient insertion position. **h** Scatterplot of actual vs. predicted fitness averaged over all positions per motif. All model performance is reported based on data withheld from all Random Forest training.

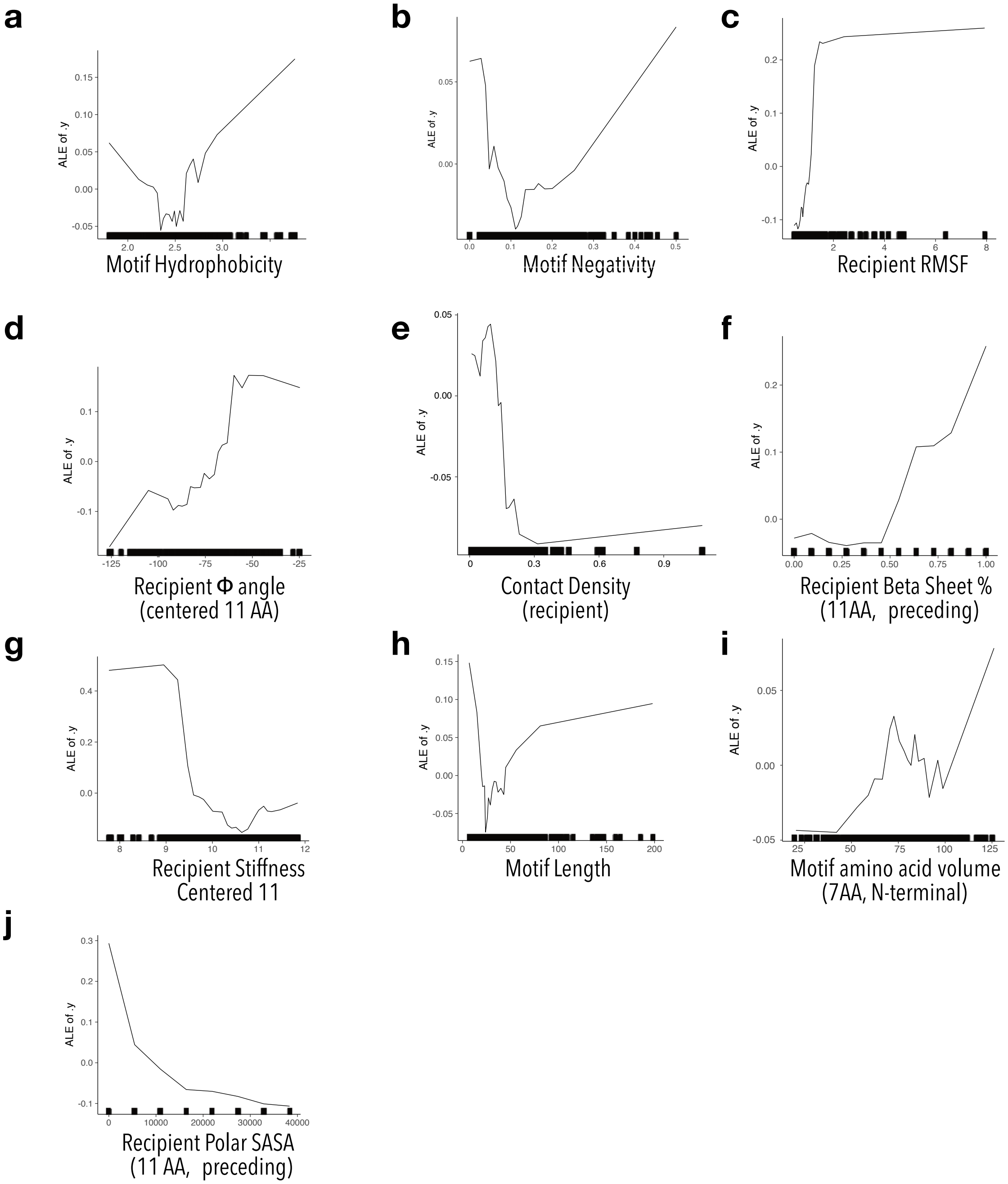

Supplementary Figure 7: ALE plots for final model properties. Plots of the Accumulated Local Effects (ALE) of properties on the prediction of insertional fitness for (**a**) mean motif hydrophobicity, (**b**) mean motif negativity, (**c**) recipient root mean square fluctuation (based on MD simulation, PDB code 3JYC), (**d**) mean recipient phi angles of 11 AA centered around insertion site, (**e**) recipient contact density, (**f**) mean beta sheet content 11 AA before insertion site (**g**) mean recipient stiffness of 11 AA centered around insertion site, (**h**) motif length, (**i**) mean amino acid volume of the motif's 7 N terminal AA, and (**j**) polar surface accessible surface area of 11 AA before insertion site.

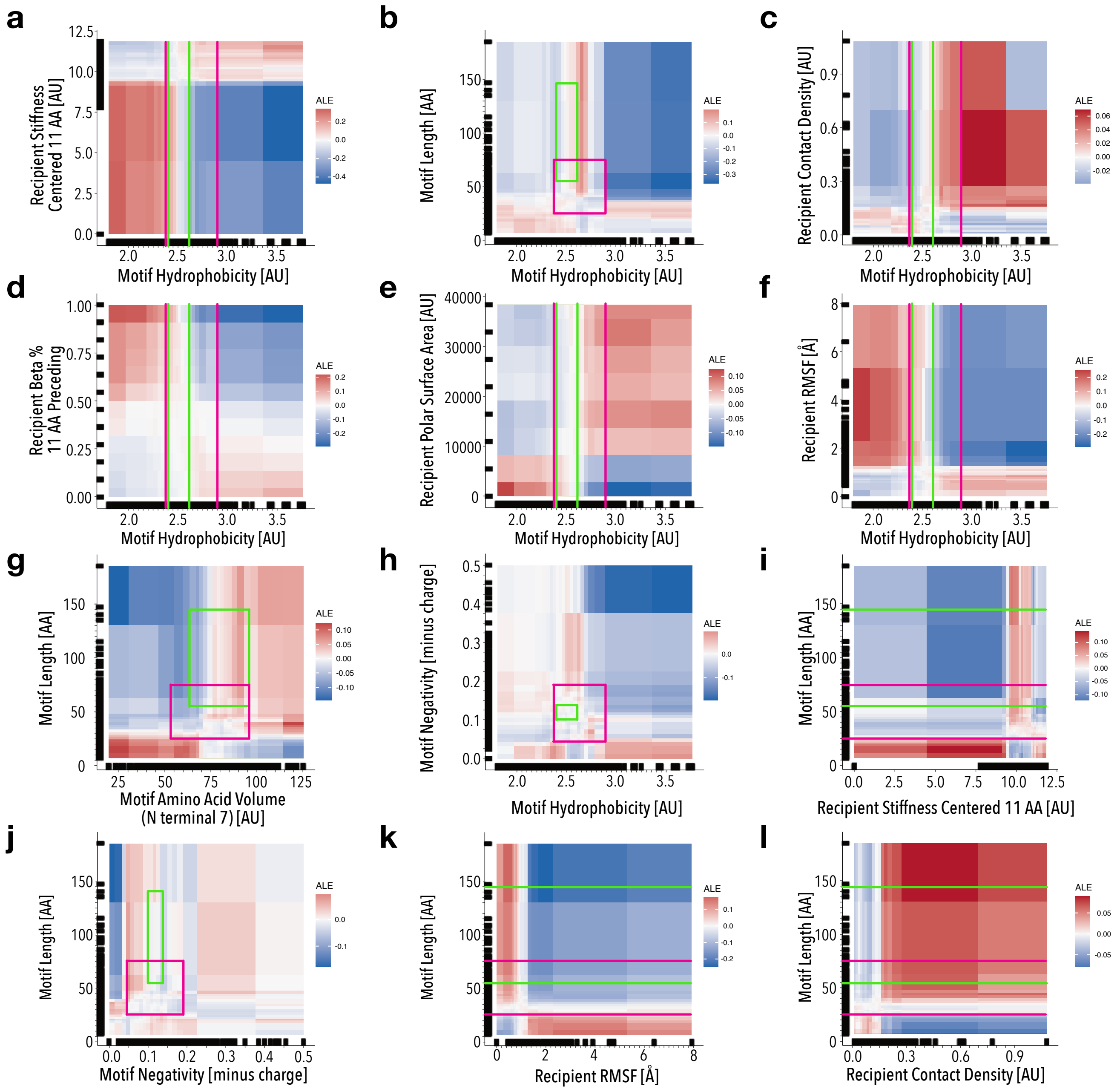

Supplementary Figure 8: Larger & Structured motif cluster pairwise ALE exploration. Pairwise ALE plots investigate how pairwise interactions contribute to prediction (quantified by ALE scores). The distributions (10th-90th percentiles) of larger & structured motifs (see **Figure 2a**) are marked by magenta lines or boxes (green for subset of frequently recombined domains; see Supplementary Table 1). Marginal ticks indicate data point used in model building.

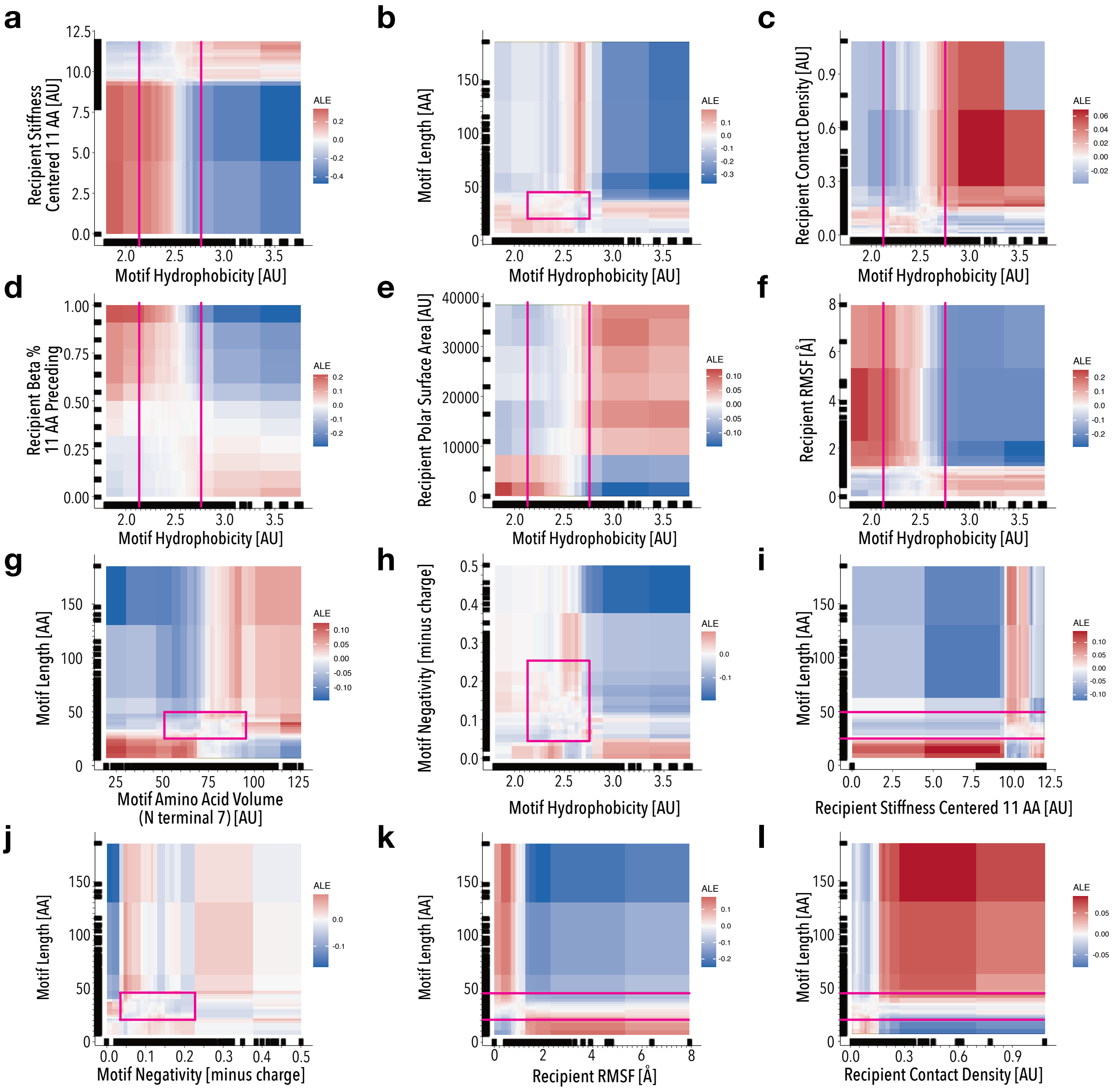

Supplementary Figure 9: Shorter & Unstructured motif cluster pairwise ALE exploration. Pairwise ALE plots investigate how pairwise interactions contribute to prediction (quantified by ALE scores). The distributions (10th-90th percentiles) of unstructured motifs (see **Figure 2a**) are marked by magenta lines or boxes. Marginal ticks indicate data point used in model building.

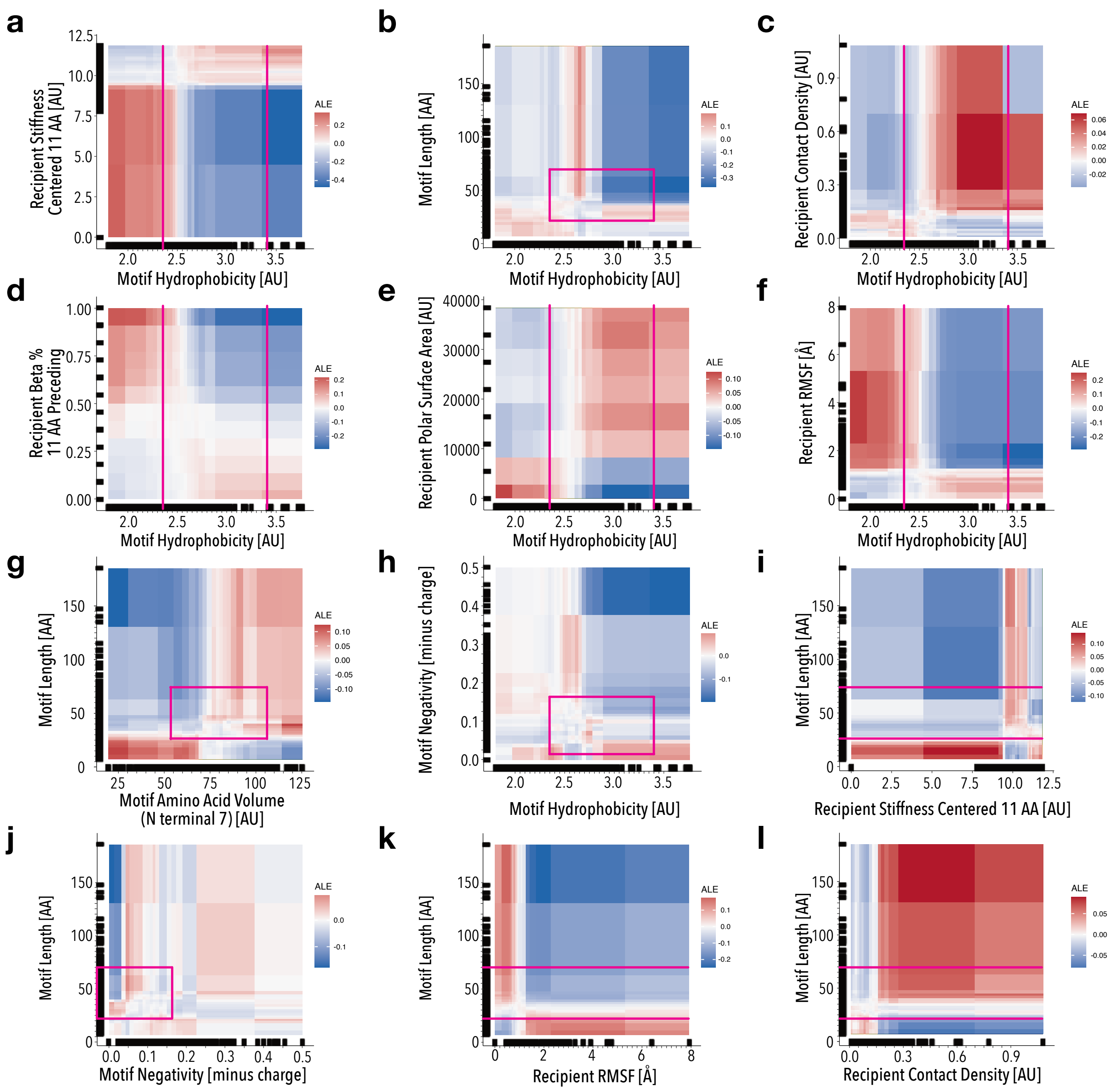

Supplementary Figure 10: Hydrophobic motif cluster pairwise ALE exploration. Pairwise ALE plots investigate how pairwise interactions contribute to prediction (quantified by ALE scores). The distributions (10th-90th percentiles) of hydrophobic motifs (see **Figure 2a**) are marked by magenta lines or boxes. Marginal ticks indicate data point used in model building.

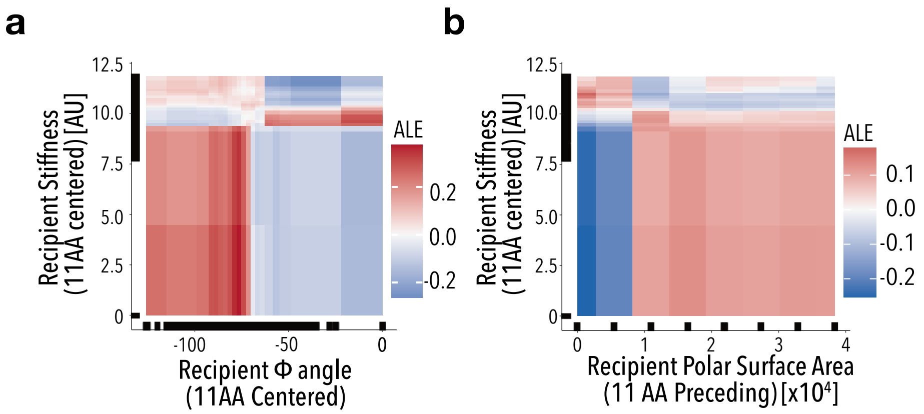

Supplementary Figure 11: Recipient property interactions. Pairwise ALE plots investigate how pairwise interactions contribute to prediction (quantified by ALE scores). Marginal ticks indicate data point used in model building.

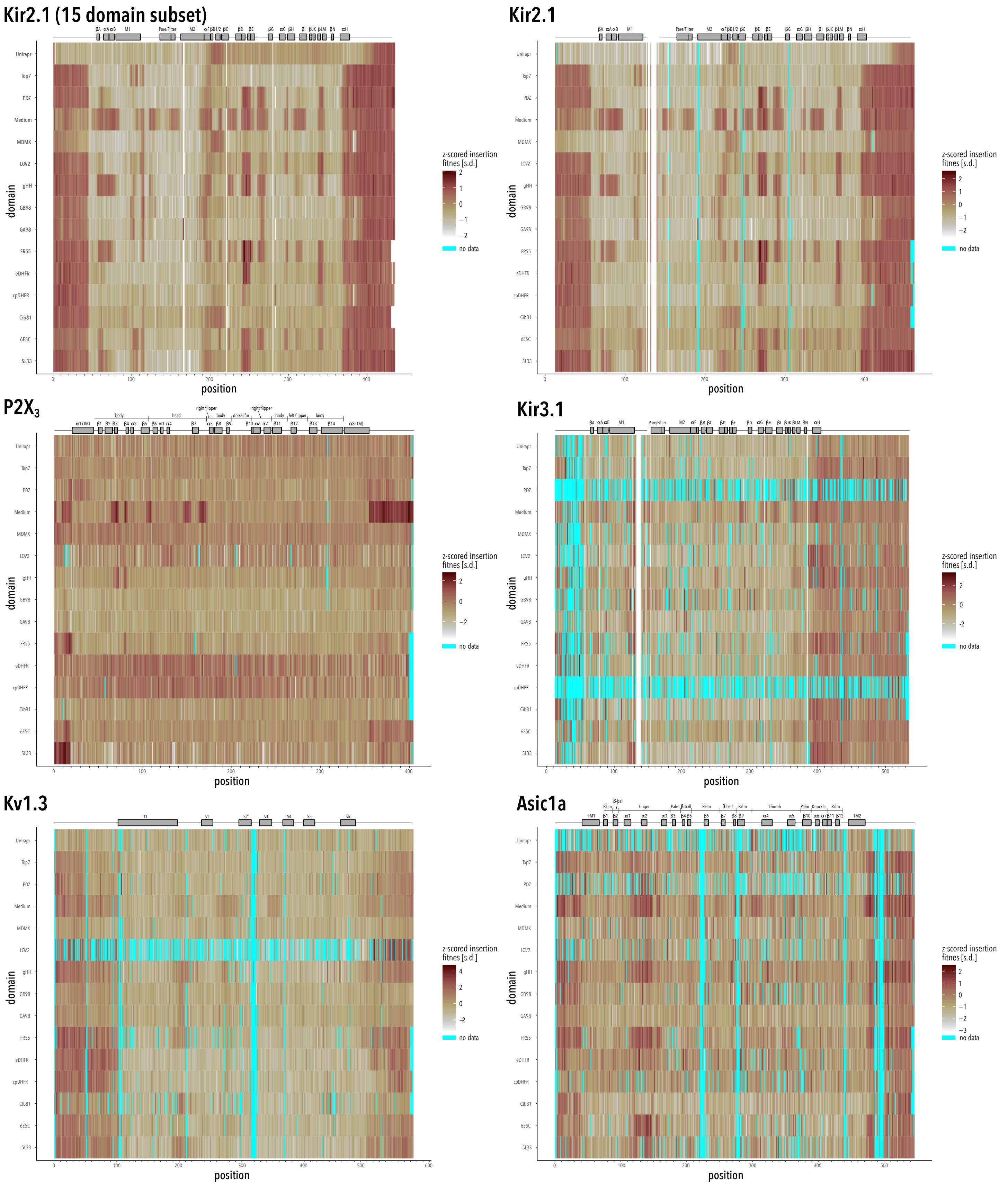

Supplementary Figure 12: Mean insertion fitness across channels and domains. All datasets are based on at least two biological replicates. Two datasets are shown for Kir2.1 that were collected with different sequencing chemistry. Secondary structure elements (and topological organization; $P2X_3$ and Asic1a only) are shown as cartoons.

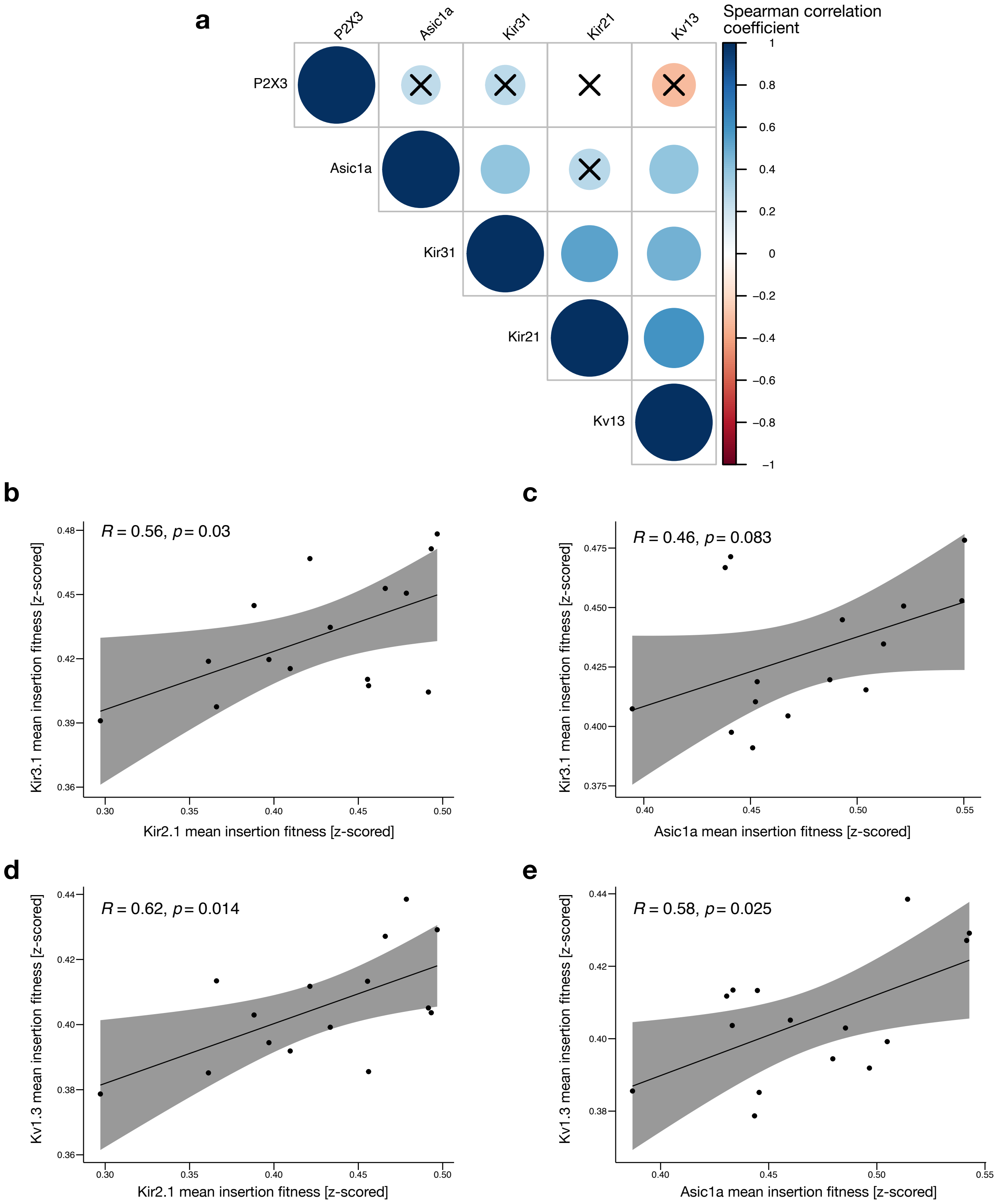

Supplementary Figure 13: Correlation of domain insertion fitness in different ion channels. a Spearman correlation of mean insertion fitness (across all channel positions and motifs) between different channel pairs. Crosses indicate coefficient p-values > 0.05 (i.e., not significant). **b-e** Scatterplots of mean insertion fitness (across all channel position) for each inserted motifs. The solid black line indicates a linear regression and the grey shaded area indicates a 90% confidence interval. Spearman correlation coefficient (R) and p-value are shown for each channel combination. Overall, correlation of motif effects on insertion fitness is moderate, suggesting a minor role relative to recipient channel properties.

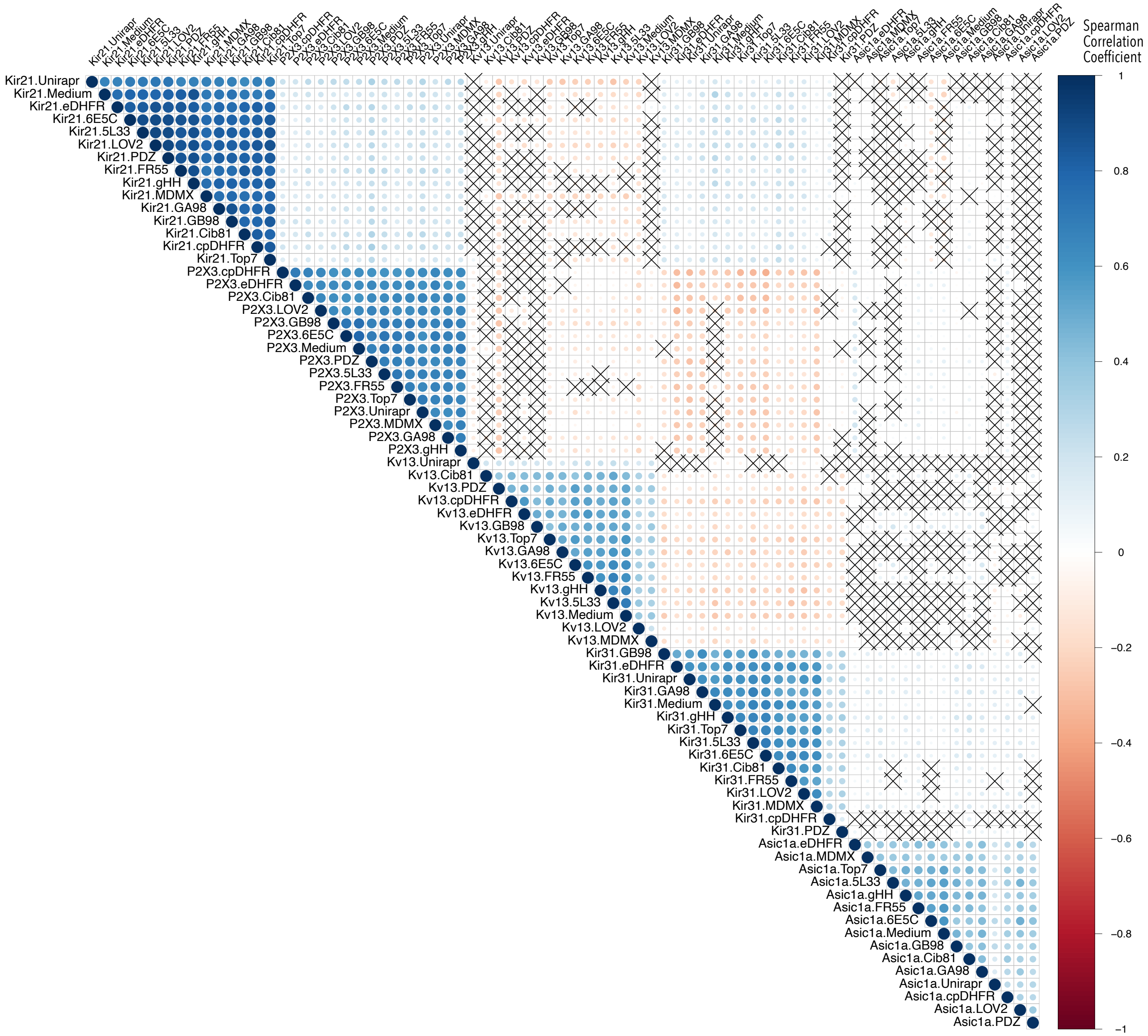

Supplementary Figure 14: Correlations of insertion fitness for motifs in different channels. Spearman correlation of mean insertion fitness (across all channel position) of a specific motif in a specific channels with all other combinations. Strong correlations of different motifs in the same channel background dominate, suggesting that the recipient properties' influence on fitness is strong. Crosses indicate coefficient p-values > 0.05 (i.e., not significant).

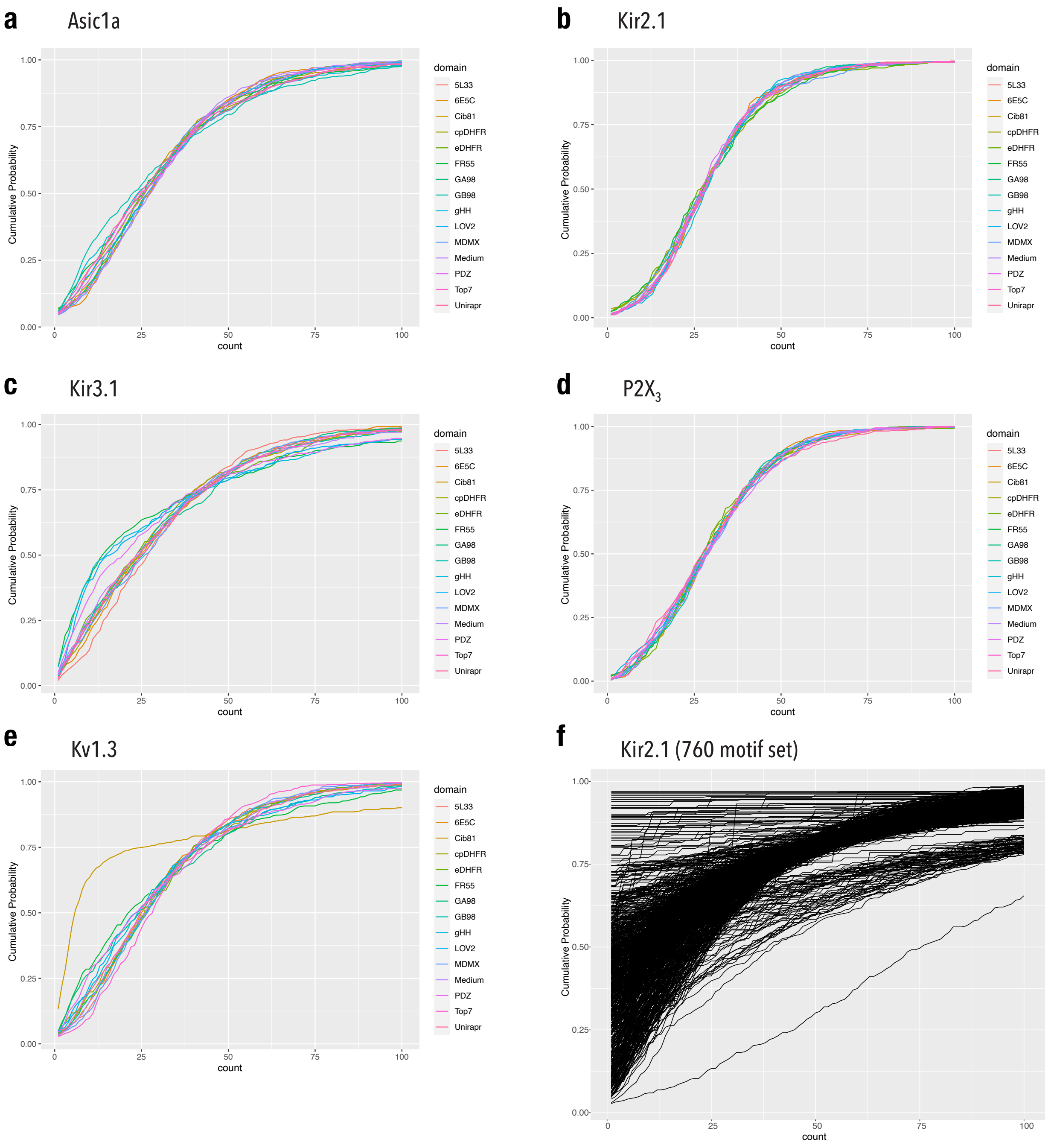

Supplementary Figure 15: Baseline profiles for each domain and gene combination. a-f Empirical cumulative distribution plots for ASIC1a (**a**), Kir2.1 (**b**), Kir3.1 (**c**), $P2X_3$ (**d**), and Kv1.3 (**e**) and the large domain set for Kir2.1. Each domain was normalized to have 30x coverage before calculating empirical cumulative distribution function. Plots show cumulative probability for each count threshold from 1 to 100. This indicates distribution of insertions in a given gene with distributions shifted to the right being more evenly distributed.

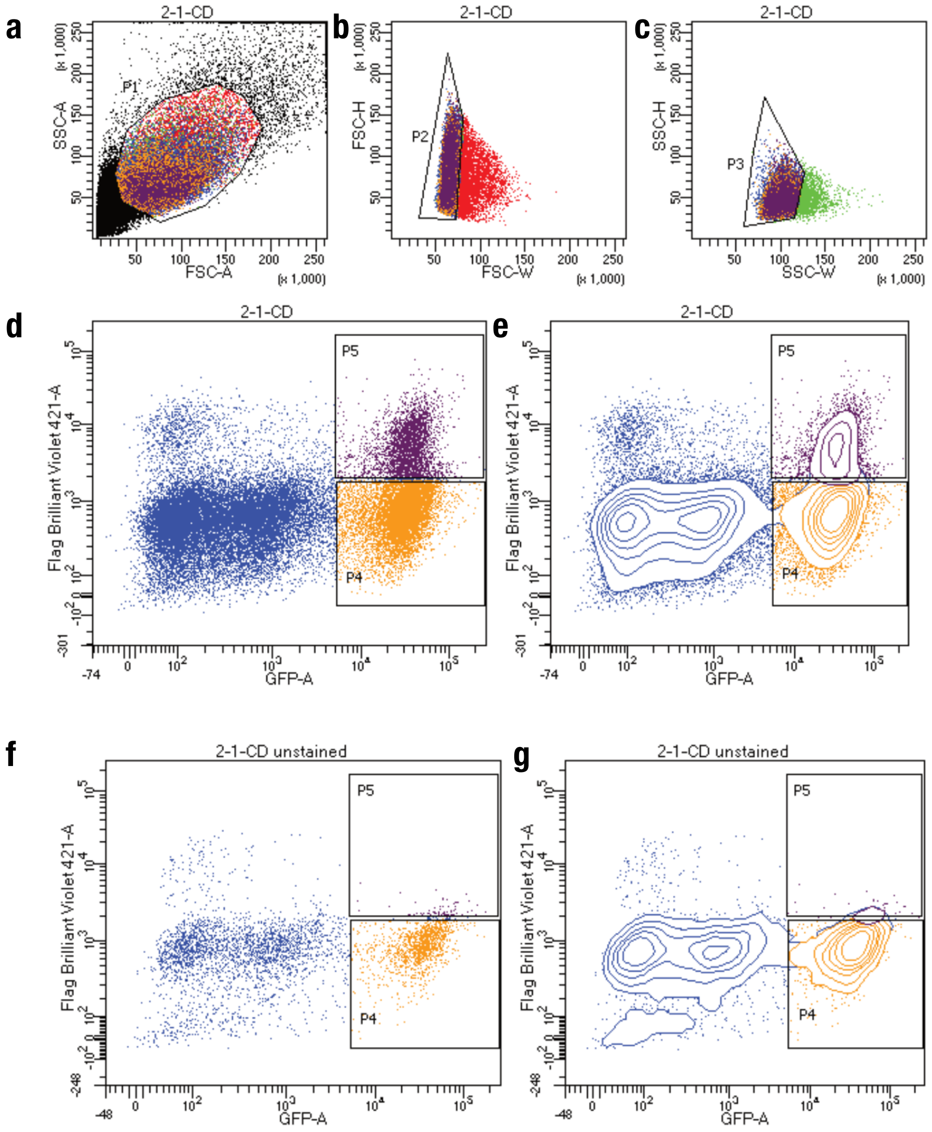

Supplementary Figure 16: Kir2.1 surface expression assay gating scheme. a Whole HEK293 cells are gated on side (SSC-A) and forward scattering (FSC-A). **b-c** Forward scattering height (SSC-H), forward scattering width (FSC-W), and Side scattering width (SSC-W) are used to gate single cells. **d-g** $EGFP_{high}$/$Label_{low}$ and $EGFP_{high}$/ $Label_{high}$ populations are gated based (**d-e**) stained and (**f-g**) unstained on EGFP (GFP-A) of Anti-Flag Brilliant Violet-421 fluorescence with (**d,f**) scatterplot and (**e,g**) contour plots shown. Contour plots represent 95% confidence intervals with outliers shown as dots.

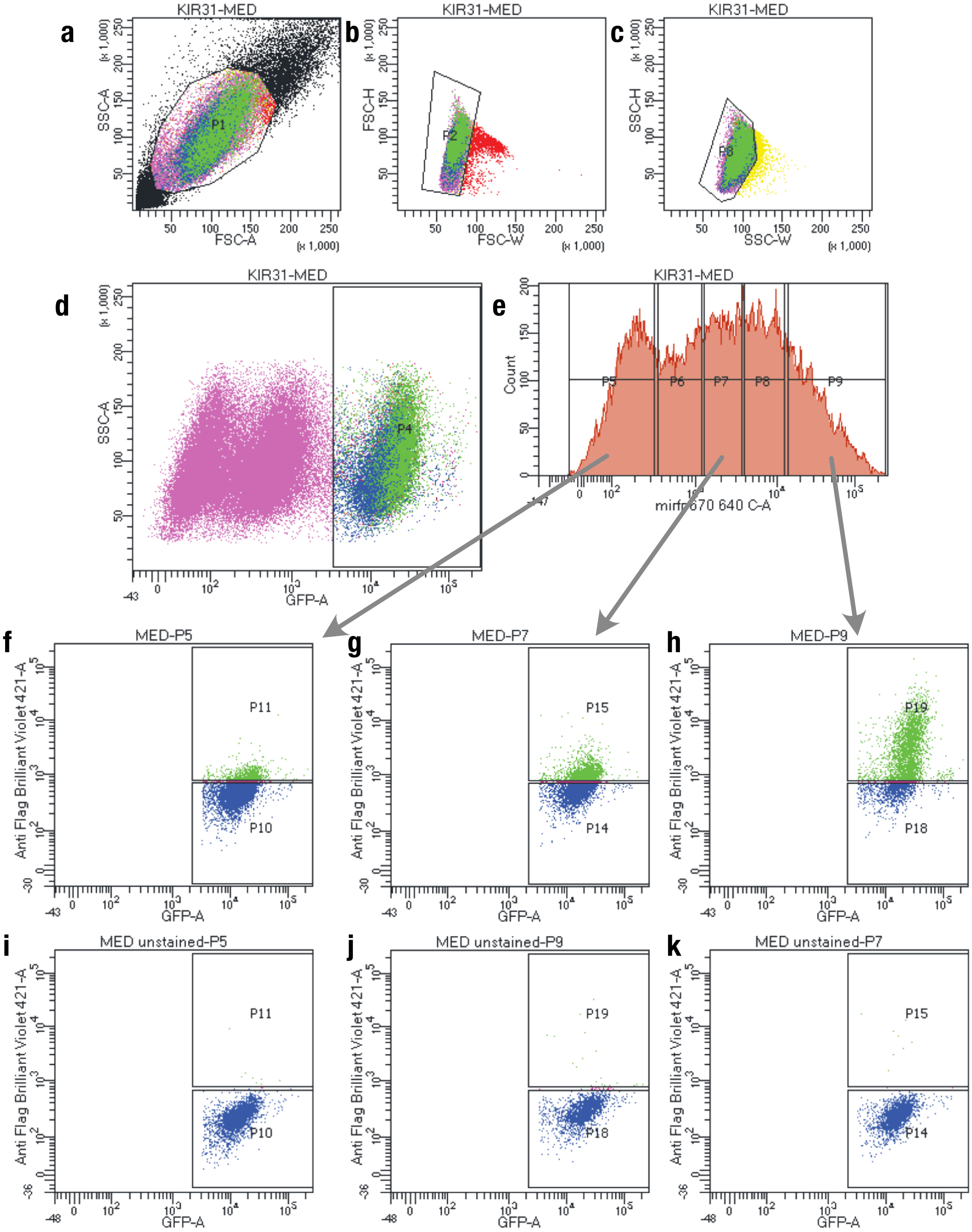

Supplementary Figure 17: Kir3.1 surface expression assay gating scheme. **a** Whole HEK293 cells are gated on side (SSC-A) and forward scattering (FSC-A). **b-c** Forward scattering height (SSC-H), forward scattering width (FSC-W), and Side scattering width (SSC-W) are used to gate single cells. **d** Cells are gated on EGFP positive cells to isolate successfully recombined libraries. **e** Cells are further split into 5 populations to separate out different populations of Kir3.2 co-expressed miRFP670. **f-k** $EGFP_{high}$/ $Label_{low}$ and $EGFP_{high}$/$Label_{high}$ populations are gated based (**f-h**) stained and (**i-k**) unstained on EGFP (GFP-A) of Anti-Flag Brilliant Violet-421 fluorescence. The data from 3 highest levels of miRFP670 were combined and reported as fitness.

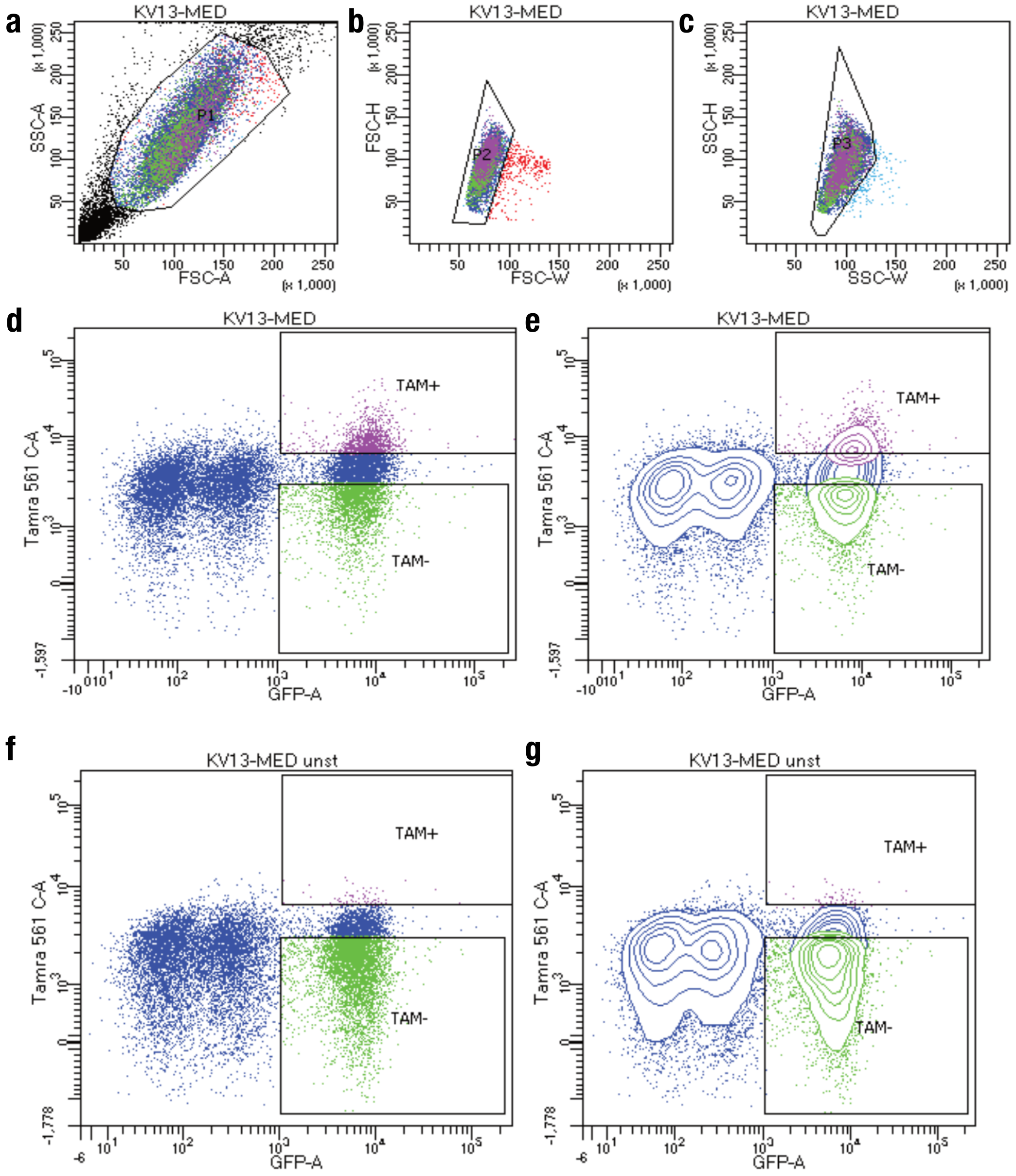

Supplementary Figure 18: Kv1.3 Surface expression assay gating scheme. a Whole HEK293 cells are gated on side (SSC-A) and forward scattering (FSC-A). **b-c** Forward scattering height (SSC-H), forward scattering width (FSC-W), and Side scattering width (SSC-W). **d-g** $EGFP_{high}$/ $Label_{low}$ and $EGFP_{high}$/$Label_{high}$ populations are gated based (**d-e**) stained and (**f-g**) unstained on EGFP (GFP-A) of Kv1.3 specific Agitoxin-Tamra fluorescence with (**d,f**) scatterplot and (**e,g**) contour plots shown. Contour plots represent 95% confidence intervals with outliers shown as dots.

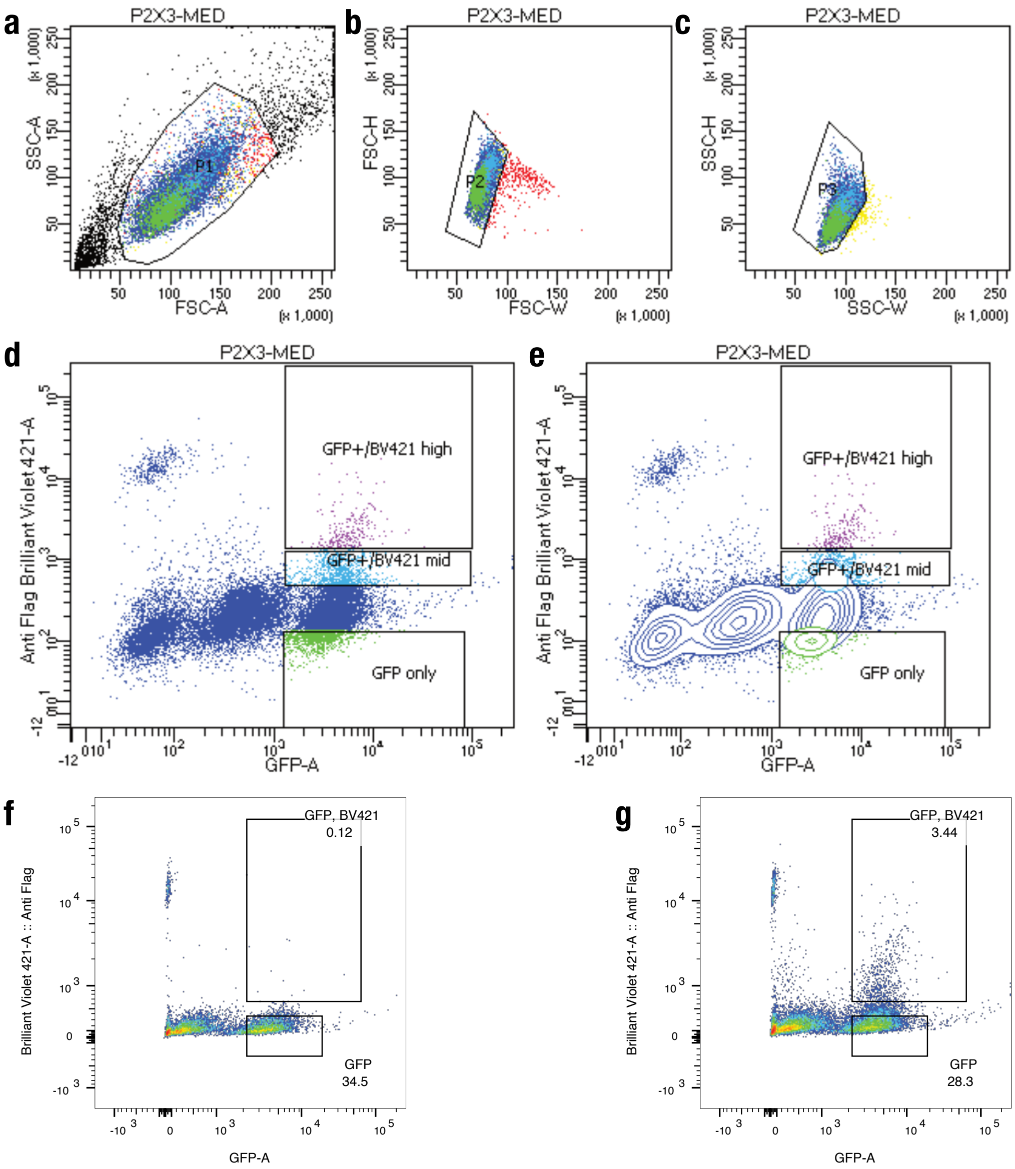

Supplementary Figure 19: $P2X_3$ Surface expression assay gating scheme. a Whole HEK293 cells are gated on side (SSC-A) and forward scattering (FSC-A). **b-c** Forward scattering height (SSC-H), forward scattering width (FSC-W), and Side scattering width (SSC-W) are used to gate single cells. **d-e** $EGFP_{high}$/ $Label_{low}$, EGFP $_{high}$/$Label_{low}$, $EGFP_{high}$/$Label_{med}$ and $EGFP_{high}$/$Label_{high}$ populations are gated based (**f**) stained and (**g**) unstained on EGFP (GFP-A) of Anti-Flag Brilliant Violet-421 fluorescence with (**d**) scatterplot, (**e**) Contour plot, and (**f-g**) pseudo color plots. In post sample collection, *Mid* and *High* label populations were combined ratiometrically based on percent populations in corresponding gates. Contour plots represent 95% confidence intervals with outliers shown as dots. Pseudo-color plots represent density of points with a blue-to-red color scale with increasing density.

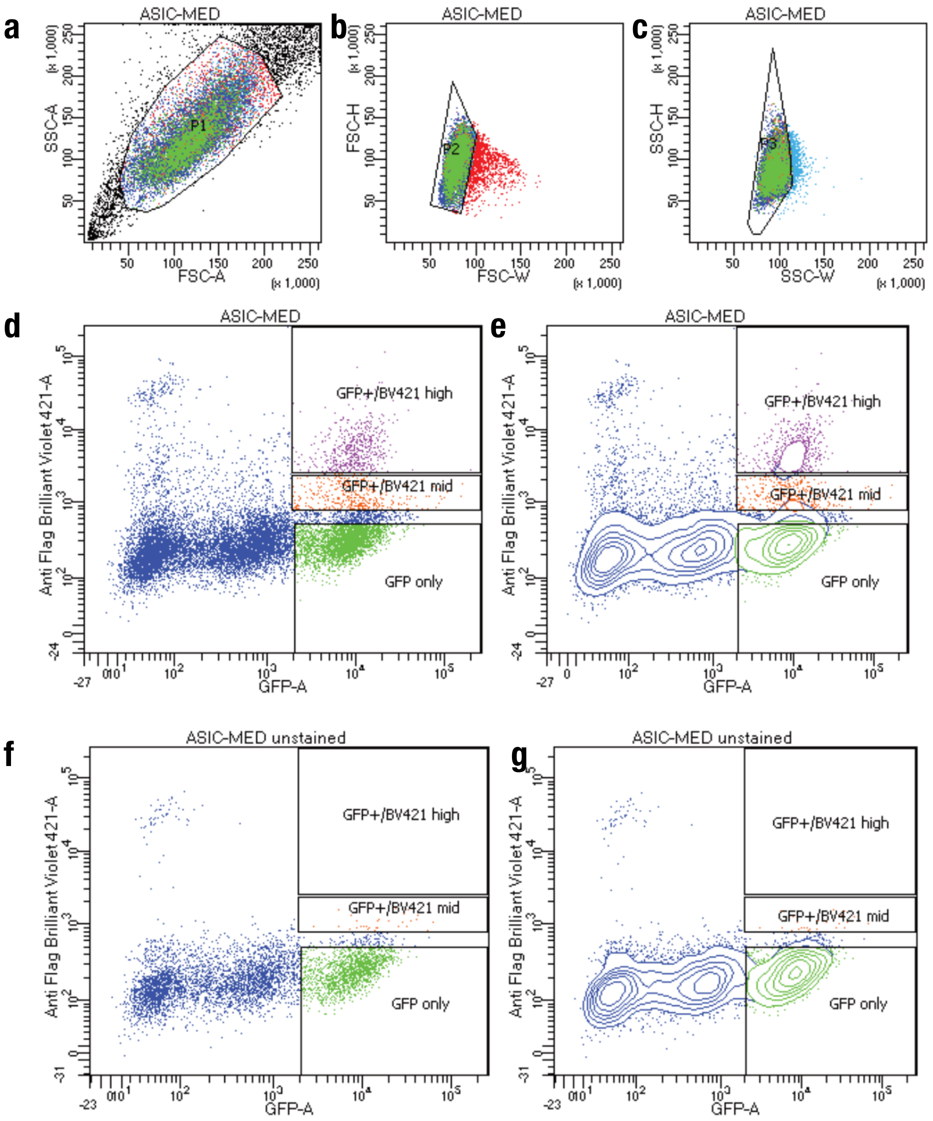

Supplementary Figure 20: ASIC1a Surface expression assay gating scheme. a Whole HEK293 cells are gated on side (SSC-A) and forward scattering (FSC-A). **b-c** Forward scattering height (SSC-H), forward scattering width (FSC-W), and Side scattering width (SSC-W) are used to gate single cells. **d-g** EGFP$_{high}$/ Label$_{low}$ and EGFP$_{high}$/Label$_{high}$ populations are gated based (**d-e**) stained and (**f-g**) unstained on EGFP (GFP-A) of Anti-Flag Brilliant Violet-421 fluorescence with (**d,f**) scatterplot and (**e,g**) contour plots shown. Contour plots represent 95% confidence intervals with outliers as shown as dots.

Motif	Number of motifs	Number of motifs pass QC (%)	Reference
common domains	20	20 (100%)	1
disordered protein fragments	105	89 (85%)	2
disordered proteins	54	27 (50%)	2
manually curated motifs	15	15 (100%)	n/a
polypeptide linkers	5	5 (100%)	n/a
ancestral motifs	40	38 (97%)	3
small non-domain proteins	5	5 (83%)	n/a
smotifs	39	38 (97%)	4
natural proteins < 50 AA	467	391 (84%)	5
peptide toxins	9	9 (100%)	n/a
Total	759	637 (84%)	

Supplementary Table 1: Motif group statistics for Kir2.1 759 motif dataset. Number of motifs, number of motifs passing QC threshold, and sources. Motifs pass QC if they contain statistically significant data in greater than 80% of insertion positions and are included in further analysis and model building.

Motif property	Abbreviation	Mean +/- SD	Reference
Motif Length [AA]	Motif_length	37.2 +/- 22.2	n/a
Phi Mean [degrees]	d_phi_mean	-68.0 +/- 12.4	Pymol
Psi Mean [degrees]	d_psi_mean	-0.49 +/- 35.9	Pymol
Radius of Gyration [Å]	d_gyradius	12.3 +/- 3.3	Pymol
NC distance [Å]	d_nc_dist	12.3 +/- 3.3	Pymol
Distance of N term to center of mass [Å]	d_center_n_dist	23.8 +/- 12.8	Pymol
Distance of C term to center of mass [Å]	d_center_c_dist	23.0 +/- 11.8	Pymol
Contact degree [AU]	d_contact_degree	450 +/- 287	6
Contact order [AU]	d_contact_order	0.41 +/- 0.038	6
Long contact degree [AU]	d_long_degree	7.99 +/- 9.12	6
Secondary Structure (%)	d_sspercent	60.0 +/- 25.0	6
Alpha helical [%]	d_alpha_percent	53.9 +/- 31.3	6
Beta sheet [%]	d_beta_percent	6.1 +/- 13.6	6
Buried nonpolar surface area [Å^2]	d_npsa	2100 +/- 1990	6
Charged solvent accessible surface area [Å^2]	d_charged_mean	39,600 +/- 53,700	6
Polar solvent accessible surface area [Å^2]	d_polar_mean	40,710 +/- 56,000	6
Hydrophobic solvent accessible surface area [Å^2]	d_hydrophob_mean	69,000 +/- 88,000	6
Root mean squared deviation between conformers	d_rmsd	2.98 +/- 2.25	Pymol
Stiffness [AU]	d_stiffness_mean	-7.62E-18 +/- 1.08 E-15	7
Mean AA Molecular Weight [Da]	d_AA_MW_mean	130. +/- 7.49	8
Mean AA Surface area [Å^2]	d_AA_SA_mean	158 +/- 16	8
Mean AA Alpha helical propensity [AU]	d_AA_alphahel_mean	1.04 +/- 0.07	8
Mean AA Beta sheet propensity [AU]	d_AA_betashe_mean	0.99 +/- 0.07	8
Mean AA Buried accessibility ratio propensity [AU]	d_AA_bur_acc_ratio_mean	1.25 +/- 0.29	8
Mean AA flexibility [AU]	d_AA_flex_mean	0.44 +/- 0.02	8
Mean AA hydropathy [AU]	d_AA_hydropath_mean	-0.43 +/- 0.84	8
Mean AA hydrophobicity [AU]	d_AA_hydrophob_mean	2.5 +/- 0.26	8
Mean AA negative charge	d_AA_negat_mean	0.117 +/- 0.079	8
Mean AA pka	d_AA_pka_mean	4.28 +/- 0.28	8
Mean AA polarity [AU]	d_AA_polar_mean	8.6 +/- 0.7	8
Mean AA positive charge	d_AA_posit_mean	0.17 +/- 0.09	8
Mean AA reverse turn propensity [AU]	d_AA_rev_turn_mean	0.97 +/- 0.11	8
Mean AA volume [Å^3]	d_AA_vol_mean	79.1 +/- 10.7	8
Length of structures [AA]	d_size	36.6 +/- 20.1	6

Supplementary Table 2: Inserted motif properties. This table contains means and standard deviations of the inserted motif properties. See Supplementary Data 4 for all calculated properties. Å refers to Angstroms, AA refers to amino acids, Da refers to Daltons, and AU to arbitrary units.

Recipient insertion position property	Abbreviation	Mean +/- SD	Reference
MD Root mean square fluctuation 3SPI (AU)	rmsf_3spi	0.96 +/- 0.70	n/a
MD Root mean square fluctuation 3JYC(AU)	rmsf_3jyc	1.14 +/- 0.85	n/a
Phi (Degrees)	phi	-75.6 +/- 57.7	Pymol
Psi (Degrees)	psi	41.3 +/- 88.4	Pymol
Contact degree (AU)	cdegree	1116.5 +/- 92.0	6
Contact order (AU)	corder	0.439 +/- 0.036	6
Long contact degree (AU)	longdegree	0.863 +/- 0.072	6
Secondary Structure (percentage)	ss	0.60 +/- 0.49	6
Alpha helix (percentage)	alpha	0.33 +/- 47	6
Beta sheet (percentage)	beta	0.27 +/- 0.44	6
Buried nonpolar surface area (A²)	npsa	-12.2 +/- 144.4	6
Charged solvent accessible surface area (A²)	chargedsasa	13,069 +/- 24,866	6
Polar solvent accessible surface area (A²)	polarsasa	16,100 +/- 26,697	6
Normal Mode based Stiffness (AU)	stiffness	10.33 +/- 1.12	7
AA Surface area (A²)	AA_SA	159.5 +/- 57.9	8
AA Buried accessibility ratio propensity (AU)	AA_bur_acc_ratio	1.41 +/- 1.17	8
AA Alpha helical propensity (AU)	AA_alphahel	1.03 +/- 0.25	8
AA Beta sheet propensity (AU)	AA_betashe	1.02 +/- 0.26	8
AA reverse turn propensity (AU)	AA_rev_turn	0.94 +/- 0.38	8
AA volume (A³)	AA_vol	79.7 +/- 39.1	8
AA flexibility (AU)	AA_flex	0.438 +/- 0.075	8
AA Buried accessibility ratio propensity (AU)	AA_bur_vol	146 +/- 39	8
AA Molecular weight (Da)	AA_MW	131 +/- 27	8
AA positive charge	AA_posit	0.133 +/- 0.340	8
AA negative charge	AA_negat	0.140 +/- 0.35	8
AA pka	AA_pka	4.33 +/- 1.02	8
AA polarity (AU)	AA_polar	8.43 +/- 2.72	8
AA hydropathy (AU)	AA_hydropath	-0.133 +/-3.138	8
AA Hydrophobicity (AU)	AA_hydrophob	2.62 +/- 1.02	8

Supplementary Table 3: Recipient insertion position properties. This table contains means and standard deviations of the insertion position properties. See Supplementary Data 5 for all calculated properties. Å refers to Angstroms, AA refers to amino acids, Da refers to daltons, and AU to arbitrary units.

Random Forest	Variance explained (%)	Mean square residuals	Recipient properties (#)	Motif properties (#)	Total properties (#)
Initial	39.89	0.652	37	32	69
Intermediate	39.44	0.657	10	8	18
Final	38.69	0.658	6	4	10

Supplementary Table 4: Random forest parameters. Despite substantially reducing the number of properties, model performance based on variance explained and mean squared residuals are not significantly impacted.

Motif	Length (AA)	Natural or designed	Reference
AGSAGSA	7	Designed	n/a
Syntrophin PDZ	86	Natural	9
Cib81	81	Natural	10
E. coli cpDHFR	164	Modified	11
E. coli DHFR	164	Natural	12
FR55	82	Designed	n/a
GA98	56	Designed	13
GB98	56	Designed	13
ghhh06	43	Designed	14
Unirapr	198	Designed	15
A. sativa LOV2	143	Natural	16
MDMX	103	Natural	17
Top7	99	Designed	18
5L33	108	Designed	19
6E5C	73	Designed	20

Supplementary Table 5: Smaller set of 15 motifs.

Supplementary Table 6: Read Count Statistics. Total reads, mean quality, and aligned reads for each domain pool, replicate, and sort condition.

Sequencing Run	Pool Name	Target Gene(s)	Sample Type	Replicate	Total Reads in Pool	Mean Quality	Aligned Reads	Number of Domains	Number of Positions	Number of Variants	Coverage [x-fold]
Project_047	base_1-16	Kir2.1	pre-sort control	1	81,371,890	34.1	7,482,860	560	435	243,600	30.7
	base_17-21	Kir2.1	pre-sort control	1	24,346,753	32.1	2,097,244	175	435	76,125	27.6
	base_CD	Kir2.1	pre-sort control	1	4,304,294	30.8	341,611	45	435	19,575	17.5
	dp_1_1-16	Kir2.1	surface expression	1	79,873,354	34.1	7,767,341	560	435	243,600	31.9
	dp_1_17-21	Kir2.1	surface expression	1	19,242,372	31.0	1,511,063	175	435	76,125	19.8
	dp_1_CD	Kir2.1	surface expression	1	2,847,220	29.2	124,711	45	435	19,575	6.4
	dp_2_11-12X15	Kir2.1	surface expression	2	9,683,945	30.2	764,060	105	435	45,675	16.7
	dp_2_13-14X18	Kir2.1	surface expression	2	10,853,687	31.0	311,539	105	435	45,675	6.8
	dp_2_19-21X16-17	Kir2.1	surface expression	2	19,264,855	29.6	1,289,393	175	435	76,125	16.9
	dp_2_3-10	Kir2.1	surface expression	2	35,490,287	33.1	2,762,399	280	435	121,800	22.7
	dp_2_CD	Kir2.1	surface expression	2	3,203,291	30.9	211,588	45	435	19,575	10.8
	gfp_1_1-16	Kir2.1	no surface expression	1	77,668,001	34.1	7,768,024	560	435	243,600	31.9
	gfp_1_17-21	Kir2.1	no surface expression	1	27,686,561	32.3	2,270,580	175	435	76,125	29.8
	gfp_1_CD	Kir2.1	no surface expression	1	4,408,355	31.4	382,483	45	435	19,575	19.5
	gfp_2_11-12X15	Kir2.1	no surface expression	2	9,433,539	31.2	937,905	105	435	45,675	20.5
	gfp_2_13-14X18	Kir2.1	no surface expression	2	9,369,676	31.1	306,212	105	435	45,675	6.7
	gfp_2_19-21X16-17	Kir2.1	no surface expression	2	21,731,747	30.9	2,014,803	175	435	76,125	26.5
	gfp_2_3-10	Kir2.1	no surface expression	2	37,722,582	33.5	3,698,554	280	435	121,800	30.4
	gfp_2_CD	Kir2.1	no surface expression	2	5,126,808	29.8	415,326	45	435	19,575	21.2
	Pool1	Kir2.2	high surface expression	1	8,757,200.00	34.6	415,084	15	441	6,615	63
		Kir2.2	no surface expression	2			522,629	15	441	6,615	79
		Kir3.1	no surface expression (level 1)	1			824,546	15	509	7,635	108
		Kir3.2	no surface expression	2			1,025,554	15	425	6,375	161
	Pool2	Asic1a	low surface expression	1	8,865,777.00	34.8	477,966	15	546	8,190	58
		Kir2.2	no surface expression	1			371,359	15	441	6,615	56
		Kir3.1	no surface expression (level 4)	1			1,007,295	15	509	7,635	132
		Kir3.2	high surface expression	2			988,708	15	425	6,375	155
	Pool3	Asic1a	high surface expression	1	8,473,412.00	34.8	330,452	15	546	8,190	40
		Kir2.2	low surface expression	1			517,070	15	441	6,615	78
		Kir3.1	no surface expression (level 3)	1			1,105,690	15	509	7,635	145
	Pool4	Asic1a	no surface expression	1	6,908,979.00	34.5	661,693	15	546	8,190	81
		Kir3.1	pre-sort control	1			36,969	15	509	7,635	5
		Kv1.3	pre-sort control	2			422,491	15	575	8,625	49
	Pool5	Asic1a	low surface expression	1	10,277,728.00	34.8	477,966	15	546	8,190	58
		Kir2.2	pre-sort control	1			667,951	15	441	6,615	101
		Kir3.1	no surface expression (level 5)	1			1,026,938	15	509	7,635	135
	Pool6	Asic1a	high surface expression	1	8,714,527.00	34.2	330,452	15	546	8,190	40
		Kir2.2	pre-sort control	1			667,951	15	441	6,615	101
		Kir3.1	surface expression (level 1)	1			910,532	15	509	7,635	119
	Pool7	Asic1a	pre-sort control	1	11,006,193.00	34.8	656,722	15	546	8,190	80
		Kir3.2	no surface expression	1			766,766	15	425	6,375	120
		Kir3.1	surface expression (level 2)	1			717,335	15	509	7,635	94
	Pool8	Asic1a	pre-sort control	1	6,180,966.00	34.5	656,722	15	546	8,190	80
		Kir3.1	pre-sort control	1			36,969	15	509	7,635	5
		Kv1.3	high surface expression	2			798,555	15	575	8,625	93
	Pool9	Asic1a	no surface expression	2	8,177,441.00	34.8	330,452	15	546	8,190	40
		Kir3.2	no surface expression	1			766,766	15	425	6,375	120
		Kir3.1	surface expression (level 4)	1			770,673	15	509	7,635	101
	Pool10	Asic1a	low surface expression	2	9,409,597.00	34.7	477,966	15	546	8,190	58
		Kir3.2	high surface expression	1			829,744	15	425	6,375	130
		Kir3.1	surface expression (level 5)	1			700,007	15	509	7,635	92
	Pool11	Asic1a	high surface expression	2	8,823,792.00	34.8	661,693	15	546	8,190	81
		Kir3.2	pre-sort control	1			1,324,434	15	425	6,375	208
		Kir3.1	no surface expression (level 1)	1			824,546	15	509	7,635	108
	Pool12	Asic1a	no surface expression	2	9,424,282.00	34.8	330,452	15	546	8,190	40
		Kir3.2	pre-sort control	1			1,324,434	15	425	6,375	208
		Kir3.1	no surface expression (level 2)	1			757,734	15	509	7,635	99
	Pool13	Asic1a	low surface expression	2	9,877,526.00	34.7	477,966	15	546	8,190	58
		Kir2.2	no surface expression	2			522,629	15	441	6,615	79
		Kir3.1	no surface expression (level 3)	1			1,105,690	15	509	7,635	145
		Asic1a	high surface expression	2			661,693	15	546	8,190	81

Project _045	Pool14	Kir2.2	low surface expression	2	8,947,322.00	34.9	718,837	15	441	6,615	109
		Kir3.1	no surface expression (level 4)	1			1,007,295	15	509	7,635	132
		P2X4	no surface expression	1			464,622	15	396	5,940	78
	Pool15	Kir2.2	high surface expression	2	8,520,972.00	34.8	523,148	15	441	6,615	79
		Kir3.1	no surface expression (level 5)	1			1,026,938	15	509	7,635	135
		Asic1a	no surface expression	1			661,693	15	546	8,190	81
	Pool16	P2X4	low surface expression	2	7,485,544.00	34.8	609,143	15	396	5,940	103
		Kir3.1	surface expression (level 3)	2			661,552	15	509	7,635	87
		P2X4	high surface expression	1			242,095	15	396	5,940	41
	Pool17	Kir2.2	low surface expression	2	5,726,099.00	34.9	718,837	15	441	6,615	109
		Kir3.1	surface expression (level 2)	1			717,335	15	509	7,635	94
		P2X4	no surface expression	1			464,622	15	396	5,940	78
	Pool18	Kir2.2	high surface expression	2	6,981,745.00	35.0	523,148	15	441	6,615	79
		Kir3.1	surface expression (level 3)	1			721,893	15	509	7,635	95
		P2X4	low surface expression	1			609,143	15	396	5,940	103
	Pool19	Kir2.1	no surface expression	1	6,635,382.00	34.9	771,084	15	436	6,540	118
		Kir3.1	surface expression (level 4)	1			770,673	15	509	7,635	101
		P2X4	high surface expression	1			242,095	15	396	5,940	41
	Pool20	Kir2.1	low surface expression	1	6,512,643.00	34.9	657,683	15	436	6,540	101
		Kir3.1	surface expression (level 5)	1			700,007	15	509	7,635	92
		P2X4	no surface expression	2			687,217	15	396	5,940	116
	Pool21	Kir2.1	high surface expression	1	7,124,671.00	35.0	324,519	15	436	6,540	50
		Kir3.1	no surface expression (level 4)	1			1,007,295	15	509	7,635	132
		P2X4	low surface expression	2			399,762	15	396	5,940	67
	Pool22	Kir2.1	no surface expression	1	6,864,512.00	35.0	771,084	15	436	6,540	118
		Kir3.1	surface expression (level 3)	1			721,893	15	509	7,635	95
		P2X4	high surface expression	2			328,645	15	396	5,940	55
	Pool23	Kir2.1	low surface expression	1	6,140,927.00	34.9	657,683	15	436	6,540	101
		Kir3.1	no surface expression (level 1)	2			876,508	15	509	7,635	115
		P2X4	no surface expression	2			687,217	15	396	5,940	116
	Pool24	Kir2.1	high surface expression	1	7,243,070.00	34.9	324,519	15	436	6,540	50
		Kir3.1	no surface expression (level 2)	2			816,146	15	509	7,635	107
		P2X4	low surface expression	2			399,762	15	396	5,940	67
	Pool25	Kir2.1	pre-sort control	1	6,105,966.00	34.9	990,928	15	436	6,540	152
		Kir3.1	no surface expression (level 3)	2			702,542	15	509	7,635	92
		P2X4	high surface expression	2			328,645	15	396	5,940	55
	Pool26	Kir2.1	pre-sort control	1	8,869,273.00	34.9	990,928	15	436	6,540	152
		Kir3.1	no surface expression (level 4)	2			764,153	15	509	7,635	100
		Asic2a	no surface expression	1			848,345	15	520	7,800	109
	Pool27	Kir2.1	no surface expression	2	7,751,443.00	34.7	1,110,887	15	436	6,540	170
		Kir3.1	no surface expression (level 5)	2			849,217	15	509	7,635	111
		Asic2a	low surface expression	1			688,644	15	520	7,800	88
	Pool28	Kir2.1	low surface expression	2	9,176,567.00	34.8	521,489	15	436	6,540	80
		Kir3.1	surface expression (level 1)	2			1,185,484	15	509	7,635	155
		Asic2a	high surface expression	1			455,576	15	520	7,800	58
	Pool29	Kir2.1	high surface expression	2	8,666,582.00	34.8	613,817	15	436	6,540	94
		Kir3.1	surface expression (level 2)	2			1,100,830	15	509	7,635	144
		Asic2a	no surface expression	1			848,345	15	520	7,800	109
	Pool30	Kir2.1	no surface expression	2	8,301,697.00	34.7	1,110,887	15	436	6,540	170
		Kir3.1	no surface expression (level 1)	1			824,546	15	509	7,635	108
		Asic2a	low surface expression	1			688,644	15	520	7,800	88
	Pool31	Kir2.1	low surface expression	2	8,407,257.00	34.8	521,489	15	436	6,540	80
		Kir3.1	surface expression (level 4)	2			801,705	15	509	7,635	105
		Asic2a	high surface expression	1			455,576	15	520	7,800	58
	Pool32	Kir2.1	high surface expression	2	7,610,875.00	34.8	613,817	15	436	6,540	94
		Kir3.1	surface expression (level 5)	2			749,245	15	509	7,635	98
		Kir3.1	no surface expression (level 1)	2			876,508	15	509	7,635	115
	Pool33	Kv1.3	no surface expression	1	9,765,084.00	34.9	744,064	15	575	8,625	86
		Kir6.2	high surface expression	1			1,230,567	15	410	6,150	200
		Kir3.1	no surface expression (level 2)	2			816,146	15	509	7,635	107
	Pool34	Kv1.3	high surface expression	1	8,876,108.00	34.7	434,511	15	575	8,625	50
		Kir6.2	no surface expression	1			1,377,532	15	410	6,150	224
		Kir3.1	no surface expression (level 3)	2			702,542	15	509	7,635	92
	Pool35	Kv1.3	no surface expression	1	10,394,399.00	34.9	744,064	15	575	8,625	86
		Kir6.2	no surface expression	1			1,377,532	15	410	6,150	224
		Kir3.1	no surface expression (level 4)	2			764,153	15	509	7,635	100

	Pool36	Kv1.3	high surface expression	1	7,953,804.00	34.8	434,511	15	575	8,625	50
		Kir6.2	high surface expression	1			1,230,567	15	410	6,150	200
		Kir3.1	no surface expression (level 5)	2			849,217	15	509	7,635	111
	Pool37	P2X3	no surface expression	1	8,420,380.00	34.8	881,800	15	405	6,075	145
		Kir6.2	pre-sort control	1			1,318,939	15	410	6,150	214
		Kir3.1	surface expression (level 1)	2			1,185,484	15	509	7,635	155
	Pool38	P2X3	low surface expression	1	10,764,162.00	34.8	580,254	15	405	6,075	96
		Kir6.2	pre-sort control	1			1,318,939	15	410	6,150	214
		P2X3	high surface expression	1			249,510	15	405	6,075	41
	Pool39	Kv1.3	no surface expression	2	8,298,152.00	34.6	810,399	15	575	8,625	94
		Kir3.1	surface expression (level 2)	2			1,100,830	15	509	7,635	144
		P2X3	no surface expression	1			881,800	15	405	6,075	145
	Pool40	Kv1.3	high surface expression	2	8,880,744.00	34.8	798,555	15	575	8,625	93
		Kir3.1	surface expression (level 3)	2			661,552	15	509	7,635	87
		P2X3	low surface expression	1			580,254	15	405	6,075	96
	Pool41	Kv1.3	no surface expression	2	8,867,974.00	34.8	810,399	15	575	8,625	94
		Kir3.1	surface expression (level 4)	2			801,705	15	509	7,635	105
		P2X3	high surface expression	1			249,510	15	405	6,075	41
	Pool42	Kir3.2	high surface expression	1	7,913,949.00	34.8	829,744	15	425	6,375	130
		Kir3.1	surface expression (level 5)	2			749,245	15	509	7,635	98
		P2X3	no surface expression	2			918,149	15	405	6,075	151
	Pool43	Kv1.3	pre-sort control	2	7,370,069.00	34.8	422,491	15	575	8,625	49
		Kir3.1	no surface expression (level 1)	3			1,005,480	15	509	7,635	132
		P2X3	low surface expression	2			489,186	15	405	6,075	81
	Pool44	Kir2.2	high surface expression	1	7,939,154.00	34.8	415,084	15	441	6,615	63
		Kir3.1	no surface expression (level 5)	3			1,073,309	15	509	7,635	141
		P2X3	high surface expression	2			379,725	15	405	6,075	63
	Pool45	Kir6.2	no surface expression	2	8,527,993.00	34.9	1,066,743	15	410	6,150	173
		Kir3.1	surface expression (level 1)	3			892,611	15	509	7,635	117
		P2X3	no surface expression	2			918,149	15	405	6,075	151
	Pool46	Kir6.2	high surface expression	2	7,948,119.00	34.8	710,829	15	410	6,150	116
		Kir3.1	surface expression (level 5)	3			734,837	15	509	7,635	96
		Kir3.1	no surface expression (level 1)	3			1,005,480	15	509	7,635	132
	Pool47	P2X3	low surface expression	2	11,010,663.00	35.0	489,186	15	405	6,075	81
		Kir6.2	no surface expression	2			1,066,743	15	410	6,150	173
		Kir3.1	no surface expression (level 5)	3			1,073,309	15	509	7,635	141
	Pool48	P2X3	high surface expression	2	8,610,090.00	34.9	379,725	15	405	6,075	63
		Kir6.2	high surface expression	2			710,829	15	410	6,150	116
		Kir3.1	surface expression (level 1)	3			892,611	15	509	7,635	117
	Pool49	Kir2.2	no surface expression	1	8,475,624.00	34.8	371,359	15	441	6,615	56
		Kir3.2	no surface expression	2			1,025,554	15	425	6,375	161
		Kir2.2	low surface expression	1			517,070	15	441	6,615	78
	Pool50	Kir3.1	surface expression (level 5)	3	8,130,236.00	34.9	734,837	15	509	7,635	96
		Kir3.2	high surface expression	2			988,708	15	425	6,375	155

Supplementary Table 7: Alignment of Kir and annotation of structural and functional features.

Feature	Function	Kir2.1(FLAG) Resno	Kir2.1(FLAG) Resid	Kir3.1(FLAG) Resno	Kir3.1(FLAG) Resid	Kir6.2(FLAG) Resno	Kir6.2(FLAG) Resid
		1	M	1	M		
		2	G	2	S		
		3	S	3	A		
		4	V	4	L		
		5	R	5	R	1	M
		6	T	6	R	2	L
		7	N	7	K	3	S
		8	R	8	F	4	R
		9	Y	9	G	5	K
		10	S	10	D	6	G
		11	I	11	D	7	I
		12	V	12	Y	8	I
		13	S	13	Q	9	P
		14	S	14	V	10	E
		15	E	15	V	11	E
		16	E	16	T	12	Y
		17	D	17	T	13	V
		18	G	18	S	14	L
		19	M	19	S	15	T
		20	K	20	S	16	R
		21	L	21	G	17	L
		22	A	22	S	18	A
		23	T	23	G	19	E
		24	M	24	L		
		25	A	25	Q		
		26	V	26	P		
		27	A	27	Q		
		28	N	28	G		
		29	G	29	P		
		30	F	30	G		
		31	G	31	Q	20	D
		32	N	32	G	21	P
		33	G	33	P	22	A
		34	K	34	Q	23	E
		35	S	35	Q	24	P
		36	K	36	Q	25	R
		37	V	37	L	26	Y
		38	H	38	V	27	R
		39	T	39	P	28	A
		40	R	40	K	29	R
		41	Q	41	K	30	Q
		42	Q	42	K	31	R
		43	C	43	R		
		44	R	44	Q	32	R
		45	S			33	A
		46	R	45	R	34	R
		47	F	46	F	35	F
		48	V	47	V	36	V
		49	K	48	D	37	S
	ATP1/PS	50	K	49	K	38	K
		51	D	50	N	39	K
		52	G	51	G	40	G
	PIP2	53	H	52	R	41	N
		54	C	53	C	42	C
		55	N	54	N	43	N
betaA		56	V	55	V	44	V
betaA		57	Q	56	Q	45	A
betaA		58	F	57	H	46	H
betaA		59	I	58	G	47	K
	pad	60	N	59	N	48	N
	pad	61	V	60	L	49	I
	ATP1	62	G	61	G	50	R
	pad			62	S		
	pad	63	E	63	E	51	E
	pad	64	K	64	T	52	Q
alphaA	pad	65	G			53	G
alphaA	pad	66	Q	65	S		
alphaA	pad	67	R	66	R	54	R
alphaA	pad	68	Y	67	Y	55	F
alphaA	pad	69	L	68	L	56	L
alphaA	pad	70	A	69	S	57	Q
	pad	71	D	70	D	58	D
alphaB	pad	72	I	71	L	59	V
alphaB	pad	73	F	72	F	60	F
alphaB	pad	74	T	73	T	61	T
alphaB	pad	75	T	74	T	62	T
alphaB	pad	76	C	75	L	63	L
alphaB	pad	77	V	76	V	64	V
alphaB	pad	78	D	77	D	65	D
	pad	79	I	78	L	66	L
	PIP2/PS	80	R	79	K	67	K
M1	PIP2	81	W	80	W	68	W
M1	PIP2/PS	82	R	81	R	69	P
M1	pad	83	W	82	W	70	H
M1		84	M	83	N	71	T

M1		85	L	84	L	72	L
M1		86	V	85	F	73	L
M1		87	I	86	I	74	I
M1		88	F	87	F	75	F
M1		89	C	88	I	76	T
M1		90	L	89	L	77	M
M1		91	A	90	T	78	S
M1		92	F	91	Y	79	F
M1		93	V	92	T	80	L
M1		94	L	93	V	81	C
M1		95	S	94	A	82	S
M1		96	W	95	W	83	W
M1		97	L	96	L	84	L
M1		98	F	97	F	85	L
M1		99	F	98	M	86	F
M1		100	G	99	A	87	A
M1		101	C	100	S	88	M
M1		102	V	101	M	89	A
M1		103	F	102	W	90	W
M1		104	W	103	W	91	W
M1		105	L	104	V	92	L
M1		106	I	105	I	93	I
M1		107	A	106	A	94	A
M1		108	L	107	Y	95	F
M1		109	L	108	T	96	A
M1		110	H	109	R	97	H
		111	G	110	G	98	G
		112	D	111	D	99	D
		113	L	112	L	100	L
						101	Y
		114	D	113	N	102	A
				114	K	103	Y
				115	D	104	M
				116	Y	105	E
		115	T	117	K	106	K
						107	G
						108	I
						109	T
						110	D
						111	L
						112	A
						113	P
		116	D	118	D	114	D
		117	Y	119	D	115	Y
		118	K	120	D	116	K
		119	D	121	D	117	D
		120	D	122	K	118	D
		121	D	123	A	119	D
		122	D	124	H	120	D
		123	K			121	K
		124	S	125	V	122	S
		125	K	126	G	123	E
		126	V	127	N	124	G
		127	S	128	Y	125	T
		128	K	129	T	126	A
		129	A	130	P	127	E
						128	P
		130	C	131	C	129	C
		131	V	132	V	130	V
		132	S	133	A	131	T
		133	E	134	N	132	S
		134	V	135	V	133	I
		135	N	136	Y	134	H
Pore Helix		136	S	137	N	135	S
Pore Helix		137	F	138	F	136	F
Pore Helix		138	T	139	P	137	S
Pore Helix		139	A	140	S	138	S
Pore Helix		140	A	141	A	139	A
Pore Helix		141	F	142	F	140	F
Pore Helix		142	L	143	L	141	L
Pore Helix		143	F	144	F	142	F
Pore Helix		144	S	145	F	143	S
Pore Helix		145	I	146	I	144	I
Pore Helix		146	E	147	E	145	E
Pore Helix		147	T	148	T	146	V
Pore Helix		148	Q	149	E	147	Q
Pore Helix		149	T	150	A	148	V
Filter		150	T	151	T	149	T
Filter		151	I	152	I	150	I
Filter		152	G	153	G	151	G
Filter		153	Y	154	Y	152	F
Filter		154	G	155	G	153	G
		155	F	156	Y	154	G
		156	R	157	R	155	R
		157	C	158	Y	156	M
		158	V	159	I	157	V

		159	T	160	T	158	T
		160	D	161	D	159	E
		161	E	162	K	160	E
M2		162	C	163	C	161	C
M2		163	P	164	P	162	P
M2		164	I	165	E	163	L
M2		165	A	166	G	164	A
M2		166	V	167	I	165	I
M2		167	F	168	I	166	L
M2		168	M	169	L	167	I
M2		169	V	170	F	168	L
M2		170	V	171	L	169	I
M2		171	F	172	F	170	V
M2		172	Q	173	Q	171	Q
M2		173	S	174	S	172	N
M2		174	I	175	I	173	I
M2		175	V	176	L	174	V
M2		176	G	177	G	175	G
M2		177	C	178	S	176	L
M2		178	I	179	I	177	M
M2		179	I	180	V	178	I
M2		180	D	181	D	179	N
M2		181	A	182	A	180	A
M2		182	F	183	F	181	I
M2		183	I	184	L	182	M
M2		184	I	185	I	183	L
M2		185	G	186	G	184	G
M2		186	A	187	C	185	C
M2		187	V	188	M	186	I
M2		188	M	189	F	187	F
M2	pad	189	A	190	I	188	M
M2	PIP2/PS	190	K	191	K	189	K
M2	PS	191	M	192	M	190	T
M2	pad	192	A	193	S	191	A
	PIP2/PS	193	K	194	Q	192	Q
alphaF	PIP2	194	P	195	P	193	A
alphaF	PIP2	195	K	196	K	194	H
alphaF	PIP2/PS	196	K	197	K	195	R
alphaF	pad	197	R	198	R	196	R
alphaF		198	N	199	A	197	A
alphaF		199	E	200	E	198	E
alphaF		200	T	201	T	199	T
betaB1	pad	201	L	202	L	200	L
betaB1	ATP2	202	V	203	M	201	I
betaB1	pad	203	F	204	F	202	F
	pad	204	S	205	S	203	S
	ATP1	205	H	206	E	204	K
	pad	206	N	207	H	205	H
betaB2		207	A	208	A	206	A
betaB2		208	V	209	V	207	V
betaB2		209	I	210	I	208	I
betaB2		210	A	211	S	209	A
betaB2		211	M	212	M	210	L
betaB2		212	R	213	R	211	R
		213	D	214	D	212	H
		214	G	215	G	213	G
betaC		215	K	216	K	214	R
betaC		216	L	217	L	215	L
betaC		217	C	218	T	216	C
betaC		218	L	219	L	217	F
betaC		219	M	220	M	218	M
betaC		220	W	221	F	219	L
betaC		221	R	222	R	220	R
betaC	pad	222	V	223	V	221	V
betaC	pad	223	G	224	G	222	G
betaC	pad	224	N	225	N	223	D
	ATP2	225	L	226	L	224	L
	pad	226	R	227	R	225	R
	PIP2/PS	227	K	228	N	226	K
	pad	228	S	229	S	227	S
	pad	229	H	230	H	228	M
	pad	230	L	231	M	229	I
		231	V	232	V	230	I
		232	E	233	S	231	S
		233	A	234	A	232	A
betaD1		234	H	235	Q	233	T
betaD1		235	V	236	I	234	I
betaD1		236	R	237	R	235	H
betaD1		237	A	238	C	236	M
betaD1		238	Q	239	K	237	Q
betaD1		239	L	240	L	238	V
betaD1		240	L	241	L	239	V
betaD1	pad	241	K	242	K	240	R
	Gbg	242	S	243	S	241	K
betaD2	pad	243	R	244	R	242	T
betaD2	Gbg	244	I	245	Q	243	T

betaD2	Gbg	245	T	246	T	244	S
	Gbg	246	S	247	P	245	P
	Gbg	247	E	248	E	246	E
	Gbg	248	G	249	G	247	G
betaE1	Ga	249	E	250	E	248	E
betaE1	Gbg	250	Y	251	F	249	V
betaE1	pad	251	I	252	L	250	V
	Gbg	252	P	253	P	251	P
	pad	253	L	254	L	252	L
betaE2		254	D	255	D	253	H
betaE2		255	Q	256	Q	254	Q
betaE2		256	I	257	L	255	V
betaE2		257	D	258	E	256	D
		258	I	259	L	257	I
		259	N	260	D	258	P
		260	V	261	V	259	M
		261	G	262	G	260	E
		262	F	263	F	261	N
		263	D	264	S	262	G
		264	S	265	T	263	V
		265	G	266	G	264	G
		266	I	267	A	265	G
		267	D	268	D	266	N
	pad	268	R	269	Q	267	S
	Gbg	269	I	270	L	268	I
	pad	270	F	271	F	269	F
		271	L	272	L	270	L
		272	V	273	V	271	V
		273	S	274	S	272	A
		274	P	275	P	273	P
betaG		275	I	276	L	274	L
betaG		276	T	277	T	275	I
betaG		277	I	278	I	276	I
betaG		278	V	279	C	277	Y
betaG		279	H	280	H	278	H
betaG		280	E	281	V	279	V
		281	I	282	I	280	I
		282	D	283	D	281	D
		283	E	284	A	282	A
		284	D	285	K	283	N
		285	S	286	S	284	S
		286	P	287	P	285	P
		287	L	288	F	286	L
		288	Y	289	Y	287	Y
		289	D	290	D	288	D
		290	L	291	L	289	L
alphaG		291	S	292	S	290	A
alphaG		292	K	293	Q	291	P
alphaG		293	Q	294	R	292	S
alphaG		294	D	295	S	293	D
alphaG		295	I	296	M	294	L
alphaG						295	H
alphaG		296	D	297	Q	296	H
alphaG		297	N	298	T	297	H
		298	A	299	E	298	Q
		299	D	300	Q	299	D
		300	F	301	F	300	L
betaH		301	E	302	E	301	E
betaH		302	I	303	V	302	I
betaH		303	V	304	V	303	I
betaH		304	V	305	V	304	V
betaH		305	I	306	I	305	I
betaH		306	L	307	L	306	L
betaH		307	E	308	E	307	E
betaH		308	G	309	G	308	G
betaH		309	M	310	I	309	V
		310	V	311	V	310	V
		311	E	312	E	311	E
		312	A	313	T	312	T
		313	T	314	T	313	T
		314	A	315	G	314	G
		315	M	316	M	315	I
betaI		316	T	317	T	316	T
betaI		317	T	318	C	317	T
betaI		318	Q	319	Q	318	Q
betaI		319	C	320	A	319	A
betaI		320	R	321	R	320	R
betaI		321	S	322	T	321	T
betaI		322	S	323	S	322	S
betaI		323	Y	324	Y	323	Y
betaI		324	L	325	T	324	L
		325	A	326	E	325	A
		326	N	327	D	326	D
		327	E	328	E	327	E
betaJ		328	I	329	V	328	I
betaJ		329	L	330	L	329	L

betaJ		330	W	331	W	330	W
		331	G	332	G	331	G
betaK	pad	332	H	333	H	332	Q
betaK	Gbg	333	R	334	R	333	R
betaK	pad	334	Y	335	F	334	F
	Gbg	335	E	336	F	335	V
	Gbg	336	P	337	P	336	P
	pad	337	V	338	V	337	I
betaL	Gbg	338	L	339	I	338	V
betaL	pad	339	F	340	S	339	A
betaL	Gbg	340	E	341	L	340	E
betaL	Gbg	341	E	342	E	341	E
	Gbg	342	K	343	E	342	D
	pad	343	H	344	G	343	G
betaM	pad	344	Y	345	F	344	R
betaM	Gbg	345	Y	346	F	345	Y
betaM	pad	346	K	347	K	346	S
betaM	pad	347	V	348	V	347	V
betaM	pad	348	D	349	D	348	D
	ATP2	349	Y	350	Y	349	Y
	pad	350	S	351	S	350	S
	pad	351	R	352	Q	351	K
	ATP2	352	F	353	F	352	F
	ATP2	353	H	354	H	353	G
	pad	354	K	355	A	354	N
betaN	pad	355	T	356	T	355	T
betaN	Gbg	356	Y	357	F	356	I
betaN	Gbg	357	E	358	E	357	K
	Gbg	358	V	359	V	358	V
	pad	359	P			359	P
		360	N	360	P		
		361	T	361	T	360	T
		362	P	362	P	361	P
		363	L	363	P	362	L
		364	C	364	Y	363	C
		365	S	365	S	364	T
alphaH		366	A	366	V	365	A
alphaH		367	R	367	K	366	R
alphaH		368	D	368	E	367	Q
alphaH		369	L	369	Q	368	L
alphaH		370	A	370	E	369	D
alphaH		371	E	371	E	370	E
alphaH		372	K	372	M	371	D
alphaH		373	K	373	L	372	H
alphaH		374	Y	374	L	373	S
alphaH		375	I	375	M	374	L
alphaH		376	L	376	S	375	L
alphaH		377	S	377	S		
		378	N	378	P		
		379	A	379	L		
		380	N	380	I		
		381	S	381	A		
		382	F	382	P		
		383	C	383	A		
		384	Y	384	I		
		385	E	385	T	376	E
		386	N	386	N	377	A
		387	E	387	S	378	L
		388	V	388	K	379	T
		389	A	389	E	380	L
		390	L	390	R	381	A
		391	T	391	H	382	S
		392	S	392	N	383	A
		393	K	393	S	384	R
		394	E	394	V	385	G
		395	E	395	E	386	P
		396	E	396	C	387	L
		397	E	397	L	388	R
		398	D	398	D	389	K
		399	S	399	G	390	R
		400	E	400	L	391	S
		401	N	401	D	392	V
		402	G	402	D	393	P
		403	V	403	I	394	M
		404	P	404	S	395	A
		405	E	405	T	396	K
		406	S	406	K	397	A
		407	T	407	L	398	K
		408	S	408	P	399	P
		409	T	409	S	400	K
		410	D	410	K	401	F
		411	S	411	L	402	S
		412	P	412	Q	403	I
		413	P	413	K	404	S
		414	G	414	I	405	P
		415	I	415	T	406	D

		416	D	416	G	407	S
		417	L	417	R	408	L
		418	H	418	E	409	S
		419	N	419	D		
		420	Q	420	F		
		421	A	421	P		
		422	S	422	K		
		423	V	423	K		
		424	P	424	L		
		425	L	425	L		
		426	E	426	R		
		427	P	427	M		
		428	R	428	S		
		429	P	429	S		
		430	L	430	T		
		431	R	431	T		
		432	R	432	S		
		433	E	433	E		
		434	S	434	K		
		435	E	435	A		
		436	I	436	Y		
		437		437	S		
		438		438	L		
		439		439	G		
		440		440	D		
		441		441	L		
		442		442	P		
				443	M		
				444	K		
				445	L		
				446	Q		
				447	R		
				448	I		
				449	S		
				450	S		
				451	V		
				452	P		
				453	G		
				454	N		
				455	S		
				456	E		
				457	E		
				458	K		
				459	L		
				460	V		
				461	S		
				462	K		
				463	T		
				464	T		
				465	K		
				466	M		
				467	L		
				468	S		
				469	D		
				470	P		
				471	M		
				472	S		
				473	Q		
				474	S		
				475	V		
				476	A		
				477	D		
				478	L		
				479	P		
				480	P		
				481	K		
				482	L		
				483	Q		
				484	K		
				485	M		
				486	A		
				487	G		
				488	G		
				489	P		
				490	T		
				491	R		
				492	M		
				493	E		
				494	G		
				495	N		
				496	L		
				497	P		
				498	A		
				499	K		
				500	L		
				501	R		

				502	K		
				503	M		
				504	N		
				505	S		
				506	D		
				507	R		
				508	F		
				509	T		
