# Supplementary material for: Probing ion channel functional architecture and domain recombination compatibility by massively parallel domain insertion profiling
Source: Nat Commun. 2021 Dec 8;12:7114. doi: 10.1038/s41467-021-27342-0 (PMC8654947; doi:10.1038/s41467-021-27342-0)
Supplement: Supplementary file 2 — Description of Additional Supplementary Files [file 41467_2021_27342_MOESM2_ESM.pdf]

Description of Additional Supplementary Files

Supplementary Data 1: Inserted Motifs nucleotide sequences.

Supplementary Data 2: Recipient channels nucleotide sequences.

Supplementary Data 3: Processed source data (z-scored surface trafficking fitness).

Supplementary Data 4: Calculated Recipient Properties.

Supplementary Data 5: Calculated Inserted Motifs Properties.

Supplementary Data 6: R Markdown script and source data.

Supplementary Data 7: Oligonucleotide Sequences.
