# Supplementary material for: Probing ion channel functional architecture and domain recombination compatibility by massively parallel domain insertion profiling
Source: Nat Commun. 2021 Dec 8;12:7114. doi: 10.1038/s41467-021-27342-0 (PMC8654947; doi:10.1038/s41467-021-27342-0)
Supplement: Supplementary file 8 — Supplementary Data 6 [file 41467_2021_27342_MOESM8_ESM.zip › Supplementary_Data_6.nb.html]

Coyote-Maestas et. al (2021) Nature Communications Source Data


Code 

- Show All Code
- Hide All Code
- Download Rmd

# Coyote-Maestas et. al (2021) Nature Communications Source Data

This is an R Markdown Notebook. When you execute code within the notebook, the results appear beneath the code.

Try executing this chunk by clicking the *Run* button within the chunk or by placing your cursor inside it and pressing *Cmd+Shift+Enter*.


#setup


```
library(reshape2)
library(plyr)
library(dplyr)
library(tidyr)
library(ggplot2)
library(ComplexHeatmap)
library(ggpubr)
library(randomForest)
library(iml)
library(RFLPtools)
library(corrplot)
library(NbClust)
library(lsa)
library(circlize)
library(plyr)
library(uwot)
library(openxlsx)
library(scico)
library(yaImpute)

range01 = function(x, remove.na = F){
  if(remove.na){
    (x-min(x, na.rm = T))/(max(x, na.rm = T)-min(x, na.rm = T))
  }
  else{
    (x-min(x))/(max(x)-min(x))
  }
}
```


#Download data from Zenodo This requires inborutils (https://github.com/inbo/inborutils) Note that the download is very large (3.16GB).


```
knitr::opts_knit$set(root.dir = './') #set to correct working directory
```


#Alternative: load a local copy of the data Download the data from Zenodo (https://doi.org/10.5281/zenodo.5683566) manually Move the datafile (Supplemental\_Data\_6.RData) in the path Uncomment the line below


```
#uncomment this line to load a local copy of the date
#load('Supplemental_Data_6.RData')
```


#Kir2.1 Large Dataset ; merge with calculated properties


```
#remove any duplicates
d = unique(kir21_large_dataset)

#remove any NAs
d_complete = d[complete.cases(d$normalized_dp),]
#replace outliers above or below
d_complete[d_complete$normalized_dp>5,'normalized_dp'] = 5
d_complete[d_complete$normalized_dp<(-5),'normalized_dp'] = (-5)

#remove domains and positions missing 80% data
perm_pivot = dcast(d_complete,position~domain,value.var='normalized_dp')
perm_pivot_plot = perm_pivot[rowMeans(is.na(perm_pivot)) <= .8,]
perm_pivot_plot = perm_pivot_plot[,colMeans(is.na(perm_pivot_plot)) <= .5]

perm_plot_melt = melt(perm_pivot_plot,id.vars='position')
perm_plot_melt = na.omit(perm_plot_melt)
colnames(perm_plot_melt) = c('position','domain','permissibility')

#merge with domain annotations
annotated_kir21 = merge(perm_plot_melt,domain_annotations)

#QC on completeness of groups
annotated_only_annotations_kir21 = annotated_kir21
annotated_only_annotations_kir21$permissibility = NULL
annotated_only_annotations_kir21$position = NULL
annotated_only_annotations_kir21 = unique(annotated_only_annotations_kir21[,1:8])

#merge with Kir architecture annotation
annotated_kir21 = merge(domain_arch_key,annotated_kir21)

#merge calculated properties
#just sequence
kir21_seq_dom = merge(annotated_kir21,domain_sequence_feat)
kir21_seq_dom_no_annot = data.frame(
  domain = kir21_seq_dom$domain,
  permissibility = kir21_seq_dom$permissibility,kir21_seq_dom[,24:293])

#merge with calculated structural properties
kir21_struc_dom = merge(annotated_kir21,domain_structural_feat)

#merge with residue contact map (Arpeggio)
count_1 =  kir21_arpeggio_contacts %>% count(ResNum1)
colnames(count_1)<-c('structure','count_1')
count_2 =  kir21_arpeggio_contacts %>% count(ResNum2)
colnames(count_2)<-c('structure','count_2')
count_all = merge(count_1,count_2,all=TRUE)
count_all[is.na(count_all)]<-0
count_all = data.frame(
  structure=count_all$structure,
  contacts = count_all$count_1+count_all$count_2)
count_all = data.frame(
  structure=count_all$structure,
  contacts=count_all$contacts,
  contact_density=count_all$contacts/330)
kir21_contacts = merge(annotated_kir21,count_all,all=TRUE)
```


#Figure 1b-e


```
#unmelt domains#####
perm_pivot<-dcast(d_complete,position~domain,value.var='normalized_dp')

#count number of missing in positions and domains
perm_miss_pos = data.frame(position=seq(1:nrow(perm_pivot)),missing=rowMeans(is.na(perm_pivot)))
perm_miss_dom = data.frame(domain=seq(1:ncol(perm_pivot)),missing=colMeans(is.na(perm_pivot)))

#plot number of missing
gg_miss_pos_scatter = ggplot(perm_miss_pos,aes(x=position,y=missing))+geom_point()+
  labs(title='Missing Amino Acid Positions',x='Positions',y='missing percentage')+
  theme_classic()
gg_miss_domain_scatter = ggplot(perm_miss_dom,aes(x=domain,y=missing))+
  geom_point()+labs(title='Missing Domains',x='Domains',y='missing percentage')+
  theme_classic()
gg_miss_pos_density<-ggplot(perm_miss_pos,aes(x=missing))+geom_density()+
  theme_classic()
gg_miss_dom_density<-ggplot(perm_miss_dom,aes(x=missing))+geom_density()+
  theme_classic()

ggarrange(gg_miss_pos_scatter, gg_miss_domain_scatter, gg_miss_pos_density, gg_miss_dom_density, 
          ncol = 2, nrow = 2)
```


#Figure 2a


```
#Start calculating group means
#remove incomplete data (domains with 50% missing positions and positions with 80% missing domains)
perm_pivot_qc = perm_pivot[rowMeans(is.na(perm_pivot)) <= .8,]
perm_pivot_qc = perm_pivot_qc[,colMeans(is.na(perm_pivot)) <= .8] 

#scale data prior to distance measure
perm_scaled = scale(perm_pivot_qc[,-1],center=F)

#replace NAs with zeros
perm_pivot_zeros = perm_scaled
perm_pivot_zeros[is.na(perm_scaled)] = 0

#calculate cosine similarity
perm_matrix = data.matrix(perm_pivot_zeros)
cosine_sim = cosine(perm_matrix)

#convert similarity to distance matrix
cosine_distance = sim2dist(cosine_sim, maxSim = 1)

#cluster based on cosine similarity
res.hc_dom_cos = hclust(cosine_distance,  method = "ward.D2")

#calculate cosine similarity across positions
cosine_sim = cosine(t(perm_matrix))

#convert similarity to distance matrix
cosine_distance= sim2dist(cosine_sim, maxSim = 1)

#cluster based on cosine similarity
res.hc_pos_cos <- hclust(cosine_distance,  method = "ward.D2")


col_fun1 = colorRamp2(c(-1, 0, 1), c("#377EB8", "white", "#E41A1C"))
Heatmap(t(perm_pivot_qc[,-1]),
        col = col_fun1,
        cluster_rows = res.hc_dom_cos,
        column_title_gp = gpar(fontsize = 14), 
        cluster_columns = FALSE,
        row_dend_width = unit(20, "mm"),
        row_names_gp = gpar(fontsize = 2),
        column_names_gp = gpar(fontsize = 2),
        heatmap_legend_param = list(title = 'z-scored fitness'))
```


#Supplementary Figure 1


```
perm_pivot_se = dcast(d_complete,position~domain,value.var='se_dp')
perm_pivot_se_qc = perm_pivot_se[rowMeans(is.na(perm_pivot)) <= .8,]
perm_pivot_se_qc = perm_pivot_se_qc[,colMeans(is.na(perm_pivot)) <= .8]

col_fun2 = colorRamp2(seq(0,2, by = 0.2), 
                                scico(11, palette = 'buda', direction = -1))
Heatmap(t(perm_pivot_se_qc[,-1]),
        col = col_fun2,
        cluster_rows = res.hc_dom_cos,
        column_title_gp = gpar(fontsize = 14), 
        cluster_columns = FALSE,
        row_dend_width = unit(20, "mm"),
        row_names_gp = gpar(fontsize = 2),
        column_names_gp = gpar(fontsize = 2),
        heatmap_legend_param = list(title = 'fitness standard error'))
```


#Figure 2c


```
ggplot(d_complete, aes(x=normalized_dp)) + geom_histogram(bins = 200, fill = 'black') + xlab('z-scored fitness')
```


#Figure 2d


```
#productive insertions
prod_ins$location = factor(prod_ins$location, levels = c('N-term','Middle','C-term'))
ggplot(prod_ins, 
       aes(x=location, y=per_prod_ins, fill=as.factor(type))) + 
  geom_col(position = 'dodge') +
  ylab('fraction productive insertions')
```


#Supplementary Figure 3


#Figure 3c-h


```
clusters_props$dom_hier_3 = as.factor(clusters_props$dom_hier_3)
clusters_props$dom_hier_3 = revalue(clusters_props$dom_hier_3,
                                          c('1' = 'structured_motif',
                                            '2' = 'unstructured_motif',
                                            '3' = 'hydrophobic_motif'))
my_comparisons = list(
  c('structured_motif', 'unstructured_motif'), 
  c('unstructured_motif', 'hydrophobic_motif'), 
  c('structured_motif', 'hydrophobic_motif')
  )

# box plots with pairwise p-values
length_boxplot = ggboxplot(clusters_props, x = "dom_hier_3", y = "domain_length",
                          color = "dom_hier_3", palette = "jco") + 
  font("xy.text", size = 7) +
  xlab('Cluster#') +
  stat_compare_means(comparisons = my_comparisons, method='wilcox.test')

hydrophob_boxplot = ggboxplot(clusters_props, x = "dom_hier_3", y = "d_AA_hydrophob_mean",
                           color = "dom_hier_3", palette = "jco") + 
  font("xy.text", size = 7) +
  xlab('Cluster#') +
  stat_compare_means(comparisons = my_comparisons, method='wilcox.test') 


negat_boxplot = ggboxplot(clusters_props, x = "dom_hier_3", y = "d_AA_negat_mean",
                              color = "dom_hier_3", palette = "jco") +
  font("xy.text", size = 7) +
  xlab('Cluster#') +
  stat_compare_means(comparisons = my_comparisons, method='wilcox.test')


figure_boxplots_props = ggarrange(length_boxplot, hydrophob_boxplot, negat_boxplot,
                                   labels = c("A", "B", "C"),
                                   ncol = 3, nrow = 1)

clusters_props$domain_cluster = ifelse(clusters_props$group == 'struc_domain', 'struc_dom', 'other')

domain_length_dens = ggplot(clusters_props,aes(x=domain_length,fill=domain_cluster))+
  geom_histogram(aes(y=0.5*..density..),position='identity',alpha=0.4)+
  theme_classic() +
  theme(legend.position = "none")

domain_negat_dens = ggplot(clusters_props,aes(x=d_AA_negat_mean,fill=domain_cluster))+
  geom_histogram(aes(y=0.5*..density..),position='identity',alpha=0.4)+
  theme_classic() +
  theme(legend.position = "none")

domain_hydrophob_dens = ggplot(clusters_props,aes(x=d_AA_hydrophob_mean,fill=domain_cluster))+
  geom_histogram(aes(y=0.5*..density..),position='identity',alpha=0.4)+
  theme_classic() +
  theme(legend.position = "none")

figure_dens_props = ggarrange(domain_length_dens, domain_negat_dens, domain_hydrophob_dens,
                               labels = c("D", "E", "F"),
                               ncol = 3, nrow = 1)

all_props = ggarrange(length_boxplot, hydrophob_boxplot, negat_boxplot,domain_length_dens, domain_negat_dens, domain_hydrophob_dens,
                      labels = c('C','D','E','F','G','H'),
                      ncol = 3, nrow = 2)
all_props
```


#Figure 4m


```
#all position based properties mean w/o weakly correlated
rownames(receiving_pos_combined) = receiving_pos_combined$rowname
receiving_pos_combined = receiving_pos_combined[,2:424]
Heatmap(receiving_pos_combined,clustering_distance_rows = "euclidean",cluster_columns =FALSE,
        row_names_gp = gpar(fontsize = 5),
        column_names_gp = gpar(fontsize = 2),
        heatmap_legend_param = list(title = 'Spearman correlation'))
```


#Supplementary Figure 4


```
Heatmap(receiving_pos_combined,clustering_distance_rows = 'euclidean',
        clustering_distance_columns = 'euclidean',
        heatmap_legend_param = list(title = 'Spearman correlation'),
        row_names_gp = gpar(fontsize = 5),
        column_names_gp = gpar(fontsize = 2))
```


#Figure 4a,b,i-k


```
recipient_stats = kir21_contacts %>%
  group_by(Kir2_1_Resid) %>%
  summarise(var_perm = sd(permissibility), mean_perm=median(permissibility),
            rmsf_3jyc=mean(rmsf_3jyc),cdegree=mean(contact_density))

recipient_stats = recipient_stats[complete.cases(recipient_stats),]

relationship_recip_rmsf_3jyc = ggplot(recipient_stats, aes(x=rmsf_3jyc, y=mean_perm)) +
  geom_point() + theme_classic() + geom_smooth(method = "loess", col='red', span=0.8)

relationship_recip_contacts = ggplot(recipient_stats, aes(x=cdegree, y=mean_perm)) +
  geom_point() + theme_classic() + geom_smooth(method = "loess", col='red', span=0.8)

donor_stats_struc = kir21_struc_dom %>%
  group_by(PDB) %>%
  summarise(var_perm = sd(permissibility), mean_perm = median(permissibility),
            d_nc_dist = mean(d_nc_dist))

relationship_nc_dist = ggplot(donor_stats_struc, aes(x = d_nc_dist, y = mean_perm)) +
  geom_point() + theme_classic() + geom_smooth(method = "loess", col='red')

donor_stats_seq = kir21_seq_dom %>%
  group_by(PDB) %>%
  summarise(var_perm = sd(permissibility), mean_perm = median(permissibility),
            d_AA_hydrophob_mean = mean(d_AA_hydrophob_mean),
            domain_length = mean(domain_length))

relationship_domain_length = ggplot(donor_stats_seq, aes(x = domain_length, y = mean_perm)) +
  geom_point() + theme_classic() + geom_smooth(method = "loess", col='red')

relationship_d_hydrophob = ggplot(donor_stats_seq, aes(x = d_AA_hydrophob_mean, y = mean_perm)) +
  geom_point() + theme_classic() + geom_smooth(method = "loess", col='red')

figure_relationships = ggarrange(relationship_recip_rmsf_3jyc, relationship_recip_contacts, relationship_domain_length, relationship_nc_dist, relationship_d_hydrophob,
                                 labels = c("a", "b", "i","j", "k"),
                                 ncol = 2, nrow = 3)
figure_relationships
```


#Figure 3a


```
set.seed(42)
perm_pivot_zeros = perm_pivot_plot
perm_pivot_zeros[is.na(perm_pivot_zeros)] = 0

df.umap = umap(perm_pivot_zeros[,-1], 
               metric = 'cosine',
               n_epochs = 50,
               n_neighbors = 50,
               nn_method = 'annoy',
               n_trees = 20
)


scores_kir21_large = data.frame(df.umap) # PC score matrix
scores_kir21_large$group = as.factor(NbClust(scores_kir21_large, 
                                             method = 'ward.D2')$Best.partition)
```


```
scores_kir21_large$position = perm_pivot_zeros$position
scores_kir21_large$gene = rep('Kir21', nrow(scores_kir21_large))

scores_kir21_large$group = revalue(scores_kir21_large$group, 
                                   c('1' = 'high',
                                     '2' = 'low',
                                     '3' = 'intermediate'))

plot_kir21_umap = ggplot(scores_kir21_large, aes(X1,X2, color = as.factor(group))) +
  geom_point() +
  xlab('UMAP1') + ylab('UMAP2') + ggtitle('Kir2.1 large dataset')
plot_kir21_umap
```


#Supplementary Figure 5c,f


```
final_model_perf = data.frame(
  'trees' = seq(1,500,by = 1),
  'Error' = plot(rf_kir21_just_seq_train)
)
```


```
p1 = ggplot(final_model_perf, aes(trees, Error)) + geom_line() + theme_classic()
p2 = plot(imp_cutoff)

final_model_perf = ggarrange(p1,p2,ncol = 2, nrow = 1)
final_model_perf
```


#Supplementary Figure 6


```
perm_heatmap = data.frame(permissibility=predictions_df_useful[,2],predictions_df_useful[,4:5])

p1 = ggplot(predictions_df_useful, aes(x = permissibility)) + geom_density() +
  ggtitle('Actual') + theme_classic() + xlab('fitness')
p2 = ggplot(predictions_df_useful, aes(x = predictions_test)) + geom_density() +
  ggtitle('Predicted') + theme_classic() + xlab('fitness')
p3 = ggplot(predictions_df_useful, aes(x = pred_actual_test)) + geom_density() +
  ggtitle('Actual minus Predicted') + theme_classic() + xlab('fitness difference')
p4 = ggplot(predictions_df_useful, aes(permissibility,predictions_test)) + geom_point() + theme_classic() +
  xlab('Actual') + ylab('Predicted')

SuppFig6 = ggarrange(p1,p2,p3,p4,
                     ncol = 4, nrow = 1,
                     labels = c('a','b','c','d'))

SuppFig6
```


#Supplementary Figure 6e-h


```
perm_heatmap_dcast = dcast(predictions_df_useful,position~domain,value.var='permissibility')
rownames(perm_heatmap_dcast) = perm_heatmap_dcast[,1]
perm_heatmap_dcast = t(perm_heatmap_dcast[,-1])
perm_pos_mean = colMeans(perm_heatmap_dcast,na.rm=TRUE)
perm_dom_mean = rowMeans(perm_heatmap_dcast,na.rm=TRUE)
pred_heatmap_dcast = dcast(predictions_df_useful,position~domain,value.var='predictions_test')
rownames(pred_heatmap_dcast) = pred_heatmap_dcast[,1]
pred_heatmap_dcast = t(pred_heatmap_dcast[,-1])
pred_pos_mean = colMeans(pred_heatmap_dcast,na.rm=TRUE)
pred_dom_mean = rowMeans(pred_heatmap_dcast,na.rm=TRUE)
perm_pred_heatmap_dcast = dcast(predictions_df_useful,position~domain,value.var='pred_actual_test')
rownames(perm_pred_heatmap_dcast) = perm_pred_heatmap_dcast[,1]
perm_pred_heatmap_dcast = t(perm_pred_heatmap_dcast[,-1])
perm_pred_pos_mean = colMeans(perm_pred_heatmap_dcast,na.rm=TRUE)
perm_pred_dom_mean = rowMeans(perm_pred_heatmap_dcast,na.rm=TRUE)


domains_mean = data.frame(perm_dom=perm_dom_mean,pred_dom=pred_dom_mean,perm_pred_dom=perm_pred_dom_mean)
domains_mean = data.frame(rownames(domains_mean),domains_mean)
colnames(domains_mean) = c('domain','perm_domain','pred_domain','perm_pred_domain')
domains_mean_melt = melt(domains_mean)

domains_mean_melt_order = domains_mean[order(domains_mean$perm_domain),]
domains_mean_melt_order$domain_num = seq_along(domains_mean_melt_order$domain)

p1 = ggplot(domains_mean_melt_order) + 
  geom_line(aes(x=domain_num, y=perm_domain), stat = 'identity', color = '#F8766D') +
  geom_line(aes(x=domain_num, y=perm_pred_domain), stat = 'identity', color = '#619CFF') +
  geom_line(aes(x=domain_num, y=pred_domain), stat = 'identity', color = '#00BA38') +
  xlab('Motif') + ylab('Fitness') +
  theme_classic2()

lm_eqn = function(df){
  m = lm(pred_domain ~ perm_domain, df);
  eq = substitute(italic(y) == a + b %.% italic(x)*","~~italic(r)^2~"="~r2, 
                   list(a = format(unname(coef(m)[1]), digits = 2),
                        b = format(unname(coef(m)[2]), digits = 2),
                        r2 = format(summary(m)$r.squared, digits = 3)))
  as.character(as.expression(eq));
}

p2 = ggplot(domains_mean, aes(x=perm_domain, y=pred_domain)) + 
  geom_point() + 
  geom_smooth(method = "lm", se=FALSE, color="black") +
  geom_text(x = -0.8, y = -0.4, label = lm_eqn(domains_mean), parse = TRUE) +
  coord_fixed() +
  ylim(-1.25,0) +
  xlim(-1.25,0) +
  theme_classic2()

positions_mean = data.frame(perm_pos=perm_pos_mean,pred_pos=pred_pos_mean,perm_pred=perm_pred_pos_mean)
positions_mean = data.frame(rownames(positions_mean),positions_mean)
colnames(positions_mean) = c('positions','perm_positions','pred_positions','perm_pred_positions')
positions_mean_melt = melt(positions_mean)
positions_mean_melt$positions = as.numeric(as.character(positions_mean_melt$positions))

p3 = ggplot(positions_mean_melt, aes(x=positions, y=value, group=variable, color=variable)) + geom_line() +
  scale_x_continuous(breaks = seq(50,400, by = 50)) +
  theme(axis.text.x = element_text(angle = 90, hjust=1)) + 
  xlab('positions') + ylab('Fitness') +
  theme_classic2()

lm_eqn = function(df){
  m = lm(pred_positions ~ perm_positions, df);
  eq = substitute(italic(y) == a + b %.% italic(x)*","~~italic(r)^2~"="~r2, 
                   list(a = format(unname(coef(m)[1]), digits = 2),
                        b = format(unname(coef(m)[2]), digits = 2),
                        r2 = format(summary(m)$r.squared, digits = 3)))
  as.character(as.expression(eq));
}

p4 = ggplot(positions_mean, aes(x=perm_positions, y=pred_positions)) + 
  geom_point() + 
  geom_smooth(method = "lm", se=FALSE, color="black") +
  geom_text(x = -0.8, y = 0.3, label = lm_eqn(positions_mean), parse = TRUE) +
  coord_fixed() +
  theme_classic2()

ggarrange(p1,p2,p3,p4,
          ncol = 2, nrow = 2,
          labels = c('e','f','g','h'))
```


#Figure 5a


```
feature_importance = imp_cutoff$results
feature_order = c('beta_start_11',
                  'contact_density',
                  'd_AA_vol_n_7',
                  'd_AA_hydrophob_mean',
                  'domain_length'
                  ,'d_AA_negat_mean',
                  'phi_mid_11',
                  'polarsasa_start_11',
                  'rmsf_3jyc',
                  'stiffness_mid_11')
feature_importance$feature = factor(feature_importance$feature,levels=feature_order)

#ggplot(feature_importance, aes(x = feature, y = importance)) + 
#  geom_col(fill='#008440')+theme_classic() +
#  theme(axis.ticks = element_blank(), axis.text.x = element_text(angle = 90, hjust=1))

ggplot(data = feature_importance, aes(x = feature, y = 1)) + 
  geom_tile(aes(fill = importance), color = "white", size = 1) + 
  scale_fill_gradient(low = "gray95", high = "tomato", limits = c(1, max(feature_importance$importance))) +
  theme(axis.text.x = element_text(angle = 90)) +
  ylab('Importance')
```


#Figure 5b-e


```
ale_contact_density = FeatureEffect$new(predictor_cutoff, feature = "contact_density")
ale_contact_density_plot = ale_contact_density$plot() + theme_classic()

ale_d_AA_hydrophob_mean = FeatureEffect$new(predictor_cutoff, feature = "d_AA_hydrophob_mean")
ale_d_AA_hydrophob_mean_plot = ale_d_AA_hydrophob_mean$plot() + theme_classic()

ale_domain_length = FeatureEffect$new(predictor_cutoff, feature = "domain_length")
ale_domain_length_plot = ale_domain_length$plot() + theme_classic()

ale_rmsf_3jyc = FeatureEffect$new(predictor_cutoff, feature = "rmsf_3jyc")
ale_rmsf_3jyc_plot = ale_rmsf_3jyc$plot() + theme_classic()

figure_ALEplot = ggarrange(ale_contact_density_plot, 
                           ale_d_AA_hydrophob_mean_plot,
                           ale_domain_length_plot,
                           ale_rmsf_3jyc_plot,
                           labels = c('b','c','d','e'),
                           ncol = 4, nrow = 1)
figure_ALEplot
```


#Supplementary Figure 7


```
ale_d_AA_negat_mean = FeatureEffect$new(predictor_cutoff, feature = "d_AA_negat_mean")
ale_d_AA_negat_mean_plot = ale_d_AA_negat_mean$plot() + theme_classic()

ale_d_AA_vol_n_7 = FeatureEffect$new(predictor_cutoff, feature = "d_AA_vol_n_7")
ale_d_AA_vol_n_7_plot = ale_d_AA_vol_n_7$plot() + theme_classic()

ale_polarsasa_start_11 = FeatureEffect$new(predictor_cutoff, feature = "polarsasa_start_11")
ale_polarsasa_start_11_plot = ale_polarsasa_start_11$plot()+theme_classic()

ale_phi_mid_11 = FeatureEffect$new(predictor_cutoff, feature = "phi_mid_11")
ale_phi_mid_11_plot = ale_phi_mid_11$plot() + theme_classic()

ale_stiffness_mid_11 = FeatureEffect$new(predictor_cutoff, feature = "stiffness_mid_11")
ale_stiffness_mid_11_plot = ale_stiffness_mid_11$plot() + theme_classic()

ale_beta_start_11 = FeatureEffect$new(predictor_cutoff, feature = "beta_start_11")
ale_beta_start_11_plot = ale_beta_start_11$plot() + theme_classic()

figure_ALEplot_supp = ggpubr::ggarrange(ale_d_AA_hydrophob_mean_plot, 
                                        ale_d_AA_negat_mean_plot,
                                        ale_rmsf_3jyc_plot,
                                        ale_phi_mid_11_plot,
                                        ale_contact_density_plot,
                                        ale_beta_start_11_plot,
                                        ale_stiffness_mid_11_plot,
                                        ale_domain_length_plot,
                                        ale_d_AA_vol_n_7_plot,
                                        ale_polarsasa_start_11_plot,
                                        labels = c('a','b','c','d',
                                                   'e','f','g','h','i','j'),
                                        ncol = 3, nrow = 4)
figure_ALEplot_supp
```


#Figure 6a


```
#order of plotting (overall on top)
feature_1_levels = c('d_AA_hydrophob_mean', 'd_AA_negat_mean', 'd_AA_vol_n_7', 'domain_length', 'beta_start_11',
                     'contact_density',  'phi_mid_11',  'polarsasa_start_11', 'rmsf_3jyc', 'stiffness_mid_11', 'overall')
feature_2_levels = c('overall','beta_start_11', 'contact_density', 'phi_mid_11', 'polarsasa_start_11', 'rmsf_3jyc',
                     'stiffness_mid_11', 'd_AA_hydrophob_mean', 'd_AA_negat_mean', 'd_AA_vol_n_7','domain_length')
interactions_heatmap$feature_1 = factor(interactions_heatmap$feature_1, levels = feature_1_levels)
interactions_heatmap$feature_2 = factor(interactions_heatmap$feature_2, levels = feature_2_levels)
ggplot(data = interactions_heatmap, aes(x = feature_2, y = feature_1)) + 
  geom_tile(aes(fill = interaction_strength), color = 'white', size = 1) + 
  scale_fill_gradient(low = "gray95", high = "tomato") + 
  xlab('Feature_2') + 
  theme_grey(base_size = 12) + 
  ggtitle('Interaction Strength Between Features') + 
  theme(axis.ticks = element_blank(), axis.text.x = element_text(angle = 90, hjust=1),
        panel.background = element_blank(), 
        plot.title = element_text(size = 12, colour = "gray50"))
```


```
NA
NA
```


#Figure 6b-d


```
#two way plots
ale_d_hydrophob_stiffness = FeatureEffect$new(predictor_cutoff, feature = c("d_AA_hydrophob_mean", "stiffness_mid_11"))
ale_d_hydrophob_stiffness_plot = ale_d_hydrophob_stiffness$plot() + 
  scale_fill_gradient2(high = '#b2182b', mid = '#f7f7f7', low = '#2166ac') +
  theme_classic()

ale_d_hydrophob_domain_length = FeatureEffect$new(predictor_cutoff, feature = c("d_AA_hydrophob_mean", "domain_length"))
ale_d_hydrophob_domain_length_plot = ale_d_hydrophob_domain_length$plot() + 
  scale_fill_gradient2(high = '#b2182b', mid = '#f7f7f7', low = '#2166ac') +
  theme_classic()

ale_phi_stiffness = FeatureEffect$new(predictor_cutoff, feature = c("phi_mid_11", "stiffness_mid_11"))
ale_phi_stiffness_plot = ale_phi_stiffness$plot() + 
  scale_fill_gradient2(high = '#b2182b', mid = '#f7f7f7', low = '#2166ac') +
  theme_classic()

figure6_twoway = ggarrange(ale_phi_stiffness_plot, 
                           ale_d_hydrophob_domain_length_plot,
                           ale_d_hydrophob_stiffness_plot,
                           labels = c('b','c','d'),
                           ncol = 3, nrow = 1)
figure6_twoway
```


#Supplementary Figure 11


```
ale_polarsasa_stiffness = FeatureEffect$new(predictor_cutoff, feature = c("polarsasa_start_11", "stiffness_mid_11"))
ale_polarsasa_stiffness_plot = ale_polarsasa_stiffness$plot() + 
  scale_fill_gradient2(high = '#b2182b', mid = '#f7f7f7', low = '#2166ac') +
  theme_classic()

suppfigure11_twoway = ggarrange(ale_phi_stiffness_plot, 
                                ale_polarsasa_stiffness_plot,
                                labels = c('a','b'),
                                ncol = 2, nrow = 1)
suppfigure11_twoway
```


#Supplementary Figure 10-12


```
ale_d_hydrophob_cdegree = FeatureEffect$new(predictor_cutoff, feature = c("d_AA_hydrophob_mean", "contact_density"))
ale_d_hydrophob_cdegree_plot = ale_d_hydrophob_cdegree$plot() + 
  scale_fill_gradient2(high = '#b2182b', mid = '#f7f7f7', low = '#2166ac') +
  theme_classic()

ale_d_hydrophob_beta_start_11 = FeatureEffect$new(predictor_cutoff, feature = c("d_AA_hydrophob_mean", "beta_start_11"))
ale_d_hydrophob_beta_start_11_plot = ale_d_hydrophob_beta_start_11$plot() + 
  scale_fill_gradient2(high = '#b2182b', mid = '#f7f7f7', low = '#2166ac') +
  theme_classic()

ale_d_hydrophob_polarsasa_start_11 = FeatureEffect$new(predictor_cutoff, feature = c("d_AA_hydrophob_mean", "polarsasa_start_11"))
ale_d_hydrophob_polarsasa_start_11_plot = ale_d_hydrophob_polarsasa_start_11$plot() + 
  scale_fill_gradient2(high = '#b2182b', mid = '#f7f7f7', low = '#2166ac') +
  theme_classic()

ale_d_rmsf_3jyc_hydrophob = FeatureEffect$new(predictor_cutoff, feature = c("d_AA_hydrophob_mean", "rmsf_3jyc"))
ale_d_rmsf_3jyc_hydrophob_plot = ale_d_rmsf_3jyc_hydrophob$plot() + 
  scale_fill_gradient2(high = '#b2182b', mid = '#f7f7f7', low = '#2166ac') +
  theme_classic()

ale_d_vol_n_7_domain_length = FeatureEffect$new(predictor_cutoff, feature = c("d_AA_vol_n_7", "domain_length"))
ale_d_vol_n_7_domain_length_plot = ale_d_vol_n_7_domain_length$plot() + 
  scale_fill_gradient2(high = '#b2182b', mid = '#f7f7f7', low = '#2166ac') +
  theme_classic()

ale_d_hydrophob_domain_negat = FeatureEffect$new(predictor_cutoff, feature = c("d_AA_hydrophob_mean", "d_AA_negat_mean"))
ale_d_hydrophob_domain_negat_plot = ale_d_hydrophob_domain_negat$plot() + 
  scale_fill_gradient2(high = '#b2182b', mid = '#f7f7f7', low = '#2166ac') +
  theme_classic()

ale_d_stiffness_mid_11_domain_length <- FeatureEffect$new(predictor_cutoff, feature = c("stiffness_mid_11", "domain_length"))
ale_d_stiffness_mid_11_domain_length_plot = ale_d_stiffness_mid_11_domain_length$plot() + 
  scale_fill_gradient2(high = '#b2182b', mid = '#f7f7f7', low = '#2166ac') +
  theme_classic()

ale_d_negat_domain_length = FeatureEffect$new(predictor_cutoff, feature = c("d_AA_negat_mean", "domain_length"))
ale_d_negat_domain_length_plot = ale_d_negat_domain_length$plot() + 
  scale_fill_gradient2(high = '#b2182b', mid = '#f7f7f7', low = '#2166ac') +
  theme_classic()

ale_d_rmsf_3jyc_domain_length = FeatureEffect$new(predictor_cutoff, feature = c("rmsf_3jyc", "domain_length"))
ale_d_rmsf_3jyc_domain_length_plot = ale_d_rmsf_3jyc_domain_length$plot() + 
  scale_fill_gradient2(high = '#b2182b', mid = '#f7f7f7', low = '#2166ac') +
  theme_classic()


ale_d_cdegree_domain_length <- FeatureEffect$new(predictor_cutoff, feature = c("contact_density", "domain_length"))
ale_d_cdegree_domain_length_plot = ale_d_cdegree_domain_length$plot() + 
  scale_fill_gradient2(high = '#b2182b', mid = '#f7f7f7', low = '#2166ac') +
  theme_classic()

suppfigure10_twoway = ggarrange(ale_d_hydrophob_stiffness_plot,
                                ale_d_hydrophob_domain_length_plot,
                                ale_d_hydrophob_cdegree_plot,
                                ale_d_hydrophob_beta_start_11_plot,
                                ale_d_hydrophob_polarsasa_start_11_plot,
                                ale_d_rmsf_3jyc_hydrophob_plot,
                                ale_d_vol_n_7_domain_length_plot,
                                ale_d_hydrophob_domain_negat_plot,
                                ale_d_stiffness_mid_11_domain_length_plot,
                                ale_d_negat_domain_length_plot,
                                ale_d_rmsf_3jyc_domain_length_plot,
                                ale_d_cdegree_domain_length_plot,
                                labels = c('a','b','c','d','e','f',
                                           'g','h','i','j','k','l'),
                                ncol = 3, nrow = 4)

suppfigure10_twoway
```


#Supplementary Figure 2 & 12 for Kir21\_15D


```
#Kir21_15 based on structured motifs

z = dcast(kir21_15D, domain + gene + position ~ variable, fun.aggregate = function(x) mean(x, na.rm = T))
z[,'normalized_lowANDhigh'][z[,'normalized_lowANDhigh'] == 0] = NA

z$perm = rep(NA, nrow(z))

for (i in levels(z$gene)){
  idx = z$gene == i
  z[idx,'perm'] = range01(z[idx,'normalized_lowANDhigh'],remove.na = T)
}

SuppFig12_b = ggplot(z) +
  geom_tile(aes(x=position,y=domain,fill=normalized_lowANDhigh)) +
  scico::scale_fill_scico(palette = 'bilbao', na.value = 'cyan') +
  facet_grid(gene~.) +
  theme_classic() +
  ggtitle('Kir21')

kir21z = z

d = z[z$gene == 'Kir21',c('domain','position','perm')]

#remove domains and positions missing 80% data
perm_pivot = dcast(d,position~domain,value.var='perm')
perm_pivot_plot = perm_pivot[rowMeans(is.na(perm_pivot)) <= .8,]
perm_pivot_plot = perm_pivot_plot[,colMeans(is.na(perm_pivot_plot)) <= .5]

perm_plot_melt = melt(perm_pivot_plot,id.vars='position')
perm_plot_melt = na.omit(perm_plot_melt)
colnames(perm_plot_melt) = c('position','domain','permissibility')

perm_pivot_zeros = perm_pivot_plot
perm_pivot_zeros[is.na(perm_pivot_zeros)] = 0


df.umap = umap(perm_pivot_zeros[,-1], 
                     metric = 'cosine',
                     n_epochs = 500,
                     n_neighbors = 20,
                     nn_method = 'annoy',
                     n_trees = 100,
                     bandwidth = 0.8
)

scores_kir21_15D = data.frame(df.umap) # PC score matrix
scores_kir21_15D$group = NbClust::NbClust(scores_kir21_15D, method = 'ward.D2')$Best.partition
```


```
*** : The Hubert index is a graphical method of determining the number of clusters.
                In the plot of Hubert index, we seek a significant knee that corresponds to a 
                significant increase of the value of the measure i.e the significant peak in Hubert
                index second differences plot.
```


```
*** : The D index is a graphical method of determining the number of clusters. 
                In the plot of D index, we seek a significant knee (the significant peak in Dindex
                second differences plot) that corresponds to a significant increase of the value of
                the measure. 
 
******************************************************************* 
* Among all indices:                                                
* 2 proposed 2 as the best number of clusters 
* 9 proposed 3 as the best number of clusters 
* 1 proposed 4 as the best number of clusters 
* 3 proposed 5 as the best number of clusters 
* 3 proposed 11 as the best number of clusters 
* 1 proposed 12 as the best number of clusters 
* 4 proposed 15 as the best number of clusters 

                   ***** Conclusion *****                            
 
* According to the majority rule, the best number of clusters is  3 
 
 
*******************************************************************
```


```
scores_kir21_15D$gene = rep('Kir21', nrow(scores_kir21_15D))

SuppFig2_b = ggplot(scores_kir21_15D, aes(X1,X2, color = as.factor(group))) +
  geom_point() + xlab('UMAP1') + ylab('UMAP2') + ggtitle('Kir2.1 (based on structural motifs')
```


#Supplementary Figure 2 & 12 for Kir3.1


```
#Kir31_15D

z = dcast(kir31_15D, domain + gene + position ~ variable, fun.aggregate = function(x) mean(x, na.rm = T))
z[,'normalized_lowANDhigh'][z[,'normalized_lowANDhigh'] == 0] = NA

z$perm = rep(NA, nrow(z))

for (i in levels(z$gene)){
    idx = z$gene == i
    z[idx,'perm'] = range01(z[idx,'normalized_lowANDhigh'],remove.na = T)
}

z$gene = revalue(z$gene, c('Kir31-2'='Kir31','Kir31-3'='Kir31','Kir31-4'='Kir31','Kir31-5'='Kir31'))
kir31z = z

z = melt(kir31z, id.vars = c('gene','domain', 'position'))
z = dcast(z, domain + gene + position ~ variable, fun.aggregate = function(x) mean(x, na.rm = T))

SuppFig12_d = ggplot(z) +
  geom_tile(aes(x=position,y=domain,fill=perm)) +
  scico::scale_fill_scico(palette = 'bilbao', na.value = 'cyan') +
  facet_grid(gene~.) +
  theme_classic()+
  ggtitle('Kir31')

d = z[z$gene == 'Kir31',c('domain','position','perm')]


#remove domains and positions missing 80% data
perm_pivot = dcast(d,position~domain,value.var='perm')
perm_pivot_plot = perm_pivot[rowMeans(is.na(perm_pivot)) <= .8,]
perm_pivot_plot = perm_pivot_plot[,colMeans(is.na(perm_pivot_plot)) <= .5]

perm_plot_melt = melt(perm_pivot_plot,id.vars='position')
perm_plot_melt = na.omit(perm_plot_melt)
colnames(perm_plot_melt) = c('position','domain','permissibility')

perm_pivot_zeros = perm_pivot_plot
perm_pivot_zeros[is.na(perm_pivot_zeros)] = 0

#umap
df.umap = umap(perm_pivot_zeros[,-1], 
                     metric = 'euclidean',
                     n_epochs = 500,
                     n_neighbors = 20,
                     nn_method = 'annoy',
                     n_trees = 100,
                     bandwidth = 1
)


scores_Kir31 = data.frame(df.umap) # PC score matrix
scores_Kir31$group = NbClust(scores_Kir31, method = 'ward.D2', min.nc = 2)$Best.partition
```


```
*** : The Hubert index is a graphical method of determining the number of clusters.
                In the plot of Hubert index, we seek a significant knee that corresponds to a 
                significant increase of the value of the measure i.e the significant peak in Hubert
                index second differences plot.
```


```
*** : The D index is a graphical method of determining the number of clusters. 
                In the plot of D index, we seek a significant knee (the significant peak in Dindex
                second differences plot) that corresponds to a significant increase of the value of
                the measure. 
 
******************************************************************* 
* Among all indices:                                                
* 13 proposed 3 as the best number of clusters 
* 8 proposed 4 as the best number of clusters 
* 1 proposed 7 as the best number of clusters 
* 1 proposed 15 as the best number of clusters 

                   ***** Conclusion *****                            
 
* According to the majority rule, the best number of clusters is  3 
 
 
*******************************************************************
```


```
scores_Kir31$gene = rep('Kir31', nrow(scores_Kir31))

SuppFig2_c = ggplot(scores_Kir31, aes(X1,X2, color = as.factor(group))) +
  geom_point()  + xlab('UMAP1') + ylab('UMAP2') + ggtitle('Kir3.1')
```


#Supplementary Figure 2 & 12 for Asic1a


```
#load Asic1a####

#SuppFig12_f = ggplot(asic1_15D) +
#  geom_tile(aes(x=position,y=domain,fill=value)) +
#  scico::scale_fill_scico(palette = 'bilbao', na.value = 'cyan') +
#  facet_grid(gene~.) +
#  theme_classic() +
#  ggtitle('Asic1a')

z = dcast(asic1_15D, domain + gene + position ~ variable, fun.aggregate = function(x) mean(x, na.rm = T))
z[,'normalized_lowANDhigh'][z[,'normalized_lowANDhigh'] == 0] = NA

z$perm = rep(NA, nrow(z))


for (i in levels(z$gene)){
  idx = z$gene == i
  z[idx,'perm'] = range01(z[idx,'normalized_lowANDhigh'],remove.na = T)
}

SuppFig12_f = ggplot(z) +
  geom_tile(aes(x=position,y=domain,fill=normalized_lowANDhigh)) +
  scico::scale_fill_scico(palette = 'bilbao', na.value = 'cyan') +
  facet_grid(gene~.) +
  theme_classic() +
  ggtitle('Asic1a')

asic1az = z


#Asic1a####

d = z[z$gene == 'Asic1a',c('domain','position','perm')]


#remove domains and positions missing 80% data
perm_pivot<-dcast(d,position~domain,value.var='perm')
perm_pivot_plot<-perm_pivot[rowMeans(is.na(perm_pivot)) <= .8,]
perm_pivot_plot<-perm_pivot_plot[,colMeans(is.na(perm_pivot_plot)) <= .5]

perm_plot_melt<-melt(perm_pivot_plot,id.vars='position')
perm_plot_melt<-na.omit(perm_plot_melt)
colnames(perm_plot_melt)<-c('position','domain','permissibility')

perm_pivot_zeros = perm_pivot_plot
perm_pivot_zeros[is.na(perm_pivot_zeros)] = 0


df.umap = umap(perm_pivot_zeros[,-1], 
                     metric = 'euclidean',
                     n_epochs = 500,
                     n_neighbors = 20,
                     nn_method = 'annoy',
                     n_trees = 100,
                     bandwidth = 1
)


scores_Asic1a = data.frame(df.umap) # PC score matrix
scores_Asic1a$group = NbClust(scores_Asic1a, method = 'ward.D2', min.nc = 2)$Best.partition
```


```
*** : The Hubert index is a graphical method of determining the number of clusters.
                In the plot of Hubert index, we seek a significant knee that corresponds to a 
                significant increase of the value of the measure i.e the significant peak in Hubert
                index second differences plot.
```


```
*** : The D index is a graphical method of determining the number of clusters. 
                In the plot of D index, we seek a significant knee (the significant peak in Dindex
                second differences plot) that corresponds to a significant increase of the value of
                the measure. 
 
******************************************************************* 
* Among all indices:                                                
* 6 proposed 2 as the best number of clusters 
* 14 proposed 3 as the best number of clusters 
* 1 proposed 4 as the best number of clusters 
* 2 proposed 12 as the best number of clusters 
* 1 proposed 15 as the best number of clusters 

                   ***** Conclusion *****                            
 
* According to the majority rule, the best number of clusters is  3 
 
 
*******************************************************************
```


```
scores_Asic1a$gene = rep('Asic1a', nrow(scores_Asic1a))

SuppFig2_d = ggplot(scores_Asic1a, aes(X1,X2, color = as.factor(group))) +
  geom_point()  + xlab('UMAP1') + ylab('UMAP2') + ggtitle('Asic1a')
```


#Supplementary Figure 2 & 12 for P2X3


```
z = dcast(p2x3_15D, domain + gene + position ~ variable, fun.aggregate = function(x) mean(x, na.rm = T))
z[,'normalized_lowANDhigh'][z[,'normalized_lowANDhigh'] == 0] = NA

z$perm = rep(NA, nrow(z))


for (i in levels(z$gene)){
  idx = z$gene == i
  z[idx,'perm'] = range01(z[idx,'normalized_lowANDhigh'],remove.na = T)
}

SuppFig12_c = ggplot(z) +
  geom_tile(aes(x=position,y=domain,fill=normalized_lowANDhigh)) +
  scico::scale_fill_scico(palette = 'bilbao', na.value = 'cyan') +
  facet_grid(gene~.) +
  theme_classic() +
  ggtitle('P2X3')

p2x3z = z

#P2X3####

d = z[z$gene == 'P2X3',c('domain','position','perm')]


#remove domains and positions missing 80% data
perm_pivot<-dcast(d,position~domain,value.var='perm')
perm_pivot_plot<-perm_pivot[rowMeans(is.na(perm_pivot)) <= .8,]
perm_pivot_plot<-perm_pivot_plot[,colMeans(is.na(perm_pivot_plot)) <= .8]

perm_plot_melt<-melt(perm_pivot_plot,id.vars='position')
perm_plot_melt<-na.omit(perm_plot_melt)
colnames(perm_plot_melt)<-c('position','domain','permissibility')

perm_pivot_zeros = perm_pivot_plot
perm_pivot_zeros[is.na(perm_pivot_zeros)] = 0


#umap
df.umap = umap(perm_pivot_zeros[,-1], 
                     metric = 'euclidean',
                     n_epochs = 500,
                     n_neighbors = 20,
                     nn_method = 'annoy',
                     n_trees = 100,
                     bandwidth = 1
)


scores_P2X3 = data.frame(df.umap) # PC score matrix
scores_P2X3$group = NbClust(scores_P2X3, method = 'kmeans')$Best.partition
```


```
*** : The Hubert index is a graphical method of determining the number of clusters.
                In the plot of Hubert index, we seek a significant knee that corresponds to a 
                significant increase of the value of the measure i.e the significant peak in Hubert
                index second differences plot.
```


```
*** : The D index is a graphical method of determining the number of clusters. 
                In the plot of D index, we seek a significant knee (the significant peak in Dindex
                second differences plot) that corresponds to a significant increase of the value of
                the measure. 
 
******************************************************************* 
* Among all indices:                                                
* 11 proposed 2 as the best number of clusters 
* 1 proposed 3 as the best number of clusters 
* 5 proposed 4 as the best number of clusters 
* 3 proposed 10 as the best number of clusters 
* 2 proposed 12 as the best number of clusters 
* 2 proposed 15 as the best number of clusters 

                   ***** Conclusion *****                            
 
* According to the majority rule, the best number of clusters is  2 
 
 
*******************************************************************
```


#Supplementary Figure 2 & 12 for Kv13


```
d = z[z$gene == 'Kv13',c('domain','position','perm')]


#remove domains and positions missing 80% data
perm_pivot = dcast(d,position~domain,value.var='perm')
perm_pivot_plot = perm_pivot[rowMeans(is.na(perm_pivot)) <= .8,]
perm_pivot_plot = perm_pivot_plot[,colMeans(is.na(perm_pivot_plot)) <= .8]

perm_plot_melt = melt(perm_pivot_plot,id.vars='position')
perm_plot_melt = na.omit(perm_plot_melt)
colnames(perm_plot_melt) = c('position','domain','permissibility')

perm_pivot_zeros = perm_pivot_plot
perm_pivot_zeros[is.na(perm_pivot_zeros)] = 0

#umap
df.umap = umap(perm_pivot_zeros[,-1], 
                     metric = 'cosine',
                     n_epochs = 500,
                     n_neighbors = 20,
                     nn_method = 'annoy',
                     n_trees = 100,
                     bandwidth = 0.8
)


scores_Kv13 = data.frame(df.umap) # PC score matrix
scores_Kv13$group = NbClust(scores_Kv13, method = 'ward.D2')$Best.partition
```


```
*** : The Hubert index is a graphical method of determining the number of clusters.
                In the plot of Hubert index, we seek a significant knee that corresponds to a 
                significant increase of the value of the measure i.e the significant peak in Hubert
                index second differences plot.
```


```
*** : The D index is a graphical method of determining the number of clusters. 
                In the plot of D index, we seek a significant knee (the significant peak in Dindex
                second differences plot) that corresponds to a significant increase of the value of
                the measure. 
 
******************************************************************* 
* Among all indices:                                                
* 6 proposed 2 as the best number of clusters 
* 8 proposed 3 as the best number of clusters 
* 4 proposed 4 as the best number of clusters 
* 2 proposed 7 as the best number of clusters 
* 1 proposed 14 as the best number of clusters 
* 3 proposed 15 as the best number of clusters 

                   ***** Conclusion *****                            
 
* According to the majority rule, the best number of clusters is  3 
 
 
*******************************************************************
```


#Supplementary Figure 2


```
ggarrange(plot_kir21_umap, SuppFig2_b,
          SuppFig2_c, SuppFig2_d,
          SuppFig2_e, SuppFig2_f,
          nrow = 3, ncol = 2,
          labels = c('a','b','c','d','e','f'))
```


#Supplementary Figure 12


```
ggarrange(SuppFig12_b, SuppFig12_b,
          SuppFig12_c, SuppFig12_d,
          SuppFig12_e, SuppFig12_f,
          nrow = 3, ncol = 2,
          labels = c('a','b','c','d','e','f'))
```


#Supplementary Figure 13a


```
#spearman correlations

df = rbind(asic1az, kir21z, kir31z, kv13z, p2x3z)
df$domain = as.factor(df$domain)
df$int = interaction(df$gene, df$domain)
df[df == 0] = NA
df[is.nan(df$perm),'perm'] = NA

df_pivot = dcast(df, domain~gene, value.var = 'perm', fun.aggregate = function(x){mean(x, na.rm = T)}) #SuppFig13

df_cor = cor(df_pivot[,-1], method = 'spearman', use = 'pairwise.complete.obs')

c1 = cor.mtest(df_pivot[,-1])

corrplot(df_cor, type = "upper", order = "hclust",
                   hclust.method = 'ward.D2',
                   tl.col = "black", tl.srt = 45,
                   p.mat = c1$p, sig.level = 0.2)
```


```
NA
NA
NA
```


#Supplementary Figure 13b-e


```
p2 = ggscatter(df_pivot, x = 'Kir21', y= 'Kir31',
                  add = 'reg.line', conf.int = T,
                  cor.coef = T, cor.method = 'pearson',
                  xlab = 'Kir21', ylab = 'Kir31')

p3 = ggscatter(df_pivot, x = 'Kir21', y= 'Kv13',
                  add = 'reg.line', conf.int = T,
                  cor.coef = T, cor.method = 'pearson',
                  xlab = 'Kir21', ylab = 'Kv13')

p4 = ggscatter(df_pivot, x = 'Asic1a', y= 'Kv13',
                  add = 'reg.line', conf.int = T,
                  cor.coef = T, cor.method = 'pearson',
                  xlab = 'Asic1a', ylab = 'Kv13')

p5 = ggscatter(df_pivot, x = 'Asic1a', y= 'Kir31',
                  add = 'reg.line', conf.int = T,
                  cor.coef = T, cor.method = 'pearson',
                  xlab = 'Asic1a', ylab = 'Kir31')

ggarrange(p2,p3,p4,p5,
          ncol = 2, nrow = 2,
          labels = c('b','c','d','e'))
```


#Supplementary Figure 14


```
df_pivot = dcast(df, position~int, value.var = 'perm', fun.aggregate = function(x){sum(x, na.rm = T)})
df_cor = cor(df_pivot[,-1], method = 'spearman', use = 'pairwise.complete.obs')

c1 = cor.mtest(df_pivot[,-1])

corrplot(df_cor, type = "upper", order = "hclust",
                   hclust.method = 'ward.D2',
                   tl.col = "black", tl.srt = 45,
                   p.mat = c1$p, sig.level = 0.2)
```


LS0tCnRpdGxlOiAiQ295b3RlLU1hZXN0YXMgZXQuIGFsICgyMDIxKSBOYXR1cmUgQ29tbXVuaWNhdGlvbnMgU291cmNlIERhdGEiCm91dHB1dDogaHRtbF9ub3RlYm9vawotLS0KClRoaXMgaXMgYW4gW1IgTWFya2Rvd25dKGh0dHA6Ly9ybWFya2Rvd24ucnN0dWRpby5jb20pIE5vdGVib29rLiBXaGVuIHlvdSBleGVjdXRlIGNvZGUgd2l0aGluIHRoZSBub3RlYm9vaywgdGhlIHJlc3VsdHMgYXBwZWFyIGJlbmVhdGggdGhlIGNvZGUuIAoKVHJ5IGV4ZWN1dGluZyB0aGlzIGNodW5rIGJ5IGNsaWNraW5nIHRoZSAqUnVuKiBidXR0b24gd2l0aGluIHRoZSBjaHVuayBvciBieSBwbGFjaW5nIHlvdXIgY3Vyc29yIGluc2lkZSBpdCBhbmQgcHJlc3NpbmcgKkNtZCtTaGlmdCtFbnRlciouIAoKYGBge3IsIHNldHVwLCBpbmNsdWRlPUZBTFNFfQprbml0cjo6b3B0c19rbml0JHNldChyb290LmRpciA9ICcuLycpICNzZXQgdG8gY29ycmVjdCB3b3JraW5nIGRpcmVjdG9yeQpgYGAKCiNzZXR1cApgYGB7ciBkYXRhIGludGFrZSwgbWVzc2FnZT1GQUxTRSwgd2FybmluZz1GQUxTRX0KbGlicmFyeShyZXNoYXBlMikKbGlicmFyeShwbHlyKQpsaWJyYXJ5KGRwbHlyKQpsaWJyYXJ5KHRpZHlyKQpsaWJyYXJ5KGdncGxvdDIpCmxpYnJhcnkoQ29tcGxleEhlYXRtYXApCmxpYnJhcnkoZ2dwdWJyKQpsaWJyYXJ5KHJhbmRvbUZvcmVzdCkKbGlicmFyeShpbWwpCmxpYnJhcnkoUkZMUHRvb2xzKQpsaWJyYXJ5KGNvcnJwbG90KQpsaWJyYXJ5KE5iQ2x1c3QpCmxpYnJhcnkobHNhKQpsaWJyYXJ5KGNpcmNsaXplKQpsaWJyYXJ5KHBseXIpCmxpYnJhcnkodXdvdCkKbGlicmFyeShvcGVueGxzeCkKbGlicmFyeShzY2ljbykKbGlicmFyeSh5YUltcHV0ZSkKCnJhbmdlMDEgPSBmdW5jdGlvbih4LCByZW1vdmUubmEgPSBGKXsKICBpZihyZW1vdmUubmEpewogICAgKHgtbWluKHgsIG5hLnJtID0gVCkpLyhtYXgoeCwgbmEucm0gPSBUKS1taW4oeCwgbmEucm0gPSBUKSkKICB9CiAgZWxzZXsKICAgICh4LW1pbih4KSkvKG1heCh4KS1taW4oeCkpCiAgfQp9CmBgYAoKI0Rvd25sb2FkIGRhdGEgZnJvbSBaZW5vZG8KVGhpcyByZXF1aXJlcyBpbmJvcnV0aWxzIChodHRwczovL2dpdGh1Yi5jb20vaW5iby9pbmJvcnV0aWxzKQpOb3RlIHRoYXQgdGhlIGRvd25sb2FkIGlzIHZlcnkgbGFyZ2UgKDMuMTZHQikuCgpgYGB7cn0KaW5ib3J1dGlsczo6ZG93bmxvYWRfemVub2RvKGRvaSA9ICIxMC41MjgxL3plbm9kby41NjgzNTY2IikKYGBgCgojQWx0ZXJuYXRpdmU6IGxvYWQgYSBsb2NhbCBjb3B5IG9mIHRoZSBkYXRhCkRvd25sb2FkIHRoZSBkYXRhIGZyb20gWmVub2RvIChodHRwczovL2RvaS5vcmcvMTAuNTI4MS96ZW5vZG8uNTY4MzU2NikgbWFudWFsbHkKTW92ZSB0aGUgZGF0YWZpbGUgKFN1cHBsZW1lbnRhbF9EYXRhXzYuUkRhdGEpIGluIHRoZSBwYXRoClVuY29tbWVudCB0aGUgbGluZSBiZWxvdwoKYGBge3J9CiN1bmNvbW1lbnQgdGhpcyBsaW5lIHRvIGxvYWQgYSBsb2NhbCBjb3B5IG9mIHRoZSBkYXRlCiNsb2FkKCdTdXBwbGVtZW50YWxfRGF0YV82LlJEYXRhJykKYGBgCgojS2lyMi4xIExhcmdlIERhdGFzZXQgOyBtZXJnZSB3aXRoIGNhbGN1bGF0ZWQgcHJvcGVydGllcwpgYGB7ciBtZXNzYWdlPUZBTFNFLCB3YXJuaW5nPUZBTFNFfQojcmVtb3ZlIGFueSBkdXBsaWNhdGVzCmQgPSB1bmlxdWUoa2lyMjFfbGFyZ2VfZGF0YXNldCkKCiNyZW1vdmUgYW55IE5BcwpkX2NvbXBsZXRlID0gZFtjb21wbGV0ZS5jYXNlcyhkJG5vcm1hbGl6ZWRfZHApLF0KI3JlcGxhY2Ugb3V0bGllcnMgYWJvdmUgb3IgYmVsb3cKZF9jb21wbGV0ZVtkX2NvbXBsZXRlJG5vcm1hbGl6ZWRfZHA+NSwnbm9ybWFsaXplZF9kcCddID0gNQpkX2NvbXBsZXRlW2RfY29tcGxldGUkbm9ybWFsaXplZF9kcDwoLTUpLCdub3JtYWxpemVkX2RwJ10gPSAoLTUpCgojcmVtb3ZlIGRvbWFpbnMgYW5kIHBvc2l0aW9ucyBtaXNzaW5nIDgwJSBkYXRhCnBlcm1fcGl2b3QgPSBkY2FzdChkX2NvbXBsZXRlLHBvc2l0aW9ufmRvbWFpbix2YWx1ZS52YXI9J25vcm1hbGl6ZWRfZHAnKQpwZXJtX3Bpdm90X3Bsb3QgPSBwZXJtX3Bpdm90W3Jvd01lYW5zKGlzLm5hKHBlcm1fcGl2b3QpKSA8PSAuOCxdCnBlcm1fcGl2b3RfcGxvdCA9IHBlcm1fcGl2b3RfcGxvdFssY29sTWVhbnMoaXMubmEocGVybV9waXZvdF9wbG90KSkgPD0gLjVdCgpwZXJtX3Bsb3RfbWVsdCA9IG1lbHQocGVybV9waXZvdF9wbG90LGlkLnZhcnM9J3Bvc2l0aW9uJykKcGVybV9wbG90X21lbHQgPSBuYS5vbWl0KHBlcm1fcGxvdF9tZWx0KQpjb2xuYW1lcyhwZXJtX3Bsb3RfbWVsdCkgPSBjKCdwb3NpdGlvbicsJ2RvbWFpbicsJ3Blcm1pc3NpYmlsaXR5JykKCiNtZXJnZSB3aXRoIGRvbWFpbiBhbm5vdGF0aW9ucwphbm5vdGF0ZWRfa2lyMjEgPSBtZXJnZShwZXJtX3Bsb3RfbWVsdCxkb21haW5fYW5ub3RhdGlvbnMpCgojUUMgb24gY29tcGxldGVuZXNzIG9mIGdyb3Vwcwphbm5vdGF0ZWRfb25seV9hbm5vdGF0aW9uc19raXIyMSA9IGFubm90YXRlZF9raXIyMQphbm5vdGF0ZWRfb25seV9hbm5vdGF0aW9uc19raXIyMSRwZXJtaXNzaWJpbGl0eSA9IE5VTEwKYW5ub3RhdGVkX29ubHlfYW5ub3RhdGlvbnNfa2lyMjEkcG9zaXRpb24gPSBOVUxMCmFubm90YXRlZF9vbmx5X2Fubm90YXRpb25zX2tpcjIxID0gdW5pcXVlKGFubm90YXRlZF9vbmx5X2Fubm90YXRpb25zX2tpcjIxWywxOjhdKQoKI21lcmdlIHdpdGggS2lyIGFyY2hpdGVjdHVyZSBhbm5vdGF0aW9uCmFubm90YXRlZF9raXIyMSA9IG1lcmdlKGRvbWFpbl9hcmNoX2tleSxhbm5vdGF0ZWRfa2lyMjEpCgojbWVyZ2UgY2FsY3VsYXRlZCBwcm9wZXJ0aWVzCiNqdXN0IHNlcXVlbmNlCmtpcjIxX3NlcV9kb20gPSBtZXJnZShhbm5vdGF0ZWRfa2lyMjEsZG9tYWluX3NlcXVlbmNlX2ZlYXQpCmtpcjIxX3NlcV9kb21fbm9fYW5ub3QgPSBkYXRhLmZyYW1lKAogIGRvbWFpbiA9IGtpcjIxX3NlcV9kb20kZG9tYWluLAogIHBlcm1pc3NpYmlsaXR5ID0ga2lyMjFfc2VxX2RvbSRwZXJtaXNzaWJpbGl0eSxraXIyMV9zZXFfZG9tWywyNDoyOTNdKQoKI21lcmdlIHdpdGggY2FsY3VsYXRlZCBzdHJ1Y3R1cmFsIHByb3BlcnRpZXMKa2lyMjFfc3RydWNfZG9tID0gbWVyZ2UoYW5ub3RhdGVkX2tpcjIxLGRvbWFpbl9zdHJ1Y3R1cmFsX2ZlYXQpCgojbWVyZ2Ugd2l0aCByZXNpZHVlIGNvbnRhY3QgbWFwIChBcnBlZ2dpbykKY291bnRfMSA9ICBraXIyMV9hcnBlZ2dpb19jb250YWN0cyAlPiUgY291bnQoUmVzTnVtMSkKY29sbmFtZXMoY291bnRfMSk8LWMoJ3N0cnVjdHVyZScsJ2NvdW50XzEnKQpjb3VudF8yID0gIGtpcjIxX2FycGVnZ2lvX2NvbnRhY3RzICU+JSBjb3VudChSZXNOdW0yKQpjb2xuYW1lcyhjb3VudF8yKTwtYygnc3RydWN0dXJlJywnY291bnRfMicpCmNvdW50X2FsbCA9IG1lcmdlKGNvdW50XzEsY291bnRfMixhbGw9VFJVRSkKY291bnRfYWxsW2lzLm5hKGNvdW50X2FsbCldPC0wCmNvdW50X2FsbCA9IGRhdGEuZnJhbWUoCiAgc3RydWN0dXJlPWNvdW50X2FsbCRzdHJ1Y3R1cmUsCiAgY29udGFjdHMgPSBjb3VudF9hbGwkY291bnRfMStjb3VudF9hbGwkY291bnRfMikKY291bnRfYWxsID0gZGF0YS5mcmFtZSgKICBzdHJ1Y3R1cmU9Y291bnRfYWxsJHN0cnVjdHVyZSwKICBjb250YWN0cz1jb3VudF9hbGwkY29udGFjdHMsCiAgY29udGFjdF9kZW5zaXR5PWNvdW50X2FsbCRjb250YWN0cy8zMzApCmtpcjIxX2NvbnRhY3RzID0gbWVyZ2UoYW5ub3RhdGVkX2tpcjIxLGNvdW50X2FsbCxhbGw9VFJVRSkKCmBgYAoKI0ZpZ3VyZSAxYi1lCmBgYHtyIEZpZ3VyZSAxYi1lfQojdW5tZWx0IGRvbWFpbnMjIyMjIwpwZXJtX3Bpdm90PC1kY2FzdChkX2NvbXBsZXRlLHBvc2l0aW9ufmRvbWFpbix2YWx1ZS52YXI9J25vcm1hbGl6ZWRfZHAnKQoKI2NvdW50IG51bWJlciBvZiBtaXNzaW5nIGluIHBvc2l0aW9ucyBhbmQgZG9tYWlucwpwZXJtX21pc3NfcG9zID0gZGF0YS5mcmFtZShwb3NpdGlvbj1zZXEoMTpucm93KHBlcm1fcGl2b3QpKSxtaXNzaW5nPXJvd01lYW5zKGlzLm5hKHBlcm1fcGl2b3QpKSkKcGVybV9taXNzX2RvbSA9IGRhdGEuZnJhbWUoZG9tYWluPXNlcSgxOm5jb2wocGVybV9waXZvdCkpLG1pc3Npbmc9Y29sTWVhbnMoaXMubmEocGVybV9waXZvdCkpKQoKI3Bsb3QgbnVtYmVyIG9mIG1pc3NpbmcKZ2dfbWlzc19wb3Nfc2NhdHRlciA9IGdncGxvdChwZXJtX21pc3NfcG9zLGFlcyh4PXBvc2l0aW9uLHk9bWlzc2luZykpK2dlb21fcG9pbnQoKSsKICBsYWJzKHRpdGxlPSdNaXNzaW5nIEFtaW5vIEFjaWQgUG9zaXRpb25zJyx4PSdQb3NpdGlvbnMnLHk9J21pc3NpbmcgcGVyY2VudGFnZScpKwogIHRoZW1lX2NsYXNzaWMoKQpnZ19taXNzX2RvbWFpbl9zY2F0dGVyID0gZ2dwbG90KHBlcm1fbWlzc19kb20sYWVzKHg9ZG9tYWluLHk9bWlzc2luZykpKwogIGdlb21fcG9pbnQoKStsYWJzKHRpdGxlPSdNaXNzaW5nIERvbWFpbnMnLHg9J0RvbWFpbnMnLHk9J21pc3NpbmcgcGVyY2VudGFnZScpKwogIHRoZW1lX2NsYXNzaWMoKQpnZ19taXNzX3Bvc19kZW5zaXR5PC1nZ3Bsb3QocGVybV9taXNzX3BvcyxhZXMoeD1taXNzaW5nKSkrZ2VvbV9kZW5zaXR5KCkrCiAgdGhlbWVfY2xhc3NpYygpCmdnX21pc3NfZG9tX2RlbnNpdHk8LWdncGxvdChwZXJtX21pc3NfZG9tLGFlcyh4PW1pc3NpbmcpKStnZW9tX2RlbnNpdHkoKSsKICB0aGVtZV9jbGFzc2ljKCkKCmdnYXJyYW5nZShnZ19taXNzX3Bvc19zY2F0dGVyLCBnZ19taXNzX2RvbWFpbl9zY2F0dGVyLCBnZ19taXNzX3Bvc19kZW5zaXR5LCBnZ19taXNzX2RvbV9kZW5zaXR5LCAKICAgICAgICAgIG5jb2wgPSAyLCBucm93ID0gMikKYGBgCiNGaWd1cmUgMmEKYGBge3IgRmlndXJlIDJhfQojU3RhcnQgY2FsY3VsYXRpbmcgZ3JvdXAgbWVhbnMKI3JlbW92ZSBpbmNvbXBsZXRlIGRhdGEgKGRvbWFpbnMgd2l0aCA1MCUgbWlzc2luZyBwb3NpdGlvbnMgYW5kIHBvc2l0aW9ucyB3aXRoIDgwJSBtaXNzaW5nIGRvbWFpbnMpCnBlcm1fcGl2b3RfcWMgPSBwZXJtX3Bpdm90W3Jvd01lYW5zKGlzLm5hKHBlcm1fcGl2b3QpKSA8PSAuOCxdCnBlcm1fcGl2b3RfcWMgPSBwZXJtX3Bpdm90X3FjWyxjb2xNZWFucyhpcy5uYShwZXJtX3Bpdm90KSkgPD0gLjhdIAoKI3NjYWxlIGRhdGEgcHJpb3IgdG8gZGlzdGFuY2UgbWVhc3VyZQpwZXJtX3NjYWxlZCA9IHNjYWxlKHBlcm1fcGl2b3RfcWNbLC0xXSxjZW50ZXI9RikKCiNyZXBsYWNlIE5BcyB3aXRoIHplcm9zCnBlcm1fcGl2b3RfemVyb3MgPSBwZXJtX3NjYWxlZApwZXJtX3Bpdm90X3plcm9zW2lzLm5hKHBlcm1fc2NhbGVkKV0gPSAwCgojY2FsY3VsYXRlIGNvc2luZSBzaW1pbGFyaXR5CnBlcm1fbWF0cml4ID0gZGF0YS5tYXRyaXgocGVybV9waXZvdF96ZXJvcykKY29zaW5lX3NpbSA9IGNvc2luZShwZXJtX21hdHJpeCkKCiNjb252ZXJ0IHNpbWlsYXJpdHkgdG8gZGlzdGFuY2UgbWF0cml4CmNvc2luZV9kaXN0YW5jZSA9IHNpbTJkaXN0KGNvc2luZV9zaW0sIG1heFNpbSA9IDEpCgojY2x1c3RlciBiYXNlZCBvbiBjb3NpbmUgc2ltaWxhcml0eQpyZXMuaGNfZG9tX2NvcyA9IGhjbHVzdChjb3NpbmVfZGlzdGFuY2UsICBtZXRob2QgPSAid2FyZC5EMiIpCgojY2FsY3VsYXRlIGNvc2luZSBzaW1pbGFyaXR5IGFjcm9zcyBwb3NpdGlvbnMKY29zaW5lX3NpbSA9IGNvc2luZSh0KHBlcm1fbWF0cml4KSkKCiNjb252ZXJ0IHNpbWlsYXJpdHkgdG8gZGlzdGFuY2UgbWF0cml4CmNvc2luZV9kaXN0YW5jZT0gc2ltMmRpc3QoY29zaW5lX3NpbSwgbWF4U2ltID0gMSkKCiNjbHVzdGVyIGJhc2VkIG9uIGNvc2luZSBzaW1pbGFyaXR5CnJlcy5oY19wb3NfY29zIDwtIGhjbHVzdChjb3NpbmVfZGlzdGFuY2UsICBtZXRob2QgPSAid2FyZC5EMiIpCgoKY29sX2Z1bjEgPSBjb2xvclJhbXAyKGMoLTEsIDAsIDEpLCBjKCIjMzc3RUI4IiwgIndoaXRlIiwgIiNFNDFBMUMiKSkKSGVhdG1hcCh0KHBlcm1fcGl2b3RfcWNbLC0xXSksCiAgICAgICAgY29sID0gY29sX2Z1bjEsCiAgICAgICAgY2x1c3Rlcl9yb3dzID0gcmVzLmhjX2RvbV9jb3MsCiAgICAgICAgY29sdW1uX3RpdGxlX2dwID0gZ3Bhcihmb250c2l6ZSA9IDE0KSwgCiAgICAgICAgY2x1c3Rlcl9jb2x1bW5zID0gRkFMU0UsCiAgICAgICAgcm93X2RlbmRfd2lkdGggPSB1bml0KDIwLCAibW0iKSwKICAgICAgICByb3dfbmFtZXNfZ3AgPSBncGFyKGZvbnRzaXplID0gMiksCiAgICAgICAgY29sdW1uX25hbWVzX2dwID0gZ3Bhcihmb250c2l6ZSA9IDIpLAogICAgICAgIGhlYXRtYXBfbGVnZW5kX3BhcmFtID0gbGlzdCh0aXRsZSA9ICd6LXNjb3JlZCBmaXRuZXNzJykpCgpgYGAKCiNTdXBwbGVtZW50YXJ5IEZpZ3VyZSAxCmBgYHtyIFN1cHBsZW1lbnRhcnkgRmlndXJlIDEsIG1lc3NhZ2U9RkFMU0UsIHdhcm5pbmc9RkFMU0V9CnBlcm1fcGl2b3Rfc2UgPSBkY2FzdChkX2NvbXBsZXRlLHBvc2l0aW9ufmRvbWFpbix2YWx1ZS52YXI9J3NlX2RwJykKcGVybV9waXZvdF9zZV9xYyA9IHBlcm1fcGl2b3Rfc2Vbcm93TWVhbnMoaXMubmEocGVybV9waXZvdCkpIDw9IC44LF0KcGVybV9waXZvdF9zZV9xYyA9IHBlcm1fcGl2b3Rfc2VfcWNbLGNvbE1lYW5zKGlzLm5hKHBlcm1fcGl2b3QpKSA8PSAuOF0KCmNvbF9mdW4yID0gY29sb3JSYW1wMihzZXEoMCwyLCBieSA9IDAuMiksIAogICAgICAgICAgICAgICAgICAgICAgICAgICAgICAgIHNjaWNvKDExLCBwYWxldHRlID0gJ2J1ZGEnLCBkaXJlY3Rpb24gPSAtMSkpCkhlYXRtYXAodChwZXJtX3Bpdm90X3NlX3FjWywtMV0pLAogICAgICAgIGNvbCA9IGNvbF9mdW4yLAogICAgICAgIGNsdXN0ZXJfcm93cyA9IHJlcy5oY19kb21fY29zLAogICAgICAgIGNvbHVtbl90aXRsZV9ncCA9IGdwYXIoZm9udHNpemUgPSAxNCksIAogICAgICAgIGNsdXN0ZXJfY29sdW1ucyA9IEZBTFNFLAogICAgICAgIHJvd19kZW5kX3dpZHRoID0gdW5pdCgyMCwgIm1tIiksCiAgICAgICAgcm93X25hbWVzX2dwID0gZ3Bhcihmb250c2l6ZSA9IDIpLAogICAgICAgIGNvbHVtbl9uYW1lc19ncCA9IGdwYXIoZm9udHNpemUgPSAyKSwKICAgICAgICBoZWF0bWFwX2xlZ2VuZF9wYXJhbSA9IGxpc3QodGl0bGUgPSAnZml0bmVzcyBzdGFuZGFyZCBlcnJvcicpKQoKYGBgCiNGaWd1cmUgMmMKYGBge3IgRmlndXJlIDJjfQpnZ3Bsb3QoZF9jb21wbGV0ZSwgYWVzKHg9bm9ybWFsaXplZF9kcCkpICsgZ2VvbV9oaXN0b2dyYW0oYmlucyA9IDIwMCwgZmlsbCA9ICdibGFjaycpICsgeGxhYignei1zY29yZWQgZml0bmVzcycpCmBgYAojRmlndXJlIDJkCmBgYHtyIEZpZ3VyZSAyZH0KI3Byb2R1Y3RpdmUgaW5zZXJ0aW9ucwpwcm9kX2lucyRsb2NhdGlvbiA9IGZhY3Rvcihwcm9kX2lucyRsb2NhdGlvbiwgbGV2ZWxzID0gYygnTi10ZXJtJywnTWlkZGxlJywnQy10ZXJtJykpCmdncGxvdChwcm9kX2lucywgCiAgICAgICBhZXMoeD1sb2NhdGlvbiwgeT1wZXJfcHJvZF9pbnMsIGZpbGw9YXMuZmFjdG9yKHR5cGUpKSkgKyAKICBnZW9tX2NvbChwb3NpdGlvbiA9ICdkb2RnZScpICsKICB5bGFiKCdmcmFjdGlvbiBwcm9kdWN0aXZlIGluc2VydGlvbnMnKQpgYGAKCiNTdXBwbGVtZW50YXJ5IEZpZ3VyZSAzCmBgYHtyIFN1cHBsZW1lbnRhcnkgRmlndXJlIDMsIGVjaG89RkFMU0UsIG1lc3NhZ2U9RkFMU0UsIHdhcm5pbmc9RkFMU0V9CiNjcmVhdGUgZ3JvdXBpbmdzIGJhc2VkIG9uIGNsdXN0ZXJzCmN1dF9jbHVzdF9kb20gPSBkYXRhLmZyYW1lKGRvbV9oaWVyXzM9Y3V0cmVlKHJlcy5oY19kb21fY29zLCBrID0gMyksZG9tX2hpZXJfMjA9Y3V0cmVlKHJlcy5oY19kb21fY29zLCBrID0gMTkpKQpjdXRfY2x1c3RfZG9tID0gZGF0YS5mcmFtZShkb21haW49cm93bmFtZXMoY3V0X2NsdXN0X2RvbSksY3V0X2NsdXN0X2RvbSkKY3V0X2NsdXN0X2RvbSA9IGxhcHBseShjdXRfY2x1c3RfZG9tLGFzLmNoYXJhY3RlcikKY2x1c3RlcnMgPSBhcy5kYXRhLmZyYW1lKGRvLmNhbGwoY2JpbmQsIGN1dF9jbHVzdF9kb20pKSAKY2x1c3RlcnNfcHJvcHMgPSBtZXJnZShjbHVzdGVycywgZG9tYWluX2Fubm90YXRpb25zKQoKZmVhdF9zZWwgPSBjKCdQREInLAogICAgICAgICAgICAgJ2RfQUFfaHlkcm9waG9iX21lYW4nLAogICAgICAgICAgICAgJ2RfQUFfbmVnYXRfbWVhbicsCiAgICAgICAgICAgICAnZF9BQV92b2xfbl83JywKICAgICAgICAgICAgICdkb21haW5fbGVuZ3RoJykKCmNsdXN0ZXJzX3Byb3BzID0gbWVyZ2UoY2x1c3RlcnNfcHJvcHMsIGRvbWFpbl9zZXF1ZW5jZV9mZWF0WyxmZWF0X3NlbF0pCgpjbHVzdGVyX3N0YXRzID0gY2x1c3RlcnNfcHJvcHMgJT4lCiAgZ3JvdXBfYnkoZG9tX2hpZXJfMywgZ3JvdXAsIGdyb3VwX3JlZHVjZWQpICU+JQogIHN1bW1hcmlzZShjb3VudCA9IG4oKSwKICAgICAgICAgICAgaHlkcm9waG9iaWNpdHkgPSBtZWFuKGRfQUFfaHlkcm9waG9iX21lYW4pLAogICAgICAgICAgICBzaXplID0gbWVhbihkb21haW5fbGVuZ3RoKSwKICAgICAgICAgICAgbmVnYXQgPSBtZWFuKGRfQUFfbmVnYXRfbWVhbiksCiAgICAgICAgICAgIHZvbF9uXzcgPSBtZWFuKGRfQUFfdm9sX25fNykpCgpnZ3Bsb3QoY2x1c3Rlcl9zdGF0cywgYWVzKHggPSBkb21faGllcl8zLCB5ID0gY291bnQpKSArCiAgZ2VvbV9jb2woYWVzKGZpbGwgPSBncm91cCkpICsKICB4bGFiKCdDbHVzdGVyIycpICsKICBzY2FsZV9maWxsX21hbnVhbCh2YWx1ZXMgPSBjKCcjN2ZjOTdmJywgJyNiZWFlZDQnLCAnI2ZkYzA4NicsICcjZmZmZjk5JywgJyMzODZjYjAnLCAnI2YwMDI3ZicsICcjYmY1YjE3JykpCgpgYGAKI0ZpZ3VyZSAzYy1oCmBgYHtyIEZpZ3VyZSAzYy1oLCBmaWcuaGVpZ2h0PTUsIGZpZy53aWR0aD01LCBtZXNzYWdlPUZBTFNFLCB3YXJuaW5nPUZBTFNFfQpjbHVzdGVyc19wcm9wcyRkb21faGllcl8zID0gYXMuZmFjdG9yKGNsdXN0ZXJzX3Byb3BzJGRvbV9oaWVyXzMpCmNsdXN0ZXJzX3Byb3BzJGRvbV9oaWVyXzMgPSByZXZhbHVlKGNsdXN0ZXJzX3Byb3BzJGRvbV9oaWVyXzMsCiAgICAgICAgICAgICAgICAgICAgICAgICAgICAgICAgICAgICAgICAgIGMoJzEnID0gJ3N0cnVjdHVyZWRfbW90aWYnLAogICAgICAgICAgICAgICAgICAgICAgICAgICAgICAgICAgICAgICAgICAgICcyJyA9ICd1bnN0cnVjdHVyZWRfbW90aWYnLAogICAgICAgICAgICAgICAgICAgICAgICAgICAgICAgICAgICAgICAgICAgICczJyA9ICdoeWRyb3Bob2JpY19tb3RpZicpKQpteV9jb21wYXJpc29ucyA9IGxpc3QoCiAgYygnc3RydWN0dXJlZF9tb3RpZicsICd1bnN0cnVjdHVyZWRfbW90aWYnKSwgCiAgYygndW5zdHJ1Y3R1cmVkX21vdGlmJywgJ2h5ZHJvcGhvYmljX21vdGlmJyksIAogIGMoJ3N0cnVjdHVyZWRfbW90aWYnLCAnaHlkcm9waG9iaWNfbW90aWYnKQogICkKCiMgYm94IHBsb3RzIHdpdGggcGFpcndpc2UgcC12YWx1ZXMKbGVuZ3RoX2JveHBsb3QgPSBnZ2JveHBsb3QoY2x1c3RlcnNfcHJvcHMsIHggPSAiZG9tX2hpZXJfMyIsIHkgPSAiZG9tYWluX2xlbmd0aCIsCiAgICAgICAgICAgICAgICAgICAgICAgICAgY29sb3IgPSAiZG9tX2hpZXJfMyIsIHBhbGV0dGUgPSAiamNvIikgKyAKICBmb250KCJ4eS50ZXh0Iiwgc2l6ZSA9IDcpICsKICB4bGFiKCdDbHVzdGVyIycpICsKICBzdGF0X2NvbXBhcmVfbWVhbnMoY29tcGFyaXNvbnMgPSBteV9jb21wYXJpc29ucywgbWV0aG9kPSd3aWxjb3gudGVzdCcpCgpoeWRyb3Bob2JfYm94cGxvdCA9IGdnYm94cGxvdChjbHVzdGVyc19wcm9wcywgeCA9ICJkb21faGllcl8zIiwgeSA9ICJkX0FBX2h5ZHJvcGhvYl9tZWFuIiwKICAgICAgICAgICAgICAgICAgICAgICAgICAgY29sb3IgPSAiZG9tX2hpZXJfMyIsIHBhbGV0dGUgPSAiamNvIikgKyAKICBmb250KCJ4eS50ZXh0Iiwgc2l6ZSA9IDcpICsKICB4bGFiKCdDbHVzdGVyIycpICsKICBzdGF0X2NvbXBhcmVfbWVhbnMoY29tcGFyaXNvbnMgPSBteV9jb21wYXJpc29ucywgbWV0aG9kPSd3aWxjb3gudGVzdCcpIAoKCm5lZ2F0X2JveHBsb3QgPSBnZ2JveHBsb3QoY2x1c3RlcnNfcHJvcHMsIHggPSAiZG9tX2hpZXJfMyIsIHkgPSAiZF9BQV9uZWdhdF9tZWFuIiwKICAgICAgICAgICAgICAgICAgICAgICAgICAgICAgY29sb3IgPSAiZG9tX2hpZXJfMyIsIHBhbGV0dGUgPSAiamNvIikgKwogIGZvbnQoInh5LnRleHQiLCBzaXplID0gNykgKwogIHhsYWIoJ0NsdXN0ZXIjJykgKwogIHN0YXRfY29tcGFyZV9tZWFucyhjb21wYXJpc29ucyA9IG15X2NvbXBhcmlzb25zLCBtZXRob2Q9J3dpbGNveC50ZXN0JykKCgpmaWd1cmVfYm94cGxvdHNfcHJvcHMgPSBnZ2FycmFuZ2UobGVuZ3RoX2JveHBsb3QsIGh5ZHJvcGhvYl9ib3hwbG90LCBuZWdhdF9ib3hwbG90LAogICAgICAgICAgICAgICAgICAgICAgICAgICAgICAgICAgIGxhYmVscyA9IGMoIkEiLCAiQiIsICJDIiksCiAgICAgICAgICAgICAgICAgICAgICAgICAgICAgICAgICAgbmNvbCA9IDMsIG5yb3cgPSAxKQoKY2x1c3RlcnNfcHJvcHMkZG9tYWluX2NsdXN0ZXIgPSBpZmVsc2UoY2x1c3RlcnNfcHJvcHMkZ3JvdXAgPT0gJ3N0cnVjX2RvbWFpbicsICdzdHJ1Y19kb20nLCAnb3RoZXInKQoKZG9tYWluX2xlbmd0aF9kZW5zID0gZ2dwbG90KGNsdXN0ZXJzX3Byb3BzLGFlcyh4PWRvbWFpbl9sZW5ndGgsZmlsbD1kb21haW5fY2x1c3RlcikpKwogIGdlb21faGlzdG9ncmFtKGFlcyh5PTAuNSouLmRlbnNpdHkuLikscG9zaXRpb249J2lkZW50aXR5JyxhbHBoYT0wLjQpKwogIHRoZW1lX2NsYXNzaWMoKSArCiAgdGhlbWUobGVnZW5kLnBvc2l0aW9uID0gIm5vbmUiKQoKZG9tYWluX25lZ2F0X2RlbnMgPSBnZ3Bsb3QoY2x1c3RlcnNfcHJvcHMsYWVzKHg9ZF9BQV9uZWdhdF9tZWFuLGZpbGw9ZG9tYWluX2NsdXN0ZXIpKSsKICBnZW9tX2hpc3RvZ3JhbShhZXMoeT0wLjUqLi5kZW5zaXR5Li4pLHBvc2l0aW9uPSdpZGVudGl0eScsYWxwaGE9MC40KSsKICB0aGVtZV9jbGFzc2ljKCkgKwogIHRoZW1lKGxlZ2VuZC5wb3NpdGlvbiA9ICJub25lIikKCmRvbWFpbl9oeWRyb3Bob2JfZGVucyA9IGdncGxvdChjbHVzdGVyc19wcm9wcyxhZXMoeD1kX0FBX2h5ZHJvcGhvYl9tZWFuLGZpbGw9ZG9tYWluX2NsdXN0ZXIpKSsKICBnZW9tX2hpc3RvZ3JhbShhZXMoeT0wLjUqLi5kZW5zaXR5Li4pLHBvc2l0aW9uPSdpZGVudGl0eScsYWxwaGE9MC40KSsKICB0aGVtZV9jbGFzc2ljKCkgKwogIHRoZW1lKGxlZ2VuZC5wb3NpdGlvbiA9ICJub25lIikKCmZpZ3VyZV9kZW5zX3Byb3BzID0gZ2dhcnJhbmdlKGRvbWFpbl9sZW5ndGhfZGVucywgZG9tYWluX25lZ2F0X2RlbnMsIGRvbWFpbl9oeWRyb3Bob2JfZGVucywKICAgICAgICAgICAgICAgICAgICAgICAgICAgICAgIGxhYmVscyA9IGMoIkQiLCAiRSIsICJGIiksCiAgICAgICAgICAgICAgICAgICAgICAgICAgICAgICBuY29sID0gMywgbnJvdyA9IDEpCgphbGxfcHJvcHMgPSBnZ2FycmFuZ2UobGVuZ3RoX2JveHBsb3QsIGh5ZHJvcGhvYl9ib3hwbG90LCBuZWdhdF9ib3hwbG90LGRvbWFpbl9sZW5ndGhfZGVucywgZG9tYWluX25lZ2F0X2RlbnMsIGRvbWFpbl9oeWRyb3Bob2JfZGVucywKICAgICAgICAgICAgICAgICAgICAgIGxhYmVscyA9IGMoJ0MnLCdEJywnRScsJ0YnLCdHJywnSCcpLAogICAgICAgICAgICAgICAgICAgICAgbmNvbCA9IDMsIG5yb3cgPSAyKQphbGxfcHJvcHMKCmBgYAojRmlndXJlIDRtCmBgYHtyIEZpZ3VyZSA0bSwgbWVzc2FnZT1GQUxTRSwgd2FybmluZz1GQUxTRX0KI2FsbCBwb3NpdGlvbiBiYXNlZCBwcm9wZXJ0aWVzIG1lYW4gdy9vIHdlYWtseSBjb3JyZWxhdGVkCnJvd25hbWVzKHJlY2VpdmluZ19wb3NfY29tYmluZWQpID0gcmVjZWl2aW5nX3Bvc19jb21iaW5lZCRyb3duYW1lCnJlY2VpdmluZ19wb3NfY29tYmluZWQgPSByZWNlaXZpbmdfcG9zX2NvbWJpbmVkWywyOjQyNF0KSGVhdG1hcChyZWNlaXZpbmdfcG9zX2NvbWJpbmVkLGNsdXN0ZXJpbmdfZGlzdGFuY2Vfcm93cyA9ICJldWNsaWRlYW4iLGNsdXN0ZXJfY29sdW1ucyA9RkFMU0UsCiAgICAgICAgcm93X25hbWVzX2dwID0gZ3Bhcihmb250c2l6ZSA9IDUpLAogICAgICAgIGNvbHVtbl9uYW1lc19ncCA9IGdwYXIoZm9udHNpemUgPSAyKSwKICAgICAgICBoZWF0bWFwX2xlZ2VuZF9wYXJhbSA9IGxpc3QodGl0bGUgPSAnU3BlYXJtYW4gY29ycmVsYXRpb24nKSkKYGBgCgojU3VwcGxlbWVudGFyeSBGaWd1cmUgNApgYGB7ciBTdXBwbGVtZW50YXJ5IEZpZ3VyZSA0LCBtZXNzYWdlPUZBTFNFLCB3YXJuaW5nPUZBTFNFfQpIZWF0bWFwKHJlY2VpdmluZ19wb3NfY29tYmluZWQsY2x1c3RlcmluZ19kaXN0YW5jZV9yb3dzID0gJ2V1Y2xpZGVhbicsCiAgICAgICAgY2x1c3RlcmluZ19kaXN0YW5jZV9jb2x1bW5zID0gJ2V1Y2xpZGVhbicsCiAgICAgICAgaGVhdG1hcF9sZWdlbmRfcGFyYW0gPSBsaXN0KHRpdGxlID0gJ1NwZWFybWFuIGNvcnJlbGF0aW9uJyksCiAgICAgICAgcm93X25hbWVzX2dwID0gZ3Bhcihmb250c2l6ZSA9IDUpLAogICAgICAgIGNvbHVtbl9uYW1lc19ncCA9IGdwYXIoZm9udHNpemUgPSAyKSkKYGBgCgojRmlndXJlIDRhLGIsaS1rCmBgYHtyIEZpZ3VyZSA0YSxiLGktaywgZmlnLmhlaWdodD01LCBmaWcud2lkdGg9NSwgbWVzc2FnZT1GQUxTRSwgd2FybmluZz1GQUxTRX0KcmVjaXBpZW50X3N0YXRzID0ga2lyMjFfY29udGFjdHMgJT4lCiAgZ3JvdXBfYnkoS2lyMl8xX1Jlc2lkKSAlPiUKICBzdW1tYXJpc2UodmFyX3Blcm0gPSBzZChwZXJtaXNzaWJpbGl0eSksIG1lYW5fcGVybT1tZWRpYW4ocGVybWlzc2liaWxpdHkpLAogICAgICAgICAgICBybXNmXzNqeWM9bWVhbihybXNmXzNqeWMpLGNkZWdyZWU9bWVhbihjb250YWN0X2RlbnNpdHkpKQoKcmVjaXBpZW50X3N0YXRzID0gcmVjaXBpZW50X3N0YXRzW2NvbXBsZXRlLmNhc2VzKHJlY2lwaWVudF9zdGF0cyksXQoKcmVsYXRpb25zaGlwX3JlY2lwX3Jtc2ZfM2p5YyA9IGdncGxvdChyZWNpcGllbnRfc3RhdHMsIGFlcyh4PXJtc2ZfM2p5YywgeT1tZWFuX3Blcm0pKSArCiAgZ2VvbV9wb2ludCgpICsgdGhlbWVfY2xhc3NpYygpICsgZ2VvbV9zbW9vdGgobWV0aG9kID0gImxvZXNzIiwgY29sPSdyZWQnLCBzcGFuPTAuOCkKCnJlbGF0aW9uc2hpcF9yZWNpcF9jb250YWN0cyA9IGdncGxvdChyZWNpcGllbnRfc3RhdHMsIGFlcyh4PWNkZWdyZWUsIHk9bWVhbl9wZXJtKSkgKwogIGdlb21fcG9pbnQoKSArIHRoZW1lX2NsYXNzaWMoKSArIGdlb21fc21vb3RoKG1ldGhvZCA9ICJsb2VzcyIsIGNvbD0ncmVkJywgc3Bhbj0wLjgpCgpkb25vcl9zdGF0c19zdHJ1YyA9IGtpcjIxX3N0cnVjX2RvbSAlPiUKICBncm91cF9ieShQREIpICU+JQogIHN1bW1hcmlzZSh2YXJfcGVybSA9IHNkKHBlcm1pc3NpYmlsaXR5KSwgbWVhbl9wZXJtID0gbWVkaWFuKHBlcm1pc3NpYmlsaXR5KSwKICAgICAgICAgICAgZF9uY19kaXN0ID0gbWVhbihkX25jX2Rpc3QpKQoKcmVsYXRpb25zaGlwX25jX2Rpc3QgPSBnZ3Bsb3QoZG9ub3Jfc3RhdHNfc3RydWMsIGFlcyh4ID0gZF9uY19kaXN0LCB5ID0gbWVhbl9wZXJtKSkgKwogIGdlb21fcG9pbnQoKSArIHRoZW1lX2NsYXNzaWMoKSArIGdlb21fc21vb3RoKG1ldGhvZCA9ICJsb2VzcyIsIGNvbD0ncmVkJykKCmRvbm9yX3N0YXRzX3NlcSA9IGtpcjIxX3NlcV9kb20gJT4lCiAgZ3JvdXBfYnkoUERCKSAlPiUKICBzdW1tYXJpc2UodmFyX3Blcm0gPSBzZChwZXJtaXNzaWJpbGl0eSksIG1lYW5fcGVybSA9IG1lZGlhbihwZXJtaXNzaWJpbGl0eSksCiAgICAgICAgICAgIGRfQUFfaHlkcm9waG9iX21lYW4gPSBtZWFuKGRfQUFfaHlkcm9waG9iX21lYW4pLAogICAgICAgICAgICBkb21haW5fbGVuZ3RoID0gbWVhbihkb21haW5fbGVuZ3RoKSkKCnJlbGF0aW9uc2hpcF9kb21haW5fbGVuZ3RoID0gZ2dwbG90KGRvbm9yX3N0YXRzX3NlcSwgYWVzKHggPSBkb21haW5fbGVuZ3RoLCB5ID0gbWVhbl9wZXJtKSkgKwogIGdlb21fcG9pbnQoKSArIHRoZW1lX2NsYXNzaWMoKSArIGdlb21fc21vb3RoKG1ldGhvZCA9ICJsb2VzcyIsIGNvbD0ncmVkJykKCnJlbGF0aW9uc2hpcF9kX2h5ZHJvcGhvYiA9IGdncGxvdChkb25vcl9zdGF0c19zZXEsIGFlcyh4ID0gZF9BQV9oeWRyb3Bob2JfbWVhbiwgeSA9IG1lYW5fcGVybSkpICsKICBnZW9tX3BvaW50KCkgKyB0aGVtZV9jbGFzc2ljKCkgKyBnZW9tX3Ntb290aChtZXRob2QgPSAibG9lc3MiLCBjb2w9J3JlZCcpCgpmaWd1cmVfcmVsYXRpb25zaGlwcyA9IGdnYXJyYW5nZShyZWxhdGlvbnNoaXBfcmVjaXBfcm1zZl8zanljLCByZWxhdGlvbnNoaXBfcmVjaXBfY29udGFjdHMsIHJlbGF0aW9uc2hpcF9kb21haW5fbGVuZ3RoLCByZWxhdGlvbnNoaXBfbmNfZGlzdCwgcmVsYXRpb25zaGlwX2RfaHlkcm9waG9iLAogICAgICAgICAgICAgICAgICAgICAgICAgICAgICAgICBsYWJlbHMgPSBjKCJhIiwgImIiLCAiaSIsImoiLCAiayIpLAogICAgICAgICAgICAgICAgICAgICAgICAgICAgICAgICBuY29sID0gMiwgbnJvdyA9IDMpCmZpZ3VyZV9yZWxhdGlvbnNoaXBzCmBgYAoKI0ZpZ3VyZSAzYQpgYGB7ciBGaWd1cmUgM2EsIG1lc3NhZ2U9RkFMU0UsIHdhcm5pbmc9RkFMU0UsIHJlc3VsdHM9RkFMU0V9CnNldC5zZWVkKDQyKQpwZXJtX3Bpdm90X3plcm9zID0gcGVybV9waXZvdF9wbG90CnBlcm1fcGl2b3RfemVyb3NbaXMubmEocGVybV9waXZvdF96ZXJvcyldID0gMAoKZGYudW1hcCA9IHVtYXAocGVybV9waXZvdF96ZXJvc1ssLTFdLCAKICAgICAgICAgICAgICAgbWV0cmljID0gJ2Nvc2luZScsCiAgICAgICAgICAgICAgIG5fZXBvY2hzID0gNTAsCiAgICAgICAgICAgICAgIG5fbmVpZ2hib3JzID0gNTAsCiAgICAgICAgICAgICAgIG5uX21ldGhvZCA9ICdhbm5veScsCiAgICAgICAgICAgICAgIG5fdHJlZXMgPSAyMAopCgoKc2NvcmVzX2tpcjIxX2xhcmdlID0gZGF0YS5mcmFtZShkZi51bWFwKSAjIFBDIHNjb3JlIG1hdHJpeApzY29yZXNfa2lyMjFfbGFyZ2UkZ3JvdXAgPSBhcy5mYWN0b3IoTmJDbHVzdChzY29yZXNfa2lyMjFfbGFyZ2UsIAogICAgICAgICAgICAgICAgICAgICAgICAgICAgICAgICAgICAgICAgICAgICBtZXRob2QgPSAnd2FyZC5EMicpJEJlc3QucGFydGl0aW9uKQpzY29yZXNfa2lyMjFfbGFyZ2UkcG9zaXRpb24gPSBwZXJtX3Bpdm90X3plcm9zJHBvc2l0aW9uCnNjb3Jlc19raXIyMV9sYXJnZSRnZW5lID0gcmVwKCdLaXIyMScsIG5yb3coc2NvcmVzX2tpcjIxX2xhcmdlKSkKCnNjb3Jlc19raXIyMV9sYXJnZSRncm91cCA9IHJldmFsdWUoc2NvcmVzX2tpcjIxX2xhcmdlJGdyb3VwLCAKICAgICAgICAgICAgICAgICAgICAgICAgICAgICAgICAgICBjKCcxJyA9ICdoaWdoJywKICAgICAgICAgICAgICAgICAgICAgICAgICAgICAgICAgICAgICcyJyA9ICdsb3cnLAogICAgICAgICAgICAgICAgICAgICAgICAgICAgICAgICAgICAgJzMnID0gJ2ludGVybWVkaWF0ZScpKQoKcGxvdF9raXIyMV91bWFwID0gZ2dwbG90KHNjb3Jlc19raXIyMV9sYXJnZSwgYWVzKFgxLFgyLCBjb2xvciA9IGFzLmZhY3Rvcihncm91cCkpKSArCiAgZ2VvbV9wb2ludCgpICsKICB4bGFiKCdVTUFQMScpICsgeWxhYignVU1BUDInKSArIGdndGl0bGUoJ0tpcjIuMSBsYXJnZSBkYXRhc2V0JykKcGxvdF9raXIyMV91bWFwCmBgYAojU3VwcGxlbWVudGFyeSBGaWd1cmUgNWMsZgpgYGB7ciBTdXBwbGVtZW50YXJ5IEZpZ3VyZSA1YyAmIGYsIHdhcm5pbmc9RkFMU0UsIG1lc3NhZ2U9RkFMU0V9CmZpbmFsX21vZGVsX3BlcmYgPSBkYXRhLmZyYW1lKAogICd0cmVlcycgPSBzZXEoMSw1MDAsYnkgPSAxKSwKICAnRXJyb3InID0gcGxvdChyZl9raXIyMV9qdXN0X3NlcV90cmFpbikKKQoKcDEgPSBnZ3Bsb3QoZmluYWxfbW9kZWxfcGVyZiwgYWVzKHRyZWVzLCBFcnJvcikpICsgZ2VvbV9saW5lKCkgKyB0aGVtZV9jbGFzc2ljKCkKcDIgPSBwbG90KGltcF9jdXRvZmYpCgpmaW5hbF9tb2RlbF9wZXJmID0gZ2dhcnJhbmdlKHAxLHAyLG5jb2wgPSAyLCBucm93ID0gMSkKZmluYWxfbW9kZWxfcGVyZgpgYGAKI1N1cHBsZW1lbnRhcnkgRmlndXJlIDYKYGBge3IgU3VwcGxlbWVudGFyeSBGaWd1cmUgNiwgbWVzc2FnZT1GQUxTRSwgd2FybmluZz1GQUxTRX0KCnBlcm1faGVhdG1hcCA9IGRhdGEuZnJhbWUocGVybWlzc2liaWxpdHk9cHJlZGljdGlvbnNfZGZfdXNlZnVsWywyXSxwcmVkaWN0aW9uc19kZl91c2VmdWxbLDQ6NV0pCgpwMSA9IGdncGxvdChwcmVkaWN0aW9uc19kZl91c2VmdWwsIGFlcyh4ID0gcGVybWlzc2liaWxpdHkpKSArIGdlb21fZGVuc2l0eSgpICsKICBnZ3RpdGxlKCdBY3R1YWwnKSArIHRoZW1lX2NsYXNzaWMoKSArIHhsYWIoJ2ZpdG5lc3MnKQpwMiA9IGdncGxvdChwcmVkaWN0aW9uc19kZl91c2VmdWwsIGFlcyh4ID0gcHJlZGljdGlvbnNfdGVzdCkpICsgZ2VvbV9kZW5zaXR5KCkgKwogIGdndGl0bGUoJ1ByZWRpY3RlZCcpICsgdGhlbWVfY2xhc3NpYygpICsgeGxhYignZml0bmVzcycpCnAzID0gZ2dwbG90KHByZWRpY3Rpb25zX2RmX3VzZWZ1bCwgYWVzKHggPSBwcmVkX2FjdHVhbF90ZXN0KSkgKyBnZW9tX2RlbnNpdHkoKSArCiAgZ2d0aXRsZSgnQWN0dWFsIG1pbnVzIFByZWRpY3RlZCcpICsgdGhlbWVfY2xhc3NpYygpICsgeGxhYignZml0bmVzcyBkaWZmZXJlbmNlJykKcDQgPSBnZ3Bsb3QocHJlZGljdGlvbnNfZGZfdXNlZnVsLCBhZXMocGVybWlzc2liaWxpdHkscHJlZGljdGlvbnNfdGVzdCkpICsgZ2VvbV9wb2ludCgpICsgdGhlbWVfY2xhc3NpYygpICsKICB4bGFiKCdBY3R1YWwnKSArIHlsYWIoJ1ByZWRpY3RlZCcpCgpTdXBwRmlnNiA9IGdnYXJyYW5nZShwMSxwMixwMyxwNCwKICAgICAgICAgICAgICAgICAgICAgbmNvbCA9IDQsIG5yb3cgPSAxLAogICAgICAgICAgICAgICAgICAgICBsYWJlbHMgPSBjKCdhJywnYicsJ2MnLCdkJykpCgpTdXBwRmlnNgpgYGAKI1N1cHBsZW1lbnRhcnkgRmlndXJlIDZlLWgKYGBge3IgU3VwcGxlbWVudGFyeSBGaWd1cmUgNmUtaCwgZmlnLmhlaWdodD01LCBmaWcud2lkdGg9MTAsIG1lc3NhZ2U9RkFMU0UsIHdhcm5pbmc9RkFMU0V9CnBlcm1faGVhdG1hcF9kY2FzdCA9IGRjYXN0KHByZWRpY3Rpb25zX2RmX3VzZWZ1bCxwb3NpdGlvbn5kb21haW4sdmFsdWUudmFyPSdwZXJtaXNzaWJpbGl0eScpCnJvd25hbWVzKHBlcm1faGVhdG1hcF9kY2FzdCkgPSBwZXJtX2hlYXRtYXBfZGNhc3RbLDFdCnBlcm1faGVhdG1hcF9kY2FzdCA9IHQocGVybV9oZWF0bWFwX2RjYXN0WywtMV0pCnBlcm1fcG9zX21lYW4gPSBjb2xNZWFucyhwZXJtX2hlYXRtYXBfZGNhc3QsbmEucm09VFJVRSkKcGVybV9kb21fbWVhbiA9IHJvd01lYW5zKHBlcm1faGVhdG1hcF9kY2FzdCxuYS5ybT1UUlVFKQpwcmVkX2hlYXRtYXBfZGNhc3QgPSBkY2FzdChwcmVkaWN0aW9uc19kZl91c2VmdWwscG9zaXRpb25+ZG9tYWluLHZhbHVlLnZhcj0ncHJlZGljdGlvbnNfdGVzdCcpCnJvd25hbWVzKHByZWRfaGVhdG1hcF9kY2FzdCkgPSBwcmVkX2hlYXRtYXBfZGNhc3RbLDFdCnByZWRfaGVhdG1hcF9kY2FzdCA9IHQocHJlZF9oZWF0bWFwX2RjYXN0WywtMV0pCnByZWRfcG9zX21lYW4gPSBjb2xNZWFucyhwcmVkX2hlYXRtYXBfZGNhc3QsbmEucm09VFJVRSkKcHJlZF9kb21fbWVhbiA9IHJvd01lYW5zKHByZWRfaGVhdG1hcF9kY2FzdCxuYS5ybT1UUlVFKQpwZXJtX3ByZWRfaGVhdG1hcF9kY2FzdCA9IGRjYXN0KHByZWRpY3Rpb25zX2RmX3VzZWZ1bCxwb3NpdGlvbn5kb21haW4sdmFsdWUudmFyPSdwcmVkX2FjdHVhbF90ZXN0JykKcm93bmFtZXMocGVybV9wcmVkX2hlYXRtYXBfZGNhc3QpID0gcGVybV9wcmVkX2hlYXRtYXBfZGNhc3RbLDFdCnBlcm1fcHJlZF9oZWF0bWFwX2RjYXN0ID0gdChwZXJtX3ByZWRfaGVhdG1hcF9kY2FzdFssLTFdKQpwZXJtX3ByZWRfcG9zX21lYW4gPSBjb2xNZWFucyhwZXJtX3ByZWRfaGVhdG1hcF9kY2FzdCxuYS5ybT1UUlVFKQpwZXJtX3ByZWRfZG9tX21lYW4gPSByb3dNZWFucyhwZXJtX3ByZWRfaGVhdG1hcF9kY2FzdCxuYS5ybT1UUlVFKQoKCmRvbWFpbnNfbWVhbiA9IGRhdGEuZnJhbWUocGVybV9kb209cGVybV9kb21fbWVhbixwcmVkX2RvbT1wcmVkX2RvbV9tZWFuLHBlcm1fcHJlZF9kb209cGVybV9wcmVkX2RvbV9tZWFuKQpkb21haW5zX21lYW4gPSBkYXRhLmZyYW1lKHJvd25hbWVzKGRvbWFpbnNfbWVhbiksZG9tYWluc19tZWFuKQpjb2xuYW1lcyhkb21haW5zX21lYW4pID0gYygnZG9tYWluJywncGVybV9kb21haW4nLCdwcmVkX2RvbWFpbicsJ3Blcm1fcHJlZF9kb21haW4nKQpkb21haW5zX21lYW5fbWVsdCA9IG1lbHQoZG9tYWluc19tZWFuKQoKZG9tYWluc19tZWFuX21lbHRfb3JkZXIgPSBkb21haW5zX21lYW5bb3JkZXIoZG9tYWluc19tZWFuJHBlcm1fZG9tYWluKSxdCmRvbWFpbnNfbWVhbl9tZWx0X29yZGVyJGRvbWFpbl9udW0gPSBzZXFfYWxvbmcoZG9tYWluc19tZWFuX21lbHRfb3JkZXIkZG9tYWluKQoKcDEgPSBnZ3Bsb3QoZG9tYWluc19tZWFuX21lbHRfb3JkZXIpICsgCiAgZ2VvbV9saW5lKGFlcyh4PWRvbWFpbl9udW0sIHk9cGVybV9kb21haW4pLCBzdGF0ID0gJ2lkZW50aXR5JywgY29sb3IgPSAnI0Y4NzY2RCcpICsKICBnZW9tX2xpbmUoYWVzKHg9ZG9tYWluX251bSwgeT1wZXJtX3ByZWRfZG9tYWluKSwgc3RhdCA9ICdpZGVudGl0eScsIGNvbG9yID0gJyM2MTlDRkYnKSArCiAgZ2VvbV9saW5lKGFlcyh4PWRvbWFpbl9udW0sIHk9cHJlZF9kb21haW4pLCBzdGF0ID0gJ2lkZW50aXR5JywgY29sb3IgPSAnIzAwQkEzOCcpICsKICB4bGFiKCdNb3RpZicpICsgeWxhYignRml0bmVzcycpICsKICB0aGVtZV9jbGFzc2ljMigpCgpsbV9lcW4gPSBmdW5jdGlvbihkZil7CiAgbSA9IGxtKHByZWRfZG9tYWluIH4gcGVybV9kb21haW4sIGRmKTsKICBlcSA9IHN1YnN0aXR1dGUoaXRhbGljKHkpID09IGEgKyBiICUuJSBpdGFsaWMoeCkqIiwifn5pdGFsaWMocileMn4iPSJ+cjIsIAogICAgICAgICAgICAgICAgICAgbGlzdChhID0gZm9ybWF0KHVubmFtZShjb2VmKG0pWzFdKSwgZGlnaXRzID0gMiksCiAgICAgICAgICAgICAgICAgICAgICAgIGIgPSBmb3JtYXQodW5uYW1lKGNvZWYobSlbMl0pLCBkaWdpdHMgPSAyKSwKICAgICAgICAgICAgICAgICAgICAgICAgcjIgPSBmb3JtYXQoc3VtbWFyeShtKSRyLnNxdWFyZWQsIGRpZ2l0cyA9IDMpKSkKICBhcy5jaGFyYWN0ZXIoYXMuZXhwcmVzc2lvbihlcSkpOwp9CgpwMiA9IGdncGxvdChkb21haW5zX21lYW4sIGFlcyh4PXBlcm1fZG9tYWluLCB5PXByZWRfZG9tYWluKSkgKyAKICBnZW9tX3BvaW50KCkgKyAKICBnZW9tX3Ntb290aChtZXRob2QgPSAibG0iLCBzZT1GQUxTRSwgY29sb3I9ImJsYWNrIikgKwogIGdlb21fdGV4dCh4ID0gLTAuOCwgeSA9IC0wLjQsIGxhYmVsID0gbG1fZXFuKGRvbWFpbnNfbWVhbiksIHBhcnNlID0gVFJVRSkgKwogIGNvb3JkX2ZpeGVkKCkgKwogIHlsaW0oLTEuMjUsMCkgKwogIHhsaW0oLTEuMjUsMCkgKwogIHRoZW1lX2NsYXNzaWMyKCkKCnBvc2l0aW9uc19tZWFuID0gZGF0YS5mcmFtZShwZXJtX3Bvcz1wZXJtX3Bvc19tZWFuLHByZWRfcG9zPXByZWRfcG9zX21lYW4scGVybV9wcmVkPXBlcm1fcHJlZF9wb3NfbWVhbikKcG9zaXRpb25zX21lYW4gPSBkYXRhLmZyYW1lKHJvd25hbWVzKHBvc2l0aW9uc19tZWFuKSxwb3NpdGlvbnNfbWVhbikKY29sbmFtZXMocG9zaXRpb25zX21lYW4pID0gYygncG9zaXRpb25zJywncGVybV9wb3NpdGlvbnMnLCdwcmVkX3Bvc2l0aW9ucycsJ3Blcm1fcHJlZF9wb3NpdGlvbnMnKQpwb3NpdGlvbnNfbWVhbl9tZWx0ID0gbWVsdChwb3NpdGlvbnNfbWVhbikKcG9zaXRpb25zX21lYW5fbWVsdCRwb3NpdGlvbnMgPSBhcy5udW1lcmljKGFzLmNoYXJhY3Rlcihwb3NpdGlvbnNfbWVhbl9tZWx0JHBvc2l0aW9ucykpCgpwMyA9IGdncGxvdChwb3NpdGlvbnNfbWVhbl9tZWx0LCBhZXMoeD1wb3NpdGlvbnMsIHk9dmFsdWUsIGdyb3VwPXZhcmlhYmxlLCBjb2xvcj12YXJpYWJsZSkpICsgZ2VvbV9saW5lKCkgKwogIHNjYWxlX3hfY29udGludW91cyhicmVha3MgPSBzZXEoNTAsNDAwLCBieSA9IDUwKSkgKwogIHRoZW1lKGF4aXMudGV4dC54ID0gZWxlbWVudF90ZXh0KGFuZ2xlID0gOTAsIGhqdXN0PTEpKSArIAogIHhsYWIoJ3Bvc2l0aW9ucycpICsgeWxhYignRml0bmVzcycpICsKICB0aGVtZV9jbGFzc2ljMigpCgpsbV9lcW4gPSBmdW5jdGlvbihkZil7CiAgbSA9IGxtKHByZWRfcG9zaXRpb25zIH4gcGVybV9wb3NpdGlvbnMsIGRmKTsKICBlcSA9IHN1YnN0aXR1dGUoaXRhbGljKHkpID09IGEgKyBiICUuJSBpdGFsaWMoeCkqIiwifn5pdGFsaWMocileMn4iPSJ+cjIsIAogICAgICAgICAgICAgICAgICAgbGlzdChhID0gZm9ybWF0KHVubmFtZShjb2VmKG0pWzFdKSwgZGlnaXRzID0gMiksCiAgICAgICAgICAgICAgICAgICAgICAgIGIgPSBmb3JtYXQodW5uYW1lKGNvZWYobSlbMl0pLCBkaWdpdHMgPSAyKSwKICAgICAgICAgICAgICAgICAgICAgICAgcjIgPSBmb3JtYXQoc3VtbWFyeShtKSRyLnNxdWFyZWQsIGRpZ2l0cyA9IDMpKSkKICBhcy5jaGFyYWN0ZXIoYXMuZXhwcmVzc2lvbihlcSkpOwp9CgpwNCA9IGdncGxvdChwb3NpdGlvbnNfbWVhbiwgYWVzKHg9cGVybV9wb3NpdGlvbnMsIHk9cHJlZF9wb3NpdGlvbnMpKSArIAogIGdlb21fcG9pbnQoKSArIAogIGdlb21fc21vb3RoKG1ldGhvZCA9ICJsbSIsIHNlPUZBTFNFLCBjb2xvcj0iYmxhY2siKSArCiAgZ2VvbV90ZXh0KHggPSAtMC44LCB5ID0gMC4zLCBsYWJlbCA9IGxtX2Vxbihwb3NpdGlvbnNfbWVhbiksIHBhcnNlID0gVFJVRSkgKwogIGNvb3JkX2ZpeGVkKCkgKwogIHRoZW1lX2NsYXNzaWMyKCkKCmdnYXJyYW5nZShwMSxwMixwMyxwNCwKICAgICAgICAgIG5jb2wgPSAyLCBucm93ID0gMiwKICAgICAgICAgIGxhYmVscyA9IGMoJ2UnLCdmJywnZycsJ2gnKSkKYGBgCgojRmlndXJlIDVhCmBgYHtyIEZpZ3VyZSA1YSwgbWVzc2FnZT1GQUxTRSwgd2FybmluZz1GQUxTRX0KZmVhdHVyZV9pbXBvcnRhbmNlID0gaW1wX2N1dG9mZiRyZXN1bHRzCmZlYXR1cmVfb3JkZXIgPSBjKCdiZXRhX3N0YXJ0XzExJywKICAgICAgICAgICAgICAgICAgJ2NvbnRhY3RfZGVuc2l0eScsCiAgICAgICAgICAgICAgICAgICdkX0FBX3ZvbF9uXzcnLAogICAgICAgICAgICAgICAgICAnZF9BQV9oeWRyb3Bob2JfbWVhbicsCiAgICAgICAgICAgICAgICAgICdkb21haW5fbGVuZ3RoJwogICAgICAgICAgICAgICAgICAsJ2RfQUFfbmVnYXRfbWVhbicsCiAgICAgICAgICAgICAgICAgICdwaGlfbWlkXzExJywKICAgICAgICAgICAgICAgICAgJ3BvbGFyc2FzYV9zdGFydF8xMScsCiAgICAgICAgICAgICAgICAgICdybXNmXzNqeWMnLAogICAgICAgICAgICAgICAgICAnc3RpZmZuZXNzX21pZF8xMScpCmZlYXR1cmVfaW1wb3J0YW5jZSRmZWF0dXJlID0gZmFjdG9yKGZlYXR1cmVfaW1wb3J0YW5jZSRmZWF0dXJlLGxldmVscz1mZWF0dXJlX29yZGVyKQoKI2dncGxvdChmZWF0dXJlX2ltcG9ydGFuY2UsIGFlcyh4ID0gZmVhdHVyZSwgeSA9IGltcG9ydGFuY2UpKSArIAojICBnZW9tX2NvbChmaWxsPScjMDA4NDQwJykrdGhlbWVfY2xhc3NpYygpICsKIyAgdGhlbWUoYXhpcy50aWNrcyA9IGVsZW1lbnRfYmxhbmsoKSwgYXhpcy50ZXh0LnggPSBlbGVtZW50X3RleHQoYW5nbGUgPSA5MCwgaGp1c3Q9MSkpCgpnZ3Bsb3QoZGF0YSA9IGZlYXR1cmVfaW1wb3J0YW5jZSwgYWVzKHggPSBmZWF0dXJlLCB5ID0gMSkpICsgCiAgZ2VvbV90aWxlKGFlcyhmaWxsID0gaW1wb3J0YW5jZSksIGNvbG9yID0gIndoaXRlIiwgc2l6ZSA9IDEpICsgCiAgc2NhbGVfZmlsbF9ncmFkaWVudChsb3cgPSAiZ3JheTk1IiwgaGlnaCA9ICJ0b21hdG8iLCBsaW1pdHMgPSBjKDEsIG1heChmZWF0dXJlX2ltcG9ydGFuY2UkaW1wb3J0YW5jZSkpKSArCiAgdGhlbWUoYXhpcy50ZXh0LnggPSBlbGVtZW50X3RleHQoYW5nbGUgPSA5MCkpICsKICB5bGFiKCdJbXBvcnRhbmNlJykKCmBgYAojRmlndXJlIDViLWUKYGBge3IgRmlndXJlIDViLWUsIGZpZy5oZWlnaHQ9MiwgZmlnLndpZHRoPTUsIG1lc3NhZ2U9RkFMU0UsIHdhcm5pbmc9RkFMU0V9CmFsZV9jb250YWN0X2RlbnNpdHkgPSBGZWF0dXJlRWZmZWN0JG5ldyhwcmVkaWN0b3JfY3V0b2ZmLCBmZWF0dXJlID0gImNvbnRhY3RfZGVuc2l0eSIpCmFsZV9jb250YWN0X2RlbnNpdHlfcGxvdCA9IGFsZV9jb250YWN0X2RlbnNpdHkkcGxvdCgpICsgdGhlbWVfY2xhc3NpYygpCgphbGVfZF9BQV9oeWRyb3Bob2JfbWVhbiA9IEZlYXR1cmVFZmZlY3QkbmV3KHByZWRpY3Rvcl9jdXRvZmYsIGZlYXR1cmUgPSAiZF9BQV9oeWRyb3Bob2JfbWVhbiIpCmFsZV9kX0FBX2h5ZHJvcGhvYl9tZWFuX3Bsb3QgPSBhbGVfZF9BQV9oeWRyb3Bob2JfbWVhbiRwbG90KCkgKyB0aGVtZV9jbGFzc2ljKCkKCmFsZV9kb21haW5fbGVuZ3RoID0gRmVhdHVyZUVmZmVjdCRuZXcocHJlZGljdG9yX2N1dG9mZiwgZmVhdHVyZSA9ICJkb21haW5fbGVuZ3RoIikKYWxlX2RvbWFpbl9sZW5ndGhfcGxvdCA9IGFsZV9kb21haW5fbGVuZ3RoJHBsb3QoKSArIHRoZW1lX2NsYXNzaWMoKQoKYWxlX3Jtc2ZfM2p5YyA9IEZlYXR1cmVFZmZlY3QkbmV3KHByZWRpY3Rvcl9jdXRvZmYsIGZlYXR1cmUgPSAicm1zZl8zanljIikKYWxlX3Jtc2ZfM2p5Y19wbG90ID0gYWxlX3Jtc2ZfM2p5YyRwbG90KCkgKyB0aGVtZV9jbGFzc2ljKCkKCmZpZ3VyZV9BTEVwbG90ID0gZ2dhcnJhbmdlKGFsZV9jb250YWN0X2RlbnNpdHlfcGxvdCwgCiAgICAgICAgICAgICAgICAgICAgICAgICAgIGFsZV9kX0FBX2h5ZHJvcGhvYl9tZWFuX3Bsb3QsCiAgICAgICAgICAgICAgICAgICAgICAgICAgIGFsZV9kb21haW5fbGVuZ3RoX3Bsb3QsCiAgICAgICAgICAgICAgICAgICAgICAgICAgIGFsZV9ybXNmXzNqeWNfcGxvdCwKICAgICAgICAgICAgICAgICAgICAgICAgICAgbGFiZWxzID0gYygnYicsJ2MnLCdkJywnZScpLAogICAgICAgICAgICAgICAgICAgICAgICAgICBuY29sID0gNCwgbnJvdyA9IDEpCmZpZ3VyZV9BTEVwbG90CmBgYAojU3VwcGxlbWVudGFyeSBGaWd1cmUgNwpgYGB7ciBTdXBwbGVtZW50YXJ5IEZpZ3VyZSA3LCBmaWcuaGVpZ2h0PTUsIGZpZy53aWR0aD01LCBtZXNzYWdlPUZBTFNFLCB3YXJuaW5nPUZBTFNFfQoKYWxlX2RfQUFfbmVnYXRfbWVhbiA9IEZlYXR1cmVFZmZlY3QkbmV3KHByZWRpY3Rvcl9jdXRvZmYsIGZlYXR1cmUgPSAiZF9BQV9uZWdhdF9tZWFuIikKYWxlX2RfQUFfbmVnYXRfbWVhbl9wbG90ID0gYWxlX2RfQUFfbmVnYXRfbWVhbiRwbG90KCkgKyB0aGVtZV9jbGFzc2ljKCkKCmFsZV9kX0FBX3ZvbF9uXzcgPSBGZWF0dXJlRWZmZWN0JG5ldyhwcmVkaWN0b3JfY3V0b2ZmLCBmZWF0dXJlID0gImRfQUFfdm9sX25fNyIpCmFsZV9kX0FBX3ZvbF9uXzdfcGxvdCA9IGFsZV9kX0FBX3ZvbF9uXzckcGxvdCgpICsgdGhlbWVfY2xhc3NpYygpCgphbGVfcG9sYXJzYXNhX3N0YXJ0XzExID0gRmVhdHVyZUVmZmVjdCRuZXcocHJlZGljdG9yX2N1dG9mZiwgZmVhdHVyZSA9ICJwb2xhcnNhc2Ffc3RhcnRfMTEiKQphbGVfcG9sYXJzYXNhX3N0YXJ0XzExX3Bsb3QgPSBhbGVfcG9sYXJzYXNhX3N0YXJ0XzExJHBsb3QoKSt0aGVtZV9jbGFzc2ljKCkKCmFsZV9waGlfbWlkXzExID0gRmVhdHVyZUVmZmVjdCRuZXcocHJlZGljdG9yX2N1dG9mZiwgZmVhdHVyZSA9ICJwaGlfbWlkXzExIikKYWxlX3BoaV9taWRfMTFfcGxvdCA9IGFsZV9waGlfbWlkXzExJHBsb3QoKSArIHRoZW1lX2NsYXNzaWMoKQoKYWxlX3N0aWZmbmVzc19taWRfMTEgPSBGZWF0dXJlRWZmZWN0JG5ldyhwcmVkaWN0b3JfY3V0b2ZmLCBmZWF0dXJlID0gInN0aWZmbmVzc19taWRfMTEiKQphbGVfc3RpZmZuZXNzX21pZF8xMV9wbG90ID0gYWxlX3N0aWZmbmVzc19taWRfMTEkcGxvdCgpICsgdGhlbWVfY2xhc3NpYygpCgphbGVfYmV0YV9zdGFydF8xMSA9IEZlYXR1cmVFZmZlY3QkbmV3KHByZWRpY3Rvcl9jdXRvZmYsIGZlYXR1cmUgPSAiYmV0YV9zdGFydF8xMSIpCmFsZV9iZXRhX3N0YXJ0XzExX3Bsb3QgPSBhbGVfYmV0YV9zdGFydF8xMSRwbG90KCkgKyB0aGVtZV9jbGFzc2ljKCkKCmZpZ3VyZV9BTEVwbG90X3N1cHAgPSBnZ3B1YnI6OmdnYXJyYW5nZShhbGVfZF9BQV9oeWRyb3Bob2JfbWVhbl9wbG90LCAKICAgICAgICAgICAgICAgICAgICAgICAgICAgICAgICAgICAgICAgIGFsZV9kX0FBX25lZ2F0X21lYW5fcGxvdCwKICAgICAgICAgICAgICAgICAgICAgICAgICAgICAgICAgICAgICAgIGFsZV9ybXNmXzNqeWNfcGxvdCwKICAgICAgICAgICAgICAgICAgICAgICAgICAgICAgICAgICAgICAgIGFsZV9waGlfbWlkXzExX3Bsb3QsCiAgICAgICAgICAgICAgICAgICAgICAgICAgICAgICAgICAgICAgICBhbGVfY29udGFjdF9kZW5zaXR5X3Bsb3QsCiAgICAgICAgICAgICAgICAgICAgICAgICAgICAgICAgICAgICAgICBhbGVfYmV0YV9zdGFydF8xMV9wbG90LAogICAgICAgICAgICAgICAgICAgICAgICAgICAgICAgICAgICAgICAgYWxlX3N0aWZmbmVzc19taWRfMTFfcGxvdCwKICAgICAgICAgICAgICAgICAgICAgICAgICAgICAgICAgICAgICAgIGFsZV9kb21haW5fbGVuZ3RoX3Bsb3QsCiAgICAgICAgICAgICAgICAgICAgICAgICAgICAgICAgICAgICAgICBhbGVfZF9BQV92b2xfbl83X3Bsb3QsCiAgICAgICAgICAgICAgICAgICAgICAgICAgICAgICAgICAgICAgICBhbGVfcG9sYXJzYXNhX3N0YXJ0XzExX3Bsb3QsCiAgICAgICAgICAgICAgICAgICAgICAgICAgICAgICAgICAgICAgICBsYWJlbHMgPSBjKCdhJywnYicsJ2MnLCdkJywKICAgICAgICAgICAgICAgICAgICAgICAgICAgICAgICAgICAgICAgICAgICAgICAgICAgJ2UnLCdmJywnZycsJ2gnLCdpJywnaicpLAogICAgICAgICAgICAgICAgICAgICAgICAgICAgICAgICAgICAgICAgbmNvbCA9IDMsIG5yb3cgPSA0KQpmaWd1cmVfQUxFcGxvdF9zdXBwCmBgYAoKI0ZpZ3VyZSA2YQpgYGB7ciBGaWd1cmUgNmEsIG1lc3NhZ2U9RkFMU0UsIHdhcm5pbmc9RkFMU0V9CiNvcmRlciBvZiBwbG90dGluZyAob3ZlcmFsbCBvbiB0b3ApCmZlYXR1cmVfMV9sZXZlbHMgPSBjKCdkX0FBX2h5ZHJvcGhvYl9tZWFuJywgJ2RfQUFfbmVnYXRfbWVhbicsICdkX0FBX3ZvbF9uXzcnLCAnZG9tYWluX2xlbmd0aCcsICdiZXRhX3N0YXJ0XzExJywKICAgICAgICAgICAgICAgICAgICAgJ2NvbnRhY3RfZGVuc2l0eScsICAncGhpX21pZF8xMScsICAncG9sYXJzYXNhX3N0YXJ0XzExJywgJ3Jtc2ZfM2p5YycsICdzdGlmZm5lc3NfbWlkXzExJywgJ292ZXJhbGwnKQpmZWF0dXJlXzJfbGV2ZWxzID0gYygnb3ZlcmFsbCcsJ2JldGFfc3RhcnRfMTEnLCAnY29udGFjdF9kZW5zaXR5JywgJ3BoaV9taWRfMTEnLCAncG9sYXJzYXNhX3N0YXJ0XzExJywgJ3Jtc2ZfM2p5YycsCiAgICAgICAgICAgICAgICAgICAgICdzdGlmZm5lc3NfbWlkXzExJywgJ2RfQUFfaHlkcm9waG9iX21lYW4nLCAnZF9BQV9uZWdhdF9tZWFuJywgJ2RfQUFfdm9sX25fNycsJ2RvbWFpbl9sZW5ndGgnKQppbnRlcmFjdGlvbnNfaGVhdG1hcCRmZWF0dXJlXzEgPSBmYWN0b3IoaW50ZXJhY3Rpb25zX2hlYXRtYXAkZmVhdHVyZV8xLCBsZXZlbHMgPSBmZWF0dXJlXzFfbGV2ZWxzKQppbnRlcmFjdGlvbnNfaGVhdG1hcCRmZWF0dXJlXzIgPSBmYWN0b3IoaW50ZXJhY3Rpb25zX2hlYXRtYXAkZmVhdHVyZV8yLCBsZXZlbHMgPSBmZWF0dXJlXzJfbGV2ZWxzKQpnZ3Bsb3QoZGF0YSA9IGludGVyYWN0aW9uc19oZWF0bWFwLCBhZXMoeCA9IGZlYXR1cmVfMiwgeSA9IGZlYXR1cmVfMSkpICsgCiAgZ2VvbV90aWxlKGFlcyhmaWxsID0gaW50ZXJhY3Rpb25fc3RyZW5ndGgpLCBjb2xvciA9ICd3aGl0ZScsIHNpemUgPSAxKSArIAogIHNjYWxlX2ZpbGxfZ3JhZGllbnQobG93ID0gImdyYXk5NSIsIGhpZ2ggPSAidG9tYXRvIikgKyAKICB4bGFiKCdGZWF0dXJlXzInKSArIAogIHRoZW1lX2dyZXkoYmFzZV9zaXplID0gMTIpICsgCiAgZ2d0aXRsZSgnSW50ZXJhY3Rpb24gU3RyZW5ndGggQmV0d2VlbiBGZWF0dXJlcycpICsgCiAgdGhlbWUoYXhpcy50aWNrcyA9IGVsZW1lbnRfYmxhbmsoKSwgYXhpcy50ZXh0LnggPSBlbGVtZW50X3RleHQoYW5nbGUgPSA5MCwgaGp1c3Q9MSksCiAgICAgICAgcGFuZWwuYmFja2dyb3VuZCA9IGVsZW1lbnRfYmxhbmsoKSwgCiAgICAgICAgcGxvdC50aXRsZSA9IGVsZW1lbnRfdGV4dChzaXplID0gMTIsIGNvbG91ciA9ICJncmF5NTAiKSkgCgoKYGBgCiNGaWd1cmUgNmItZApgYGB7ciBGaWd1cmUgNmItZCwgZmlnLmhlaWdodD0yLCBmaWcud2lkdGg9NywgbWVzc2FnZT1GQUxTRSwgd2FybmluZz1GQUxTRX0KI3R3byB3YXkgcGxvdHMKYWxlX2RfaHlkcm9waG9iX3N0aWZmbmVzcyA9IEZlYXR1cmVFZmZlY3QkbmV3KHByZWRpY3Rvcl9jdXRvZmYsIGZlYXR1cmUgPSBjKCJkX0FBX2h5ZHJvcGhvYl9tZWFuIiwgInN0aWZmbmVzc19taWRfMTEiKSkKYWxlX2RfaHlkcm9waG9iX3N0aWZmbmVzc19wbG90ID0gYWxlX2RfaHlkcm9waG9iX3N0aWZmbmVzcyRwbG90KCkgKyAKICBzY2FsZV9maWxsX2dyYWRpZW50MihoaWdoID0gJyNiMjE4MmInLCBtaWQgPSAnI2Y3ZjdmNycsIGxvdyA9ICcjMjE2NmFjJykgKwogIHRoZW1lX2NsYXNzaWMoKQoKYWxlX2RfaHlkcm9waG9iX2RvbWFpbl9sZW5ndGggPSBGZWF0dXJlRWZmZWN0JG5ldyhwcmVkaWN0b3JfY3V0b2ZmLCBmZWF0dXJlID0gYygiZF9BQV9oeWRyb3Bob2JfbWVhbiIsICJkb21haW5fbGVuZ3RoIikpCmFsZV9kX2h5ZHJvcGhvYl9kb21haW5fbGVuZ3RoX3Bsb3QgPSBhbGVfZF9oeWRyb3Bob2JfZG9tYWluX2xlbmd0aCRwbG90KCkgKyAKICBzY2FsZV9maWxsX2dyYWRpZW50MihoaWdoID0gJyNiMjE4MmInLCBtaWQgPSAnI2Y3ZjdmNycsIGxvdyA9ICcjMjE2NmFjJykgKwogIHRoZW1lX2NsYXNzaWMoKQoKYWxlX3BoaV9zdGlmZm5lc3MgPSBGZWF0dXJlRWZmZWN0JG5ldyhwcmVkaWN0b3JfY3V0b2ZmLCBmZWF0dXJlID0gYygicGhpX21pZF8xMSIsICJzdGlmZm5lc3NfbWlkXzExIikpCmFsZV9waGlfc3RpZmZuZXNzX3Bsb3QgPSBhbGVfcGhpX3N0aWZmbmVzcyRwbG90KCkgKyAKICBzY2FsZV9maWxsX2dyYWRpZW50MihoaWdoID0gJyNiMjE4MmInLCBtaWQgPSAnI2Y3ZjdmNycsIGxvdyA9ICcjMjE2NmFjJykgKwogIHRoZW1lX2NsYXNzaWMoKQoKZmlndXJlNl90d293YXkgPSBnZ2FycmFuZ2UoYWxlX3BoaV9zdGlmZm5lc3NfcGxvdCwgCiAgICAgICAgICAgICAgICAgICAgICAgICAgIGFsZV9kX2h5ZHJvcGhvYl9kb21haW5fbGVuZ3RoX3Bsb3QsCiAgICAgICAgICAgICAgICAgICAgICAgICAgIGFsZV9kX2h5ZHJvcGhvYl9zdGlmZm5lc3NfcGxvdCwKICAgICAgICAgICAgICAgICAgICAgICAgICAgbGFiZWxzID0gYygnYicsJ2MnLCdkJyksCiAgICAgICAgICAgICAgICAgICAgICAgICAgIG5jb2wgPSAzLCBucm93ID0gMSkKZmlndXJlNl90d293YXkKYGBgCgojU3VwcGxlbWVudGFyeSBGaWd1cmUgMTEKYGBge3IgU3VwcGxlbWVudGFyeSBGaWd1cmUgMTEsIGZpZy5oZWlnaHQ9MywgZmlnLndpZHRoPTUsIG1lc3NhZ2U9RkFMU0UsIHdhcm5pbmc9RkFMU0V9CmFsZV9wb2xhcnNhc2Ffc3RpZmZuZXNzID0gRmVhdHVyZUVmZmVjdCRuZXcocHJlZGljdG9yX2N1dG9mZiwgZmVhdHVyZSA9IGMoInBvbGFyc2FzYV9zdGFydF8xMSIsICJzdGlmZm5lc3NfbWlkXzExIikpCmFsZV9wb2xhcnNhc2Ffc3RpZmZuZXNzX3Bsb3QgPSBhbGVfcG9sYXJzYXNhX3N0aWZmbmVzcyRwbG90KCkgKyAKICBzY2FsZV9maWxsX2dyYWRpZW50MihoaWdoID0gJyNiMjE4MmInLCBtaWQgPSAnI2Y3ZjdmNycsIGxvdyA9ICcjMjE2NmFjJykgKwogIHRoZW1lX2NsYXNzaWMoKQoKc3VwcGZpZ3VyZTExX3R3b3dheSA9IGdnYXJyYW5nZShhbGVfcGhpX3N0aWZmbmVzc19wbG90LCAKICAgICAgICAgICAgICAgICAgICAgICAgICAgICAgICBhbGVfcG9sYXJzYXNhX3N0aWZmbmVzc19wbG90LAogICAgICAgICAgICAgICAgICAgICAgICAgICAgICAgIGxhYmVscyA9IGMoJ2EnLCdiJyksCiAgICAgICAgICAgICAgICAgICAgICAgICAgICAgICAgbmNvbCA9IDIsIG5yb3cgPSAxKQpzdXBwZmlndXJlMTFfdHdvd2F5CmBgYAoKI1N1cHBsZW1lbnRhcnkgRmlndXJlIDEwLTEyCmBgYHtyIFN1cHBsZW1lbnRhcnkgRmlndXJlIDEwLTEyLCBmaWcuaGVpZ2h0PTE5LCBmaWcud2lkdGg9OCwgbWVzc2FnZT1GQUxTRSwgd2FybmluZz1GQUxTRX0KYWxlX2RfaHlkcm9waG9iX2NkZWdyZWUgPSBGZWF0dXJlRWZmZWN0JG5ldyhwcmVkaWN0b3JfY3V0b2ZmLCBmZWF0dXJlID0gYygiZF9BQV9oeWRyb3Bob2JfbWVhbiIsICJjb250YWN0X2RlbnNpdHkiKSkKYWxlX2RfaHlkcm9waG9iX2NkZWdyZWVfcGxvdCA9IGFsZV9kX2h5ZHJvcGhvYl9jZGVncmVlJHBsb3QoKSArIAogIHNjYWxlX2ZpbGxfZ3JhZGllbnQyKGhpZ2ggPSAnI2IyMTgyYicsIG1pZCA9ICcjZjdmN2Y3JywgbG93ID0gJyMyMTY2YWMnKSArCiAgdGhlbWVfY2xhc3NpYygpCgphbGVfZF9oeWRyb3Bob2JfYmV0YV9zdGFydF8xMSA9IEZlYXR1cmVFZmZlY3QkbmV3KHByZWRpY3Rvcl9jdXRvZmYsIGZlYXR1cmUgPSBjKCJkX0FBX2h5ZHJvcGhvYl9tZWFuIiwgImJldGFfc3RhcnRfMTEiKSkKYWxlX2RfaHlkcm9waG9iX2JldGFfc3RhcnRfMTFfcGxvdCA9IGFsZV9kX2h5ZHJvcGhvYl9iZXRhX3N0YXJ0XzExJHBsb3QoKSArIAogIHNjYWxlX2ZpbGxfZ3JhZGllbnQyKGhpZ2ggPSAnI2IyMTgyYicsIG1pZCA9ICcjZjdmN2Y3JywgbG93ID0gJyMyMTY2YWMnKSArCiAgdGhlbWVfY2xhc3NpYygpCgphbGVfZF9oeWRyb3Bob2JfcG9sYXJzYXNhX3N0YXJ0XzExID0gRmVhdHVyZUVmZmVjdCRuZXcocHJlZGljdG9yX2N1dG9mZiwgZmVhdHVyZSA9IGMoImRfQUFfaHlkcm9waG9iX21lYW4iLCAicG9sYXJzYXNhX3N0YXJ0XzExIikpCmFsZV9kX2h5ZHJvcGhvYl9wb2xhcnNhc2Ffc3RhcnRfMTFfcGxvdCA9IGFsZV9kX2h5ZHJvcGhvYl9wb2xhcnNhc2Ffc3RhcnRfMTEkcGxvdCgpICsgCiAgc2NhbGVfZmlsbF9ncmFkaWVudDIoaGlnaCA9ICcjYjIxODJiJywgbWlkID0gJyNmN2Y3ZjcnLCBsb3cgPSAnIzIxNjZhYycpICsKICB0aGVtZV9jbGFzc2ljKCkKCmFsZV9kX3Jtc2ZfM2p5Y19oeWRyb3Bob2IgPSBGZWF0dXJlRWZmZWN0JG5ldyhwcmVkaWN0b3JfY3V0b2ZmLCBmZWF0dXJlID0gYygiZF9BQV9oeWRyb3Bob2JfbWVhbiIsICJybXNmXzNqeWMiKSkKYWxlX2Rfcm1zZl8zanljX2h5ZHJvcGhvYl9wbG90ID0gYWxlX2Rfcm1zZl8zanljX2h5ZHJvcGhvYiRwbG90KCkgKyAKICBzY2FsZV9maWxsX2dyYWRpZW50MihoaWdoID0gJyNiMjE4MmInLCBtaWQgPSAnI2Y3ZjdmNycsIGxvdyA9ICcjMjE2NmFjJykgKwogIHRoZW1lX2NsYXNzaWMoKQoKYWxlX2Rfdm9sX25fN19kb21haW5fbGVuZ3RoID0gRmVhdHVyZUVmZmVjdCRuZXcocHJlZGljdG9yX2N1dG9mZiwgZmVhdHVyZSA9IGMoImRfQUFfdm9sX25fNyIsICJkb21haW5fbGVuZ3RoIikpCmFsZV9kX3ZvbF9uXzdfZG9tYWluX2xlbmd0aF9wbG90ID0gYWxlX2Rfdm9sX25fN19kb21haW5fbGVuZ3RoJHBsb3QoKSArIAogIHNjYWxlX2ZpbGxfZ3JhZGllbnQyKGhpZ2ggPSAnI2IyMTgyYicsIG1pZCA9ICcjZjdmN2Y3JywgbG93ID0gJyMyMTY2YWMnKSArCiAgdGhlbWVfY2xhc3NpYygpCgphbGVfZF9oeWRyb3Bob2JfZG9tYWluX25lZ2F0ID0gRmVhdHVyZUVmZmVjdCRuZXcocHJlZGljdG9yX2N1dG9mZiwgZmVhdHVyZSA9IGMoImRfQUFfaHlkcm9waG9iX21lYW4iLCAiZF9BQV9uZWdhdF9tZWFuIikpCmFsZV9kX2h5ZHJvcGhvYl9kb21haW5fbmVnYXRfcGxvdCA9IGFsZV9kX2h5ZHJvcGhvYl9kb21haW5fbmVnYXQkcGxvdCgpICsgCiAgc2NhbGVfZmlsbF9ncmFkaWVudDIoaGlnaCA9ICcjYjIxODJiJywgbWlkID0gJyNmN2Y3ZjcnLCBsb3cgPSAnIzIxNjZhYycpICsKICB0aGVtZV9jbGFzc2ljKCkKCmFsZV9kX3N0aWZmbmVzc19taWRfMTFfZG9tYWluX2xlbmd0aCA8LSBGZWF0dXJlRWZmZWN0JG5ldyhwcmVkaWN0b3JfY3V0b2ZmLCBmZWF0dXJlID0gYygic3RpZmZuZXNzX21pZF8xMSIsICJkb21haW5fbGVuZ3RoIikpCmFsZV9kX3N0aWZmbmVzc19taWRfMTFfZG9tYWluX2xlbmd0aF9wbG90ID0gYWxlX2Rfc3RpZmZuZXNzX21pZF8xMV9kb21haW5fbGVuZ3RoJHBsb3QoKSArIAogIHNjYWxlX2ZpbGxfZ3JhZGllbnQyKGhpZ2ggPSAnI2IyMTgyYicsIG1pZCA9ICcjZjdmN2Y3JywgbG93ID0gJyMyMTY2YWMnKSArCiAgdGhlbWVfY2xhc3NpYygpCgphbGVfZF9uZWdhdF9kb21haW5fbGVuZ3RoID0gRmVhdHVyZUVmZmVjdCRuZXcocHJlZGljdG9yX2N1dG9mZiwgZmVhdHVyZSA9IGMoImRfQUFfbmVnYXRfbWVhbiIsICJkb21haW5fbGVuZ3RoIikpCmFsZV9kX25lZ2F0X2RvbWFpbl9sZW5ndGhfcGxvdCA9IGFsZV9kX25lZ2F0X2RvbWFpbl9sZW5ndGgkcGxvdCgpICsgCiAgc2NhbGVfZmlsbF9ncmFkaWVudDIoaGlnaCA9ICcjYjIxODJiJywgbWlkID0gJyNmN2Y3ZjcnLCBsb3cgPSAnIzIxNjZhYycpICsKICB0aGVtZV9jbGFzc2ljKCkKCmFsZV9kX3Jtc2ZfM2p5Y19kb21haW5fbGVuZ3RoID0gRmVhdHVyZUVmZmVjdCRuZXcocHJlZGljdG9yX2N1dG9mZiwgZmVhdHVyZSA9IGMoInJtc2ZfM2p5YyIsICJkb21haW5fbGVuZ3RoIikpCmFsZV9kX3Jtc2ZfM2p5Y19kb21haW5fbGVuZ3RoX3Bsb3QgPSBhbGVfZF9ybXNmXzNqeWNfZG9tYWluX2xlbmd0aCRwbG90KCkgKyAKICBzY2FsZV9maWxsX2dyYWRpZW50MihoaWdoID0gJyNiMjE4MmInLCBtaWQgPSAnI2Y3ZjdmNycsIGxvdyA9ICcjMjE2NmFjJykgKwogIHRoZW1lX2NsYXNzaWMoKQoKCmFsZV9kX2NkZWdyZWVfZG9tYWluX2xlbmd0aCA8LSBGZWF0dXJlRWZmZWN0JG5ldyhwcmVkaWN0b3JfY3V0b2ZmLCBmZWF0dXJlID0gYygiY29udGFjdF9kZW5zaXR5IiwgImRvbWFpbl9sZW5ndGgiKSkKYWxlX2RfY2RlZ3JlZV9kb21haW5fbGVuZ3RoX3Bsb3QgPSBhbGVfZF9jZGVncmVlX2RvbWFpbl9sZW5ndGgkcGxvdCgpICsgCiAgc2NhbGVfZmlsbF9ncmFkaWVudDIoaGlnaCA9ICcjYjIxODJiJywgbWlkID0gJyNmN2Y3ZjcnLCBsb3cgPSAnIzIxNjZhYycpICsKICB0aGVtZV9jbGFzc2ljKCkKCnN1cHBmaWd1cmUxMF90d293YXkgPSBnZ2FycmFuZ2UoYWxlX2RfaHlkcm9waG9iX3N0aWZmbmVzc19wbG90LAogICAgICAgICAgICAgICAgICAgICAgICAgICAgICAgIGFsZV9kX2h5ZHJvcGhvYl9kb21haW5fbGVuZ3RoX3Bsb3QsCiAgICAgICAgICAgICAgICAgICAgICAgICAgICAgICAgYWxlX2RfaHlkcm9waG9iX2NkZWdyZWVfcGxvdCwKICAgICAgICAgICAgICAgICAgICAgICAgICAgICAgICBhbGVfZF9oeWRyb3Bob2JfYmV0YV9zdGFydF8xMV9wbG90LAogICAgICAgICAgICAgICAgICAgICAgICAgICAgICAgIGFsZV9kX2h5ZHJvcGhvYl9wb2xhcnNhc2Ffc3RhcnRfMTFfcGxvdCwKICAgICAgICAgICAgICAgICAgICAgICAgICAgICAgICBhbGVfZF9ybXNmXzNqeWNfaHlkcm9waG9iX3Bsb3QsCiAgICAgICAgICAgICAgICAgICAgICAgICAgICAgICAgYWxlX2Rfdm9sX25fN19kb21haW5fbGVuZ3RoX3Bsb3QsCiAgICAgICAgICAgICAgICAgICAgICAgICAgICAgICAgYWxlX2RfaHlkcm9waG9iX2RvbWFpbl9uZWdhdF9wbG90LAogICAgICAgICAgICAgICAgICAgICAgICAgICAgICAgIGFsZV9kX3N0aWZmbmVzc19taWRfMTFfZG9tYWluX2xlbmd0aF9wbG90LAogICAgICAgICAgICAgICAgICAgICAgICAgICAgICAgIGFsZV9kX25lZ2F0X2RvbWFpbl9sZW5ndGhfcGxvdCwKICAgICAgICAgICAgICAgICAgICAgICAgICAgICAgICBhbGVfZF9ybXNmXzNqeWNfZG9tYWluX2xlbmd0aF9wbG90LAogICAgICAgICAgICAgICAgICAgICAgICAgICAgICAgIGFsZV9kX2NkZWdyZWVfZG9tYWluX2xlbmd0aF9wbG90LAogICAgICAgICAgICAgICAgICAgICAgICAgICAgICAgIGxhYmVscyA9IGMoJ2EnLCdiJywnYycsJ2QnLCdlJywnZicsCiAgICAgICAgICAgICAgICAgICAgICAgICAgICAgICAgICAgICAgICAgICAnZycsJ2gnLCdpJywnaicsJ2snLCdsJyksCiAgICAgICAgICAgICAgICAgICAgICAgICAgICAgICAgbmNvbCA9IDMsIG5yb3cgPSA0KQoKc3VwcGZpZ3VyZTEwX3R3b3dheQpgYGAKCiNTdXBwbGVtZW50YXJ5IEZpZ3VyZSAyICYgMTIgZm9yIEtpcjIxXzE1RApgYGB7ciBTdXBwbGVtZW50YXJ5IEZpZ3VyZSAyICYgMTIgZm9yIEtpcjIxXzE1RCwgbWVzc2FnZT1GQUxTRSwgd2FybmluZz1GQUxTRX0KI0tpcjIxXzE1IGJhc2VkIG9uIHN0cnVjdHVyZWQgbW90aWZzCgp6ID0gZGNhc3Qoa2lyMjFfMTVELCBkb21haW4gKyBnZW5lICsgcG9zaXRpb24gfiB2YXJpYWJsZSwgZnVuLmFnZ3JlZ2F0ZSA9IGZ1bmN0aW9uKHgpIG1lYW4oeCwgbmEucm0gPSBUKSkKelssJ25vcm1hbGl6ZWRfbG93QU5EaGlnaCddW3pbLCdub3JtYWxpemVkX2xvd0FORGhpZ2gnXSA9PSAwXSA9IE5BCgp6JHBlcm0gPSByZXAoTkEsIG5yb3coeikpCgpmb3IgKGkgaW4gbGV2ZWxzKHokZ2VuZSkpewogIGlkeCA9IHokZ2VuZSA9PSBpCiAgeltpZHgsJ3Blcm0nXSA9IHJhbmdlMDEoeltpZHgsJ25vcm1hbGl6ZWRfbG93QU5EaGlnaCddLHJlbW92ZS5uYSA9IFQpCn0KClN1cHBGaWcxMl9iID0gZ2dwbG90KHopICsKICBnZW9tX3RpbGUoYWVzKHg9cG9zaXRpb24seT1kb21haW4sZmlsbD1ub3JtYWxpemVkX2xvd0FORGhpZ2gpKSArCiAgc2NpY286OnNjYWxlX2ZpbGxfc2NpY28ocGFsZXR0ZSA9ICdiaWxiYW8nLCBuYS52YWx1ZSA9ICdjeWFuJykgKwogIGZhY2V0X2dyaWQoZ2VuZX4uKSArCiAgdGhlbWVfY2xhc3NpYygpICsKICBnZ3RpdGxlKCdLaXIyMScpCgpraXIyMXogPSB6CgpkID0gelt6JGdlbmUgPT0gJ0tpcjIxJyxjKCdkb21haW4nLCdwb3NpdGlvbicsJ3Blcm0nKV0KCiNyZW1vdmUgZG9tYWlucyBhbmQgcG9zaXRpb25zIG1pc3NpbmcgODAlIGRhdGEKcGVybV9waXZvdCA9IGRjYXN0KGQscG9zaXRpb25+ZG9tYWluLHZhbHVlLnZhcj0ncGVybScpCnBlcm1fcGl2b3RfcGxvdCA9IHBlcm1fcGl2b3Rbcm93TWVhbnMoaXMubmEocGVybV9waXZvdCkpIDw9IC44LF0KcGVybV9waXZvdF9wbG90ID0gcGVybV9waXZvdF9wbG90Wyxjb2xNZWFucyhpcy5uYShwZXJtX3Bpdm90X3Bsb3QpKSA8PSAuNV0KCnBlcm1fcGxvdF9tZWx0ID0gbWVsdChwZXJtX3Bpdm90X3Bsb3QsaWQudmFycz0ncG9zaXRpb24nKQpwZXJtX3Bsb3RfbWVsdCA9IG5hLm9taXQocGVybV9wbG90X21lbHQpCmNvbG5hbWVzKHBlcm1fcGxvdF9tZWx0KSA9IGMoJ3Bvc2l0aW9uJywnZG9tYWluJywncGVybWlzc2liaWxpdHknKQoKcGVybV9waXZvdF96ZXJvcyA9IHBlcm1fcGl2b3RfcGxvdApwZXJtX3Bpdm90X3plcm9zW2lzLm5hKHBlcm1fcGl2b3RfemVyb3MpXSA9IDAKCgpkZi51bWFwID0gdW1hcChwZXJtX3Bpdm90X3plcm9zWywtMV0sIAogICAgICAgICAgICAgICAgICAgICBtZXRyaWMgPSAnY29zaW5lJywKICAgICAgICAgICAgICAgICAgICAgbl9lcG9jaHMgPSA1MDAsCiAgICAgICAgICAgICAgICAgICAgIG5fbmVpZ2hib3JzID0gMjAsCiAgICAgICAgICAgICAgICAgICAgIG5uX21ldGhvZCA9ICdhbm5veScsCiAgICAgICAgICAgICAgICAgICAgIG5fdHJlZXMgPSAxMDAsCiAgICAgICAgICAgICAgICAgICAgIGJhbmR3aWR0aCA9IDAuOAopCgpzY29yZXNfa2lyMjFfMTVEID0gZGF0YS5mcmFtZShkZi51bWFwKSAjIFBDIHNjb3JlIG1hdHJpeApzY29yZXNfa2lyMjFfMTVEJGdyb3VwID0gTmJDbHVzdDo6TmJDbHVzdChzY29yZXNfa2lyMjFfMTVELCBtZXRob2QgPSAnd2FyZC5EMicpJEJlc3QucGFydGl0aW9uCnNjb3Jlc19raXIyMV8xNUQkZ2VuZSA9IHJlcCgnS2lyMjEnLCBucm93KHNjb3Jlc19raXIyMV8xNUQpKQoKU3VwcEZpZzJfYiA9IGdncGxvdChzY29yZXNfa2lyMjFfMTVELCBhZXMoWDEsWDIsIGNvbG9yID0gYXMuZmFjdG9yKGdyb3VwKSkpICsKICBnZW9tX3BvaW50KCkgKyB4bGFiKCdVTUFQMScpICsgeWxhYignVU1BUDInKSArIGdndGl0bGUoJ0tpcjIuMSAoYmFzZWQgb24gc3RydWN0dXJhbCBtb3RpZnMnKQoKYGBgCiNTdXBwbGVtZW50YXJ5IEZpZ3VyZSAyICYgMTIgZm9yIEtpcjMuMQpgYGB7ciBTdXBwbGVtZW50YXJ5IEZpZ3VyZSAyICYgMTIgZm9yIEtpcjMuMSwgbWVzc2FnZT1GQUxTRSwgd2FybmluZz1GQUxTRX0KI0tpcjMxXzE1RAoKeiA9IGRjYXN0KGtpcjMxXzE1RCwgZG9tYWluICsgZ2VuZSArIHBvc2l0aW9uIH4gdmFyaWFibGUsIGZ1bi5hZ2dyZWdhdGUgPSBmdW5jdGlvbih4KSBtZWFuKHgsIG5hLnJtID0gVCkpCnpbLCdub3JtYWxpemVkX2xvd0FORGhpZ2gnXVt6Wywnbm9ybWFsaXplZF9sb3dBTkRoaWdoJ10gPT0gMF0gPSBOQQoKeiRwZXJtID0gcmVwKE5BLCBucm93KHopKQoKZm9yIChpIGluIGxldmVscyh6JGdlbmUpKXsKICAgIGlkeCA9IHokZ2VuZSA9PSBpCiAgICB6W2lkeCwncGVybSddID0gcmFuZ2UwMSh6W2lkeCwnbm9ybWFsaXplZF9sb3dBTkRoaWdoJ10scmVtb3ZlLm5hID0gVCkKfQoKeiRnZW5lID0gcmV2YWx1ZSh6JGdlbmUsIGMoJ0tpcjMxLTInPSdLaXIzMScsJ0tpcjMxLTMnPSdLaXIzMScsJ0tpcjMxLTQnPSdLaXIzMScsJ0tpcjMxLTUnPSdLaXIzMScpKQpraXIzMXogPSB6Cgp6ID0gbWVsdChraXIzMXosIGlkLnZhcnMgPSBjKCdnZW5lJywnZG9tYWluJywgJ3Bvc2l0aW9uJykpCnogPSBkY2FzdCh6LCBkb21haW4gKyBnZW5lICsgcG9zaXRpb24gfiB2YXJpYWJsZSwgZnVuLmFnZ3JlZ2F0ZSA9IGZ1bmN0aW9uKHgpIG1lYW4oeCwgbmEucm0gPSBUKSkKClN1cHBGaWcxMl9kID0gZ2dwbG90KHopICsKICBnZW9tX3RpbGUoYWVzKHg9cG9zaXRpb24seT1kb21haW4sZmlsbD1wZXJtKSkgKwogIHNjaWNvOjpzY2FsZV9maWxsX3NjaWNvKHBhbGV0dGUgPSAnYmlsYmFvJywgbmEudmFsdWUgPSAnY3lhbicpICsKICBmYWNldF9ncmlkKGdlbmV+LikgKwogIHRoZW1lX2NsYXNzaWMoKSsKICBnZ3RpdGxlKCdLaXIzMScpCgpkID0gelt6JGdlbmUgPT0gJ0tpcjMxJyxjKCdkb21haW4nLCdwb3NpdGlvbicsJ3Blcm0nKV0KCgojcmVtb3ZlIGRvbWFpbnMgYW5kIHBvc2l0aW9ucyBtaXNzaW5nIDgwJSBkYXRhCnBlcm1fcGl2b3QgPSBkY2FzdChkLHBvc2l0aW9ufmRvbWFpbix2YWx1ZS52YXI9J3Blcm0nKQpwZXJtX3Bpdm90X3Bsb3QgPSBwZXJtX3Bpdm90W3Jvd01lYW5zKGlzLm5hKHBlcm1fcGl2b3QpKSA8PSAuOCxdCnBlcm1fcGl2b3RfcGxvdCA9IHBlcm1fcGl2b3RfcGxvdFssY29sTWVhbnMoaXMubmEocGVybV9waXZvdF9wbG90KSkgPD0gLjVdCgpwZXJtX3Bsb3RfbWVsdCA9IG1lbHQocGVybV9waXZvdF9wbG90LGlkLnZhcnM9J3Bvc2l0aW9uJykKcGVybV9wbG90X21lbHQgPSBuYS5vbWl0KHBlcm1fcGxvdF9tZWx0KQpjb2xuYW1lcyhwZXJtX3Bsb3RfbWVsdCkgPSBjKCdwb3NpdGlvbicsJ2RvbWFpbicsJ3Blcm1pc3NpYmlsaXR5JykKCnBlcm1fcGl2b3RfemVyb3MgPSBwZXJtX3Bpdm90X3Bsb3QKcGVybV9waXZvdF96ZXJvc1tpcy5uYShwZXJtX3Bpdm90X3plcm9zKV0gPSAwCgojdW1hcApkZi51bWFwID0gdW1hcChwZXJtX3Bpdm90X3plcm9zWywtMV0sIAogICAgICAgICAgICAgICAgICAgICBtZXRyaWMgPSAnZXVjbGlkZWFuJywKICAgICAgICAgICAgICAgICAgICAgbl9lcG9jaHMgPSA1MDAsCiAgICAgICAgICAgICAgICAgICAgIG5fbmVpZ2hib3JzID0gMjAsCiAgICAgICAgICAgICAgICAgICAgIG5uX21ldGhvZCA9ICdhbm5veScsCiAgICAgICAgICAgICAgICAgICAgIG5fdHJlZXMgPSAxMDAsCiAgICAgICAgICAgICAgICAgICAgIGJhbmR3aWR0aCA9IDEKKQoKCnNjb3Jlc19LaXIzMSA9IGRhdGEuZnJhbWUoZGYudW1hcCkgIyBQQyBzY29yZSBtYXRyaXgKc2NvcmVzX0tpcjMxJGdyb3VwID0gTmJDbHVzdChzY29yZXNfS2lyMzEsIG1ldGhvZCA9ICd3YXJkLkQyJywgbWluLm5jID0gMikkQmVzdC5wYXJ0aXRpb24Kc2NvcmVzX0tpcjMxJGdlbmUgPSByZXAoJ0tpcjMxJywgbnJvdyhzY29yZXNfS2lyMzEpKQoKU3VwcEZpZzJfYyA9IGdncGxvdChzY29yZXNfS2lyMzEsIGFlcyhYMSxYMiwgY29sb3IgPSBhcy5mYWN0b3IoZ3JvdXApKSkgKwogIGdlb21fcG9pbnQoKSAgKyB4bGFiKCdVTUFQMScpICsgeWxhYignVU1BUDInKSArIGdndGl0bGUoJ0tpcjMuMScpCmBgYAoKI1N1cHBsZW1lbnRhcnkgRmlndXJlIDIgJiAxMiBmb3IgQXNpYzFhCmBgYHtyIFN1cHBsZW1lbnRhcnkgRmlndXJlIDIgJiAxMiBmb3IgQXNpYzFhLCBtZXNzYWdlPUZBTFNFLCB3YXJuaW5nPUZBTFNFfQojbG9hZCBBc2ljMWEjIyMjCgojU3VwcEZpZzEyX2YgPSBnZ3Bsb3QoYXNpYzFfMTVEKSArCiMgIGdlb21fdGlsZShhZXMoeD1wb3NpdGlvbix5PWRvbWFpbixmaWxsPXZhbHVlKSkgKwojICBzY2ljbzo6c2NhbGVfZmlsbF9zY2ljbyhwYWxldHRlID0gJ2JpbGJhbycsIG5hLnZhbHVlID0gJ2N5YW4nKSArCiMgIGZhY2V0X2dyaWQoZ2VuZX4uKSArCiMgIHRoZW1lX2NsYXNzaWMoKSArCiMgIGdndGl0bGUoJ0FzaWMxYScpCgp6ID0gZGNhc3QoYXNpYzFfMTVELCBkb21haW4gKyBnZW5lICsgcG9zaXRpb24gfiB2YXJpYWJsZSwgZnVuLmFnZ3JlZ2F0ZSA9IGZ1bmN0aW9uKHgpIG1lYW4oeCwgbmEucm0gPSBUKSkKelssJ25vcm1hbGl6ZWRfbG93QU5EaGlnaCddW3pbLCdub3JtYWxpemVkX2xvd0FORGhpZ2gnXSA9PSAwXSA9IE5BCgp6JHBlcm0gPSByZXAoTkEsIG5yb3coeikpCgoKZm9yIChpIGluIGxldmVscyh6JGdlbmUpKXsKICBpZHggPSB6JGdlbmUgPT0gaQogIHpbaWR4LCdwZXJtJ10gPSByYW5nZTAxKHpbaWR4LCdub3JtYWxpemVkX2xvd0FORGhpZ2gnXSxyZW1vdmUubmEgPSBUKQp9CgpTdXBwRmlnMTJfZiA9IGdncGxvdCh6KSArCiAgZ2VvbV90aWxlKGFlcyh4PXBvc2l0aW9uLHk9ZG9tYWluLGZpbGw9bm9ybWFsaXplZF9sb3dBTkRoaWdoKSkgKwogIHNjaWNvOjpzY2FsZV9maWxsX3NjaWNvKHBhbGV0dGUgPSAnYmlsYmFvJywgbmEudmFsdWUgPSAnY3lhbicpICsKICBmYWNldF9ncmlkKGdlbmV+LikgKwogIHRoZW1lX2NsYXNzaWMoKSArCiAgZ2d0aXRsZSgnQXNpYzFhJykKCmFzaWMxYXogPSB6CgoKI0FzaWMxYSMjIyMKCmQgPSB6W3okZ2VuZSA9PSAnQXNpYzFhJyxjKCdkb21haW4nLCdwb3NpdGlvbicsJ3Blcm0nKV0KCgojcmVtb3ZlIGRvbWFpbnMgYW5kIHBvc2l0aW9ucyBtaXNzaW5nIDgwJSBkYXRhCnBlcm1fcGl2b3Q8LWRjYXN0KGQscG9zaXRpb25+ZG9tYWluLHZhbHVlLnZhcj0ncGVybScpCnBlcm1fcGl2b3RfcGxvdDwtcGVybV9waXZvdFtyb3dNZWFucyhpcy5uYShwZXJtX3Bpdm90KSkgPD0gLjgsXQpwZXJtX3Bpdm90X3Bsb3Q8LXBlcm1fcGl2b3RfcGxvdFssY29sTWVhbnMoaXMubmEocGVybV9waXZvdF9wbG90KSkgPD0gLjVdCgpwZXJtX3Bsb3RfbWVsdDwtbWVsdChwZXJtX3Bpdm90X3Bsb3QsaWQudmFycz0ncG9zaXRpb24nKQpwZXJtX3Bsb3RfbWVsdDwtbmEub21pdChwZXJtX3Bsb3RfbWVsdCkKY29sbmFtZXMocGVybV9wbG90X21lbHQpPC1jKCdwb3NpdGlvbicsJ2RvbWFpbicsJ3Blcm1pc3NpYmlsaXR5JykKCnBlcm1fcGl2b3RfemVyb3MgPSBwZXJtX3Bpdm90X3Bsb3QKcGVybV9waXZvdF96ZXJvc1tpcy5uYShwZXJtX3Bpdm90X3plcm9zKV0gPSAwCgoKZGYudW1hcCA9IHVtYXAocGVybV9waXZvdF96ZXJvc1ssLTFdLCAKICAgICAgICAgICAgICAgICAgICAgbWV0cmljID0gJ2V1Y2xpZGVhbicsCiAgICAgICAgICAgICAgICAgICAgIG5fZXBvY2hzID0gNTAwLAogICAgICAgICAgICAgICAgICAgICBuX25laWdoYm9ycyA9IDIwLAogICAgICAgICAgICAgICAgICAgICBubl9tZXRob2QgPSAnYW5ub3knLAogICAgICAgICAgICAgICAgICAgICBuX3RyZWVzID0gMTAwLAogICAgICAgICAgICAgICAgICAgICBiYW5kd2lkdGggPSAxCikKCgoKCnNjb3Jlc19Bc2ljMWEgPSBkYXRhLmZyYW1lKGRmLnVtYXApICMgUEMgc2NvcmUgbWF0cml4CnNjb3Jlc19Bc2ljMWEkZ3JvdXAgPSBOYkNsdXN0KHNjb3Jlc19Bc2ljMWEsIG1ldGhvZCA9ICd3YXJkLkQyJywgbWluLm5jID0gMikkQmVzdC5wYXJ0aXRpb24Kc2NvcmVzX0FzaWMxYSRnZW5lID0gcmVwKCdBc2ljMWEnLCBucm93KHNjb3Jlc19Bc2ljMWEpKQoKU3VwcEZpZzJfZCA9IGdncGxvdChzY29yZXNfQXNpYzFhLCBhZXMoWDEsWDIsIGNvbG9yID0gYXMuZmFjdG9yKGdyb3VwKSkpICsKICBnZW9tX3BvaW50KCkgICsgeGxhYignVU1BUDEnKSArIHlsYWIoJ1VNQVAyJykgKyBnZ3RpdGxlKCdBc2ljMWEnKQoKCmBgYAojU3VwcGxlbWVudGFyeSBGaWd1cmUgMiAmIDEyIGZvciBQMlgzCgpgYGB7ciBTdXBwbGVtZW50YXJ5IEZpZ3VyZSAyICYgMTIgZm9yIFAyWDMsIG1lc3NhZ2U9RkFMU0UsIHdhcm5pbmc9RkFMU0V9CiNsb2FkIFAyWDMjIyMjCgoKI1N1cHBGaWcxMl9jID0gZ2dwbG90KHAyeDNfMTVEKSArCiMgIGdlb21fdGlsZShhZXMoeD1wb3NpdGlvbix5PWRvbWFpbixmaWxsPXZhbHVlKSkgKwojICBzY2ljbzo6c2NhbGVfZmlsbF9zY2ljbyhwYWxldHRlID0gJ2JpbGJhbycsIG5hLnZhbHVlID0gJ2N5YW4nKSArCiMgIGZhY2V0X2dyaWQoZ2VuZX4uKSArCiMgIHRoZW1lX2NsYXNzaWMoKSArCiMgIGdndGl0bGUoJ1AyWDMnKQoKeiA9IGRjYXN0KHAyeDNfMTVELCBkb21haW4gKyBnZW5lICsgcG9zaXRpb24gfiB2YXJpYWJsZSwgZnVuLmFnZ3JlZ2F0ZSA9IGZ1bmN0aW9uKHgpIG1lYW4oeCwgbmEucm0gPSBUKSkKelssJ25vcm1hbGl6ZWRfbG93QU5EaGlnaCddW3pbLCdub3JtYWxpemVkX2xvd0FORGhpZ2gnXSA9PSAwXSA9IE5BCgp6JHBlcm0gPSByZXAoTkEsIG5yb3coeikpCgoKZm9yIChpIGluIGxldmVscyh6JGdlbmUpKXsKICBpZHggPSB6JGdlbmUgPT0gaQogIHpbaWR4LCdwZXJtJ10gPSByYW5nZTAxKHpbaWR4LCdub3JtYWxpemVkX2xvd0FORGhpZ2gnXSxyZW1vdmUubmEgPSBUKQp9CgpTdXBwRmlnMTJfYyA9IGdncGxvdCh6KSArCiAgZ2VvbV90aWxlKGFlcyh4PXBvc2l0aW9uLHk9ZG9tYWluLGZpbGw9bm9ybWFsaXplZF9sb3dBTkRoaWdoKSkgKwogIHNjaWNvOjpzY2FsZV9maWxsX3NjaWNvKHBhbGV0dGUgPSAnYmlsYmFvJywgbmEudmFsdWUgPSAnY3lhbicpICsKICBmYWNldF9ncmlkKGdlbmV+LikgKwogIHRoZW1lX2NsYXNzaWMoKSArCiAgZ2d0aXRsZSgnUDJYMycpCgpwMngzeiA9IHoKCiNQMlgzIyMjIwoKZCA9IHpbeiRnZW5lID09ICdQMlgzJyxjKCdkb21haW4nLCdwb3NpdGlvbicsJ3Blcm0nKV0KCgojcmVtb3ZlIGRvbWFpbnMgYW5kIHBvc2l0aW9ucyBtaXNzaW5nIDgwJSBkYXRhCnBlcm1fcGl2b3Q8LWRjYXN0KGQscG9zaXRpb25+ZG9tYWluLHZhbHVlLnZhcj0ncGVybScpCnBlcm1fcGl2b3RfcGxvdDwtcGVybV9waXZvdFtyb3dNZWFucyhpcy5uYShwZXJtX3Bpdm90KSkgPD0gLjgsXQpwZXJtX3Bpdm90X3Bsb3Q8LXBlcm1fcGl2b3RfcGxvdFssY29sTWVhbnMoaXMubmEocGVybV9waXZvdF9wbG90KSkgPD0gLjhdCgpwZXJtX3Bsb3RfbWVsdDwtbWVsdChwZXJtX3Bpdm90X3Bsb3QsaWQudmFycz0ncG9zaXRpb24nKQpwZXJtX3Bsb3RfbWVsdDwtbmEub21pdChwZXJtX3Bsb3RfbWVsdCkKY29sbmFtZXMocGVybV9wbG90X21lbHQpPC1jKCdwb3NpdGlvbicsJ2RvbWFpbicsJ3Blcm1pc3NpYmlsaXR5JykKCnBlcm1fcGl2b3RfemVyb3MgPSBwZXJtX3Bpdm90X3Bsb3QKcGVybV9waXZvdF96ZXJvc1tpcy5uYShwZXJtX3Bpdm90X3plcm9zKV0gPSAwCgoKI3VtYXAKZGYudW1hcCA9IHVtYXAocGVybV9waXZvdF96ZXJvc1ssLTFdLCAKICAgICAgICAgICAgICAgICAgICAgbWV0cmljID0gJ2V1Y2xpZGVhbicsCiAgICAgICAgICAgICAgICAgICAgIG5fZXBvY2hzID0gNTAwLAogICAgICAgICAgICAgICAgICAgICBuX25laWdoYm9ycyA9IDIwLAogICAgICAgICAgICAgICAgICAgICBubl9tZXRob2QgPSAnYW5ub3knLAogICAgICAgICAgICAgICAgICAgICBuX3RyZWVzID0gMTAwLAogICAgICAgICAgICAgICAgICAgICBiYW5kd2lkdGggPSAxCikKCgpzY29yZXNfUDJYMyA9IGRhdGEuZnJhbWUoZGYudW1hcCkgIyBQQyBzY29yZSBtYXRyaXgKc2NvcmVzX1AyWDMkZ3JvdXAgPSBOYkNsdXN0KHNjb3Jlc19QMlgzLCBtZXRob2QgPSAna21lYW5zJykkQmVzdC5wYXJ0aXRpb24Kc2NvcmVzX1AyWDMkZ2VuZSA9IHJlcCgnUDJYMycsIG5yb3coc2NvcmVzX1AyWDMpKQoKU3VwcEZpZzJfZSA9IGdncGxvdChzY29yZXNfUDJYMywgYWVzKFgxLFgyLCBjb2xvciA9IGFzLmZhY3Rvcihncm91cCkpKSArCiAgZ2VvbV9wb2ludCgpICArIHhsYWIoJ1VNQVAxJykgKyB5bGFiKCdVTUFQMicpICsgZ2d0aXRsZSgnUDJYMycpCmBgYAoKI1N1cHBsZW1lbnRhcnkgRmlndXJlIDIgJiAxMiBmb3IgS3YxMwpgYGB7ciBTdXBwbGVtZW50YXJ5IEZpZ3VyZSAyICYgMTIgZm9yIEt2MTMsIG1lc3NhZ2U9RkFMU0UsIHdhcm5pbmc9RkFMU0V9CiNsb2FkIEt2MS4zCgojU3VwcEZpZzEyX2UgPSBnZ3Bsb3Qoa3YxMykgKwojICBnZW9tX3RpbGUoYWVzKHg9cG9zaXRpb24seT1kb21haW4sZmlsbD12YWx1ZSkpICsKIyAgc2NpY286OnNjYWxlX2ZpbGxfc2NpY28ocGFsZXR0ZSA9ICdiaWxiYW8nLCBuYS52YWx1ZSA9ICdjeWFuJykgKwojICBmYWNldF9ncmlkKGdlbmV+LikgKwojICB0aGVtZV9jbGFzc2ljKCkgKwojICBnZ3RpdGxlKCdLdjEzJykKCnogPSBkY2FzdChrdjEzXzE1RCwgZG9tYWluICsgZ2VuZSArIHBvc2l0aW9uIH4gdmFyaWFibGUsIGZ1bi5hZ2dyZWdhdGUgPSBmdW5jdGlvbih4KSBtZWFuKHgsIG5hLnJtID0gVCkpCnpbLCdub3JtYWxpemVkX2hpZ2gnXVt6Wywnbm9ybWFsaXplZF9oaWdoJ10gPT0gMF0gPSBOQQoKeiRwZXJtID0gcmVwKE5BLCBucm93KHopKQoKCmZvciAoaSBpbiBsZXZlbHMoeiRnZW5lKSl7CiAgaWR4ID0geiRnZW5lID09IGkKICB6W2lkeCwncGVybSddID0gcmFuZ2UwMSh6W2lkeCwnbm9ybWFsaXplZF9oaWdoJ10scmVtb3ZlLm5hID0gVCkKfQoKU3VwcEZpZzEyX2UgPSBnZ3Bsb3QoeikgKwogIGdlb21fdGlsZShhZXMoeD1wb3NpdGlvbix5PWRvbWFpbixmaWxsPW5vcm1hbGl6ZWRfaGlnaCkpICsKICBzY2ljbzo6c2NhbGVfZmlsbF9zY2ljbyhwYWxldHRlID0gJ2JpbGJhbycsIG5hLnZhbHVlID0gJ2N5YW4nKSArCiAgZmFjZXRfZ3JpZChnZW5lfi4pICsKICB0aGVtZV9jbGFzc2ljKCkgKwogIGdndGl0bGUoJ0t2MTMnKQoKa3YxM3ogPSB6CmNvbG5hbWVzKGt2MTN6KSA9IGMoJ2RvbWFpbicsJ2dlbmUnLCdwb3NpdGlvbicsJ25vcm1hbGl6ZWRfbG93QU5EaGlnaCcsJ3Blcm0nKQoKI0t2MTMjIyMjCgpkID0gelt6JGdlbmUgPT0gJ0t2MTMnLGMoJ2RvbWFpbicsJ3Bvc2l0aW9uJywncGVybScpXQoKCiNyZW1vdmUgZG9tYWlucyBhbmQgcG9zaXRpb25zIG1pc3NpbmcgODAlIGRhdGEKcGVybV9waXZvdCA9IGRjYXN0KGQscG9zaXRpb25+ZG9tYWluLHZhbHVlLnZhcj0ncGVybScpCnBlcm1fcGl2b3RfcGxvdCA9IHBlcm1fcGl2b3Rbcm93TWVhbnMoaXMubmEocGVybV9waXZvdCkpIDw9IC44LF0KcGVybV9waXZvdF9wbG90ID0gcGVybV9waXZvdF9wbG90Wyxjb2xNZWFucyhpcy5uYShwZXJtX3Bpdm90X3Bsb3QpKSA8PSAuOF0KCnBlcm1fcGxvdF9tZWx0ID0gbWVsdChwZXJtX3Bpdm90X3Bsb3QsaWQudmFycz0ncG9zaXRpb24nKQpwZXJtX3Bsb3RfbWVsdCA9IG5hLm9taXQocGVybV9wbG90X21lbHQpCmNvbG5hbWVzKHBlcm1fcGxvdF9tZWx0KSA9IGMoJ3Bvc2l0aW9uJywnZG9tYWluJywncGVybWlzc2liaWxpdHknKQoKcGVybV9waXZvdF96ZXJvcyA9IHBlcm1fcGl2b3RfcGxvdApwZXJtX3Bpdm90X3plcm9zW2lzLm5hKHBlcm1fcGl2b3RfemVyb3MpXSA9IDAKCiN1bWFwCmRmLnVtYXAgPSB1bWFwKHBlcm1fcGl2b3RfemVyb3NbLC0xXSwgCiAgICAgICAgICAgICAgICAgICAgIG1ldHJpYyA9ICdjb3NpbmUnLAogICAgICAgICAgICAgICAgICAgICBuX2Vwb2NocyA9IDUwMCwKICAgICAgICAgICAgICAgICAgICAgbl9uZWlnaGJvcnMgPSAyMCwKICAgICAgICAgICAgICAgICAgICAgbm5fbWV0aG9kID0gJ2Fubm95JywKICAgICAgICAgICAgICAgICAgICAgbl90cmVlcyA9IDEwMCwKICAgICAgICAgICAgICAgICAgICAgYmFuZHdpZHRoID0gMC44CikKCgoKc2NvcmVzX0t2MTMgPSBkYXRhLmZyYW1lKGRmLnVtYXApICMgUEMgc2NvcmUgbWF0cml4CnNjb3Jlc19LdjEzJGdyb3VwID0gTmJDbHVzdChzY29yZXNfS3YxMywgbWV0aG9kID0gJ3dhcmQuRDInKSRCZXN0LnBhcnRpdGlvbgpzY29yZXNfS3YxMyRnZW5lID0gcmVwKCdLdjEzJywgbnJvdyhzY29yZXNfS3YxMykpCgpTdXBwRmlnMl9mID0gZ2dwbG90KHNjb3Jlc19LdjEzLCBhZXMoWDEsWDIsIGNvbG9yID0gYXMuZmFjdG9yKGdyb3VwKSkpICsKICBnZW9tX3BvaW50KCkgKyB4bGFiKCdVTUFQMScpICsgeWxhYignVU1BUDInKSArIGdndGl0bGUoJ0t2MS4zJykKCmBgYAoKI1N1cHBsZW1lbnRhcnkgRmlndXJlIDIKYGBge3IgU3VwcGxlbWVudGFyeSBGaWd1cmUgMiwgZmlnLmhlaWdodD0xMCwgZmlnLndpZHRoPTgsIG1lc3NhZ2U9RkFMU0UsIHdhcm5pbmc9RkFMU0V9CmdnYXJyYW5nZShwbG90X2tpcjIxX3VtYXAsIFN1cHBGaWcyX2IsCiAgICAgICAgICBTdXBwRmlnMl9jLCBTdXBwRmlnMl9kLAogICAgICAgICAgU3VwcEZpZzJfZSwgU3VwcEZpZzJfZiwKICAgICAgICAgIG5yb3cgPSAzLCBuY29sID0gMiwKICAgICAgICAgIGxhYmVscyA9IGMoJ2EnLCdiJywnYycsJ2QnLCdlJywnZicpKQpgYGAKCiNTdXBwbGVtZW50YXJ5IEZpZ3VyZSAxMgpgYGB7ciBTdXBwbGVtZW50YXJ5IEZpZ3VyZSAxMiwgZmlnLmhlaWdodD0xMCwgZmlnLndpZHRoPTgsIG1lc3NhZ2U9RkFMU0UsIHdhcm5pbmc9RkFMU0V9CmdnYXJyYW5nZShTdXBwRmlnMTJfYiwgU3VwcEZpZzEyX2IsCiAgICAgICAgICBTdXBwRmlnMTJfYywgU3VwcEZpZzEyX2QsCiAgICAgICAgICBTdXBwRmlnMTJfZSwgU3VwcEZpZzEyX2YsCiAgICAgICAgICBucm93ID0gMywgbmNvbCA9IDIsCiAgICAgICAgICBsYWJlbHMgPSBjKCdhJywnYicsJ2MnLCdkJywnZScsJ2YnKSkKYGBgCgojU3VwcGxlbWVudGFyeSBGaWd1cmUgMTNhCmBgYHtyIFN1cHBsZW1lbnRhcnkgRmlndXJlIDEzYSwgZmlnLmhlaWdodD0zLCBmaWcud2lkdGg9MywgbWVzc2FnZT1GQUxTRSwgd2FybmluZz1GQUxTRX0KI3NwZWFybWFuIGNvcnJlbGF0aW9ucwoKZGYgPSByYmluZChhc2ljMWF6LCBraXIyMXosIGtpcjMxeiwga3YxM3osIHAyeDN6KQpkZiRkb21haW4gPSBhcy5mYWN0b3IoZGYkZG9tYWluKQpkZiRpbnQgPSBpbnRlcmFjdGlvbihkZiRnZW5lLCBkZiRkb21haW4pCmRmW2RmID09IDBdID0gTkEKZGZbaXMubmFuKGRmJHBlcm0pLCdwZXJtJ10gPSBOQQoKZGZfcGl2b3QgPSBkY2FzdChkZiwgZG9tYWlufmdlbmUsIHZhbHVlLnZhciA9ICdwZXJtJywgZnVuLmFnZ3JlZ2F0ZSA9IGZ1bmN0aW9uKHgpe21lYW4oeCwgbmEucm0gPSBUKX0pICNTdXBwRmlnMTMKCmRmX2NvciA9IGNvcihkZl9waXZvdFssLTFdLCBtZXRob2QgPSAnc3BlYXJtYW4nLCB1c2UgPSAncGFpcndpc2UuY29tcGxldGUub2JzJykKCmMxID0gY29yLm10ZXN0KGRmX3Bpdm90WywtMV0pCgpjb3JycGxvdChkZl9jb3IsIHR5cGUgPSAidXBwZXIiLCBvcmRlciA9ICJoY2x1c3QiLAogICAgICAgICAgICAgICAgICAgaGNsdXN0Lm1ldGhvZCA9ICd3YXJkLkQyJywKICAgICAgICAgICAgICAgICAgIHRsLmNvbCA9ICJibGFjayIsIHRsLnNydCA9IDQ1LAogICAgICAgICAgICAgICAgICAgcC5tYXQgPSBjMSRwLCBzaWcubGV2ZWwgPSAwLjIpCgoKCmBgYAoKI1N1cHBsZW1lbnRhcnkgRmlndXJlIDEzYi1lCmBgYHtyIFN1cHBsZW1lbnRhcnkgRmlndXJlIDEzYi1lLCBmaWcuaGVpZ2h0PTQsIGZpZy53aWR0aD00LCBtZXNzYWdlPUZBTFNFLCB3YXJuaW5nPUZBTFNFfQoKcDIgPSBnZ3NjYXR0ZXIoZGZfcGl2b3QsIHggPSAnS2lyMjEnLCB5PSAnS2lyMzEnLAogICAgICAgICAgICAgICAgICBhZGQgPSAncmVnLmxpbmUnLCBjb25mLmludCA9IFQsCiAgICAgICAgICAgICAgICAgIGNvci5jb2VmID0gVCwgY29yLm1ldGhvZCA9ICdwZWFyc29uJywKICAgICAgICAgICAgICAgICAgeGxhYiA9ICdLaXIyMScsIHlsYWIgPSAnS2lyMzEnKQoKcDMgPSBnZ3NjYXR0ZXIoZGZfcGl2b3QsIHggPSAnS2lyMjEnLCB5PSAnS3YxMycsCiAgICAgICAgICAgICAgICAgIGFkZCA9ICdyZWcubGluZScsIGNvbmYuaW50ID0gVCwKICAgICAgICAgICAgICAgICAgY29yLmNvZWYgPSBULCBjb3IubWV0aG9kID0gJ3BlYXJzb24nLAogICAgICAgICAgICAgICAgICB4bGFiID0gJ0tpcjIxJywgeWxhYiA9ICdLdjEzJykKCnA0ID0gZ2dzY2F0dGVyKGRmX3Bpdm90LCB4ID0gJ0FzaWMxYScsIHk9ICdLdjEzJywKICAgICAgICAgICAgICAgICAgYWRkID0gJ3JlZy5saW5lJywgY29uZi5pbnQgPSBULAogICAgICAgICAgICAgICAgICBjb3IuY29lZiA9IFQsIGNvci5tZXRob2QgPSAncGVhcnNvbicsCiAgICAgICAgICAgICAgICAgIHhsYWIgPSAnQXNpYzFhJywgeWxhYiA9ICdLdjEzJykKCnA1ID0gZ2dzY2F0dGVyKGRmX3Bpdm90LCB4ID0gJ0FzaWMxYScsIHk9ICdLaXIzMScsCiAgICAgICAgICAgICAgICAgIGFkZCA9ICdyZWcubGluZScsIGNvbmYuaW50ID0gVCwKICAgICAgICAgICAgICAgICAgY29yLmNvZWYgPSBULCBjb3IubWV0aG9kID0gJ3BlYXJzb24nLAogICAgICAgICAgICAgICAgICB4bGFiID0gJ0FzaWMxYScsIHlsYWIgPSAnS2lyMzEnKQoKZ2dhcnJhbmdlKHAyLHAzLHA0LHA1LAogICAgICAgICAgbmNvbCA9IDIsIG5yb3cgPSAyLAogICAgICAgICAgbGFiZWxzID0gYygnYicsJ2MnLCdkJywnZScpKQpgYGAKCiNTdXBwbGVtZW50YXJ5IEZpZ3VyZSAxNApgYGB7ciBTdXBwbGVtZW50YXJ5IEZpZ3VyZSAxNCwgZmlnLmhlaWdodD02LCBmaWcud2lkdGg9NiwgbWVzc2FnZT1GQUxTRSwgd2FybmluZz1GQUxTRX0KZGZfcGl2b3QgPSBkY2FzdChkZiwgcG9zaXRpb25+aW50LCB2YWx1ZS52YXIgPSAncGVybScsIGZ1bi5hZ2dyZWdhdGUgPSBmdW5jdGlvbih4KXtzdW0oeCwgbmEucm0gPSBUKX0pCmRmX2NvciA9IGNvcihkZl9waXZvdFssLTFdLCBtZXRob2QgPSAnc3BlYXJtYW4nLCB1c2UgPSAncGFpcndpc2UuY29tcGxldGUub2JzJykKCmMxID0gY29yLm10ZXN0KGRmX3Bpdm90WywtMV0pCgpjb3JycGxvdChkZl9jb3IsIHR5cGUgPSAidXBwZXIiLCBvcmRlciA9ICJoY2x1c3QiLAogICAgICAgICAgICAgICAgICAgaGNsdXN0Lm1ldGhvZCA9ICd3YXJkLkQyJywKICAgICAgICAgICAgICAgICAgIHRsLmNvbCA9ICJibGFjayIsIHRsLnNydCA9IDQ1LAogICAgICAgICAgICAgICAgICAgcC5tYXQgPSBjMSRwLCBzaWcubGV2ZWwgPSAwLjIpCmBgYAo=
