# Supplementary material for: Probing ion channel functional architecture and domain recombination compatibility by massively parallel domain insertion profiling
Source: Nat Commun. 2021 Dec 8;12:7114. doi: 10.1038/s41467-021-27342-0 (PMC8654947; doi:10.1038/s41467-021-27342-0)
Supplement: Supplementary file 10 — Reporting summary [file 41467_2021_27342_MOESM10_ESM.pdf]

nature portfolio

Corresponding author(s): Daniel Schmidt

Last updated by author(s): Nov 8, 2021

Reporting Summary

Nature Portfolio wishes to improve the reproducibility of the work that we publish. This form provides structure for consistency and transparency in reporting. For further information on Nature Portfolio policies, see our Editorial Policies and the Editorial Policy Checklist.

Statistics

For all statistical analyses, confirm that the following items are present in the figure legend, table legend, main text, or Methods section.

n/a	Confirmed	
☐	☒	The exact sample size (*n*) for each experimental group/condition, given as a discrete number and unit of measurement
☐	☒	A statement on whether measurements were taken from distinct samples or whether the same sample was measured repeatedly
☐	☒	The statistical test(s) used AND whether they are one- or two-sided<br>*Only common tests should be described solely by name; describe more complex techniques in the Methods section.*
☒	☐	A description of all covariates tested
☒	☐	A description of any assumptions or corrections, such as tests of normality and adjustment for multiple comparisons
☐	☒	A full description of the statistical parameters including central tendency (e.g. means) or other basic estimates (e.g. regression coefficient) AND variation (e.g. standard deviation) or associated estimates of uncertainty (e.g. confidence intervals)
☐	☒	For null hypothesis testing, the test statistic (e.g. *F*, *t*, *r*) with confidence intervals, effect sizes, degrees of freedom and *P* value noted<br>*Give P values as exact values whenever suitable.*
☒	☐	For Bayesian analysis, information on the choice of priors and Markov chain Monte Carlo settings
☒	☐	For hierarchical and complex designs, identification of the appropriate level for tests and full reporting of outcomes
☐	☒	Estimates of effect sizes (e.g. Cohen's *d*, Pearson's *r*), indicating how they were calculated

Our web collection on statistics for biologists contains articles on many of the points above.

Software and code

Policy information about availability of computer code

Data collection	Flow data was collected using FACSDiva v.8.0.1
Data analysis	The SPINE code is available at: https://github.com/schmidt-lab/spine. The code for handling data from domain insertion library sequencing is available at: https://github.com/SavageLab/dipseq. Enrichment calculation was based on Enrich2 and implemented in R version 4.1.0. Mapping data onto crystal structures was done using UCSF Chimera, v 1.16 (build 42330). Further numerical data analysis (correlation, Random Forest models, etc.) was done using R version 4.1.0. Flow cytometry analysis was done in FlowJo 10. Molecular dynamics simulations were carried out using the charmm36 force field in NAMD2.12. Property calculations were done in Python 2.7.16. Sequence-based motifs properties were calculated using the Quantiprot python package (version 0.2.4). Protein structural properties were calculated with python code provided by Alexander Golinski and Benjamin Hackel (University of Minnesota). Scripts to reproduce manuscript figures are available as Supplementary Data 6.

For manuscripts utilizing custom algorithms or software that are central to the research but not yet described in published literature, software must be made available to editors and reviewers. We strongly encourage code deposition in a community repository (e.g. GitHub). See the Nature Portfolio guidelines for submitting code & software for further information.

Data

Policy information about availability of data

All manuscripts must include a data availability statement. This statement should provide the following information, where applicable:

- Accession codes, unique identifiers, or web links for publicly available datasets
- A description of any restrictions on data availability
- For clinical datasets or third party data, please ensure that the statement adheres to our policy

Sequencing data generated in this study have been deposited in the Sequence Raw Archive (https://www.ncbi.nlm.nih.gov/sra) under accession codes PRJNA766040 (Project_047) and PRJNA766074 (Project_045); refer to Supplementary Table 6 for corresponding metadata. Source data are provided with this paper: Processed data (z-scored surface trafficking scores) are available as Supplementary Data 3. Calculated inserted motif and recipient protein properties are available as Supplementary Data 4 & 5. Additional source data (machine learning model, raw data of manuscript figures) are deposited on Zenodo (https://doi.org/10.5281/zenodo.5683566). All structural models are available at the Protein Data Bank (https://www.rcsb.org) under accession codes 3SPI (PIP2-bound Kir2.2), 3JYC (apo state Kir2.2), 4KFM (Kir3.2), 2R9R (Kv1.2/Kv2.1 paddle chimera), 5SVK (P2X3), and 6AVE (Asic1a). Primary sequences of all channels used in this study are available at https://www.uniprot.org under accession codes P35561 (mouse Kir2.1), P63250 (mouse Kir3.1), P48542 (mouse Kir3.2), P78348 (human Asic1a), P56373 (human P2X3), P22001 (human Kv1.3). Supplementary Data 1 & 2 contain inserted domain and target channel sequences, respectively. Together these are the minimal set of data required to replicate the analysis. All data are available without restriction.

Field-specific reporting

Please select the one below that is the best fit for your research. If you are not sure, read the appropriate sections before making your selection.

- [x] Life sciences
- [ ] Behavioural & social sciences
- [ ] Ecological, evolutionary & environmental sciences

For a reference copy of the document with all sections, see nature.com/documents/nr-reporting-summary-flat.pdf

Life sciences study design

All studies must disclose on these points even when the disclosure is negative.

Sample size	Our study determines surface-trafficking phenotypes of the over 300,000 domain recombination variants; this is the largest study of this type to date. To produce a rich dataset from which these potential insights and improvement can be sourced, we generated 759 polypeptide motif (donor) insertions at all 435 amino acids of the Inward Rectifier K+ channel Kir2.1 (recipient) and measured cell surface expression of the resulting channel / insertion variants. Previously, we found surprising variability between three motif's insertional profiles, which implies complex constraints on donor-recipient compatibility (Coyote-Maestas et al. Nature Communications (2019)). We chose 759 donor motifs as a representative sample to exhaustively study compatibility (see Supp. Table 1 for polypeptide motif sources, see Supp. Table 2 for summary statistic of biophysical properties).
Data exclusions	Forward and reverse reads were aligned individually using a DIP-seq pipeline, slightly modified for SPINE compatibility and for updated python packages. If both forward and reverse reads report an insertion, duplicated domain insertion calls are removed to avoid artificially boosting counts.<br><br>Enrichment scores for each replicate were calculated as described in the Material and Methods. Replicates were combined by a weighted average, which was calculated by a restricted maximum likelihood estimate and standard error using 50 Fisher scoring iterations. Position with data missing in either replicate are treated as NA and are not considered in further analysis (exclusion criteria), except for correlations between datasets as removing data adds more noise than treating NAs as 0s due to sampling.<br><br>Inserting 759 motifs into 435 Kir2.1 positions yields a total theoretical library diversity of 327,888 variants. Each sub-pooled library we generated and screened encompassed 12,500 variants. Due to random variance, some datasets were incomplete (Fig. 1b-e). To make downstream analysis more robust, we only included motifs with data (after exclusion criteria outlined in Enrichment Calculations) in >80% of positions. This left us with 637 out of 759 motifs (further details in Supp. Table 1).
Replication	All raw data collections were performed with a least two biological / independent replicates which is a commonly accepted standard (see for example Starr et al Cell. 2020 Sep 3; 182(5): 1295–1310.e20. "Deep mutational scanning of SARS-CoV-2 receptor binding domain reveals constraints on folding and ACE2 binding" and Rubin et al., "A statistical framework for analyzing deep mutational scanning data" Genome Biology (2017) 18:150.)
Randomization	Randomization was not relevant to this study as samples are not divided into experimental groups.
Blinding	Experimenters were not blinded; it seemed not necessary. As this was a discovery-driven project, and in the absence of a theory to be disproved, there wasn't any bias one way or another.

Reporting for specific materials, systems and methods

We require information from authors about some types of materials, experimental systems and methods used in many studies. Here, indicate whether each material, system or method listed is relevant to your study. If you are not sure if a list item applies to your research, read the appropriate section before selecting a response.

Materials & experimental systems

n/a	Involved in the study	
☐	☒	Antibodies
☐	☒	Eukaryotic cell lines
☒	☐	Palaeontology and archaeology
☒	☐	Animals and other organisms
☒	☐	Human research participants
☒	☐	Clinical data
☒	☐	Dual use research of concern

Methods

n/a	Involved in the study	
☒	☐	ChIP-seq
☐	☒	Flow cytometry
☒	☐	MRI-based neuroimaging

Antibodies

Antibodies used	Brilliant Violet 421™ anti-DYKDDDDK Tag Antibody, Clone: L5, Isotype: IgG2a, BioLegend, catalog# 637321, lot#B281782, dilution 1:200.
Validation	Antibody was validated by flow cytometry using mock-transfected HEK293 (negative control) and HEK293 transfected with Kir2.1-FLAG (positive control; FLAG tag inserted into an extracelullar loop). Kir2.1 variant in which the inserted domain is disrupting the FLAG tag are additional internal negative controls (see Fig. 2a, yellow box).

Eukaryotic cell lines

Policy information about cell lines

Cell line source(s)	TetBxB1BFP-iCasp-Blast Clone 12 HEK293T cells (gift from Douglas Fowler, University of Washington). This cell line is based on HEK293T (ThermoFisher Scientific).
Authentication	Not authenticated.
Mycoplasma contamination	Not tested for mycoplasma.
Commonly misidentified lines (See ICLAC register)	No commonly misidentified cell lines were used.

Flow Cytometry

Plots

Confirm that:

☒ The axis labels state the marker and fluorochrome used (e.g. CD4-FITC).

☒ The axis scales are clearly visible. Include numbers along axes only for bottom left plot of group (a 'group' is an analysis of identical markers).

☒ All plots are contour plots with outliers or pseudocolor plots.

☒ A numerical value for number of cells or percentage (with statistics) is provided.

Methodology

Sample preparation	Sample preparation is described in the Material and Methods ("Sequencing-based surface expression assay").
Instrument	BD FACSAria II P69500132 and BD Fortessa H0081 flow cytometer
Software	FACSDIVA v8.0.1
Cell population abundance	For larger pooled sublibrary samples, we collected between at least 100,000 to 500,00 cells per gate which is ~8-35x coverage. 15,000 cells in both gates of a Kir2.1 library with a small flexible ASGASGA linker was collected each day to normalize all the pooled libraries. For smaller 15 motifs samples, we collected between 4,000-50,000 of each sample/library pair which is ~10-120x coverage for all libraries.
Gating strategy	Gating strategies are described in the Material and Methods, and in Supp. Fig. 16-20.

☒ Tick this box to confirm that a figure exemplifying the gating strategy is provided in the Supplementary Information.
